# No Sustained Attention Differences in a Longitudinal Randomized Trial Comparing Mindfulness Based Stress Reduction versus Active Control

**DOI:** 10.1371/journal.pone.0097551

**Published:** 2014-06-23

**Authors:** Donal G. MacCoon, Katherine A. MacLean, Richard J. Davidson, Clifford D. Saron, Antoine Lutz

**Affiliations:** 1 Waisman Center for Brain Imaging and Behavior, University of Wisconsin–Madison, Madison, Wisconsin, United States of America; 2 Center for Investigating Healthy Minds at the Waisman Center, University of Wisconsin–Madison, Madison, Wisconsin, United States of America; 3 Department of Psychology, University of Wisconsin–Madison, Madison, Wisconsin, United States of America; 4 Department of Psychiatry and Behavioral Sciences, Johns Hopkins University School of Medicine, Baltimore, Maryland, United States of America; 5 Center for Mind and Brain, University of California Davis, Davis, California, United States of America; 6 The Medical Investigation of Neurodevelopmental Disorders Institute, University of California Davis, Davis, California, United States of America; 7 INSERM U1028, CNRS UMR5292, Lyon Neuroscience Research Center, Brain Dynamics and Cognition Team, Lyon, France; 8 Lyon 1 University, Lyon, France; Carnegie Mellon University, United States of America

## Abstract

**Background:**

Mindfulness Based Stress Reduction (MBSR) is a secular form of meditation training. The vast majority of the extant literature investigating the health effects of mindfulness interventions relies on wait-list control comparisons. Previous studies have found that meditation training over several months is associated with improvements in cognitive control and attention.

**Methodology/Principal Findings:**

We used a visual continuous performance task (CPT) to test the effects of eight weeks of mindfulness training on sustained attention by comparing MBSR to the Health Enhancement Program (HEP), a structurally equivalent, active control condition in a randomized, longitudinal design (ClinicalTrials.gov, NCT01301105) focusing on a non-clinical population typical of MBSR participants. Researchers were blind to group assignment. 63 community participants were randomized to either MBSR (n = 31) or HEP (n = 32). CPT analyses were conducted on 29 MBSR participants and 25 HEP participants. We predicted that MBSR would improve visual discrimination ability and sustained attention over time on the CPT compared to HEP, with more home practice associated with greater improvements. Our hypotheses were not confirmed but we did find some evidence for improved visual discrimination similar to effects in partial replication of other research. Our study had sufficient power to demonstrate that intervention groups do not differ in their improvement over time in sustained attention performance. One of our primary predictions concerning the effects of intervention on attentional fatigue was significant but not interpretable.

**Conclusions:**

Attentional sensitivity is not affected by mindfulness practice as taught in MBSR, but it is unclear whether mindfulness might positively affect another aspect of attention, vigilance. These results also highlight the relevant procedural modifications required by future research to correctly investigate the role of sustained attention in similar samples.

**Trial Registration:**

ClinicalTrials.gov, NCT01301105

## Introduction

Introduced more than 20 years ago [1], Mindfulness-Based Stress Reduction (MBSR; [2]) provides a secular form of training in mindfulness meditation for stress reduction and emotion regulation. Mindfulness is a broad term that can encompass many different meditation practices and cognitive skills, including skills conceptualized as involving attention regulation and response inhibition [3]. In the context of MBSR, Kabat-Zinn embraced a broad view of mindfulness which he has defined as “paying attention in a particular way: on purpose, in the present moment, and non-judgmentally” [4], p. 4) and included a variety of attention, emotional, and interoceptive techniques with clinical efficacy (for a discussion of clinical effects, see [5]), such as yoga, body-focused practices, and walking and sitting meditation involving compassion, self-acceptance, and breath awareness.

Reflecting a trend toward more detailed and mechanistic accounts of MBSR – and meditation training more generally – some authors have more rigorously explicated each of these latter three components [6]. On the other hand, traditional Buddhist accounts emphasize and clarify different aspects of meditation, including focused attention meditation, which involves moment by moment selective attention focused on a particular object (e.g., sensations associated with the breath), and open monitoring or open presence meditation, which involves an open awareness of any stimuli that occur in the present moment and a subsequent shift away from a steady focus on one particular object [7]. Though a source of some debate (see [3]), the mindfulness meditation taught in MBSR is likely to include aspects of both types of meditation. The current study is part of this trend toward a focus on mechanism generally, and sustained attention in particular.

Sustained attention has been found to be related to intensive meditation practice [8], [9]. For instance, three months of intensive vipassana practice led to reductions in trial-by-trial variability in reaction times in a sustained attention task, a standard behavioral marker of attention stability [8]. MacLean and colleagues [9] found meditation-related improvements on a continuous performance task (CPT) with participants randomly assigned to a three-month residential focused attention meditation training program (retreat 1) or the wait-list control condition (who then went on to do their own three-month retreat 2). After retreat 1, individuals in the meditation training group, but not those in the wait-list control group, showed improvements in visual discrimination (ability to discriminate targets from non-targets), but not improvements in vigilance (performance over time). In retreat 2, vigilance was related to visual discrimination: with visual threshold settings held constant from pre to post retreat, individuals in retreat 2 showed improvements in both visual discrimination and improvements in sustained performance over time on the CPT. The overall pattern of results from the Lutz et al. [2] and MacLean et al. [3] studies suggests that meditation training can improve both visual perceptual sensitivity and sustained attention.

Furthermore, there are theoretical reasons to suggest that sustained attention may be trained in MBSR specifically. As clarified by Lutz and colleagues [7], Mindfulness meditation is a form of attentional control training by which individuals first develop the ability to direct and maintain attention towards a chosen object. To this end, mindfulness practice requires skills involved in monitoring the focus of attention and in detecting distraction, disengaging attention from the source of distraction, and flexibly (re)directing and engaging attention to the intended object. In addition to this training of attention, mindfulness meditation cultivates the skill to maintain a non-judgmental, open presence to the present moment. This form of meta-awareness consists of non-reactively monitoring the content of experience from moment-to-moment without being carried away by thoughts, emotions, or perceptions. While MBSR seems to focus more on the second aspect of mindfulness, it still offers exercises that aim at strengthening attention control (e.g. breath awareness meditation). It is thus plausible that MBSR affects sustained attention, even if it may not be the dominant cognitive mechanism of action of MBSR training. In addition, the formal practice of developing mindful attention is carried out over periods of time similar to the sustained attention task used in this study – 24 minutes – and therefore what is cultivated during the formal practice of mindfulness could reasonably be conjectured to transfer to such a task. Finally, “sustained attention” as measured in CPT-type tasks reflects the transient and repeated application of the attentional skills of detecting distraction and reorienting attention and is not, per se, “sustained” in the sense of absorption on an object in an unwavering sense. This is another reason that the CPT-type task is a good candidate for measuring changes in attention associated with mindfulness meditation.

These theoretical and empirical issues raise the possibility that sustained attention may be an active ingredient in less intensive clinical interventions with meditation-naïve populations. A recent review of 23 meditation studies with adult samples and neuropsychological measures [10] revealed mixed effects of mindfulness training on different aspects of attention. However, only three of the reviewed studies were randomized controlled trials involving eight-week interventions (MBSR or Mindfulness-Based Cognitive Therapy) and each of them used a wait-list condition as their control [11]–[13]. Two studies focused on autobiographical memory and meta-awareness, finding group differences in favor of MBCT [12], [13]. A third study [11] found no attention differences between their MBSR and wait-list control groups in sustained attention as measured by the Vigil Continuous Performance Test (The Psychological Corporation), a task involving the identification of a target letter (e.g., “K”) in a series of other letters.

This literature raises two issues that should be resolved. First, there are inconsistent findings pointing to improvements in various aspects of attention. Second, a significant weakness inherent in this (and other clinical) research investigating mindfulness-based trainings is the lack of a good control condition. To address these issues, the present study uses a randomized, controlled, longitudinal design (see [5]), focuses on sustained attention, using the identical CPT as the MacLean et al study [9] (retreat 1), and running concurrently with that study. Study participants completed their lab assessments from August, 2006 to April, 2007. One potential benefit of using the same task as MacLean et al is that we can gain some insight into the importance of training dosage on attention effects since our study uses 8-week interventions while theirs uses a 3-month intervention.

Second, we also used an active control condition, the Health Enhancement Program (HEP), an intervention designed to isolate mindfulness as a testable active ingredient while addressing the three major limitations typical of active controls in behavioral intervention research: researcher allegiance, structural equivalence, and factors common to any effective group intervention [5]. This approach allowed us to test the effects of a less intensive dose of meditation of more relevance to a typical population on the identical task used by MacLean.

Using this framework, and consistent with some of the extant literature, we hypothesized that MBSR would improve measures of sustained attention compared to HEP and that home practice would be associated with improvements in attention in MBSR relative to HEP.

## Methods

The protocol for this trial and supporting CONSORT checklist are available as supporting information; see Checklist S1 and Protocol S1.

### Ethics Statement

All study and task details were approved by the UW-Madison Health Sciences Internal Review Board. Participants provided written informed consent for all study procedures.

### Participants

**Figure 1:**
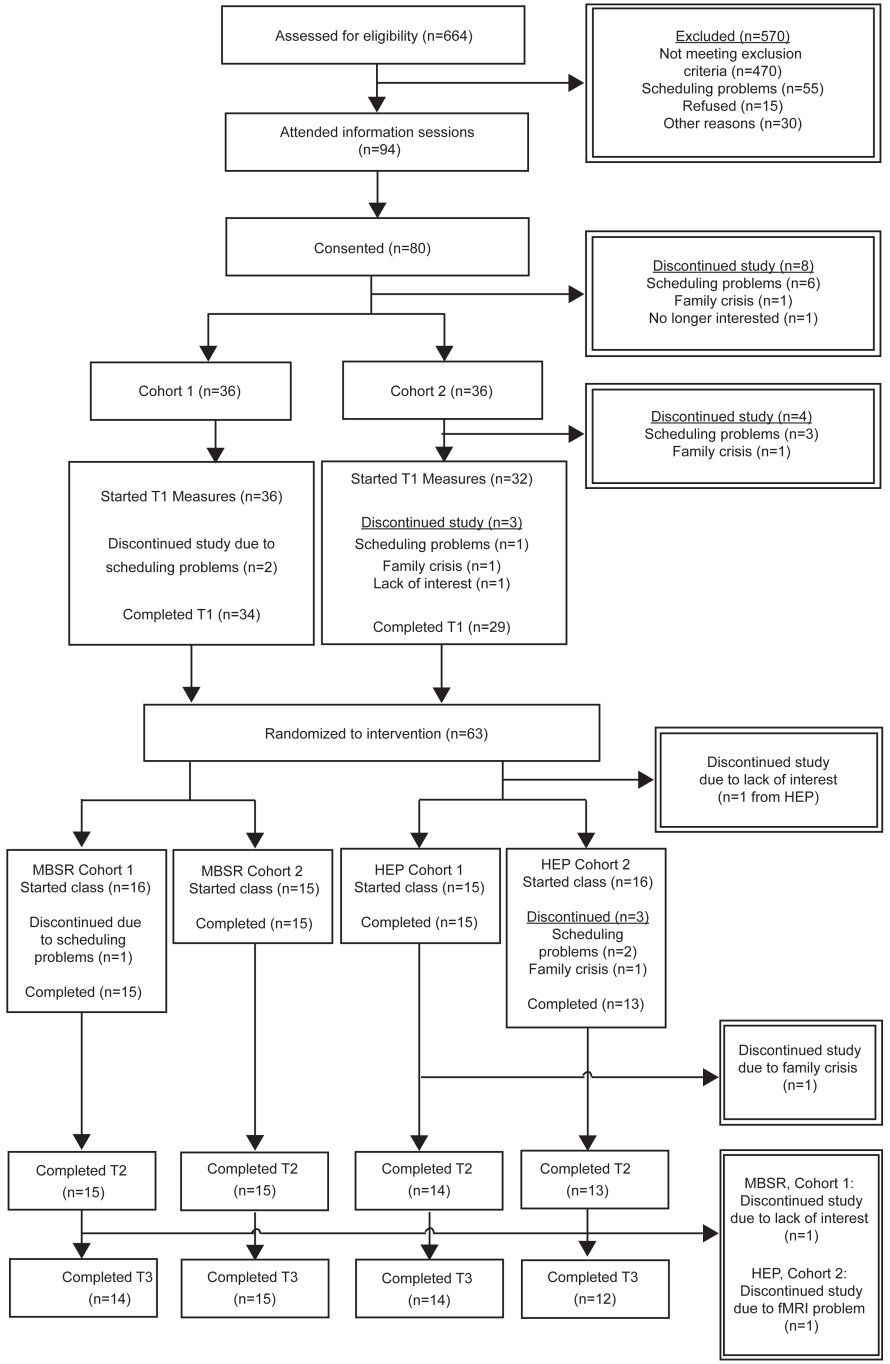
CONSORT diagram detailing retention rates by study phase and reasons for dropouts.

Participants were recruited as part of a larger study comparing MBSR and HEP (see [5] for details). In brief, participants were recruited for a study on “health and well-being” and offered $475 plus a free “8 week Health Enhancement Program” or a free “8 week Mindfulness-Based Stress Reduction Class”. After telephone screening, 94 people attended one of four information sessions in which the study was described by project scientists, the classes were described by instructors, written consent was obtained, and lab visits were scheduled. Participants were organized into two cohorts based on schedules and class size restrictions, and members of each cohort were randomized to intervention by a logistical staff member through a random-number generator at the time of assignment, and underwent identical procedures separated by approximately 4 weeks. Our sample size was comparable to other MBSR studies and power analyses based on effect sizes reported in the literature. Participants were masked to research questions and researchers were masked to intervention assignment throughout data collection. As part of our effort to maximize the potential effectiveness of both interventions, our inclusion/exclusion criteria (see Table 1) included elements important for both MBSR and HEP. For example, just as we required inexperience in meditation practice prior to participating in MBSR, we required relative inexperience in HEP components as well (e.g., excluding participants with “Engagement in moderate sport and recreational activities more than 5 times a week.”). Data were collected and analyzed at UW-Madison. All analyses reported are based on participants who started and completed their intervention. There was no significant difference in drop-out between MBSR (1 person dropped, 3.2%) and HEP [3 people dropped, 9.7%; *F*(1,60) = 1.05, n.s.]. Reasons for dropping, discussions with participants, and dates of drop suggest random life events as the cause (see Figure 1; see Table 2 for participant demographic information).

**Table 1:** Inclusion/Exclusion Criteria.

Inclusion criteria
Able to lie still in the scanner for 90 minutes
Meets MRI safety standards
Weighs under 300 pounds
18–65 years old
Right-handed
No previous experience with meditation.
No daily practice with other mind-body techniques (e.g., yoga, tai-chi, but previous exposure to yoga is okay)
In good general health as determined by the investigator
Able to walk
Able to understand and speak English
Able to provide written consent prior to admission
Able to see without glasses (as if looking through binoculars)

Exclusion Criteria
Diabetes
Peripheral vascular disease, peripheral arterial disease, Raynaud's disease, or any other
diagnosed circulatory disorders
Body mass index (BMI) below 18.5
Any involuntary motor disorders
Allergic to adhesive tape
A history of problems of any kind during blood draws or needle phobia
2 or more of the following: Diagnosed hypertension, Hyperlipidemia, High cholesterol, Obesity, Smoke cigarettes, Family history of coronary or atherosclerotic disease (parents/siblings prior to age 55)
Current medical disorders that might make interpretation of scan data difficult
Has a problem with alcohol or non prescription drugs
Currently uses or plans to start medications that affect CNS function, including psychotropics, opiate medication or corticosteroids, during the last 3 months (including medications for anxiety, depression, or other psychological problems)
Takes inhaled steroids for asthma? (e.g., Fluticasone)
Takes any corticosteroids
Night shift workers (11 pm to 7 am) d/t potential disruption of cortisol level variability
Diabetes requiring insulin treatment
Has TMJ (Temporal Mandibular Joint) disorder or other problems with biting/chewing
Previous training in meditation
Currently meditates on a regular basis
Daily yoga, tai-chi, or Qigong practice
Engagement in moderate sport and recreational activities more than 5 times a week
Engagement in vigorous sport and recreational activities more than 4 times a week
Not able to attend an informational session, all class meetings, and all clinic visits

**Table 2:** Demographics by intervention.

	Gender	Age		Ethnicity			
Intervention	n (% female)	*M* (*SD*)	Range	Caucasian	AA	Asian	Indian
HEP (n = 27)	23 (85.2%)	47.5 (9.3)	19–59	23 (85.2%)	3 (11.1%)	1 (3.7%)	0 (0.0%)
MBSR (n = 30)	24 (80.0%)	44.5 (12.2)	21–59	24 (80.0%)	1 (3.3%)	2 (6.5%)	2 (6.7%)

AA = African American.

### Interventions

Both HEP and MBSR were structurally equivalent, having a group format, and meeting once a week for 2.5 hours (3 hours for first and last sessions) for 8 weeks with an “all day” component (9 a.m. to 4 p.m.) after week 6. Furthermore, all participants were asked by their instructors to complete 45 minutes of practice at home for 6 of 7 days each week. Analyses detailed in MacCoon, et al. (2012) indicated no group differences in variables relevant to structural equivalence, including drop status, number of classes attended, length of time spent in class, or time spent on home practice (on average, 1849 min of homework were completed during an 8-week class, about 44 min of for 6 of 7 days per week compared to 45 min assigned). The only exception to this was a group difference in number of sessions of home practice completed where HEP practiced more often than MBSR (*M* = 95.60 and *M* = 61.19 sessions respectively).

The content of the HEP intervention met the following criteria: (1) class activities were chosen to match MBSR activities as closely as possible (see Table 3), (2) these activities represented valid, active, therapeutic ingredients in their own right, and (3) these ingredients did not include mindfulness. Thus, the purpose of walking in MBSR is to cultivate awareness in movement, whereas the purpose of walking in HEP is the benefits of the physical activity for cardiovascular training and followed recommendations from the Centers for Disease Control regarding intensity and frequency of physical activity [14]. Similarly, the purpose of yoga in MBSR is largely to cultivate nonjudgmental awareness of physical sensations and respecting one's own physical limits as they change over time. In contrast, the purpose of the balance, posture, and agility exercises in HEP's functional movement is to augment one's physical strength, balance, agility and joint mobility resulting in a physically more resilient individual less prone to sustain injury from spontaneous or unpredictable events (e.g., tripping on a curb, slipping on icy ground, lifting a heavy object; e.g., [15], [16]). The music therapy component included an exercise that matched the body scan in several ways with a primary difference being the importance of the music as the change agent rather than MBSR's emphasis on awareness of one's own internal states. The nutrition component included didactic material and reading, both modalities used in MBSR but the content was not related to mindfulness. For further details about the two interventions, see [5].

**Table 3:** Intervention content comparison.

MBSR		HEP	
In-Class	Homework	In-Class	Homework
Body Scan	Body Scan and light reading	Music Therapy: Relax, listen to music, imagery, and drawing	Relax, listen to music, imagery, and drawing
Sitting Meditation	Body Scan, Sitting Meditation, and light reading	Nutrition Education around Food Guide Pyramid	Planning meals, tracking diet, food labels, journaling
Yoga	Alternate Yoga and Body Scan, and Sitting Meditation	Functional Movement (posture, balance, core movement)	Posture, balance, coordinated movement
Walking Meditation	Walking and other practices	Physical Activity (walk/jog, stretch)	Walking and stretching
All Day (7 hours): Work with all practices, Group discussion & exercises	–	“Spa Day” (7 hours): Work with all practices, Group discussion & exercises	–

### Procedure

Participants were asked to complete laboratory visits before the classes began (and before random assignment was completed; T1), after the classes ended (T2), and approximately 4 months following completion of the classes (T3). The CPT was administered during these laboratory visits. Task stimuli, instructions and administration procedures were identical to those used by MacLean and colleagues [9] in their first study (retreat 1) except where noted.

The first, and possibly most important deviation, was the fact that in our study participants were tested at T1 prior to being randomized to group whereas in the MacLean et al. study, participants knew their group assignment prior to being tested. Specifically, McLean et al. randomly assigned participants to a wait-list or retreat group. Both groups were assessed pre, mid, and post a 3-month retreat (retreat 1). When this was complete, the wait-list group was assessed again pre, mid, and post their 3-month retreat (retreat 2).

Participants in our study were tested in a quiet, darkened room using Presentation software (Neurobehavioral Systems, available from http://www.neurobs.com) and a Windows laptop computer. At each assessment, participants completed a threshold procedure (∼10 min) followed by a 24-min CPT (although this was 8 min shorter than the task used by MacLean et al, the analysis in MacLean et al focused on training-related improvements in vigilance that were evident within the first 16 min of task performance). In both tasks, participants saw a single vertical line appear at the center of the screen. This line was either a frequent (90%) long non-target or a rare (10%) short target. Stimuli appeared for 150 ms, followed by a mask of dotted lines presented during the variable interstimulus interval of 1550 to 2150 ms. Speed and accuracy of responding was emphasized and participants responded by pressing a key on the computer keyboard whenever a target appeared.

In the threshold procedure, the length of the target line varied according to a parameter estimation (PEST) algorithm [17], which determined the target line length that participants could correctly detect at 75% accuracy. The length of the target on the CPT was set to each participant's threshold at each time point (T1 through T3).

As in MacLean et al (2010) [9], a nonparametric index of perceptual sensitivity (A′, [18]), was calculated from hit (correct response to a target) and false alarm (incorrect response to a non-target) rates for each of six continuous 120-trial blocks during the CPT. Improvements in vigilance were operationalized as positive (or less negative) changes in the slope of A′ during the first four blocks of the task.

Participants with hit rates below 50% in block 1 of the CPT (indicating lack of successful thresholding to 75% accuracy) were removed (*n* = 5) from analyses. In addition, analyses were conducted with and without participants who had outlier (>1.5 *SD*) A′ values for any block relative to their mean A′ for that timepoint. Because results were similar with and without these outliers, analyses reported here include those participants. Data with RTs greater than 2 *SD* from participant's own mean for each task half were removed. Furthermore, outliers were removed based on boxplot graphs of A′ for the entire task and for first and second half of the task for each time point. One participant was removed as an extreme outlier for Time 1 A′. Another was removed as the only participant with outlier A′ data for both halves at Time 2. Finally, two participants were removed from analyses who had extreme outlier scores on target threshold at any time point. Thus, there were 29 MBSR participants and 25 HEP participants for analyses. All analyses are conducted with participants' originally assigned interventions.

We also collected diary reports of both minutes and sessions of class-related home practice during the intervention (between T1 and T2) and from the end of the intervention to the four-month follow-up (between T2 and T3). By summing these data, we created variables indicating practice minutes and sessions from T1 to T2 and the same variables extending from T1 to T3.

Self-report measures were also collected at each time point (see [5] for details about the measures and primary self-report results). Primary self-report measures included the 90-item Symptom Checklist-90-R (SCL-90-R; [19]) consisting of nine subscales and three global scales. The Global Severity Index (GSI) provides a measure of overall psychological distress and has demonstrated sensitivity to change and adequate internal consistency [20]. The depression, anxiety, and hostility subscales also were used (Cronbach's alpha = .90, .85, and .84 respectively; test-retest reliabilities are *r* = .82, .80, and .78 respectively). The Medical Symptoms Checklist (MSC; [21]) measures the number of medical symptoms participants' experienced as problems in the last month. While the MSC has demonstrated sensitivity to change in past studies of MBSR [22], no further psychometric data is available.

## Results

### Discrimination (Target Height)

Target height (the length of the short line determined by the visual threshold procedure) served as an index of participants' ability to discriminate between target and non-target lines, with increases in target height indicating better visual discrimination (see [9]). To test the effects of Intervention, Time, and their interaction, univariate ANOVAs. We report repeated-measures ANOVAs as our primary analyses for these measures to facilitate comparison with MacLean et al (2010) [9] but report HLM-based results when they differ. Univariate ANOVAs were calculated using intervention as a between-participant variable and the Target Height for each time point (T1, T2, and T3) as a repeated, within-participant variable. There were significant linear main effects of time, *F*(1, 49) = 9.44, *p* = .003, *η* ^2^ = .16, indicating that Target Height increased at each time point (*M* = 96.93, 99.14, and 100.34 respectively). There were no other significant effects. Because target height was calibrated at each time point, any group differences in target height have the effect of making the task more difficult for the group with the greater target height. Thus, we performed simple comparisons in the form of univariate analyses for each time point, with Target Height as the dependent variable and Intervention as the between-participant variable. There were no group effects at any time point [T1: *F*(1, 56)<1; T2: *F*(1, 51) = 3.24, *p* = .08, *η^2^* = .06; T3: *F*(1, 49)<1], though the Time 2 trend is toward a higher Target Height for the MBSR relative to the HEP group (*M* = 101.35 and 97.25 respectively; see Table 4 for discrimination means by time and intervention). However, when Hierarchical Linear Models (HLM) were applied to test these effects, the estimated coefficient for the Intervention×Time (T1, T2, T3) effect was −1.796, *p* = .021 (with robust standard errors), implying that the MBSR group was able to identify targets at a reduced target height at T2 and T3 relative to HEP.

**Table 4:** Means by Intervention and Time for Discrimination (Target Height), Sensitivity (Average A′), and Vigilance (A′ slope over blocks).

	HEP			MBSR		
	T1	T2	T3	T1	T2	T3
Discrimination	95.83	97.25	99.61	98.04	101.35	101.07
Average Sensitivity	0.91	0.91	0.90	0.91	0.90	0.91
Vigilance	−0.021	−0.017	−0.024	−0.014	−0.009	−0.014

Finally, we found no significant correlations between Target Height change and either total minutes of practice or sessions of practice from T1 to T2 (all *r*'s<.09) or from T1 to T3 (the presence of two outliers in the HEP group was responsible for a significant correlation between Target Height change from T1 to T3 and Total practice minutes over the same time period, *r*(44) = .49, *p* = .04, which was seen in the HEP group, *r*(21) = .43, *p* = .05, rather than the MBSR group, *r*(23) = .17, n.s.).

### Average Sensitivity

Similar to Maclean and colleagues (2010), we tested for overall group differences in ability to discriminate between targets and non-targets by computing average sensitivity (A′) collapsing across blocks. We found no group differences in average A′ (across all 6 blocks) at any time point (all *F*'s<1), nor were their Intervention×Time interactions between T1 and T2 (all *F*'s<2.5) or between T1 and T3 (all *F*'s<1.3; see Table 4 for sensitivity by time and intervention). We also found no correlations between average A′ differences between T1 and T2 or between T1 and T3 and our measures of practice (the only significant correlation was between Average A′ from T1 to T3 and Total practice sessions and only for the MBSR group, *r*(23) = .43, *p* = .04, but was due to the presence of one outlier). We also found no significant Intervention×Time interactions for the relationship between Target Height and A′ either between T1 and T2 (*F*<1.2) or between T1 and T3 (*F*<1). Finally, there were no Intervention×Time interactions for the relationship between average A′ and our primary self-report measures between T1 and T2 (all *F*'s<1.3, except the Intervention×Time effect for the MSC, which was also non-significant but had a larger F-value, *F*(1,40) = 2.46, *p* = .13.) or between T1 and T3 (all *F*'s<1.3).

### Vigilance

**Figure 2:**
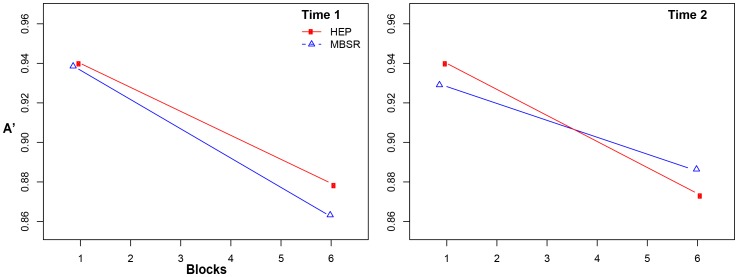
Graph based on an HLM model of A′ data for each block by time point for both MBSR and HEP participants. A′ is a nonparametric version of D′, an index of perceptual sensitivity [18] in which a score of 1 represents perfect discrimination between targets and distractors, .5 represents an inability to distinguish target from distractors, and a score below .5 indicates response confusion.

**Figure 3:**
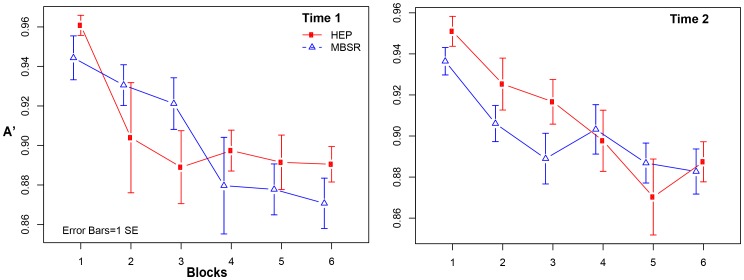
Graphs of A′ data (with 1 *SE* error bars) for each block by time point for both MBSR and HEP participants.

Using hierarchical linear models (HLMs), we modeled changes in vigilance as a function of the random effects of block (slope) and group, and their interaction, with random intercepts to allow for individual differences in initial performance (A′ during block 1) and random slopes. We used random slopes as opposed to fixed slopes so as to treat subjects as the units of analysis in testing for intervention effects, as subjects were the level at which randomization was applied. Though MacLean et al (2010) reported their results based on analyses with fixed slopes, their results were similar when random slopes were used. Furthermore, the results we report use threshold as a covariate. Both our results and MacLean's results were similar when threshold was used as a covariate. The estimated coefficient for the Intervention×Time (T1, T2)×Block effect was .0114, *p* = .006 (with robust standard errors), implying that the MBSR group showed .011 units improved vigilance (negative A′ slope) on the task at T2 compared to HEP, controlling for vigilance at T1 (see Figure 2). Although this effect implies confirmation of our hypothesis, further inspection revealed that the result is likely due to the fact that the MBSR group performed more poorly than the HEP group in early blocks at T2 relative to HEP (see Figure 3). Thus, the flatter slope (implying less fatigue) is not consistent with our hypothesis (see Table 4 for vigilance slopes by time and intervention). Because slope appeared inadequate as a model of sustained attention/fatigue, we attempted a number of alternative ways of modeling fatigue effects, including quadratic approaches, and became confident that the significant interaction was not consistent with our hypothesis that MBSR training improved sustained attention (or less fatigue) relative to HEP training. There was no significant Intervention×Time (T1, T3)×Block effect at T3 (results did not differ substantively with analyses involving various outlier strategies).

Hierarchical linear models used to investigate the relationship between changes in sustained attention and both self-report measures at T1 and changes over time in self-report measures revealed no significant effects. Hierarchical linear models were also used to investigate the effects of minutes and sessions of home practice on changes in sustained attention. Controlling for these respective practice effects in separate HLMs did not change the vigilance results reported above. There were no significant Intervention×Time interactions for change in slope and our primary self-report measures. Finally, there were no statistically significant effects of practice on A′ slope across sessions and no interaction with group.

## Discussion

In the present study, we investigated the effect of mindfulness training on sustained attention, hypothesizing that MBSR would improve sustained attention, as measured by a visual continuous performance task (CPT), compared to a novel active control group (HEP). Some of our results are consistent with our predictions and replicate the results from MacLean et al (2010) [9] and some do not.

Whereas MacLean and colleagues [9] found a significant group effect of discrimination (*η* ^2^ = .09; as measured by Target Height) in which the meditation retreat group showed increases in discrimination post-training relative to the wait-list group, the analogous comparison in our study (using the same statistical method of a repeated-measures ANOVA) revealed only a trend (*p* = .08, *η* ^2^ = .06) in the same direction, with MBSR participants tending to have a higher Target Height at T2 relative to HEP (*M* = 101.35 and 97.25 respectively). However, when HLM was used as the statistical method, the estimated coefficient for the Intervention×Time (T1, T2, T3) effect was significant, implying that the MBSR group was able to identify targets at 75% accuracy at a reduced target height at T2 and T3 relative to HEP. The *R^2^* for the effect from T1 to T2 was about .02, corresponding to a small-to-medium effect size with a Cohen's *d* of about .26. The *R^2^* for the effect from T1 to T3 was about .05, corresponding to a medium effect size with a Cohen's *d* of .53 (we converted our observed HLM effects into traditional effect size measures that reflected the difference between intervention groups divided by the pooled within-group standard deviation of effects, as quantified in our HLM models).

Our null results for average sensitivity (A′) and similar results for A′×time were also consistent with MacLean and colleagues [9], retreat 1. The latter effect had an *R^2^* of about .42, a large effect size corresponding to a Cohen's *d* of 1.73, assuming effect sizes are defined in relation to the residual standard deviation of A′ slopes within session. Our study was powered for this effect (power of .80 at alpha of .05 requires about 16 participants per group).

Like MacLean et al (2010) [9], we predicted improvements in vigilance as a result of mindfulness training. Unlike MacLean et al (2010) [9], retreat 1, who did not find group differences in vigilance (as measured by changes in A′ over blocks) pre-post intervention, we did find a significant intervention×time (T1, T2)×block interaction consistent with our prediction. However, this two-part hypothesis requires (1) equal A′ intercepts for both groups (or MBSR higher), and (2) a flatter A′ slope for MBSR across blocks relative to HEP; as the HLM model (Figure 2) and raw data (Figure 3) suggest, this significant result is consistent with the latter but not the former. Thus, this result is not interpretable.

Finally, our predictions that improvements in attention would be moderated by class practice were also not confirmed. Likewise, CPT indices were also not related to changes in self-report measures over time by intervention.

In summary, the following results replicate those found by MacLean et al (2010) [9] for their first retreat: a null result for sensitivity, and a significant result for Discrimination. For vigilance, MacLean et al (2010) [9] found no Intervention×Time interaction whereas we found a significant Intervention×Time effect for vigilance that is not interpretable.

Because our study ran concurrently with MacLean et al.'s first retreat, we were unable to change our procedure as they did for their second cohort (retreat 2). Specifically, in their second cohort, instead of calibrating Target Height for each participant at each time point, MacLean et al used the Target Height determined at the beginning of retreat 2 for all subsequent time points. With this change in procedure for their second cohort, MacLean et al found that meditation retreat participants showed increases in visual discrimination, overall sensitivity (A′ collapsed across blocks) and vigilance over time compared to these same participants when they were wait-list controls. Given that the null vigilance results in the present study are similar to those in MacLean et al (for retreat 1), and that MacLean et al found improvements in vigilance in a second cohort, it seems promising to replicate the procedures used in MacLean et al's retreat 2 in a future study. Only then would be able to investigate primary questions of interest: namely, the effect of using an active control condition (in addition to a wait-list control condition) and the effect of training dose (8 weeks versus 3 months).

In the meantime, our results suggest four other possible conclusions. First, it is possible that MBSR training does not improve sustained attention per se, but exerts effects on attention through indirect mechanisms, such as compassion for self and others; attitudes such as patience and curiosity; insight into various aspects of interpersonal relationships, mental life, emotions, and behavior; and equanimity.

Second, it is possible that MBSR training does not improve sustained attention, but affects other aspects of attention. Indeed, changes in attention have been found as a result of MBSR, including in the orienting sub-component of voluntary-dorsal attention networks [23], in interoceptive attention [24], selective attention, perception, and visual working memory [25]. In other words, if we had used one more of these other tasks, we might have found significant attention effects. Brief training over time has also been associated with increased ability to focus attention as measured by EEG [26]. However, only the study by Jensen and colleagues involved a non-waitlist control.

Third, it is possible that 8 weeks of MBSR training does not provide a sufficient dose to achieve measureable changes in sustained attention specifically. The findings on MBSR-induced effects on sustained attention are not currently conclusive. Whereas [27] reported an effect of MBSR on vigilance, the two studies quoted above, found rather an effect of MBSR on selective attention [23], [25]. These mixed finding could be an indication of the small effect size of this effect. Indeed, more intensive mindfulness training has been associated with larger attention effects. Brown and colleagues [28] found increased perceptual discrimination in participants of a 3-month mindfulness retreat compared to a retreat staff control group. Slagter and colleagues [29] found that participants of a 3-month retreat had improved attentional performance on a rapid serial visualization task than demographically matched, naïve controls with minimum meditation training. Of most relevance, Lutz and colleagues found that a three month retreat reduced trial-to-trial variability in reaction time and brain response during a sustained attention task compared to a wait-list control [8]. Of course, MacLean and colleagues (2010) [9] also found their effects in a 3-month retreat.

Fourth, MBSR is a secular form of intervention whereas other research, including MacLean et al, investigates more traditional Buddhist training. For future research it is important to note that the mode (secular vs. non-secular) and context of training (residential retreat vs. community-based groups) is often confounded with length of training (weeks vs. months). This presents a challenge for research interested in identifying the active ingredients and effective dose of meditation training. In the present case, it is possible that each of these factors account for our null findings relative to the findings of MacLean et al.

Whatever conclusion future research proves most compelling, our findings are likely to be generalizable to a typical MBSR population given our focus on recruiting such a population and the typical nature of the MBSR intervention itself. The only limitations to this generalizability are due to eligibility criteria for the study itself (Table 1).

In conclusion, our results are largely consistent with those reported by MacLean and colleagues [9], retreat 1, in which CPT procedures were nearly identical to ours. This fact, and the significant attention effects found by MacLean and colleagues in retreat 2, suggest that relatively minor procedural changes are important to include in future studies. When such changes are incorporated, future studies will be able to test attention effects related to intensity/duration of training (community groups for 8 weeks vs. residential retreat for 3 months) and type of control group (active control group vs. wait-list), two variables that may account for the smaller effects found in the current study relative to MacLean et al.'s study.

## Supporting Information

Checklist S1 **CONSORT checklist.**
(DOC)

Protocol S1 **Trial Protocol.**
(DOC)
