# Supplementary material for: No Sustained Attention Differences in a Longitudinal Randomized Trial Comparing Mindfulness Based Stress Reduction versus Active Control
Source: PLoS One. 2014 Jun 23;9(6):e97551. doi: 10.1371/journal.pone.0097551 (PMC4067292; doi:10.1371/journal.pone.0097551)
Supplement: Protocol S1 — Trial Protocol. (DOC) [file pone.0097551.s002.doc]

Health Enhancement Program (HEP) – Mindfulness Based Stress Reduction (MBSR) Study

OPERATIONS MANUAL

Principal Investigator: Antoine Lutz, Ph.D.

Co-investigators: Donal MacCoon, Ph.D., Melissa Rosenkranz, M.S., Helen Weng

Co-investigator/Senior Scientist: Richard Davidson, Ph.D.

Consultant: Timothy Salomons, M.S.

NIH Program Officer & Project Scientist: Catherine M. Stoney, Ph.D.;

NIH Project Scientist: Laura Lee Johnson, Ph. D.
Study Coordinator/Research Assistant: Jenna Sheftel

Waisman Center

UW Department of Psychology

1500 Highland Ave

Madison, WI 53705

Table of Contents

Introduction

How to Use the Operations Manual…………………………………………………..

Chapter 1

Overview of Study……………………………………………………………………

Key Personnel…………………………………………………………………

Clinic Visit Structure………………………………………………………………

Overall Study Timeline........……………………………………………………

Cohort Timelines……………………………………………………………...

Physiological Measures Timeline………………………………………………

HEP and MBSR: Comparison of components…………………………………

Advertising Locations…………………………………………………………

Phone Interview……………………………………………………………….

Global Meeting……………………………………………………….....

Pre-training Information Sessions…………………………………………….

Randomization…………………………………………………………………

Pre-training Data Collection…………………………………………………...

Teacher Qualifications…………………………..………………………………...

Training Courses………………………………………………………………

Post-training Data Collection………………………………………………….

Follow-up Data Collection……………………………………………………..

Chapter 2

Description of Study Procedures……………………………………………………….

Teacher Critieria………………………………………………………………

Advertisement………………………………………………………………….

Criteria of Exclusion & Inclusion……………………………………………...

Information Session Content…………………………………………………..

Randomization Procedures……………………………………………………..

Organization of Data Files……………………………………………………….

Payment of Participants…………………………………………………………

Chapter 3

Runlogs…………………………………………………………………………………

Voice Mail Protocol……………………………………………………………

E-Mail Protocol………………………………………………………………..

Telephone Screening Instructions……………………………………………...

Phone Script……………………………………………………………………

Reminder and Scheduling Call Runlogs………………………………………..

How to Make a Cortisol Kit……………………………………………………

Pre All Sessions Runlog……………………………………………………….

Post All Sessions Runlog………………………………………………………

Mock fMRI Scan Runlog……………………………………………………….

Attention Task Runlog………………………………………………………….

Attention Task Log Sheet……………………………………………………….

TSST Runlog……………………………………………………………………

TSST Committee Member Instructions – Visit #1……………………………

TSST Committee Member Instructions – Visit #2……………………………

TSST Committee Member Instructions – Visit #3……………………………

Heat Stimulation & Emotion Regulation Tasks Runlog………………………

Chapter 4

Handout Timeline……………………………………………………………………..

Participant Handouts Timeline………………………………………………..

Teacher Handouts Timeline…………………………………………………..

Appendix

Additional Forms/Information

Recruitment E-Mail……………………………………………………………

Information Packet…………………………………………………………….

Class Assignment Notification, HEP-cohort1…………………………………

Class Assignment Notification, HEP-cohort2…………………………………

Class Assignment Notification, MBSR-cohort1……………………………….

Class Assignment Notification, MBSR-cohort2………………………………

Maps & Directions…………………………………………………………….

Consent Form………………………………………………………………….

Medical & Physical Activity History Form …………………………………..

Registration Form……………………………………………………………...

MRI Screening Form………………………………………………………….

MRI Screening Form Key……………………………………………………..

MRI Doctors Note Packet …………………………………………………….

Saliva Sampling Instructions & Packets……………………………………..

Post Class Teacher Questionnaire……………………………………………..

4-month Practice Log (HEP, cohort #1) ………………………………………

4-month Practice Log (HEP, cohort #2) ………………………………………

4-month Practice Log (MBSR, cohort #1) ……………………………………

4-month Practice Log (MBSR, cohort #2) ……………………………………

Home Assignments Log (HEP) ……………………………………………….

Home Assignments Log (MBSR) …………………………………………….

Make-Up Class Documentation (HEP) ……………………………………….

Make-Up Class Documentation (MBSR) ……………………………………..

Adverse Reactions Protocol…………………………………………………..

Emergency Phone Numbers………………………………………………….

**Introduction**

How to Use the Operations Manual

**How to Use the Operations Manual**

The main functions of this manual are to provide, one, an overview of the HEP-MBSR Study and, two, detailed instructions for the carrying out of any and all study procedures or processes.

Chapter 1 includes a summary of the main elements of the study. The remainder of the manual contains highly specified and detailed information. In Chapters 2 there are in depth descriptions of specific study procedures. Chapter 3 contains the runlogs and instructions used during any researcher contact with study participants, such as data collection sessions. Chapter 4 details the appropriate time for all handouts to be given to participants or course teachers. All additional forms can be found in the Appendix.

**Chapter 1**

Overview of Study

**Overview of Study**

Key Personnel

| **Name & Credentials** | **Job Title & Role** | **Contact Information** |
| --- | --- | --- |
| Richard Davidson, Ph.D | - Professor  - Co-investigator | W: (608) 262-8972 |
| Antoine Lutz, Ph. D. | - Research Scientist  - Primary Investigator | W: (608) 262-8705  C: (608) 335-9845  alutz@wisc.edu |
| Donal MacCoon Ph.D. | - Post-Doc  - Co-investigator | W: (608) 263-1968  H: (608) 240-9950  dgmaccoon@wisc.edu |
| Melissa Rosenkranz, BA | - Graduate Student  - TSST task | W: (608) 262-5050  H: (608) 250-3086 |
| Helen Weng, BA | - Graduate Student  - fMRI task with IAPS pictures | W: (608) 263-0269  C: (917) 543-7292  hweng1@gmail.com |
| Tim Salomons | - Graduate Student  - fMRI pain task consultant | 263-1968 [tvsalomons@wisc.edu](mailto:tvsalomons@wisc.edu) |
| Daniel Levinson, BA | - Research Assistant  - fMRI data analysis | danlevinson@gmail.com |
| Jenna Sheftel, BA | - Research Assistant  - Study Coordinator | C: (847) 602-0482  MBSRstudy@gmail.com |
| Six (+) research assistants | - Undergraduate Research Assistants from the  Department of Psychology  - Research, supervised by investigators |  |
| Diana Elizabeth Grove, BA, BS | - Instructor, Mindfulness Based Stress Reduction  Center  - Research consultant of MBSR class | (608) 233-1640; dg1946@charter.net |
| Cindy McCallum, CSW | - Instructor, Mindfulness Based Stress Reduction  Center  - MBSR Instructor & Research consultant | (608) 220-4015; camccallum@sbcglobal.net |
| Katherine Bonus, M.A | - Manager, Mindfulness Based Stress Reduction Center  - Teacher of MBSR class and Research Consultant | (608) 265-8417 |
| Jude Sullivan, M.S. | - Senior Clinical Exercise Physiologist UW Health  Integrative Medicine  - Chief instructor for Health Enhancement  Program | jc.sullivan@hosp.wisc.edu |
| Aaron Field, M.D. Ph.D. | - Radiologist at the University Hospital  - Supervisor of the reading of the fMRI scans |  |
| James Davis, M.D. | - Physician collaborator in the Lab for Affective Neuroscience  - Will be present for the first 5 blistering sessions and on call for the  rest |  |
| Pat Roseboom, Ph.D. | - Associate Scientist  - Cortisol Assay Supervisor | (608) 263-0504 |
| Julie Thurlow | - Nutritionist |  |
| Pamela Christmas-Polcyn | - Music Therapist |  |
| Lael Sheber | Clinical Nurse Specialist  Nurse responsible for blood draws |  |
| Hannah Copp | Clinical Nurse Specialist  Nurse responsible for blood draws |  |

Clinic Visit Structure

|  | Task | Time (mins) |
| --- | --- | --- |
| Visit 1, 4, 7 | Initial paperwork (visit 1 only) | 15 |
|  | Mock scan (visit 1 only) | 15 |
|  |  |  |
|  | Cortisol kit instructions | 15 |
|  | Computer task | 45 |
|  | Questionnaires | 20 |
|  | Subtotal (hours): | 2 |
|  |  |  |
| Visit 2, 5, 8 | Introduction and baseline cortisol | 20 |
|  | TSST/capsaicin challenge (mean) | 60 |
|  | Cortisol samples/questionnaires | 30 |
|  | Subtotal (mean hours): | 1.83 |
|  |  |  |
| Visit 3, 6, 9 | Scans for both studies | 90 |
|  | Questionnaires | 15 |
|  | Subtotal (hours): | 1.75 |
|  |  |  |
|  | TOTAL per phase (hours): | 5.58 |
|  |  |  |
| Before Class #1 | Blood Draw | 15 |
| After Class #8 | Blood Draw | 15 |
| Visit 10 | Blood Draw | 15 |
|  |  |  |
|  | TOTAL for all 3 phases (hours): | 17.50 |

Overall Study Timeline

| Overall Study Timeline | | |
| --- | --- | --- |
| **Day** | **Date** | **Description** |
| Monday | 7/10/06 | First Information Session |
| Tuesday | 7/11/06 | Second Information Session |
| Wednesday | 7/26/06 | Third Information Session |
| Thursday | 7/27/06 | Fourth Information Session |
| Wednesday | 8/2/2006 | MBSR1 -Pre-training data collection begins (4 wks) |
| Thursday | 8/3/2006 | HEP1 -Pre-training data collection begins (4 wks) |
| Monday | 8/28/2006 | HEP2 -Pre-training data collection begins (4 wks) |
| Tuesday | 8/29/2006 | MBSR1 -End of pre-training data collection |
|  |  | MBSR2 -Pre-training data collection begins (4 wks) |
| Wednesday | 8/30/2006 | HEP1 -End of pre-training data collection |
|  |  | MBSR1 -Class #1- First day of training (8 wks; 6-8:30 p.m.) |
| Thursday | 8/31/2006 | HEP1 -Class #1- First day of training (8 wks; 6-8:30 p.m.) |
| Wednesday | 9/6/2006 | MBSR1 -Class #2 |
| Thursday | 9/7/2006 | HEP1 -Class #2 |
| Wednesday | 9/13/2006 | MBSR1 -Class #3 |
| Thursday | 9/14/2006 | HEP1 -Class #3 |
| Wednesday | 9/20/2006 | MBSR1 -Class #4 |
| Thursday | 9/21/2006 | HEP1 -Class #4 |
| Sunday | 9/24/2006 | HEP2 -End of pre-training data collection |
| Monday | 9/25/2006 | HEP2 -Class #1- First day of training (8 wks; 6-8:30 p.m.) |
|  |  | MBSR2 -End of pre-training data collection |
| Tuesday | 9/26/2006 | MBSR2 -Class #1- First day of training (8 wks; 6-8:30 p.m.) |
| Wednesday | 9/27/2006 | MBSR1 -Class #5 |
| Thursday | 9/28/2006 | HEP1 -Class #5 |
| Monday | 10/2/2006 | HEP2 -Class #2 |
| Tuesday | 10/3/2006 | MBSR2 -Class #2 |
| Wednesday | 10/4/2006 | MBSR1 -Class #6 |
| Thursday | 10/5/2006 | HEP1 -Class #6 |
| Saturday | 10/7/2006 | HEP1 -Day of Training (9am-4pm) |
|  |  | MBSR1 -Day of Mindfulness (9am-4pm) |
| Monday | 10/9/2006 | HEP2 -Class #3 |
| Tuesday | 10/10/2006 | MBSR2 -Class #3 |
| Wednesday | 10/11/2006 | MBSR1 -Class #7 |
| Thursday | 10/12/2006 | HEP1 -Class #7 |
| Monday | 10/16/2006 | HEP2 -Class #4 |
| Tuesday | 10/17/2006 | MBSR2 -Class #4 |
| Wednesday | 10/18/2006 | MBSR1 -Class #8 - Last day of training |
| Thursday | 10/19/2006 | HEP1 -Class #8 - Last day of training |
|  |  | MBSR1 -Post-training data collection begins (4 wks) |
| Friday | 10/20/2006 | HEP1 -Post-training data collection begins (4 wks) |
| Monday | 10/23/2006 | HEP2 -Class #5 |
| Tuesday | 10/24/2006 | MBSR2 -Class #5 |
| Monday | 10/30/2006 | HEP2 -Class #6 |
| Tuesday | 10/31/2006 | MBSR2 -Class #6 |
| Saturday | 11/4/2006 | HEP2 -Day of Training (9am-4pm) |
|  |  | MBSR2 -Day of Mindfulness (9am-4pm) |
| Monday | 11/6/2006 | HEP2 -Class #7 |
| Tuesday | 11/7/2006 | MBSR2 -Class #7 |
| Monday | 11/13/2006 | HEP2 -Class #8 - Last day of training |
| Tuesday | 11/14/2006 | HEP2 -Post-training data collection begins (4 wks) |
|  |  | MBSR2 -Class #8 - Last day of training |
| Wednesday | 11/15/2006 | MBSR1 -End of Post-training data collection |
|  |  | MBSR2 -Post-training data collection begins (4 wks) |
| Thursday | 11/16/2006 | HEP1 -End of Post-training data collection |
| Monday | 12/11/2006 | HEP2 -End of Post-training data collection |
| Tuesday | 12/12/2006 | MBSR2 -End of Post-training data collection |
| Thursday | 2/15/2007 | 17 weeks post-training data collection begins (4 wks) |
| Friday | 2/16/2007 | HEP1 -17 weeks post-training data collection begins (4 wks) |
| Tuesday | 3/13/2007 | HEP2 -17 weeks post-training data collection begins (4 wks) |
| Wednesday | 3/14/2007 | MBSR1 -End of 17 weeks post-training data collection |
|  |  | MBSR2 -17 weeks post-training data collection begins (4 wks) |
| Thursday | 3/15/2007 | HEP1 -End of 17 weeks post-training data collection |
| Monday | 4/9/2007 | HEP2 -End of 17 weeks post-training data collection |
| Tuesday | 4/10/2007 | MBSR2 -End of 17 weeks post-training data collection |

Cohort Timelines

| Timeline: MBSR-1 | | |
| --- | --- | --- |
| **Day** | **Date** | **Description** |
|  | 7/10/06 | First Information Session |
|  | 7/11/06 | Second Information Session |
| Wednesday | 8/2/2006 | Pre-training data collection begins (4 wks) |
| Tuesday | 8/29/2006 | End of pre-training data collection |
| Wednesday | 8/30/2006 | Class #1- First day of training (8 wks; 6-8:30 p.m.) |
| Wednesday | 9/6/2006 | Class #2 |
| Wednesday | 9/13/2006 | Class #3 |
| Wednesday | 9/20/2006 | Class #4 |
| Wednesday | 9/27/2006 | Class #5 |
| Wednesday | 10/4/2006 | Class #6 |
| Saturday | 10/7/2006 | Day of Mindfulness (9am-4pm) |
| Wednesday | 10/11/2006 | Class #7 |
| Wednesday | 10/18/2006 | Class #8 - Last day of training |
| Thursday | 10/19/2006 | Post-training data collection begins (4 wks) |
| Wednesday | 11/15/2006 | End of Post-training data collection |
| Thursday | 2/15/2007 | 17 weeks post-training data collection begins (4 wks) |
| Wednesday | 3/14/2007 | End of 17 weeks post-training data collection |

| Timeline: HEP-1 | | |
| --- | --- | --- |
| **Day** | **Date** | **Description** |
|  | 7/10/06 | First Information Session |
|  | 7/11/06 | Second Information Session |
| Thursday | 8/3/2006 | Pre-training data collection begins (4 wks) |
| Wednesday | 8/30/2006 | End of pre-training data collection |
| Thursday | 8/31/2006 | Class #1- First day of training (8 wks; 6-8:30 p.m.) |
| Thursday | 9/7/2006 | Class #2 |
| Thursday | 9/14/2006 | Class #3 |
| Thursday | 9/21/2006 | Class #4 |
| Thursday | 9/28/2006 | Class #5 |
| Thursday | 10/5/2006 | Class #6 |
| Saturday | 10/7/2006 | Day of Training (9am-4pm) |
| Thursday | 10/12/2006 | Class #7 |
| Thursday | 10/19/2006 | Class #8 - Last day of training |
| Friday | 10/20/2006 | Post-training data collection begins (4 wks) |
| Thursday | 11/16/2006 | End of Post-training data collection |
| Friday | 2/16/2007 | 17 weeks post-training data collection begins (4 wks) |
| Thursday | 3/15/2007 | End of 17 weeks post-training data collection |

| Timeline: HEP-2 | | |
| --- | --- | --- |
| **Day** | **Date** | **Description** |
|  | 7/26/06 | Third Information Session |
|  | 7/27/06 | Fourth Information Session |
| Monday | 8/28/2006 | Pre-training data collection begins (4 wks) |
| Sunday | 9/24/2006 | End of pre-training data collection |
| Monday | 9/25/2006 | Class #1- First day of training (8 wks; 6-8:30 p.m.) |
| Monday | 10/2/2006 | Class #2 |
| Monday | 10/9/2006 | Class #3 |
| Monday | 10/16/2006 | Class #4 |
| Monday | 10/23/2006 | Class #5 |
| Monday | 10/30/2006 | Class #6 |
| Saturday | 11/4/2006 | Day of Training (9am-4pm) |
| Monday | 11/6/2006 | Class #7 |
| Monday | 11/13/2006 | Class #8 - Last day of training |
| Tuesday | 11/14/2006 | Post-training data collection begins (4 wks) |
| Monday | 12/11/2006 | End of Post-training data collection |
| Tuesday | 3/13/2007 | 17 weeks post-training data collection begins (4 wks) |
| Monday | 4/9/2007 | End of 17 weeks post-training data collection |

| Timeline: MBSR-2 | | |
| --- | --- | --- |
| **Day** | **Date** | **Description** |
|  | 7/26/06 | Third Information Session |
|  | 7/27/06 | Fourth Information Session |
| Tuesday | 8/29/2006 | Pre-training data collection begins (4 wks) |
| Monday | 9/25/2006 | End of pre-training data collection |
| Tuesday | 9/26/2006 | Class #1- First day of training (8 wks; 6-8:30 p.m.) |
| Tuesday | 10/3/2006 | Class #2 |
| Tuesday | 10/10/2006 | Class #3 |
| Tuesday | 10/17/2006 | Class #4 |
| Tuesday | 10/24/2006 | Class #5 |
| Tuesday | 10/31/2006 | Class #6 |
| Saturday | 11/4/2006 | Day of Mindfulness (9am-4pm) |
| Tuesday | 11/7/2006 | Class #7 |
| Tuesday | 11/14/2006 | Class #8 - Last day of training |
| Wednesday | 11/15/2006 | Post-training data collection begins (4 wks) |
| Tuesday | 12/12/2006 | End of Post-training data collection |
| Wednesday | 3/14/2007 | 17 weeks post-training data collection begins (4 wks) |
| Tuesday | 4/10/2007 | End of 17 weeks post-training data collection |

Physiological Measures Timeline

| **Physiological measure** | **Pre-Class** | **First Class** | **Last Class** | **Post-Class** | **Follow-Up** |
| --- | --- | --- | --- | --- | --- |
| Salivary Cortisol | ⯌ |  |  | ⯌ | ⯌ |
| Blood Sample |  | ⯌ | ⯌ |  | ⯌ |
| fMRI | ⯌ |  |  | ⯌ | ⯌ |
| Immune Response | ⯌ |  |  | ⯌ | ⯌ |

HEP and MBSR: Comparison of components

Qualified experts in a relevant field conduct both interventions and instructors believe in the efficacy of the treatments being offered.

The two interventions are structurally equivalent:

- Same class schedule (1 class per week for 8 weeks)
- Same amount of time per class
- All-day class exists for both
- Classes are taught by competent instructors
- Weekly peer supervision meetings will be held for both groups
- Class formats are similar (see table below)
- Class ingredients are similar (see table below)
- Equivalent type and quantity of homework will be assigned

| MBSR Components | HEP Components |
| --- | --- |
| Exercises used in every course: |  |
| Body Scan Meditation | Music Therapy: Guided Imagery |
| Sitting meditation | Music Therapy: Music-making, guided imagery, song-writing; Nutrition (didactics) |
| Yoga | Functional Movement |
| Walking meditation | Physical Activity |
| Eating meditation | Music Therapy: Music-making, guided imagery, song-writing; Nutrition (didactics) |
|  |  |
| Every class will contain: |  |
| 30-60 min guided practice | Same |
| Large group, dyad or triad dialog depending on situation | Same |
| Question and answer with teacher | Same |
|  |  |
| Homework: |  |
| Body Scan Meditation | Music therapy: guided imagery |
| Sitting meditation | Nutrition (sitting in front of computer); Music making |
| Yoga | Functional movement homework |
| Walking meditation | Physical activity homework |

Advertising

The following is a list of places where posters for the study will be placed

State Street (& near by)

**International Friendship Center (Closed until after Memorial Day)**

701 State Street

608 255-7214

*Director must approve flyers prior to posting. Call to arrange.

**Catacombs Coffee House**

731 State St
608 257-3025

**Starbucks**

661 State St

608 294-8345

**Espresso Royale**

650 State St
608 259-0300

**Steep & Brew**

544 State St
608 256-2902

**Rainbow Book Store**

426 W Gilman St, Madison, WI

608 257-6050

**Fair Trade Coffee House**

418 State St
 608 268-0477

**A Room of Ones Own**

307 W Johnson St, Madison, WI

608 257-7888

**Espresso Royal Café**

208 State St, Madison, WI

608 259-0800

**Michelangelo's Coffee House**

114 State St
 608 251-5299

Minority Recruitment

**Asian Midway Foods**

301 S. Park

**Centro Hispano**

810 W. Badger Rd

608 255-3018

**Copps Food Centers**

1312 S. Park (7am-11pm)

**Copps Food Centers**

2502 Shopko Dr (open 24 hrs)

**Cub Foods**

4716 Verona Rd

**Dane County Department of Human Services**

1202 Northport Dr.
Madison, WI 53704

**Eagle Heights Community Center**

611 Eagle Heights

**Madison Public Library**

Central Branch

201 W. Mifflin St.

**United Refugee Services of Wisconsin Inc**

312 North 3^rd^ Ave

256-6400

**Urban League of Greater Madison**

151 E. Gorham St

608 251-8550

**Woodmans**

3817 Milwaukee St

**Multicultural Student Center**

2nd Floor 'The Old Red Gym'
716 Langdon Street

Wellness Centers

**Synergy Wellness Center**

3310 University Ave
2nd Floor Danford Plaza
608 233-3588

**Whole Foods**

3313 University Avenue
608 233-9566

**Mifflin St Community Co-op**

32 N Bassett St, Madison, WI

608 251-5899

**Willy St Co-op**

1221 Williamson St.
608251-0884

**Electric Earth**

546 W Washington Ave

608 255-2310

**Body Works Wellness Center**

6417 Odana Rd

608 663-4905

**Integrative Medicine Center**

Research Park Clinic
621 Science Dr.
608 265-8303

Phone interview

Each interested person will be contacted by phone (*For runlog, refer to Chapter 3*)

Global Meeting

On Wednesday June 7^th^ from 6:30-8:30pm, HEP-MBSR study experimenters and coordinators will all gather at the Integrative Medicine Center to meet with the instructors for both courses. This meeting will be used to:

1. Review the operations manual
2. Go over the importance of the participants remaining naïve to the purpose of the study
3. Give the center an advertisement for the study to place in the waiting room

Pre-training Information Sessions

- Information sessions will be held on the July 10, 11, 26, and 27, 6:30-8:30 p.m.
- Each participant is required to attend one information session.
- There will be a half hour presentation about both programs. For these presentations the group of potential participants at the session will be split into two smaller groups and move to separate rooms. One group will hear about HEP for the first ½ hour while the other group hears about MBSR. Then the teachers will switch rooms and the groups will hear about the other class. The smaller groups will allow for a more intimate setting and for the teachers to get a better sense of the potential participants.
- Goals
  - Inform participants of research & answer questions
  - Inform participants of HEP and MBSR & discern who is a good fit
  - Give out initial research materials
  - Schedule first 3 clinical visits
  - Give out pre-class materials
  - Instructors red flag potential participants who do not have a 18.5 body mass index (BMI) or higher
  - Pre-paid envelopes will be provided so that participants can mail in their consent forms at a later point
- Wait list
  - A wait list will be created as soon as all of the class spots are full
  - This wait list will be used to fill seats that are emptied due to drop out
  - After Cohort #2 begins their class drop out spaces can no longer be filled

** *When a person is scheduled, this must be noted in the room calendar and the Master Logistics Spreadsheet (Y:/MBSR/Logistics/Master Logistics Spreadsheet). All scheduling (initial and changes) and folder preparations are done by Jenna’s Team

Please refer to Chapter 2 for more detailed information about the pre-training information sessions.

Randomization

- The study statistician will randomize participants to classes after information sessions have been completed and all decisions about exclusion have been made. Component heads will be blind to this information.
- Contact participants with course information via mail or e-mail

Pre-training Data Collection

- All data for participants within a single cohort will be collected in a 4-week time frame.
- The pre-training data collection is split up into three, approximately 2 hour, visits to the Waisman Center per participant (It is not necessary for the visits to occur in any specific order. The numbers are used as identifying marks)
- Completed cortisol kit #1 can be returned at any of the three clinic visits and must be returned within the 4-week data collection period.
- Lab assistant running participants will check WiscCal for scheduled participants, pick up their component file from the master file, complete required tasks, and return completed file to master file. Lab Assistant is responsible for completing the Master Logistics Spreadsheet (MLS).
- Visit content
  - Visit #1 (Donal-Antoine-Helen Team) – create run-log with all details below.
    - Collection of Physician Authorization and Medical/Physical History forms
      - If Physician Authorization form is not turned in, participant must be reminded that if it isn’t turned in at visit 2, visit 2 will need to be rescheduled.
    - Attention Task (*For runlog, refer to Chapter 3*)
    - Mock fMRI scan (*For runlog, refer to Chapter 3*)
    - Cortisol kit instructions refresher
      - Saliva Sampling Instructions (*For a copy, refer to the Appendix*)
    - Scheduling
      - Reminder of upcoming clinic visit date/time
      - Reminder to bring in forms and cortisol kit

When 2 participants are run at one time, one participant will begin with the Mock Scan & Cortisol Instructions and the other will begin with the Behavioral Task.

- - Visit #2 (Melissa Team)
    - Questionnaires & forms (*For list, refer to chapter 4*)
    - Collection of Physician Authorization and Medical/Physical History forms
      - If participant does not have their completed Physician Authorization form, session cannot be run and must be rescheduled
      - E-mail Jenna (MBSR@gmail.com) to inform her that session must be rescheduled
    - TSST Task (*For runlog, refer to Chapter 3*)
      - Twenty-minute rest period
      - Two, five-minute “challenges”
      - Blistering procedure with questionnaires & forms (*For list, refer to chapter 4*)
        - Dr James Davis, M.D. will be present for the first 5 blistering procedures and on call for all subsequent blistering procedures
      - Six salivary cortisol samples collected (throughout the length of the visit)
    - Scheduling
      - Reminder of upcoming clinic visit date/time
  - Visit #3 (Antoine-Helen Team)
    - Female participants only
      - Warned about possibility of risk due to an fMRI scan during pregnancy.
      - Asked if she is pregnant or believes there may be a chance of pregnancy
      - A pregnancy test is offered
      - If female participant will not sign the portion of consent form that asks about pregnancy and will also not take a pregnancy test, she will not be included in the fMRI portion of the study
      - Pregnant participants will not participate in the fMRI scan
    - fMRI Scans (*For runlog, refer to Chapter 3*)
      - Prepare participant for scan
      - Heat Stimulation Regulation Task
      - Emotion Regulation Task
    - Scheduling
      - Ask participant whether they would like to be contacted by e-mail or phone for course assignment information
      - Forms for payment #1 completed & placed in Donna Cole’s mail box on the second floor
- Class Assignment Notification
  - As soon as participants have finished visit #3 they will be contacted via mail and phone to inform them of their class assignment.
  - Drop-out rates early on will inform decisions about needed recruitment numbers.

Teacher Qualifications

- See *MBSR Providers: Qualifications and Recommended Guidelines*
- See *HEP Providers: Qualifications and Recommended Guidelines*

Training Courses

- Health Enhancement Program
  - Blood Draw #1 occurs on first day of class (*For runlog, refer to Chapter 3*)
  - Questionnaires (*Listed in Chapter 4)*
  - Blood Draw #2 occurs on the last day of class (*For runlog, refer to Chapter 3*)
  - Hand out cortisol kit #2 and schedule visits 4-6 at class #8
- Mindfulness Based Stress Reduction Program
  - Blood Draw #1 occurs on first day of class (*For runlog, refer to Chapter 3*)
  - Questionnaires (*Listed in Chapter 4)*
  - Blood Draw #2 occurs on the last day of class (*For runlog, refer to Chapter 3*)
  - Hand out cortisol kit #2 and schedule visits 4-6 at class #8

Post-Training Data Collection

- All data for participants within a single cohort will be collected in a 4 week time frame.
- The post-training data collection is split up into four visits to the Waisman Center per participant. The first three lasting approximately 2 hours and the fourth lasting 10-15 minutes (It is not necessary for the visits to occur in any specific order. The numbers are used as identifying marks).
- Completed cortisol kit #1 can be returned at any of the three clinic visits and must be returned within the 4-week data collection period.
- Visit content
  - Visit #1 (Donal Team)
    - Attention Task (*For runlog, refer to Chapter 3*)
    - Cortisol kit instructions refresher
      - Saliva Sampling Instructions (*For a copy, refer to the Appendix*)
    - Scheduling
      - Reminder of upcoming clinic visit date/time
  - Visit #2 (Melissa Team)
    - Questionnaires & forms (*For list, refer to chapter 4*)
    - TSST Task (*For runlog, refer to Chapter 3*)
      - Twenty-minute rest period
      - Two, five-minute “challenges”
      - Blistering procedure
      - Six salivary cortisol samples collected (throughout the length of the visit)
    - Scheduling
      - Reminder of upcoming clinic visit date/time
  - Visit #3 (Antoine-Helen Team)
    - Female participants only
      - Warned about possibility of risk due to an fMRI scan during pregnancy.
      - Asked if she is pregnant or believes there may be a chance of pregnancy
      - A pregnancy test is offered
      - If female participant will not sign the portion of consent form that asks about pregnancy and will also not take a pregnancy test, she will not be included in the fMRI portion of the study
      - Pregnant participants will not participate in the fMRI scan
    - fMRI Scans (*For runlog, refer to Chapter 3*)
      - Prepare participant for scan
      - Heat Stimulation Regulation Task
      - Emotion Regulation Task
    - Scheduling
      - Reminder of upcoming clinic visit date/time
      - Forms for payment # 2 completed & placed in Donna Cole’s mail box on the second floor

Follow-up Data Collection

- All data for participants within a single cohort will be collected in a 4 week time frame.
- The follow-up data collection is split up into one half hour visit and three, approximately 2 hour, visits to the Waisman Center per participant (It is not necessary for the visits to occur in any specific order. The numbers are used as identifying marks)
- Completed cortisol kit #2 can be returned at any of the three clinic visits and must be returned within the 4-week data collection period.
- Visit content
  - Visit #1 (Donal-Antoine-Helen Team)
    - Attention Task (*For runlog, refer to Chapter 3*)
    - Mock fMRI scan (*For runlog, refer to Chapter 3*)
    - Participant receives cortisol kit #3
      - Saliva Sampling Instructions (*For a copy, refer to the Appendix*)
    - Scheduling
      - Reminder of upcoming clinic visit date/time

When 2 participants are run at one time, one participant will begin with the Mock Scan & Cortisol Instructions and the other will begin with the Behavioral Task. After 1.5 hours the participants will switch.

- - Visit #2 (Melissa Team)
    - TSST Task (*For runlog, refer to Chapter 3*)
      - Twenty-minute rest period
      - Two, five-minute “challenges”
      - Blistering procedure
      - Six salivary cortisol samples collected (throughout the length of the visit)
    - Questionnaires & forms (*For list, refer to chapter 4*)
    - Scheduling
      - Reminder of upcoming clinic visit date/time
  - Visit #3 (Antoine-Helen Team)
    - Questionnaires & forms (*For list, refer to chapter 4*)
    - Female participants only
      - Warned about possibility of risk due to an fMRI scan during pregnancy.
      - Asked if she is pregnant or believes there may be a chance of pregnancy
      - A pregnancy test is offered
      - If female participant will not sign the portion of consent form that asks about pregnancy and will also not take a pregnancy test, she will not be included in the fMRI portion of the study
      - Pregnant participants will not participate in the fMRI scan
    - fMRI Scans (*For runlog, refer to Chapter 3*)
      - Prepare participant for scan
      - Heat Stimulation Regulation Task
      - Emotion Regulation Task
    - Scheduling
      - Reminder of upcoming clinic visit date/time
      - Forms for payment # 3 completed & placed in Donna Cole’s mail box on the second floor
  - Visit #4
    - Blood Draw #3
    - Forms for payment # 4 (bonus payment) completed & placed in Donna Cole’s mail box on the second floor

**Chapter 2**

Description of Study Procedures

**Description of Study Procedures**

# MBSR Teacher Criteria

The following is excerpted from the UMASS 2004 MBSR guidelines.

**THE CENTER FOR MINDFULNESS IN MEDICINE, HEALTH CARE, AND SOCIETY**

**UNIVERSITY OF MASSACHUSETTS MEDICAL SCHOOL**

**MINDFULNESS-BASED STRESS REDUCTION:**

**QUALIFICATIONS AND RECOMMENDED GUIDELINES FOR PROVIDERS**

**By:**

**Saki F. Santorelli, EdD, MA**

# **Executive Director, Center for Mindfulness**

**Director, Stress Reduction Program**

[These guidelines have been adjusted by Katherine Bonus, M.A., Cindy McCallum, and Donal MacCoon, Ph. D. to more accurately reflect the program offered by the University of Wisconsin-Madison: Integrative Medicine Mindfulness Center for the current MBSR-HEP empirical trial. These changes are highlighted when possible]

**Center for Mindfulness in Medicine, Health Care & Society**

**2001 © Revised 2004**

**GUIDELINES FOR ASSESSING THE QUALIFICATIONS OF**

**MINDFULNESS-BASED STRESS REDUCTION (MBSR) PROVIDERS**

#### Background

The following recommended qualifications and guidelines for MBSR providers are used by the Center for Mindfulness in Medicine, Health Care, and Society (CFM) at the University of Massachusetts Medical School. In the early 1980’s, as the clinic of origin (The Stress Reduction Clinic) we assumed primary responsibility for the initial establishment and elucidation of these guidelines. In 1996, as MBSR programs and providers began to proliferate, Drs. Jon Kabat-Zinn and Saki Santorelli collaborated with member colleagues of The Northern California Advisory Group on Mindfulness and Medicine to further refine these guidelines (see Standards of Practice). In 2004, Saki Santorelli, Executive Director of the Center for Mindfulness, updated the current edition.

**Mindfulness Meditation and Mindfulness-Based Stress Reduction (MBSR)**

Mindfulness meditation is a highly refined, systematic attentional strategy aimed at developing both stability of mind and body (technically referred to as s*amatha*) and deep insight (technically referred to as *vipassana*) into an array of mental and physical conditions that inhibit an individual's capacity to respond effectively and pro-actively in demanding, highly charged or more commonplace everyday activities. Mindfulness, our innate capacity to flexibly and fluidly pay attention from moment-to-moment, is a universal human capacity taught within a contemporary health context as *mindfulness-based stress reduction* (MBSR). Framed within the context of medical and preventive health services, mindfulness is aimed at assisting individuals to develop an array of self-regulatory, self-care skills. The effectiveness of Mindfulness-Based Stress Reduction (and in our opinion, other mindfulness-based interventions) is predicated on providers who are:

- experientially and substantively grounded in mindfulness meditation practice,
- capable of effectively and flexibly utilizing MBSR as a method for assisting people who are suffering from stress, pain, and illness, and
- committed to ongoing growth and learning that includes a consistent, daily mindfulness meditation practice, continued engagement in extended mindfulness meditation retreats, and active participation in continuing professional education, training, and development.

Because there is an inherent weakness in assessing a person's capacity to provide MBSR when criteria are based exclusively on the fulfillment of a listed set of minimum standards, to be effectively implemented, these guidelines require further clarification. Our experience strongly suggests that there are individuals who meet and sometimes exceed the minimum standards but are incapable of effectively teaching MBSR. Conversely, there are individuals who do not meet the minimum standards but who have developed, via their unique life trajectories, the qualities of a teacher and are capable of effectively elucidating and delivering MBSR. Taking this experience into account and to further clarify an MBSR provider's potential readiness, three domains should be taken into account when assessing an individual's capacity to provide MBSR. These are: *education, life experience,* and *ability*. Considered in aggregate, these domains as described below offer a more precise, operational method for establishing the readiness and competency of an MBSR provider.

#### MBSR Providers: Qualifications and Recommended Guidelines

Two levels of MBSR providers are delineated:

- **Level I**: Provider meets the minimum requirements of entry-level MBSR teachers.
- **Level II:** Provider exceeds minimum requirements and is distinguished by additional experience and training in MBSR.

All MBSR providers shall meet the following minimum standards or equivalent

**Level I Providers: Minimum Qualifications**

**Education:**

Master's degree or equivalent in social sciences, health sciences, education, or related field

**Experience:**

1. A daily meditation practice
2. Three years of consistent mindfulness meditation practice
3. Two cloistered, silent, teacher-led, mindfulness meditation retreats (each retreat

5-10 days). *

1. Three years of *Hatha* yoga and/or body-centered awareness disciplines
2. Two years teaching stress reduction and yoga or body-centered disciplines in a group setting
3. Completion of one 5 or 7-Day *Professional Training Program in Mindfulness-Based Stress Reduction in Mind-Body Medicine* sponsored by the Center for Mindfulness (CFM) and led by Dr. Jon Kabat-Zinn and Dr. Saki F. Santorelli .
4. Participant-observer in at least one 8-week Mindfulness-Based Stress Reduction program conducted by an experienced MBSR teacher. **
5. Strongly recommended: student teaching or apprenticeship under the guidance of a Level II MBSR Provider before teaching MBSR on one’s own. ***
6. Four letters of recommendation - two from professionals who have participated in programs and workshops taught by the Provider; two from lay people who have participated in Provider programs and workshops.

* Mindfulness meditation as utilized in MBSR most closely resembles the Vipassana (Insight) meditation practices taught in the Theravada Buddhist traditions. Therefore, we highly recommend that an individual intent on teaching MBSR participate in several meditation retreats in this tradition even if they are primarily grounded in another meditative tradition or consciousness discipline.

** Utilized since 1981, this approach provides newer MBSR teachers a valuable, first-hand experience of being both a program participant and, in parallel, a professional carefully observing the competencies, attitudes, and qualities embodied in a seasoned MBSR instructor. Used effectively, this methodology functions as a self-reflective, learner directed experience that can assist teachers-in-training to become skillful MBSR providers.

*** In the late 1980’s the Center for Mindfulness adapted this model for teacher preparation and readiness. To date, it has proven to be an effective approach for MBSR teacher training,

providing new teachers with 1) direct mentoring, 2) timely feedback about teaching style and approaches observed in the classroom by the mentor teacher, 3) skill building suggestions critical to new teachers learning how to assist MBSR participants to more readily integrate mindfulness practices into their everyday lives and situations, and 4) instruction and feedback aimed at developing and enhancing competency in the areas of teacher-student dialogue and mindfulness-based critical inquiry that is a hallmark of MBSR.

**Ability:**

- Proven ability to translate mindfulness practice into practical, readily accessible language
- Strong commitment to ethical integrity and honesty regarding the establishment and maintenance of appropriate participant-practitioner boundaries, attention to cultural sensitivity and respect for diversity, commitment to becoming increasingly aware of the array of personal conditioning and habits that lead to poor judgment and inappropriate actions, and a willingness to discuss openly and in-depth these issues with colleagues
- Demonstrated emotional maturity and experience which provide the basic skills for managing a diverse adult patient population presenting with a wide range of life-style related health issues and/or medical diagnoses
- Well-developed group facilitation skills
- Demonstrated ability to establish effective interpersonal relationships with client population
- Ongoing affiliation with an MBSR Professional Group

**Minimum Qualifications: Level II Providers**

Level II Providers meet the minimum requirements of Level I Providers plus the following:

**Education:**

- Master's degree or equivalent in social sciences, health sciences, education, or related field,
- Completion of the *Practicum in Mindfulness-Based Stress Reduction* (Formerly: *Professional Internship Program (PIP)* and/or the *Teacher Development Intensive* (TDI) conducted by the Center for Mindfulness in Medicine, Health Care, and Society at the University of Massachusetts Medical School.

**Experience:**

- Five years of consistent mindfulness meditation practice
- Five extended intensive meditation retreats (5-10 days in duration)
- 200 hours of teaching Mindfulness-Based Stress Reduction (MBSR) in a group setting

**Ability:**

- Proven ability to work both independently and collaboratively
- Demonstrated commitment to pursue professional development in the field of mindfulness-based stress reduction
- Proven ability to communicate effectively with physicians and other health care providers

Katherine Bonus, M.A. and Cindy McCallum are level II instructors. Cindy McCallum has practiced for 22 years as a social worker, considered as the equivalent of master’s level work by the UW-Health Integrative Medicine Mindfulness Program.

**Mindfulness-Based Stress Reduction Provider Screening Criteria**

1. Commitment to adhering to the ethical and moral principles and the domains of non-harming, compassion, and wisdom that underlie mindfulness practice and MBSR.
2. Commitment to provide a professional, clean, safe environment with access for disabled persons (OOE)

# HEP Teacher Criteria

GUIDELINES FOR ASSESSING QUALIFICATIONS OF HEP PROGRAM PROVIDERS

The following qualifications and guidelines for Health-enhancement Program (HEP) providers are used by the University of Wisconsin Integrative Medicine program at the University of Wisconsin—Madison. Jude Sullivan, M.S. played a major role in the development of HEP. In addition to Jude Sullivan, these guidelines and HEP were developed by Zac Imel, M.A., Antoine Lutz, Ph.D. and Donal MacCoon, Ph.D. Instrumental in consulting on these guidelines were Katherine Bonus, M.A., Jackie Kuta Bangsberg, M.S., Pam Polcyn, and Julie Thurlow To facilitate its use in research, the HEP program was designed to be structurally similar to Mindfulness Based Stress Reduction (Kabat-Zinn, 1990) while representing a truly stand-alone, bona-fide treatment intended to increase health and well-being by incorporating health practices known to have positive benefits.

HEP in the UW Health Integrative Medicine Department is intended for anyone wishing to enrich their personal life experience. Through this program, individuals have the opportunity to experience an integrative program that blends contemporary western and traditional therapies designed for both therapeutic and preventive wellness.

The program offers participants to experience individual techniques taught in a group setting in an environment that is open, accepting and non-judgmental. Education about the disciplines is important. The origin and history of each discipline are described along with a didactic description of its current scientific understanding and research showing its efficacy. A proficient expert then demonstrates the technical aspects to the group. This demonstration evolves into teaching a self-practice. With self-application of the techniques, one is taught to understand the nuances of this self-care and the need for regular weekly practice. Individuals are encouraged to share their personal experiences with the group as they feel comfortable.

Given the importance of provider characteristics for treatment outcomes (e.g., Wampold & Brown, 2005), we view the qualifications and approach of the providers to be of paramount importance in HEP. Thus, the approach taken here is to carefully choose providers and allow them to use an intuitive approach to their instruction that can evolve to provide what is needed at the time it is needed. In other words, these guidelines are meant to be flexibly applied by competent professionals who are encouraged to use their professional and clinical judgment to adjust course components, process, or content according to the needs of their particular class and their individual students on a given day. Because of the importance placed on the provider, the qualifications detailed in “qualifications and recommended guidelines for providers” are not sufficient. We believe it likely that some individuals would meet these qualifications but remain unsuitable for teaching HEP. Choice of HEP providers should include awareness of the provider characteristics shown by research to affect treatment outcomes.

## Kabat-Zinn, J. (1990). *Full Catastrophe Living: Using the wisdom of your body and mind to face stress, pain, and illness*. New York: Delacorte Press.

Wampold, B. E., & Brown, G. S. (2005). Estimating Variability in Outcomes Attributable to Therapists : A Naturalistic Study of Outcomes in Managed Care. *Journal of Consulting and Clinical Psychology, 73*(5), 914-923.

## HEALTH-ENHANCEMENT PROGRAM:

## QUALIFICATIONS AND RECOMMENDED GUIDELINES FOR PROVIDERS

### Nutrition

The following Standards of Practice were developed to guide the practice of Nutrition.

- Complete a minimum of a bachelor’s degree at a U.S. regionally accredited university or college and course week approved by the Commission on Accreditation for Dietetics Education (CADE) of the American Dietetic Association (ADA).
- Complete a CADE-accredited supervised practice program at a healthcare facility, community agency, or a foodservice corporation, or combined with undergraduate or graduate studies. Preferably, a program running 6-12 months.
- Pass a national examination administered by the Commission on Dietetic Registration (CDR).
- Complete continuing professional educational requirements to maintain registration.
- Hold an additional certification in nutritional programming for integrative medicine.

For more background information about Nutrition experts, go to:

American Dietetic Association

120 South Riverside Plaza

Suite 2000

Chicago, Illinois 60606-6995

(800)-877-1600

[http://www.eatright.org](http://www.eatright.org/)

Julie Thurlow, zz, the instructor for the nutrition component of HEP, meets or exceeds these standards.

### Physical Activity & Functional Movement

The following Standards of Practice were developed to guide the practice of Physical Activity (PA). An exercise physiologist who is committed to the highest standard of care will serve as a role model by practicing exemplary behaviors when working with customers and colleagues. Cooperation with other experts is expected while at the same time assuming teaching responsibilities are integral to the success of this program. The program serves a diverse clientele and problems encountered can be challenging and require a high level of independence in decision making. Creativity, innovation and good communication skills are necessary. A high degree of organizational and interpersonal skills is required for the position.

The PA expert is capable of performing the following clinical responsibilities as needed:

- Masters degree in exercise science, exercise physiology, kinesiology or related field
- Extensive work experience in a hospital based health/fitness facility
- Two years cumulative clinical experience in broad based programming – fitness, cardiac rehab, integrative medicine
- Risk assessment to determine appropriateness and readiness for lifestyle enhancement
- Administer submaximal and maximal exercise test on low and high risk populations
- Demonstrate mastery in the use of testing equipment, protocol selection, indications and contraindications, and termination criteria for exercise testing
- Record, analyze, and interpret results of tests and prepare appropriate lifestyle recommendations
- Demonstrate mastery in utilizing psychological interviewing skills to mentor clients as they seek to adopt a healthier lifestyle
- Nationally recognized certification in lifestyle coaching curriculum – completed or in process
- Experience working with a wide array of the public
- Excellent communication and interpersonal skills with both individuals and groups
- Current Health Care Provider CPR-BLS certification or higher

For more background information about Physical Activity experts, go to:

The American College of Sports Medicine

P.O. Box 1440
Indianapolis, IN 46206-1440

(317) 637-9200
(317) 634-7817 (Fax)

[http://www.acsm.org](http://www.acsm.org/)

Jude Sullivan, the instructor for the physical activity and functional movement components of HEP, meets or exceeds these standards.

**Music Therapy**

Persons who complete one of the approved college music therapy curricula (including an internship) are then eligible to sit for the national examination offered by the Certification Board for Music Therapists. Music therapists who successfully complete the independently administered examination hold the music therapist-board certified credential (MT-BC).

The National Music Therapy Registry (NMTR) serves qualified music therapy professionals with the following designations: RMT, CMT, ACMT. These individuals have met accepted educational and clinical training standards and are qualified to practice music therapy. [www.musictherapy.org](http://www.musictherapy.org/)

The recertification cycle is a five-year period following successful completion of the music therapy board certification examination, during which the Music Therapist-Board Certified (MT-BC) must fulfill requirements for recertification to assure continued competence in music therapy. The end of the cycle corresponds to the expiration date of the certificant’s current certificate, issued by the Certification Board for Music Therapists (CBMT).

To be recertified, the MT-BC must meet the following conditions by the end of their five-year cycle:

1. Complete a [Summary of Music Therapy Education Activities Form](http://www.cbmt.org/default.asp?page=Summary Form), with 100 CMTE credits claimed, or take the CBMT Exam in the fourth year of their five-year cycle. Exam information may be found at [CBMT Examination Information](http://www.cbmt.org/default.asp?page=CBMT Examination);
2. Pay all maintenance fees;
3. Participate in the Annual Audit of CMTE credits, if selected;
4. Complete remedial CMTE credits if required as a result of utilizing the exam option;
5. Complete and submit the
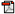
[Application for Recertification](http://www.cbmt.org/Application_for_Recertification.pdf)

Source: [www.cbmt.org](http://www.cbmt.org/) (May, 2006)

Pam Polcyn, the instructor for the music therapy component of HEP, meets or exceeds these standards.

Advertisement

Newspaper Advertisement

Flyer

Criteria of Exclusion & Inclusion

**1) Inclusion criteria**

1.1 Risk Factors

- Able to lie still in the scanner for 90 minutes
- Weighs under 300 pounds

1.2 Factors impacting on the outcome measures

- 18-65 years old
- Right-handed
- No previous experience with meditation.
- No daily practice with other mind-body techniques (e.g., yoga, tai-chi, but previous exposure to yoga is okay)
- In good general health as determined by the investigator

1.3 Logistic Issues

- Able to walk
- Able to understand and speak English
- Able to provide written consent prior to admission
- Able to see without glasses (as if looking through binoculars)

**2) Exclusion Criteria**

2.1 Risk Factors

- Any metallic implants, such as prostheses that cannot be removed, aneurysm clip-S, dental or orthodontic (filings are okay), or orthopedic (e.g., pins, rods, screws, nails) [okay with doctors note]
- Any electronic implants, such as cardiac pacemakers, cardiac defibrillator, artificial heart valve, cardiac pace wires,
- Lens implants prior to 1983
- Breast/penile, electrodes, neurostimulators/biostimulators, pumps (e.g. drug infusion device), bone or socket implants (Check with PI or MR technician)
- Any type of prosthesis
- Any permanent cosmetics (e.g., eyeliner) prior to 1975
- Any tattoos on upper body prior to 1975
- Any irremovable non plastic body piercing(s)
- Has ever worked as an occupational metal grinder without protective glasses
- Has ever worked with metal as a hobby without protective glasses
- Has ever had or sought medical attention for metal in the eyes [requires orbit x-ray]
- Has ever has metal fragments removed from eyes [requires orbit x-ray]
- Has ever been struck by a gun shot, B.B. or shrapnel, and the bullet stuck [judgement]
- Has a history of any metal in body
- Diabetes
- Peripheral vascular disease, peripheral arterial disease, Raynaud's disease, or any other
  diagnosed circulatory disorders
- Body mass index (BMI) below 18.5
- Any involuntary motor disorders
- Has a cochlear implant
- Wears a hearing aid that cannot be taken out (Check with PI or MR technician)
- Wears a hearing aid
- Wears dentures that cannot be removed or contain metal
- A subject who is claustrophobic
- A female subject who is pregnant or has an intrauterine device (IUD)
- Allergic to adhesive tape
- A history of problems of any kind during blood draws
- Women who may be pregnant
- Women who have an intrauterine device (IUD) [okay with a doctors note]
- 2 or more of the following [judgment]
  - Diagnosed hypertension?
  - Hyperlipidemia
  - High cholesterol
  - Obesity can be calculated from weight & height info.
  - Smoke cigarettes
  - Family history of coronary or atherosclerotic disease (parents/siblings prior to age 55)

2.2 Factors impacting on the outcome measures

- Current medical disorders that might make interpretation of scan data difficult
- Has a problem with alcohol or non prescription drugs
- Use of medications that affects CNS function, including psychotropics, opiate medication or corticosteroids, during the last 3 months
- Any prescribed psychotropic medications, currently or during the last 3 months (These include medications for anxiety, depression, or other psychological problems)
- Plans to start taking any prescribed psychotropic medications
- Takes inhaled steroids for asthma? (e.g., Fluticasone)
- Takes any corticosteroids?
- Night shift workers (11 pm to 7 am) d/t potential disruption of cortisol level variability
- Diabetes requiring insulin treatment
- Has TMJ (Temporal Mandibular Joint) disorder or other problems with biting/chewing
- Previous training in meditation
- Currently meditates on a regular basis
- Daily yoga, tai-chi, or Qigong practice
- Engagement in moderate sport and recreational activities more than 5 times a week
- Engagement in vigorous sport and recreational activities more than 4 times a week
  1. Logistic Issues
- Phobia of needles
- Not able to attend an informational session, all class meetings, and all clinic visits

**(NOT USED IN THE STUDY)**

**Screening Criteria for Exclusion from the SRP [These are the criteria used by UMASS for their MBSR classes. The following is taken from their 2004 guidelines]**

MBSR is a practical educational approach rather than a conventional group therapy intervention. As such, the current study will involve 13 participants, is time-limited (8-weeks), and relies on the creation of a highly participatory community of learners intent on cultivating and integrating into their everyday lives the various practices, approaches, and attitudes characteristic of MBSR (see Kabat-Zinn excerpt “Mindfulness-Based Stress Reduction”). Its primary focus of attention is directed toward the development of a person’s first hand understanding of the body, mind, and body-mind interactions leading to the incremental development of greater somato-psychic awareness that can be fluidly integrated into the life of program participants as a means of coping more effectively with the challenges and demands of everyday life.

Keeping in mind the structure, methods, and key characteristics of MBSR as previously described, the following screening criteria for exclusion were informally established with the founding of the Stress Reduction Program in 1979. These criteria were formalized, updated and refined in 1993, 2001 and again in 2004.

***Lifestyle Issues:***

- - - - Active substance dependence - legal or illicit
      - People with substance dependence new to recovery (less than one year)
      - Inadequate comprehension of language in which the course is taught

*Exceptions*: In terms of addiction:

- If someone is in recovery less than one year and seems highly motivated or is in a highly supportive treatment environment that is congruent with the approach used in the Stress Reduction Program, they may be considered for program participation.
- If a program candidate is dependent to pain medication for a chronic pain condition and is capable of mounting the requisite mental concentration and energy required to learn and practice the meditation and mindfulness practices utilized in the SRP, they may be considered for program participation.

If there is a problem with language comprehension, provisions can be made for interpretation services. The same holds true for hearing impairment. While we attempt to arrange for these services, we cannot guarantee that these services will be available to Stress Reduction Program (SRP) candidates.

# *Psychological issues:*

- Suicidality
- Psychosis (not treatable with medication)
- PTSD
- Depression (clinical) or other major psychiatric diagnosis (if it interferes with participation in the SRP).
- Social anxiety (difficulty with being in a classroom situation)

*Exceptions*: Anyone who is highly motivated and receiving therapy and/or medication for the above mentioned diagnoses may be considered for enrollment in the Stress Reduction Program. However, in these cases, it is essential that:

- Prior to the start of classes we receive permission from program candidates to speak with their primary mental health provider. We require the same pre-program permission to speak with the program candidate’s primary care physician when deemed appropriate,
- Both the program candidate and primary mental health provider understand and agree that the mental health provider is the primary care giver and first contact person in the case of mental health emergency rather than the SRP instructor.
- Appropriate SRP program staff maintain active and on-going collaboration with the program candidate’s current mental healthcare provider and primary care physician (when deemed necessary) during the duration of the program.

# *Attitudinal Issues:*

- Inability to comprehend the nature and limitations of program (wanting a “quick fix” without the necessary and required investment of time and energy)

- Inability to commit to attending classes (if someone is going to miss three or more classes they are referred to a future program cycle)

***Physical Issues****:*

- Inability to physically attend weekly program classes. This does not refer to physical impairment, which is not an exclusion criterion. Rather, this refers to the inability of program candidates to actually get to class because of being bedridden or homebound or without transportation.

Note: In rare instances, home visits for individual instruction may be arranged. The use of this service is based on SRP instructor availability. Fees for this service differ significantly from usual program costs.

***In all cases, final decisions regarding these exclusion criteria are subject to the judgment of the instructor conducting pre-program Information sessions.***

Information Session Content

Four information sessions will be held after participants have indicated an interest in participating in the study. Attendance at an *Information session* is required for all incoming participants. These sessions are two hours in duration. During this time, researchers will present the consent form and answer questions about the study. People who agree to participate in the study will sign the consent form. MBSR and HEP instructors will then describe their respective programs in detail and people are given the opportunity to speak about their lives and their reasons for considering participation in the program. Discussions are usually quite lively and informative. Instructors conducting these sessions have the opportunity to have individual exchanges with participants who either ask for or require more individual attention.

To better prepare participants for the both programs, prior to attendance at an *Information session* all participants receive information describing the structure and key characteristics of both programs with information that describes commitment required of participants.

## *Schedule*

Time: 6:30 – 8:30 PM

6:30 p.m. Participants begin arriving and staff members greet people and direct them to our meeting room.

6:40 p.m. Researchers welcome potential participants, hand out copies of the consent form, and review the consent form verbally. Any questions are answered.

7:20 p.m. Instructors from programs outline the session format and class details in 30-minute presentations for each class. This will be followed by questions and discussion and participants have an opportunity to speak about their intentions and reasons for considering taking the course.

After completing these phases of the *Information session,* those attendees who decide to enroll in the study will be asked to sign the consent form and provide contact information.

8:25 p.m. Hand out cortisol kits and instruct participants on their use

Hand out Physician Authorization Form (participants bring to first research visit)

Hand out registration form for HEP and MBSR (participants bring to first research visit)

Hand out complete the Medical History & Physical Activity History Form (participants bring to first research visit)

MBSR & HEP Instructors:

- Anyone need to be eliminated? (BMI, bad fit, etc.)
- Include in presentations practical information for participants (e.g., what to wear, what to bring, etc).

Randomization Procedures

Participants will be assigned to the MBSR and HEP after the information sessions, once they have signed up for all of their clinic visits for the first time point. However, the participants will not be informed of group assignment until after the end of pre-intervention data collection. During the entire data collection phase of the study (including two post-intervention time points), the experimenters will be blind to participants’ group assignment. The person who will do the randomization will be not participating in the collection of the data. The randomization will be done after the last information session on the pool of participants who signed the consent form. As gender may affect some outcome measures (in particular salivary cortisol), and as this exploratory study will not investigate gender differences, each group will be stratified with respect to gender (forcing balance) during this randomization.

Power Analyses

**Power analysis for the fmri tasks:**

We will collect fmri data from 26 participants. Yet, we compute a power analysis for a conservative sample size of 16 participants forecasting dropouts of participants due to motion artifacts or retraction from the study. The following analysis uses the statistical distribution for the BOLD signal of two recent fmri studies from our lab. The first study, a methodological one, studied the stability of amygdale BOLD response to fearful faces over multiple scan sessions (Johnstone et al. 2004). This study showed that the stability through three sessions of the average percentage BOLD signal change measured between the presentation of fear faces and the baseline (mean 0.17 %,0.15% and 0.16%, respectively). A similar finding was reported for the standard deviation (0.19 %, 0.22 %, 0.25% respectively). From this study we anticipate that the BOLD signal in the brain sensory areas during the presentation of our painful thermal stimuli or our visual stimuli will be stable through the three sessions (pre intervention, post intervention time 1 and time 2). Furthermore, we expect a minimal effect of habituation to the tasks as a consequence of the training session in the mock scanner prior to the collection of the data. If there is a variation through time in the pain or compassion neural circuitry during our task, it is thus likely to be a result of the clinical intervention. Thus, we expect to reduce within-participant error by taking advantage of the stability of the BOLD signal across time. The effect of meditation training was estimated from a second study (Lutz et al. under submission). In this study, we showed that the state of compassion meditation enhances the brain response to a stressful auditory sound in several brain regions and that this effect was modulated by the degree of training to meditation. This study thus suggests that meditation training can modulate the brain response to sensory event and supports our main assumption for this new fMRI study. We used this study to estimate the possible impact of mental training on sensory stimulation (thermal stimulation or visual stimulation). In the inferior parietal cortex for instance, the difference in the mean percentage of increase from neutral to meditation between expert meditators and novices was 0.18% with a standard deviation of 0.15%. By looking at the group difference between pre and post intervention time1, the main effect of the study can be reduced to a ttest comparing two independent groups. If it is assumed that the between-subjects variance measured in the previous study on meditation is a good approximation of the population variance in this area, then one can estimate that the effect size *d* is equal to 1.125. From there, one can estimate that the degree of noncentrality *δ* for a conservative sample size of N=16 is *δ=d*sqrt(N/2)=3.2*. Then, from statistical tables, one can estimate that the power is 0.81 using a two-tailed test at *α=0.02* and for *δ=3.2*. From this analysis we predict that a conservative sample of N= 16 participants in each group will enable rejection of the null hypothesis at an alpha of 0.02.

Johnstone T, Somerville LH, Alexander AL, Oakes TR, Davidson RJ, Kalin NH, Whalen PJ. Stability of amygdala BOLD response to fearful faces over multiple scan sessions. Neuroimage. 2005 May 1;25(4):1112-23.

**Power analysis for TSST**

The Trier Social Stress Test (TSST) was designed to reliably produce a significant increase in salivary cortisol at the individual level, and is certainly potent enough to produce significant mean increases with a sample size of 50, or 25 per group (Kirschbaum *et al.*, 1993).

The size of the effect that training in mindfulness-based stress reduction (MBSR) has on the physiological stress response is unknown. However, several studies with similar sample sizes (range 11-125 per group) have had sufficient power to detect buffering effects (range of cohen’s *d* = .3-1) of MBSR training on indices of psychological stress (e.g. Majmumdar *et al.*, 2002; Tiefenthaler-Gilmer, 2002; for review, see Grossman *et al.*, 2004). Moreover, it is worth noting that psychological indices of stress tend to be far less sensitive than physiological indices. In addition, other forms of stress-reduction interventions have been shown to produce significant reductions in cortisol levels (e.g. Wardell & Engebretson, 2001; *d* = .35) with a similar or smaller sample size (n= 23). Therefore, using a conservative estimate of effect size at the low end of those reported in Grossman *et al.*  (*d* = .35; psychological indices of stress), a sample size of 25 per group will provide moderately high power (.65) to find an effect of MBSR training on cortisol levels.

References:

Majumdar ­*et al.* Does mindfulness meditation contribute to health: Outcome evaluation of a German sample. *Journal of Alternative and Complementary Medicine* 8(6): 719-730, 2002.

Tiefenthaler-Gilmer, U. Mindfulness meditation as clinical intervention: A controlled study of a mindfulness-meditation program for fibromyalgia patients. Dissertation, University of Vienna, Austria (2002).

Grossman *et al.* Mindfulness-based stress reduction and health benefits: A Meta-analysis. *Journal of Psychosomatic Research* 57: 35-43 (2004).

Kirschbaum, C., Pirke, K.M. & Hellhammer, D. H. The "Trier Social Stress Test" - a tool for investigating psychobiology stress responses in a laboratory setting. *Neuropsychobiology* 28: 76-81, 1993.

Wardell DW. Engebretson J. Biological correlates of Reiki Touch(sm) healing. *Journal of Advanced Nursing* 33(4):439-45, 2001 Feb.

**Power analysis for behavioral tasks**

Effect sizes for a Continuous Performance Task (CPT; Cohen, Barch, Carter et al., 1999), an attentional blink task (ABT; Raymond, Shapiro, & Arnell, 1992), an implicit association task (IAT; Greenwald, McGhee, & Schwartz, 1998), and a measure of mind wandering (Schooler, Reichle, & Halpern, 2004) vary from 9% (some group effects) to around 50% (task effects). However, we know of no data relevant to MBSR training for these tasks. The most relevant data we have is from our own laboratory involving the use of the ABT with mindfulness practitioners and controls before and after training. The effect size for this result (very similar to what we would test in the current study) was R^2^ = .20. Given this effect size, our current sample should be sufficient to achieve at least a power of .80.

References:

Cohen, J. D., Barch, D. et al. (1999). "Context-Processing Deficits in Schizophrenia : Converging Evidence From Three Theoretically Motivated Cognitive Tasks." Journal of Abnormal Psychology **108**(1): 120-133.

Greenwald, A.G., McGhee, D.E., & Schwartz, J.L.K. (1998). Measuring individual differences in implicit cognition: The implicit association test. Journal of Personality and Social Psychology, 74, 1464-1480.

Raymond, J. E., Shapiro, K. L., & Arnell, K. M. (1992). Temporary suppression of visual processing in an RSVP task: An attentional blink? Journal of Experimental Psychology: Human Perception and Performance,18, 849–860.

Schooler, J.W., Reichle, E.D., Halpern, D.V. (2004). Zoning out while reading: Evidence for dissociations between experience and metaconsciousness. In D.T. Levin (Ed) Thinking and Seeing: Visual Metacognition in Adults and Children (pp. 203-226) Cambridge, MA. MIT Press.

Organization of Data Files

There are electronic and paper files.

Paper Files

All participant paper materials will be kept in a central locked location. Files will consist of one green hanging folder labeled with ID # and several manila folders within this hanging folder. These manila folders correspond to each component of the HEP-MBSR study:

- Information Session
- Logistics
  - Recruiting
  - Payment information
  - Visit schedules
- Pain &Emotion Regulation Task
- AX-CPT Task
- TSST
- Blood Draw

Electronic Files

All files will be kept on a secure server located at the Keck Laboratory.

Payment of Participants

- The participants will be paid at four time points during the study

1. After the 3rd clinic visit is completed ($75)

2. After the 6^th^ clinic visit is completed ($75)

3. After the 9^th^ clinic visit is completed ($75)

4. After the 10^th^ clinic visit is completed ($250)

- Payment forms will be filled out by the participants at the first visit of each of the three phases and at the last clinic visit.

- The study coordinator will complete the form and turn it in to Donna Cole, the lab manager, when the participant has finished all of the visits within each phase.

#### Chapter 3

Runlogs

**Runlogs**

Voice Mail Runlog

**Voice Mail Protocol:**

1. This can be done in the Keck Analysis Bay. There is a wireless phone in this room that you can use. It is on the desk below and to the left of the digital clock that is on the wall.
2. Open the **Master Logistics Spreadsheet** (Y:\MBSR\Logistics\Master Logistics Spreadsheet)

To get into the Y directory:

Double click on the “MyComputer” desktop icon

Double click on “study on 'chipotle' (Y:)

You are now in the correct directory

1. In the excel document make sure that the “Recruitment-Phone” tab near the bottom left hand side of the screen is selected.

Voice Mail Tips & Tricks

While listening to a message dial the following to:

1 = Rewind 10 secs

2 = Pause

3 = Fast forward 10 secs

1-1 = Return to beginning

3-3 = Fast forward to end

0-4 = Replay (at end of message)

1. Using the telephone, check the voicemail messages:
   1. Dial **9**
   2. Dial **262-6100** (wait for prompt)
   3. Press # (**pound**) (wait for prompt)
   4. Enter Mailbox Number: **262-9888** (You should hear, “*Laboratory for Affective Neuroscience Subject Recruitment Line”*)
   5. Enter Password: **39461** (If you make a mistake beyond this point and/or want to start over, press 5)
   6. Wait to hear the Operator say, “*Main Menu*” and gives options
   7. **Press 1-1** – “*to* *hear new messages”*
   8. **Record the information** provided in the message into the Phone Screening Record.

🟋 If someone else is working on the MLS at the same time, it will be necessary for you to copy and paste your updates into the most up-to-date version when you are done. If this is the case please ask Jenna for an explanation of the appropriate way to record the information.

- - - If the caller is already listed in the Phone Screen Record, check over the information that is already there and fill in any gaps that you are able to fill in
    - If some information was not provided type “Not Given” in the appropriate column
    - To *replay* the message, wait for the message to end, **Press 0, and then press 4** (In case you weren’t able to record all of the information the 1^st^ time)
    - If you cannot understand all or some of the message, select the word that you did not understand and make the text red. If you couldn’t make out the work at all insert three question marks in red font (???) where the word would go.
    - Any further information can go in the “Voicemail Notes” column (please leave your initials in parentheses after any comment in the notes section)
  1. **Press 7** to delete the message *IF*:
     1. You are certain that you recorded the available information correctly
     2. You were able to clearly make out & correctly record the phone number, if given
     3. If no phone number was given, BUT you were able to clearly make out & correctly record an e-mail address
     - **If you have any doubts, do not erase the message and clearly record your reasons in the “Voicemail Notes” column and contact Jenna (MBSRstudy@gmail.com)
     - Missed or incorrect information could result in losing a potential participant. Given the short amount of recruiting time that we will have, it is important that this does NOT happen

E-mailing Runlog

**E-mail Protocol:**

- This can be done in the Keck Analysis Bay.
- You will need to use two computers.
- On the computer directly in front of you open the **Master Logistics Spreadsheet** (Y:\MBSR\Logistics\Master Logistics Spreadsheet)

To get into the Y directory:

Double click on the “MyComputer” desktop icon

Double click on “study on 'chipotle' (Y:)

You are now in the correct directory

🟋 If someone else is working on the MLS at the same time, it will be necessary for you to copy and paste your updates into the most up-to-date version when you are done. If this is the case please ask Jenna for an explanation of the appropriate way to record the information.

- One the other computer open up the “Recruitment E-mail” document (Y;\MBSR\Logistics\Recruiting\Recruitment E-mail)

In this document there are four versions of the e-mail:

- - **RECRUITMENT E-MAIL**: Use the following e-mail in response to initial e-mail inquiries that DO NOT CONTAIN a phone number at which to contact the individual.
  - **ABRIDGED E-MAIL**: Use the following e-mail in response to initial e-mail inquiries that DO CONTAIN a phone number at which to contact the individual.
  - **THANK YOU E-MAIL SENT**: Use the following e-mail in response to e-mails CONTAINING a phone number responding to our request for more contact information
  - **REJECTION E-MAIL SENT**: Use the following e-mail in response to e-mails containing information that would automatically exclude him/her for participating in the study (i.e., over the age of 65).

** The instructions for when to use each e-mail are also in the “Recruitment E-mail” document**

- You will copy and paste these versions of the e-mails as responses
- On the same computer as the “Recruitment E-mail” document, open a web browser and go to *www.gmail.com*
- Sign in as: HepMbsrStudy, Password: present (no capital letters)
- ONLY record an individual’s information in the MLS once we have a phone number at which they can be reached. Check for new e-mails and record all the pertinent information in the Phone Screening Record excel document.
- If a response e-mail has new information in it, then find this person in the MLS and update their info.
- Please label each e-mail according to what e-mail you have sent the participant and whether or not we have received a phone number. If you do not know how to label e-mails ask Jenna to show you.
- **Archive** (DO NOT DELETE) the e-mail when you are sure that you have recorded all of the information correctly.

Phone Screening Runlog

🟎**RECORD YOUR HOURS IN THE “Hours” TAB OF THE MLS EVERYTIME YOU WORK**🟎

**REMEMBER:**

1. When you first get in go to the “LUTZ1” cabinet and check to see if there are packets in the “To Be Completed” file. These should be the first individuals that you address
2. When you receive an individuals contact information during a phone call, please make sure to compare it to the information in the MLS to verify that we have their correct information

*** If for some reason you cannot complete the recruitment process with an individual, you MUST:

- - Staple all the packets used together
  - Write a detailed note describing what has happened so far so that someone else can pick up where you left off (post-its are in “LUTZ2” for this purpose)
  - Attach the note to the first page of the screening sheets
  - Place packet in the “To be Completed” green hanging file folder in “LUTZ1”

**Telephone Screening Instructions:**

1. Open file cabinet on the second floor, labeled “LUTZ 1”, there should be green hanging file folders labeled: Phone Screening Runlogs, Phone Script Part 1, and Phone Script Part 2.
2. You will need packets from each of these file folders . *Make sure to lock the file cabinet when you are done!* (key is labeled “LUTZ” and located in the far left, top cabinet in the second floor Keck kitchen)
3. Write you name and the date on top of any packet that you use. This way we will know who recruited which individual.
4. Make sure that the **Master Logistics Spreadsheet** (Y:\MBSR\Logistics\Master Logistics Spreadsheet) is open
   - 1. To get into the Y directory:
     2. Double click on the “MyComputer” desktop icon
     3. Double click on “study on 'chipotle' (Y:)
     4. You are now in the correct directory
5. In the excel document make sure that the “Recruitment-Phone” tab near the bottom left hand side of the screen is selected.
6. Call each person who has left a message or e-mail, (Before calling people who have recently left a message, call those individuals who have already been called, but have not yet been reached as indicated on the Phone Screening Record Sheet. When you do so, replace the name of the RA who attempted before you with your name in the “RA” column)
7. Call potential participant. See Telephone Script.
8. When phone conversation has ended fill in the Recruiting-Phone excel sheet ( Y:\MBSR\Logistics\ Master Logistics Spreadsheet)
   - 1. ** Info Session Dates: (all times are 6:30-8:30 pm)
        - 1. 10-Jul-06: Room 1111A
          2. 11-Jul-06: Room 1111A
          3. 26-Jul-06: Room 1111A
          4. 27-Jul-06: Room 2386
9. *** Those people who will only be available to attend the first 2 sets of class dates should get priority for attendance at the first two info sessions!
10. If participant is INCLUDED:
11. Assign ID for participant
    - 1. Before participants have consented to participation in the study they will be assigned an ID. Find the most recently assigned ID and assign the new potential participant the ID that is next. (e.g. A1, A2, A3….A45, A46)
      2. Write the ID on the upper right hand corner of the screening sheet
      3. Make sure to staple together the two packets
12. Open file cabinet “LUTZ 2” (key is located in the far left, top cabinet in the second floor Keck kitchen)
13. Take out 1 manila folder, 1 labeling sticker, 1 information packet, & 1 envelope
14. Prepare the Informational Packet
    1. Fill in blank spaces on the info session letter:

Info Session Date & Room Number: : (all times are 6:30-8:30 pm)

- - - - 1. 10-Jul-06: Room 1111A
        2. 11-Jul-06: Room 1111A
        3. 26-Jul-06: Room 1111A
        4. 27-Jul-06: Room 2386
  1. Fold information packet and put in envelope. You should be able to find the address in the Phone Screening Record.
  2. Put the sealed letter in the outgoing mail cubby hole in T-115

(It is on your right when you walk into the room)

1. Place a labeling sticker on the manila folder and write the participants ID and Info Session number on the sticker (Sample in “LUTZ2”)
2. Put phone script in this manila folder
3. In the file cabinet on the second floor, labeled “LUTZ 1”, there should be four green hanging file folders labeled: Info Session #1, Info Session #2, Info Session #3, and Info Session #4. Place the manila folder in the green hanging file folder that corresponds to the Info Session that the potential participant has been scheduled to attend. *Make sure to lock the second floor file cabinets when you are done!*
4. If participant is EXCLUDED: delete their name & # from the Phone Screening Record, but leave other information.
5. If participant is JUDGMENT:
   - - - 1. Type “Yes” in the judgment column
         2. Assign ID for participant. These IDs will not be the same as the IDs for those who are included. The IDs for the Judgments should begin with the letter “J” instead of “A”. They will look like the following: J1, J2, J3, …J56, J57.
         3. Write the ID in the upper right hand corner of the screening sheet
         4. Highlight the judgment(s) on the screening sheet so that they are easy to find when others look at the form
         5. Make sure that the MLS contains the contact information that you recorded in the screening form.
         6. Click on the “Judgments” tab
         7. Enter your name, date/time, and the name, ID and phone number of the individual classified as a judgment
         8. Type in the appropriate type of judgment(s) needed to be made (possibly more than 1)

- MRI = anything from the MRI screening form
- Meditation Related =
  - currently meditates on a regular basis
  - meditated daily for at least 2 months
  - daily practice with some sort of mind-body technique
- Unspecific = Neither in the MRI or Mediation Related categories
  - - - 1. Contact the appropriate person(s) to make judgment
- MRI = call Ron Fisher or Michael Anderle at 262-9230. If you do not reach them by phone, DO NOT leave a message. Send Michael an e-mail (E-mail = [manderle@wisc.edu](mailto:manderle@wisc.edu).) from the [HepMbsrStudy@gmail.com](mailto:HepMbsrStudy@gmail.com) e-mail address. (The subject of the e-mail should read: *Important Participant Judgment Needed, Please Read & Respond ASAP(Participant ID)*).
- Meditation Related = E-mail Antoine Lutz ([Alutz@wisc.edu](mailto:Alutz@wisc.edu)) & Donal Maccoon (Dgmaccoon@gmail.com). (The subject of the e-mail should read: *Important Participant Judgment Needed, Please Read & Respond ASAP(Participant ID)*)
- Not Specific = no contact necessary
- Multiple= Contact the appropriate people depending on the combination of judgments
  - - - 1. If the there are multiple judgments to be made, please list these on the top of the first page of the packet.
        2. If judgment decision(s) is not made right away put the file in the appropriate manila folder (MRI, Meditation Related, Unspecific, Multiple ) in the green hanging file in “Lutz1”
        3. Indicate whether or not you have contacted the necessary decision maker(s).
        4. When a decision is made, select the appropriate decision.

🡪Depending on the decision that you select, certain cells in the columns to the right will automatically read “N/A”. You do not need to enter any information in those cells. Only fill in the cells that remain empty.

- - - - 1. Go into the “Recruitment Phone” tab and make sure that the individual’s status as a potential participant (included or excluded) is updated accordingly.

# Script for Calling Back “Judgments”

#

Scripts for calling back “Judgments” to give an update on the status as a potential participant

***(These packets will be found in the “To Do” file once a judgment has been made)

- 1. IF the participant is to be EXCLUDED:
     - 1. Call the participant back and say:

| Hello, my name is _____________. I am an investigator with the laboratory for Affective Neuroscience in Madison, Wisconsin. Recently you were contacted about participating in the study and it was not clear whether you qualified to participate. We have reviewed your information and …  ****From here read the section that is read to all who do not meet the study criteria. |
| --- |

2. Record in the “judgment” tab that the participant has been contacted and change their information in the “Recruiting-phone” tab to reflect that s/he has been excluded and remove all personally identifying information.

- 1. IF participant is to be INCLUDED:
     - 1. Call the participant back and say:

| [Screener]  Hello, my name is _____________. I am an investigator with the laboratory for Affective Neuroscience in Madison, Wisconsin. Recently you were contacted about participating in the study and it was not clear whether you qualified to participate. After reviewing your information it seems that, so far, you do qualify to participate in the study. I would like to read a short description of the study. Is now a good time?  [Potential Participant]  “No, I do not want to be in the study.”  [Screener]  Thank you for your consideration.  Good bye.  NOTE TO SCREENER: Please destroy any and all personally identifying information regarding people who will not be a part of the study.  [Potential Participant]  “No, I don’t have time to talk now”  [Screener]  Is there a better time for me to call to discuss this?  [Potential Participant]  “Yes, a better time to call would be ________________________”  [Screener]  Thank you, I will try to reach you then.  Good bye.  [Potential Participant]  “Yes, I have a few minutes”  [Screener]  Great. Please feel free to interrupt me to ask questions.  ****Continue from the section in the phone script that is on the bottom half of page 9 and begins with “*The University of Wisconsin, Laboratory for Affective Neuroscience, is conduction the study. The study examines…”* |
| --- |

- - - 1. Record that the participant has been contacted
      2. Change his/her information in the “Recruiting-phone” tab to reflect that s/he has been included
      3. Follow the usual steps that are followed for a participant that is included

c. If the participant NEEDS A DOCTORS NOTE

1. Send the potential participant the MRIdoctors note forms and a pre-addressed envelope.
   1. The materials to make this packet are in “LUTZ2” in a green hanging file folder. (A sample package can also be found there)
2. Place a red post-it flag on the top left hand side of the packet (the red post-it flags are in “LUTZ2”)
3. Record that the forms have been sent
   - 1. RETURN CALL AFTER DOCTORS NOTE RECEIVED.
4. When you see that the spreadsheet indicates that a doctors note has been receive, contact the potential participant
5. If you reach the participant select your name in the last column of the “Judgment” tab
6. When you contact the participant say the following:

| [Screener]  Hello, my name is _____________. I am an investigator with the laboratory for Affective Neuroscience in Madison, Wisconsin. We have received a note from your doctor which authorizes your participation in the study. Thank you very much for having your physician to complete the forms and sending them back to us. At this point it seems that you qualify to participate in the study. I would like to read a short description of the study. Is now a good time?  [Potential Participant]  “No, I do not want to be in the study.”  [Screener]  Thank you for your consideration.  Good bye.  NOTE TO SCREENER: Please destroy any and all personally identifying information regarding people who will not be a part of the study.  [Potential Participant]  “No, I don’t have time to talk now”  [Screener]  Is there a better time for me to call to discuss this?  [Potential Participant]  “Yes, a better time to call would be ________________________”  [Screener]  Thank you, I will try to reach you then.  Good bye.  [Potential Participant]  “Yes, I have a few minutes”  [Screener]  Great. Please feel free to interrupt me to ask questions.    ****Continue from the section in the phone script that is on the bottom half of page 9 and begins with “*The University of Wisconsin, Laboratory for Affective Neuroscience, is conduction the study. The study examines…”* |
| --- |

1. Change his/her information in the “Recruiting-phone” tab to reflect that s/he has been included
2. Follow the usual steps that are followed for a participant that is included

*** REMEMBER to make sure that you have documented & initialed everything in the MLS correctly before saving, closing, or leaving!

Pre-screening Telephone Script

[Screener]

Hello, my name is _________________. I am an investigator with the Laboratory for Affective Neuroscience in Madison, Wisconsin. UW Health Integrative Medicine has agreed to let us conduct a study on individuals interested in participating in Mindfulness-Based Stress Reduction (MBSR) or a Health Enhancement Program (HEP) this Fall. The goal of the study is to evaluate the effects of these two programs on pain regulation, negative affect, attention, and social stress. Do you have a few minutes to discuss possibly being a part of the study we are running this year?

[Potential Participant]

“No, I do not want to be in the study.”

[Screener]

Thank you for your consideration.

Good bye.

NOTE TO SCREENER: Please destroy any and all personally identifying information regarding people who will not be a part of the study.

[Potential Participant]

“No, I don’t have time to talk now”

[Screener]

Is there a better time for me to call to discuss this?

[Potential Participant]

“Yes, a better time to call would be ____________________________”

[Screener]

Thank you, I will try to reach you then.

Good bye.

[Potential Participant]

“Yes, I have a few minutes”

Wonderful. Before I tell you about the study, I would like to ask you several questions to see if you would qualify to be a participant. This information will be kept confidential. If you feel uncomfortable answering one of these questions, you don’t have to give an answer. Some of the questions concern alcohol and drug abuse. Would you like to continue?

*Yes No*

*___ _*__ Are you between the ages of 18 and 65? [N=Exclude]

*___ _*__ Are you right-handed? [N=Exclude]

*___ _*__ Do you work a night shift (11 pm to 7 am)? [Y=Exclude]

*___ _*__ Can you walk? [N=Exclude]

*___ _*__ Do you have diabetes? [Y=Exclude]

*___ _*__ Do you currently have a problem with alcohol or non-prescription drugs?

[Screener: Do not record answer to this question. Instead simply make a decision to exclude if the answer is Yes, note in tally form the reason for the exclusion, and then destroy all personally identifying information related to this participant]

*___ _*__ Do you have a phobia of needles [Y=Exclude]

*___ _*__ Are you currently taking any prescribed psychotropic medications? [Y=Exclude]

(These include medications for anxiety, depression, or other psychological problems)

*___ _*__ Do you have any plans to start taking any prescribed psychotropic medications? [Y=Exclude]

*___ _*__ Do you take inhaled steroids for asthma? (e.g., Fluticasone) [Y = Exclude]

*___ _*__ Are you taking any corticosteroids? (Y / N) [Y=Exclude]

[Screener: Read some or all of list if needed]

 [Betamethasone](http://arthritis.about.com/od/betamethasone/) (Celestone)

 [Budesonide](http://arthritis.about.com/od/budesonide/) (Entocort EC)

 [Cortisone](http://arthritis.about.com/od/cortisone/) (Cortone)

 [Dexamethasone](http://arthritis.about.com/od/dexamethasone/) (Decadron)

 [Hydrocortisone](http://arthritis.about.com/od/hydrocortisone/) (Cortef)

 [Methylprednisolone](http://arthritis.about.com/od/methylprednisolone/) (Medrol)

 [Prednisolone](http://arthritis.about.com/od/prednisolone/) (Prelone)

 [Prednisone](http://arthritis.about.com/od/prednisone/) (Deltasone)

 [Triamcinolone](http://arthritis.about.com/od/triamcinolone/) (Kenacort, Kenalog)

*Yes No*

*___ _*__ Have you previously been trained in meditation?

*___ _*__ Have you ever been involved in a mindfulness class or mindfulness

practice?

[If yes] What was the class called? ___________________________

*___ _*__ Mindfulness Based Stress Reduction (MBSR)? [Y=Exclude]

*___ _*__ Do you currently meditate on a regular basis? [Y=Judgment]

*___ _*__ Have you meditated daily for at least the last 2 months? [Y=Exclude]

*___ _*__ In the last year, have you meditated at least weekly for two or more

months in a row?

Y=Judgment]

*___ _*__ Have you had daily practice with other mind-body techniques?

What techniques?

[Screener: Start with open-ended question. But if participant needs examples, give them. Also, after the open-ended response, please ask about the examples as well.]

*___ _*__ Daily yoga practice [Y=Exclude; previous exposure ok]

*___ _*__ Daily tai-chi [Y=Exclude]

*___ _*__ Other: ________________ [Y=Judgment]

[2 or more Y of the following are likely to need a stress test. Thus, 2 or more Y = Judgment]

*Yes No*

*___ _*__ Diagnosed hypertension?

*___ _*__ Hyperlipidemia

*___ _*__ High cholesterol

*___ _*__ Diabetes (Type I or II)

*___ _*__ Smoke cigarettes

*___ _*__ Family history of coronary or atherosclerotic disease (parents/siblings prior to age 55)

How many times per week do you engage in moderate sport and recreational activities such as walking, tennis doubles, ballroom dancing, weight training, etc. that lasts at least 20 minutes per occasion?

A) 1 time per week; B) 2 times per week; C) 3 times per week; D) >4 times per week.

[Answered D? Y=Exclude; If less than 1 time per week = may be inactive or sedentary which is 1 risk factor for needed a stress test before participating in exercise program]

How many times per week do you engage vigorous sport and recreational activities such as jogging, swimming, cycling, singles tennis, aerobic dance or other similar activities lasting at least 20 minutes per occasion?

A) 1 time per week; B) 2 times per week; C) > 3 times per week.

[Answered C? Y=Exclude; If less than 1 time per week = may be inactive or sedentary which is 1 risk factor for needed a stress test before participating in exercise program]

**MRI Screening Form**

________________________________________________________________________________________

# Sex: Female Male

# Age: ______ [18-65 ok]

# DOB: _____/_____/_____

Weight: _______

Height: ____”____’

[Obesity can be calculated from height and weight]

Hat Size: ______

Ethnicity: __________________

*Yes No*

*___ _*__ **Do you have corrected vision?**

*___ ___* **Have you ever had an MRI scan?**

When? ________________________________________________________________________________

Why? _________________________________________________________________________________

*___ ___* **Have you ever had surgery or a similar invasive procedure?**

When? ________________________________________________________________________________

Type? _________________________________________________________________________________

___ ___ **Have you ever had heart surgery?**

When? ________________________________________________________________________________

Type? _________________________________________________________________________________

___ ___ Do you have a Pacemaker? [Y= Exclude]

___ ___ Do you have an implanted cardiac defibrillator? [Y= Exclude]

___ ___ Do you have an artificial heart valve? [Y= Exclude]

___ ___ Do you have cardiac pace wires? [Y= Exclude]

___ ___ **Have you ever had head or brain surgery?**

When? ________________________________________________________________________________

Type? _________________________________________________________________________________

___ ___ **Have you ever had eye surgery?**

___ ___ Do you have lens implants? [Prior to 1983 = Exclude; 1984 or later = ok]

___ ___ **Do you wear dentures?**

**[If YES] Can you take them out? [Y=ok; N=Judgment]**

**[Note: Judgment means we must check with PI or MR Technician]**

___ ___ **Have you ever had ear surgery? [Tubes ok]**

___ ___ **Do you have a cochlear implant? [Y=Exclude]**

___ ___ **Do you wear a hearing aid?**

**[If YES] Can you take it out [Y=ok; N=Judgment]**

___ ___ **Have you ever had back surgery?**

When? ________________________________________________________________________________

Type? _________________________________________________________________________________

___ ___ **Do you have any implanted devices of any type?**

___ ___ Breast/Penile? [N=ok; Y=Judgment]

___ ___ Electrodes? [N=ok; Y=Judgment]

___ ___ Pumps (e.g., drug infusion device)? [N=ok; Y=Judgment]

___ ___ Bone or socket? [N=ok; Y=Judgment. Titanium ok but need Dr. note]

___ ___ Neurostimulators/Biostimulator? [N=ok; Y=Judgment] *Yes No*

___ ___ **Do you have any dental or orthodontic implants (fillings are O.K.)? [Metal bars in mouth = PI Judgment]**

Type? _________________________________________________________________________________

___ ___ **Do you have any type of prosthesis? [Y=ok if can take it off]**

Type? _________________________________________________________________________________

___ ___ **Do you have any type of orthopedic implant (e.g., pins, rods, screws, nails)? [Y= ok with Dr. note]**

Type? _________________________________________________________________________________

___ ___ **Do you have any permanent cosmetics (e.g., eyeliner)?**

**[If yes] What year did you get this done? __________ [Ok after 1975, else Exclude]**

___ ___ **Do you have any tattoos on your upper body?**

**[If yes] What year did you get this done? __________ [Ok after 1975, else Exclude]**

Where/Extent? __________________________________________________________________________

___ ___ **Do you have any body piercing(s)?**

**[If yes] Are they plastic? __________ [Y=ok]**

**[If no] Can you take the piercing(s) out? [Y=ok, N=Exclude]**

Where? ________________________________________________________________________________

___ ___ **Do you have a history of any metal in your body?**

___ ___ **Have you every worked as an occupational metal grinder? [Y = red flag for next few questions. Mostly we wantto know whether people have the potential for having metal in their eyes]**

Description: __________________________________________________________________________

___ ___ **Have you ever worked with metal as a hobby?**

Description: __________________________________________________________________________

___ ___ Did you routinely wear safety glasses? [Y=ok, N=Exclude]

___ ___ **Have you ever sought medical attention for metal in your eyes? [Y=Judgment: requires orbit x-ray]**

___ ___ **Have you ever had metal fragments removed from your eyes? [Y=Judgment: requires orbit x-ray]**

___ ___ **Have you ever been struck by a gun shot, B.B. or shrapnel?**

**[IF yes] Did it stick? [N=ok; Y=Judgment]**

___ ___ **Do you have any physical disabilities?**

Type? ________________________________________________________________________________

___ ___ **Do you have any involuntary motor disorders? [Point is: can they stay still for 90 mins in scanner]**

Type? ________________________________________________________________________________

___ ___ **Have you ever experienced claustrophobia?** [Y=Exclude]

When? _______________________________________________________________________________

___ ___ **Do you have any back problems that would prevent you from lying still for up to 2 hours? [Y=Exclude]**

**Female Subjects**

___ ___ **Are you or is there a chance you are pregnant? [Y=Exclude]**

___ ___ **Do you have an intrauterine device (IUD)? [Y=need Dr. note]**

****ANY QUESTIONABLE CONDITIONS MUST BE APPROVED BY THE MR TECHNICIANS**

**Ron Fisher or Michael Anderle 262-9230**

[Screener: Based on phone call to this point, can this person understand & speak English? [N=Exclude]

[Screener: IF participant DOES NOT MEET STUDY CRITERIA (i.e., 1 or more answers require exclusion)]

I am sorry but based on our exclusion criteria, you do not qualify for the study. We will destroy any information we currently have on you so you can be assured of confidentiality. Although you do not qualify to participate in our study, the UW Health Integrative Medicine Center offers similar classes that you can sign up for. Are you interested in receiving more information about these classes?.

[If yes] How would you like to be contacted?____________

(If by phone) What would be a good day and time? ______________

Thank you. You will be contacted soon with more information.

Thank you for your interest in our study. Goodbye.

[If no]. Okay. Thank you for your interest in our study. Goodbye.

NOTE TO SCREENER: Please document the reason(s) this person does not qualify for the study. Do NOT do this by tying it to them as a specific person. It is merely part of a tally so we can determine whether we need to change study criteria if recruitment of participants is too low. Please destroy any and all information about participant.

[Screener: IF 1 or more answers require JUDGMENT, they MAY not meet study criteria: Discussion with investigators is needed]

There is some question about whether you qualify for the study or not.

[Screener IF judgment classification is due to needing a stress test or any of the MRI exclusion criteria: It is possible that we will need you a note from your doctor in order for you to qualify for this study. If this is the case you will be sent the necessary forms in the mail within the week.

Would you mind if I discussed this with one of the other investigators and re-contacted you by phone or letter at a later time

[If yes] What would be a good day and time? ______________

Thank you, I will try to reach you then. We will contact you within the next two weeks with our decision

Good bye.

[If no] I am sorry but based on our exclusion criteria, you do not qualify for the study. We will destroy any information we currently have on you so you can be assured of confidentiality. I would like to wish you the best during the classes you decide to take at UW Health. Have a nice day.

Good Bye.

[Screener: Based on phone call to this point, can this person understand & speak English? [N=Exclude]

[Screener: IF participant DOES NOT MEET STUDY CRITERIA (i.e., 1 or more answers require exclusion)]

I am sorry but based on our exclusion criteria, you do not qualify for the study. We will destroy any information we currently have on you so you can be assured of confidentiality. I would like to wish you the best during the classes you decide to take at UW Health. Have a nice day.

Good Bye.

NOTE TO SCREENER: Please document the reason(s) this person does not qualify for the study. Do NOT do this by tying it to them as a specific person. It is merely part of a tally so we can determine whether we need to change study criteria if recruitment of participants is too low. Please destroy any and all information about participant.

[Screener: IF 1 or more answers require JUDGMENT, they MAY not meet study criteria: Discussion with investigators is needed]

There is some question about whether you qualify for the study or not. Would you mind if I discussed this with one of the other investigators and re-contacted you by phone or letter at a later time?

[If yes] What would be a good day and time? ______________

Met me also take down some information so we can contact you in the future.

Name_________________________

Email Address_______________________

Phone Number _______________________

Home Address _________________________

Thank you, we will contact you within the next two weeks

Good bye.

[If no] I am sorry but based on our exclusion criteria, you do not qualify for the study. We will destroy any information we currently have on you so you can be assured of confidentiality. I would like to wish you the best during the classes you decide to take at UW Health. Have a nice day.

Good Bye.

[Screener: IF participant MEETS STUDY CRITERIA]

I would now like to read a short description of the study. Please feel free to interrupt me to ask questions.

The University of Wisconsin, Laboratory for Affective Neuroscience, is conducting the study. The study examines the impact of Mindfulness-Based-Stress-Reduction (MBSR) and a Health-Enhancement Program (HEP) on pain regulation, social stress, basic affective and attention functions, and on the brain, immune, and endocrine mechanisms that subserve these processes.

Participating in this study will involve participating in one of these two eight-week classes designed to increase your health and well-being. You will be randomly assigned to one of these two classes for your first class. At the conclusion of the study, participants will be invited to participate in the second class should you be interested. The study will also involve having your brain scanned by functional magnetic resonance imaging (fMRI), performing a task involving identifying letters on a computer screen, answering a series of questionnaires, participating in a stressful social situation, and providing blood and saliva samples. One of the fMRI studies involves the infliction of safe and controlled amounts of pain. If you choose to participate, several painful heat sensations will be administered.

We will collect these measures at three time points: before your first class begins, immediately after your first class ends, and four months after your first class ends. You will then be offered the opportunity to take the second class but no measurements will be taken.

Participation in this study is voluntary.

The data collected in this study will not have your name or any personal information on it. Instead you will be given a participant identification number (PIN). All data obtained from you will be linked to that number. This number will be linked to your name on a data sheet that will be kept under lock and key.

Your participation includes completing 10 clinic visits and completing one of the two 8-week classes that we are researching. We will decide, based on chance (i.e., the flip of a coin) which class you will participate in. After the research study is completed, you are free to take the class that you did not complete during the research study, at your own expense. In this research study, you will be monetarily reimbursed for your participation after completing each phase of measurements and will receive a bonus for completing the final round of measures. Specifically, you will be paid at the rate of $25 per clinic visit, or $250 for ten visits total. This is approximately $14 per research hour. You will not be paid for your time attending the intervention program. You can withdraw from the research study at any time without penalty. In addition to the $250 for the 10 clinic visits, you will be paid a bonus of $225 for completing the entire program (attending all 10 clinic visits and all classes), a total of $475. After the study, you will be given information about the class you did not attend. The second class is optional; if you wish to join the second class, you must pay for it ($475) and you will not be paid for your time in the class since it is not part of the research study. The bonus is not contingent upon your participation in the second 8-week class.

Do you have any questions?

[Potential Participant]

Yes

[Screener]

Answer questions as well as possible

[Potential Participant]

No. I do not have questions.

[Screener] If you think you might be interested in participation in the study, we would like to schedule an initial meeting with you to explain the study and the classes in more detail. There we will review a consent form, choose your first class, have you complete some initial questionnaires, and schedule your next clinic visit. Are you interested in participating in this study?

[If no] We are interested in why some people may not want to be in the study. If you are comfortable, we would be interested in why you would prefer not to participate.

________________________________________________________________________

________________________________________________________________________

### [Check Schedule]

[If Yes] Great! Before scheduling you for the information meeting, the last thing we need to check is whether it is realistic for you to commit the time necessary for completing this study. This is very important so do you have access to your calendar right now?

[If no] Do you think you can give a realistic idea of what dates will work for you without it?

[if no] When would be a good time to call you back? ________________________

Thank you, I will try to reach you then.

Good-bye.

[Doesn’t want call back]. Ok. Thank you for your time. Good-bye.

[IF they have calendar or can commit without it, then continue]

Right now, we have a fair degree of flexibility with times and days, but later we will not. So please be as accurate and thoughtful as possible in indicating your availability. That way we can count on what you say.

Each clinic visit will last for about 2 hours, except the last one which will last 15 minutes. We need you to complete 3 visits at the first 2 time points and 4 visits during the 3^rd^ time point.10 total.

For the 1st time point, is it realistic for you to attend 3 clinic visits…

A1] Between August 2 and August 30? (Y / N)________________________________________________

B1] Between August 28 and September 26? (Y / N)_____________________________________________

For the 2nd time point, is it realistic for you to attend 3 clinic visits …

A2] Between October 19 and November 16? (Y / N)_____________________________________________

B2] Between November 14 and December 12? (Y / N)___________________________________________

For the 3rd time point, is it realistic for you to attend 4 clinic visits …

A3] Between February 15 and March 15, 2007? (Y / N)___________________________________________

B3] Between March 13 and April 10, 2007? (Y / N)_____________________________________________

You will also need to be able to attend one of the classes. I’m going to read off some dates. Please tell me which dates would work for you. For all dates the classes meet from 6 to 8:30 p.m.

*Yes No*

___ ___ Wednesday 8/30/2006

___ ___ Wednesday 9/6/2006

___ ___ Wednesday 9/13/2006

___ ___ Wednesday 9/20/2006

___ ___ Wednesday 9/27/2006

___ ___ Wednesday 10/4/2006

___ ___ Saturday 10/7/2006 (All day: 9am – 4 pm)

___ ___ Wednesday 10/11/2006

___ ___ Wednesday 10/18/2006

___ ___ Thursday 8/31/2006

___ ___ Thursday 9/7/2006

___ ___ Thursday 9/14/2006

___ ___ Thursday 9/21/2006

___ ___ Thursday 9/28/2006

___ ___ Thursday 10/5/2006

___ ___ Thursday 10/12/2006

___ ___ Thursday 10/19/2006

___ ___ Monday 9/25/2006

___ ___ Monday 10/2/2006

___ ___ Monday 10/9/2006

___ ___ Monday 10/16/2006

___ ___ Monday 10/23/2006

___ ___ Monday 10/30/2006

___ ___ Saturday 11/4/2006 (All day: 9am – 4 pm)

___ ___ Monday 11/6/2006

___ ___ Monday 11/13/2006

___ ___ Tuesday 9/26/2006

___ ___ Tuesday 10/3/2006

___ ___ Tuesday 10/10/2006

___ ___ Tuesday 10/17/2006

___ ___ Tuesday 10/24/2006

___ ___ Tuesday 10/31/2006

___ ___ Tuesday 11/7/2006

___ ___ Tuesday 11/14/2006

[If participant cannot make any class or they cannot commit to coming to clinical visits]

Going through the schedule, it does not look like it is realistic to be part of this study. Thank you for your time. Good-bye.

[Screener]

It seems like the study might work for you. Are you still interested in participating in this study?

[If no] We are interested in why some people may not want to be in the study. If you are comfortable, we would be interested in why you would prefer not to participate.

________________________________________________________________________

________________________________________________________________________

[If yes]

Wonderful. I am delighted that you are interested. I’d like to do two more things now. First, let me take down some information so we can contact you in the future.

Name_________________________

Email Address_______________________

Phone Number _______________________

Home Address _________________________

Best times:

Thank you. Now let’s schedule you to come in for an information meeting so we can tell you more about the study, answer any questions you might have, and start the study if you decide that is what you’d like to do.

[Potential Participant chooses a time]

Wonderful. So, we’ll plan on seeing you on ______________ at _________ a.m./p.m. at UW Health Integrative Medicine in room # _______ on the _______ floor. Integrative Medicine is located at 621 Science Drive (between Mineral Point and S. Whitney Way). Do you mind if we call or email you the night before your scheduled meeting to remind you?

[Potential Participant]

“Yes.”

Okay, we won’t call or email you to remind you. We’ll see you on _________ at ________ a.m./p.m.

[Potential Participant]

“No.”

Okay, we’ll send you a reminder email or give you a reminder phone call on __________.

In addition, we will send you an information packet containing directions for how to get to UW Health Integrative Medicine and information about the study. If you have any questions between now and the information session, please call (608) 262 – 9888. Thank you.

[Screener: If it becomes apparent that the potential subject is either not eligible or not interested, please keep a tally of the reason they are not eligible and destroy the information on that individual (contact information and any preliminary screening data).]

**Details on Classes for Answering Questions**

**Raised by Potential Participants**

**Both Classes**: Participants will meet in groups of up to 25 participants, for 2 ¼ – 2 ½ hour sessions, once per week for 8-weeks. An additional “all day” runs from 0900-1545, with the total duration of the sessions about 30 h per participant.

**Health Enhancement Program (HEP) Details**. The class will focus on four modules for improving well-being. Jude Sullivan, who specializes in the physical activity module, will guide each class with the assistance of an expert guest for the other three modules.

1. Physical Activity – Jude Sullivan

DESCRIPTION: Class participants will engage in consistent rhythmic movement that can be accomplished in a single session per day or aggregated over multiple sessions. The goal is to complete up to 30 minutes daily for 6 days per week. The focal activity is walking but can be any similar activity preferred by the participant. In addition, passive, static (non-moving) stretching will be incorporated to complete the 30 minute time commitment. The effort expended while engaging in the rhythmic activity is not to exceed what participants would self-judge to be between “fairly light” and “somewhat hard” (numeric = “11-13”) using the Borg Perceived Exertion Scale for physical activity.

1. Nutrition – Julie Thurlow, DrPH, RD, LD

DESCRIPTION: Optimal nutrition is not only the provision of nutrients to prevent deficiencies but also provision of amounts of nutrients and other dietary components/constituents to provide optimal health and decrease the risk of the development of chronic disease. Current best evidence indicates that the protective effects of food come from a dietary pattern that provides foods in appropriate types and amounts, and not supplements of specific nutrients and/or food components. Participants will learn what the current recommended dietary pattern is for their specific age, gender and activity as well as ways of identifying the strengths and weakness of their current diet and how to effect change if necessary.

1. Functional Movement – Jude Sullivan

DESCRIPTION: People of all ages and levels of physical ability are innately dependent on the ability to move. Motion occurs in the frontal, sagittal and transverse planes. Muscles are orchestrated to either evoke movement or maintain dynamic stability via concentric, eccentric and/or isometric contractions. Yet, skeletal muscle itself is incapable of controlling movement unless it is connected to bone. Bones, and their associated joints, act as lever systems with muscle forces acting as the counterbalance to an applied load. As we age, it is observed that lifestyle choices we make reduce our ability to take advantage of the infinite manner in which we can move and use our bodies. Functional movement training will help improve*:*

- Balance
- Core Stability
- Agility
- Mobility

Specifically, we address quality posture and alignment. Posture and alignment are the keys to creating optimal function. The functional interaction of each component in the kinetic chain allows for efficient and effective movement. The core (the entire circumferential area and within between the armpits and the groin) is an often neglected chain “link” that can dramatically contribute to improved health, athleticism, or sport performance. In addition, functional movement focuses on proprioception, defined as a sensory modality that includes sensation of joint movement and joint position sense. Balance is controlled by vision, the vestibular system and proprioception.

As a result, the goal of the overall training plan is to expand one’s movement repertoire in order to become more physically adaptable and “free.” This freedom opens doors to choose more activities to participate in as well as supply one with the necessary tools to be ready to adjust reactively as one needs (e.g. when tripping on a curb or slipping on a patch of ice). When training is introduced in an integrated manner, one quickly realizes that “different” forms of training overlap with one another (i.e. balance and core training). Ideally, they will blend together to make a complete training program. The following briefly describes the manner in which the program can be advanced:

- DECELERATE (slow down) before ACCELERATE (speed up); resist movement before assist movement
- SIMPLE movement patterns to COMPLEX movement patterns
- SLOW TO FAST (Do drills as fast as you CAN; NOT as fast as you CAN’T)
- RHYTHM first, then TEMPO; be coordinated and graceful before moving quickly
- Change the venue/modality = Changing the stimulus (e.g. WATER, SAND, HILLS, RESISTANCE CORDS adds intriguing problems to solve)
- Add REACTIONS and SKILLS (e.g. cables for advanced acceleration/deceleration, ball toss with a partner while squatting)

1. Music Therapy – Pamela Polcyn, MT-BC, WMTR

DESCRIPTION: The American Music Therapy Association defines music therapy as “an established healthcare profession that uses music to address physical, emotional, cognitive, and social needs of individuals of all ages. Music therapy improves the quality of life for persons who are well and meets the needs of children and adults with disabilities or illnesses. Music therapy interventions can be designed to promote wellness, manage stress, alleviate pain, express feelings, enhance memory, improve communication and promote physical rehabilitation. Music therapists assess emotional well-being, physical health, social functioning, communication abilities, and cognitive skills through musical responses; design music sessions for individuals and groups based on client needs using music improvisation, receptive music listening, song writing, lyric discussion, music and imagery, music performance, and learning through music; participate in interdisciplinary treatment planning, ongoing evaluation, and follow up”. ([www.musictherapy.org](http://www.musictherapy.org/)).

As a certified music therapist, I believe in the power of music and its ability to bring about change in the clients that I serve. I believe in music **as** therapy versus music **in** therapy. My belief is that the power for change lies in the music and the therapist serves as the instructor. Music is an effective use for therapy due to the way the body and the brain respond to the musical stimulus.

Music therapy can be used for stress reduction using active music making, music and relaxation with guided imagery, and songwriting. Exploring a variety of music interventions with class participants will maximize the class’ impact for a group with diverse musical and cultural backgrounds as well as physical and emotional needs. Exploring three different techniques will increase the probability that at least one of the interventions will help participants manage their stress. Initial homework assignments will be designed for class members to obtain a level of comfort with various types of music. The assignments will progress to the point where participants take an active role in their own music making.

**HEP All-Day Format:**

| 9:00-9:15 | Welcome, Introduction and Ground Rules |
| --- | --- |
|  |  |
| 9:15-9:45 | Guided Music Therapy Practice |
|  |  |
| 9:45-10:15 | Music Therapy Self-Practice |
|  |  |
| 10:15-10:45 | Group Discussion of Music Therapy Practice |
|  |  |
| 10:45-11:15 | Nutrition Review |
|  |  |
| 11:15-11:45 | Group Discussion of Nutrition Review |
|  |  |
| 11:45-12:45 | Lunch |
|  |  |
| 12:45-1:15 | Walking Activity |
|  |  |
| 1:15-1:45 | Stretch and Mobility Movement Activity |
|  |  |
| 1:45-2:15 | Group Discussion of Walking & Stretching Activity |
|  |  |
| 2:15-2:45 | Guided Functional Movement Practice |
|  |  |
| 2:45-3:15 | Functional Movement Self-Practice |
|  |  |
| 3:15-3:45 | Group Discussion of Functional Movement |
|  |  |
| 3:45-4:00 | Wrap Up of Days Events |

**Mindfulness Based Stress Reduction (MBSR) Details**. Two current MBSR instructors for the Mindfulness Center at Integrative Medicine will teach the MBSR class. They are Cindy McCallum and Ms. Katherine Bonus MA. Katherine Bonus is Manager and Instructor of Mindfulness Based Stress Reduction Program (MBSR) at UW Health Integrative Medicine.

Mindfulness meditation originates from a Buddhist tradition, but in its current formulation requires no particular religious orientation or belief system. It involves systematic training in the development of a sustained, non-aroused state of attention and clear awareness. Mindfulness is a meditation practice that cultivates paying attention in the present moment, bare of judgment, commentary, and decision.

- It trains the recognition of automatic reactivity such as habituated patterns of behavior, emotional reactivity, momentary distractions and loss of attention.
- It cultivates awareness, stability, insight and choice.
- Mindfulness includes working with how one relates to environmental stimuli, one’s sensitivity to bodily sensations, breathing, states of mind (thinking and emotions), and habitual patterns of automatic reactivity.
- MBSR training includes the cultivation of attitudes of self-appreciation, compassion and empathy.
- Audiotapes or compact discs of varying length but no longer than 45 min with guided instruction are initially provided that guide the participants in their formal meditation practice of the
  - body scan,
  - sitting meditation,
  - gentle yoga.
- Participants will be requested to practice for 6 days per week at home. Participants are asked to select a daily practice time best for them and to record on a log sheet what they practiced and how long.

Class will normally involve a review of previously assigned homework with discussion of difficulties participants may have experienced, introduction of new material, and hands-on practice/experience with new techniques or skills. The 8-week schedule is outlined below.

Weeks 1&2 - Body scan, 6 days/wk, 45 min/day. Sitting with awareness of breathing 10 min/day.

Weeks 3&4 - Alternate body scan with yoga, 6 days/wk, 45 min/day. Sitting with awareness of breathing 15-20 min/day.

Weeks 5&6 - Sitting exercises for 30-45 min/day. Begin walking meditation.

Week 7 - Practice 45 min/day, using any choice of methods. If using tapes, try not using them.

Week 8 - Go back to using tapes. Do body scan at least twice this week. Continue sitting and yoga. Provision of guided imagery tapes from Dr. Kabat-Zinn’s collection of 5 tapes/compact discs.

**MBSR All-Day Format:**

| 9:00 - 9:30 | (30 min) | Welcome Overview of Day & Intro. |
| --- | --- | --- |
| 9:30 – 10:30 | (1 hour) | Yoga |
| 10:30 – 10:40 | (15 min) | Sitting |
| 10:40 – 11:00 | (20 min) | Walking |
| 11:00 – 11:30 | (30 min) | Sitting |
| 11:30 – 12:00 | (30 min) | Walking |
| 12:00 – 1:00 | (1 hour) | Lunch and rest |
| 1:00 - 1:15 | (15 min) | Stretch, Walking in a circle |
| 1:15 – 2:00 | (45 min) | Mountain meditation or loving kindness forgiveness meditation |
| 2:00 – 3:00 | (1 hour) | Fast walking and Laughing meditation |
| 3:00 – 4:00 | (1 hour) | Wrap Up: Dialogue and Inquiry about participants’ first hand experience of the day. |

Reminder & Scheduling Call Runlog

***All reminder calls will be made the evening before a visit or session, unless otherwise specified.

Before Information Session

- Reminder of date and time of class
- Reminder to PLEASE bring calendar!
- Answer any questions that the participant may have

Before Visit 1

- Reminder of Visit #1 date, time, and content
  - If they will be in the scanner, remind them that they will need to lie still for an extended period of time and suggest that they might want to wear comfortable clothing
- Reminder to bring completed physician authorization form
- Reminder to bring completed medical history/physical activity history forms
- Reminder that they should plan to bring their salivary cortisol samples to one of the three visits
  - Reminder that cortisol samples MUST be refrigerated
- Confirm that they know how to get to the Waisman Center and offer directions if necessary

Before Visit 2

- Reminder of Visit #2 date, time, and content
  - If the will be in the scanner, remind them that they will need to lie still for an extended period of time and suggest that they might want to wear comfortable clothing
- If participant has not already turned in their completed physician authorization form, remind that they will need to bring it to Visit #2. If they come to visit #2 without the completed form the session will have to be rescheduled.
- If participant has not already turned in their cortisol samples, remind them that they should plan to bring their salivary cortisol samples to one of the three visits
  - Reminder that cortisol samples MUST be refrigerated
- Reminder to bring completed medical history/physical activity history forms, if they have not already been turned in

Before Visit #3

- Reminder of Visit #3 date, time, and content
  - If the will be in the scanner, remind them that they will need to lie still for an extended period of time and suggest that they might want to wear comfortable clothing
- Reminder to bring completed medical history/physical activity history forms, if they have not already been turned in
- If participant has not already turned in their cortisol samples, remind them that they should plan to bring their salivary cortisol samples to this visit, to save them from an extra trip to the Waisman Center
  - Reminder that cortisol samples MUST be refrigerated

After Visit #3 (prior to Class #1)

- This should be done as soon as the person is randomized. Do not wait until the evening before the class if possible.
- Telephone participants and tell them what class they are assigned to.
- Confirm that they intend to commit to coming to the scheduled class dates and times (including the all-day).
- Reminder of class #1 date and time
- Reminder to bring their completed Registration Form to Class #1
- Confirm they know how to get to the class.
- Send letter containing class schedule and directions

Before Class #2

- Reminder of date and time of class
- Answer any questions that the participant may have

Before Class #3

- Reminder of date and time of class
- Answer any questions that the participant may have

Before Class #4

- Reminder of date and time of class
- Answer any questions that the participant may have

Before Class #5

- Reminder of date and time of class
- Answer any questions that the participant may have

Before Class #6

- Reminder of date and time of class
- Answer any questions that the participant may have

Before Class #7

- Reminder of date and time of class
- Answer any questions that the participant may have

Before Class #8

- Remind participants that after class #8 there will be a blood draw and sign-up for visits 4-6
- Reminder to PLEASE bring calendar

Before Visit #4

- Reminder of Visit #4 date, time, and content
  - If the will be in the scanner, remind them that they will need to lie still for an extended period of time and suggest that they might want to wear comfortable clothing
- Reminder that they should plan to bring their salivary cortisol samples to one of the three visits
  - Reminder that cortisol samples MUST be refrigerated

Before Visit #5

- Reminder of Visit #5 date, time, and content
  - If the will be in the scanner, remind them that they will need to lie still for an extended period of time and suggest that they might want to wear comfortable clothing
- Reminder that they should plan to bring their salivary cortisol samples to one of the three visits
  - Reminder that cortisol samples MUST be refrigerated

Before Visit #6

- Reminder of Visit #6 date, time, and content
  - If the will be in the scanner, remind them that they will need to lie still for an extended period of time and suggest that they might want to wear comfortable clothing
- If participant has not already turned in their cortisol samples, remind them that they should plan to bring their salivary cortisol samples to this visit, to save them from an extra trip to the Waisman Center
  - Reminder that cortisol samples MUST be refrigerated

Before Visit #7

- Reminder of Visit #7 date, time, and content
  - If the will be in the scanner, remind them that they will need to lie still for an extended period of time and suggest that they might want to wear comfortable clothing
- Reminder that they should plan to bring their salivary cortisol samples to one of the three visits
  - Reminder that cortisol samples MUST be refrigerated

Before Visit #8

- Reminder of Visit #8 date, time, and content
  - If the will be in the scanner, remind them that they will need to lie still for an extended period of time and suggest that they might want to wear comfortable clothing
- Reminder that they should plan to bring their salivary cortisol samples to one of the three visits
  - Reminder that cortisol samples MUST be refrigerated

Before Visit #9

- Reminder of Visit #9 date, time, and content
  - If the will be in the scanner, remind them that they will need to lie still for an extended period of time and suggest that they might want to wear comfortable clothing
- Reminder that they should plan to bring their salivary cortisol samples to one of the three visits
  - Reminder that cortisol samples MUST be refrigerated

Before Visit #10

- Reminder of Visit #10 date , time, and content (Blood Draw #3)
- If participant has not already turned in their cortisol samples, remind them that they should plan to bring their salivary cortisol samples to this visit, to save them from an extra trip to the Waisman Center
  - Reminder that cortisol samples MUST be refrigerated

How to Make a Cortisol Kit

**MBSR Cortisol Kit Instructions**

Labels:

Currently the cortisol labels are in the directory: study/mrmri/Cortisol_study/MBSR_cortisol_labels

Jenna might change the location later.

There will be three days with five collection times each day. The days are already color coded. All that will have to be changed on these labels should be the subject number and the time number. When changes are made, try to be watchful, because it is really easy to miss a little mistake like printing a wrong subject number….

Printing Labels:

Labels have to be printed on address labels that have three labels in a row/across the page, not on the ones that have two per row.

Print the labels to: serrano\HP Color LaserJet 2500. It is in the second floor hallway.

Don’t use the PS format; sometimes things go wrong with that one. Don’t hit print just yet. Go in the second floor hallway, pull out the bottom paper drawer and place the labels face down in the tray. Then go back and hit print. You might have to go back to the printer once more and hit the flashing green button in order for it to finally print.

Putting the kit together:

Peel off the label and wrap it around a salivette tube, and try to wrap it around so that if something gets covered it is the last half of the label. This way the important information remains visible. When each day is done place them into a separate sandwich size Ziploc bags that are labeled the day and the time point. Make an extra tube with a blank label for each day and put one in each of the three bags. Then put all three sandwich bags into one large (gallon size) bag that is labeled with MBSR, the subject number, and the time point. Finally, Jenna will have some paperwork that will go right inside the big bag.

HEP-MBSR Cortisol Runlog

**HEP-MBSR Cortisol Run Log**

1. **Get the cortisol kit.** Ask the participant whether he/she has any question and whether the data collection went well. Write below if there is any feedback:

**_____________________________________________________________________________________________________________________________________________________________________________________________________________________________________________________________________________**

1. **Data storage: questionnaire** Store the questionnaire from the cortisol kit in the subject’s “forms” folder in the “lutz’ cabinet on the second floor.
2. **Data storage: cortisol**
    Store the cortisol kit in the freezer in the PET room (T-155, door can be unlocked using the B1234 key). The freezer is located in the back of the room along the left wall (the freezer door is silver). Store the Cortisol kits in the second shelf from the bottom.

*****Be sure to use the gloves when working with the freezer. It is kept at an extremely low temperature

(-80°F) and can be harmful to your skin if you do not use the gloves.

Pre Session Runlog:

**Pre Session Runlog** : #’s 1 – 5 **MUST** be completed **BEFORE** the participant arrives.

RA NAME: __________________ DATE: ____________________

1. Check your WiscCal Account to find the participant’s name, ID#, Session Time, & Visit #.

1. Get one set of keys from the “hiding place” on the second floor.
2. Go to the “Lutz” file cabinets on the second floor and find the file folder that corresponds to the correct subject ID and session type.
3. Record below the information from WiscCal below. This information is located on the front of the “Completed Forms” folder.

You can access your WiscCal account one of two ways.

Go to [http://wisccal.wisc.edu](http://wisccal.wisc.edu/) and log in using your NetID & password

Using any of the computers in the analysis bay, go to the Start Menu 🡪 All programs 🡪 Oracle Calendar 🡪 Oracle Calendar. Then sign-in using your NetID & password

ID#: _______________

Phase #: _______________

Visit #: ­­­­­­­­­­­­­­­­_______________

Session Time: _______________

1. Also check the box corresponding to the session type below (as indicated in WiscCal):

TSST

fMRI

Behavioral Only

Behavioral + Questionnaires

Mock Scan + Behavioral (Indicate below which order the tasks will occur in) \

___ 1. Mock Scan 2. Behavioral

___ 1. Behavioral 2. Mock Scan

1. Find the following information on the front of the “Completed Forms” folder. Fill in the information here and on the participants “Next Session Reminder” ½ sheet.

If this is the participant’s **last visit in phase #1** of data collection, there will be an envelope in the file folder containing class assignment information, location, date, & time of class, directions, etc. The participant cannot know this information until the **END** of the session. Also, you must be blind to this information, so if the participant asks, tell him/her that they will be given all of that information at the end of the session.

Task: _________________

Date: _________________

Time: _________________

1. On the front of the forms folder you will also see information about what the participant has/has not yet turned in. Please fill in below, under “**Previously Turned In**” what the participant had turned into BEFORE their session today. Under “**Turned In Today**”, check the “YES” line if the participant turned any of the materials in at the session TODAY.

Previously Turned In Turned in Today

YES NO YES

___ ___ ___ Newest Version of the Consent Form

___ ___ ___ Cortisol Kit

(You must refrigerate as soon as the participant is settled

into the experiment room)

___ ___ ___ Physical History / Medical History Form

___ ___ ___ Registration Form

___ ___ ___ Physician Authorization Form

** If a participant arrives for their fMRI without this form, inform the participant that it is of utmot important that the form be turned in ASAP. Without this form s/he will not be allowed to participate in the class

1. Follow the setup instructions on the session runlog(s).
2. Go to the waiting area to pick up the participant at the appropriate time.
3. Welcome the participant. Ask if he/she needs to use the bathroom before beginning. Also offer the participant water (you can get a glass and water from the 2^nd^ floor kitchen).
4. Ask the participant if s/he has brought with them any of the items listed in #7 that s/he has not yet turned in. (These forms will go into the participant’s manila folder labeled “forms” at the end of the session). Check the Yes” line under “Turned in Today” for anything that the participant turned in at the time of the clinic visit. Also make sure to put check marks next to items that have not yet been turned in on the participants “Your Next Clinic Visit” reminder sheet.

Notes: ______________________________________________________________________________

Post Session Runlog:

**Post Session Runlog:**

- - - 1. Ask participant to fill out the payment form that is in their manila folder.

*(It is important that we receive their Social Security #. If a participant asks why we need this, explain that we only use them in the case that we are audited to prove that we have paid actual participants).*

- - - 1. Ask the participant if s/he needs to use the restroom or a phone to contact their ride.
      2. IF the participant has more clinic visits during this phase of data collection give the participant the “Your Next Clinic Visit” Reminder Sheet. This should be filled out with the date and time of his/her next clinic visit and there should be check marks next to any item that s/he has not yet turned in. Also verbally remind the participant about what they need to bring to the next session

OR

IF this is the participant’s LAST clinic visit AND all materials have been turned in (paper work & Cortisol kit) during this phase of data collection, give the participant his/her “Class Assignment envelop which contains class assignment information, location, date, & time of class, & directions to UW Health Integrative Medicine where the class will be held. This envelope will be in the participant’s session folder when appropriate.

Script:

Experimenter says to the participant, “*This envelope contains your class assignment and additional information about the class. For research purposes I cannot know what class you have been assigned to, however, I would like you to open it here with me in case you have any questions that I can answer. Please keep in mind that it is essential that I remain blind to which class you are in.”*

🡪 Participant opens letter

Experimenter answers any questions that s/he can and then says to the participant, “*Now that you know what class you are in, I would like to ask if you are still committed to participating in the remaining portions of the study.*

- If participant says that they are no longer interested in participating

Experimenter says, “*Is it okay if one of the researchers call you to discuss your decision with you a bit further so that we can have a better understanding of why you have decided to drop-out of the study?*

🡪 If the participant says that they wish to stay in the study

Experimenter says, “*Great. Please remember that if you have any questions, feel free to contact the study coordinator at the number provided in the letter.*

- - - 1. Thank the participant for coming and guide them to the exit.
      2. Put any completed paperwork specific to that session (ie, session runlogs) back in the manila folder labeled for that particular session and return the folder to the file cabinet where it was found.
      3. Put any forms that were turned in at today’s session into the participant’s manila folder that is labeled “Forms”
      4. Place the completed payment form and the “Before Session” runlog sheet in Jenna’s mailbox in T-115 (above the fax machine).
      5. Make sure that you record complete details in the notes section of the runlog if the participant never showed up, the session had to be canceled, the participant decided that they no longer wish to be in the study, or any other oddity occurred.
      6. Lock all doors and cabinets.
      7. Put keys away!!!!! (DO NOT LEAVE WITH THE KEYS!!!)
      8. You’re all done! Great job! Thanks for your hard work!

## Behavioral Tasks Runlog

## Behavioral Tasks Runlog: Face-IAT & Line-CPT

Logsheet

Subject ID:____________ Session (circle one): 1=Aug/Sep; 2=Nov/Dec; 3=Feb/Mar, 2007

Date: ____________ Room #: _____ Experimenter(s):_________________________

Order of tasks:

- Mock Scan & then Behavioral Tasks (most are this order)
- Behavioral Tasks & then Mock Scan

Music Selection (circle one): 1 2 3 [Look this up in the black behavioral notebook]

Notes: _________________________________________________________________

_______________________________________________________________________

_______________________________________________________________________

_______________________________________________________________________

_______________________________________________________________________

_______________________________________________________________________

_______________________________________________________________________

_______________________________________________________________________

_______________________________________________________________________

_______________________________________________________________________

_______________________________________________________________________

_______________________________________________________________________

_______________________________________________________________________

_______________________________________________________________________

Setup room #266 or **268** (2^nd^ floor, Waisman side. 268 is preferred & will be normal room):

1. Go to study file drawer and take out Behavioral folder for each participant scheduled.
2. Go to cabinet #10 in the computer bay (right side): get laptop, power cable, and black Behavioral notebook
3. CONSENT FORM: Some participants need to sign a new consent form. If so, you can help speed this up by pointing out the few differences between the current one and the one they signed previously. See black Behavioral notebook for summary and a highlighted version of the consent that shows all the changes.
4. Tape “Do not Disturb” sign to door (it lives on the shelf inside the door)
5. Plug in and turn on computer (“Cowboy”)
6. Complete your portion of the logsheet (the one for each participant)
7. Set computer volume

- Make sure mute hardware button is NOT lit (button is located right under the name “Cowboy” on the just above the function keys on the keyboard)
- In the system tray/task bar at the bottom right of the computer screen, double-click on the little speaker icon
- For “Volume Control”, put slider at the 2^nd^ line
- Close volume window

1. Turn off room lights, turn on lamp by touching the metal. There are 3 levels of light (one per touch). We want the 2^nd^ level.
2. There’s no antivirus software active so please don’t check email on this “Cowboy”
3. Make sure external speakers are plugged into the headphone jack on the left side of the computer.
4. Set the volume on the external speakers as marked.
5. Look up the participant’s ID# on the Music Selection Log in the black Behavioral notebook & note the track number indicated. This is the track # you will play when the participant arrives. Also note that a few participants will get ONLY the CPT and not the IAT.
6. Open the “HEP-MBSR Music” folder on the desktop
7. Note participants name & take parking pass in hand and…

…Go get participant

1. Wait in the Keck lobby and watch for people to come in from the parking lot.
2. If they are late, call them
3. We may not be able to run someone who is more than 15 minutes late, especially if this is going to mean delaying participants who come after this person
4. Ask participants if they need to use the bathroom or get a drink before beginning tasks. Tell them this is their last chance to take a break for the next 70 minutes.

5 minutes of Music & Noticing

1. Note time: _______
2. After participants are seated in front and center of the table, Read:

**Thank you for participating in this research.**

**Please sit in your chair and take the next 5 minutes to notice the music or any physical sensations, emotions, or thoughts. You may close your eyes or keep them open as you prefer.**

1. Double-click on the correct track # in the “HEP-MBSR Music” folder on the desktop
2. Windows Media Player will start to play the music.
3. Turn the computer screen away from participants so the screen is facing the right wall.
4. The music will automatically end after 5 minutes.
5. Read:

**Now I’ll set up the computer tasks and get us started.**

1. Quit Windows Media Player, close the “HEP-MBSR Music” folder
2. Turn off speakers and remove audio plug from left side of the computer

Face IAT Task (~25 mins)

Steps for you & participants for Face IAT

1. Double-click on FaceIAT (v11).ebs [it’s on the desktop above the folders]
2. Press f7 (function key) to run [FYI: if you ever need to quit: ALT+Shift+ESC. Last resort, try CTRL+ESC]
3. Position participant on right side of table in front of computer.
4. Adjust seating / display for comfort. Try to ensure that monitor is eye-level and approximately 57cm away from eyes (participant can use pre-measured string).
5. Enter Subject Number WITHOUT A ZERO (e.g. ID # “1” NOT “01”) and press OK (or just hit Enter)
6. Enter the Session Number:
   1. 1=Aug/Sep,
   2. 2=Nov/Dec
   3. 3=Feb/Mar
7. Note start time:_____________
8. Read:

**"You will complete two different computer tasks. They will take about 70 minutes to complete. We’ll now start with the first task.**

**Please read the instructions on the screen and let me know when you’ve finished before continuing”**

1. Press “c” to continue
2. Show participant finger placement:
   1. Use 2 hands. First & 2^nd^ finger of left hand on 1 and 2 keys (negative end of the scale), first and 2^nd^ finger of right hand on 4 and 5 keys (positive end of the scale). They can use either hand for the 3 key (neutral).
   2. **“When you get there, you’ll use the 1^st^ and 2^nd^ fingers of your right hand on these keys”** [point out the “n” and “m“ keys labeled with a sticker “-“ and “+”]
3. Read: **“You will only have 6 seconds to respond. It’s pretty quick so be ready.”**
4. Make sure their hands are in place.
5. Hit “e” on the keyboard to continue the task
6. Participant will be guided through the entire task by the computer.
7. Make sure participants are hitting the right buttons (i.e., that they understand the task). If they aren’t, correct them asap
8. Here is the timing of the task:

- ~10 minutes in: There is a break in the middle of the face part of the task. Participants should stay seated and can continue when they wish to. The program will automatically begin after 1 minute, so you should warn them to get ready at about 50 seconds.
- 8 minutes more of faces
- Be ready to tell people to put their fingers on the numbered keys and respond quickly
- 7 minutes of feel, connected, similar questions

1. Note end time: ______________
2. Hit “e” to exit the program. This is important b/c only when you hit “e” will the data be saved. You should see the message “Script terminated successfully”. If you do not, check the box below and email Donal when you have time.

- Did NOT get “Script terminated successfully” message

1. Close out of window
2. Read: **Do you have any comments on this task?**

(Write their response verbatim with legible handwriting)

_________________________________________________________________________

_________________________________________________________________________

_________________________________________________________________________

1. **Aside from pictures of yourself, did you know anyone else from the pictures that you saw?** Yes No

If yes, **names and descriptions**:______________________________________

_________________________________________________________________________

_________________________________________________________________________

**How do you know them?** ______________________________________

#### Line CPT Task (~45 mins)

Steps for you & participants for Line CPT task:

1. Double-click on desktop short-cut: “Line-CPT.exp” [FYI: q = to quit during the task; p=pause; r=resume after pause]
2. Note: You will normally be brought to the “Main” tab in presentation. All instructions assume you start there.
3. Start first tutorial in Presentation: "scenario_tutorial_stable.sce" As with everything you will run in Presentation, start on the 'Main' tab, select the file to run in the selection box, and then click 'run scenario'. When you see the 'ready' screen, press "Enter" on the keyboard to launch.
4. Adjust seating / display for comfort. Try to ensure that monitor is eye-level and approximately 57cm away from eyes (use pre-measured string).
5. Note start time:____________
6. SIT NEAR PARTICIPANT and read:

**“We’re ready to start the second task.**

**First we’ll go over a short verbal description of the task, and then you’ll do a visual tutorial that will help to clarify the instructions. So, please pay attention to the description, but if anything is unclear, you’ll have an opportunity to ask questions during the tutorial.**

**In this task you will see single lines appear at the center of the screen one at a time. Whenever a line is not on the screen, you will see a pattern of dots. You can ignore the dots completely and focus on the lines when they appear. Throughout the task, you must keep your eyes on the red dot at the center of the screen.**

**The lines you see will be either long or short. When you are certain you have seen a short line, you should respond as quickly as possible with the left mouse button.** [Point out the left mouse button – they should use their right hand] **Whenever you see a long line, do not respond.**

**Now we’ll run through a short tutorial of what you will see during the task.**

1. Once you have read the preliminary instructions, begin the tutorial. Note: no subject # or session # for this b/c we are not recording data
2. Hit “enter” to start task.
3. Click mouse to continue
4. Sit next to subject as they work through tutorial - ask the following questions along the way:

- After long and short lines have been displayed (after 2 screens): "could you tell the difference between those lines?"
- When mask is on the screen: "do you see the red dot at the center of the screen?"
- After both trials have completed: "could you tell the difference between the lines?" (before the sound screens)

1. Click mouse button to end tutorial
2. Read: **“Do you have any other questions before we begin the practice trials?”**
3. Hit “Continue” and “Finish” at the Status window

## ## ## ## ##

1. Start the PRACTICE scenario PEST: "scenario_cpt_stable_practice_PEST.sce"
2. Type subject # followed by “_” [underscore] and then session # (1=Aug/Sep, 2=Nov/Dec, 3=Feb/Mar). For example, “99_1” is subject 99, session 1.
3. Click OK
4. At the 'ready' screen, read:

**“Now we’ll do a couple trials of practice. The first line you see will be a long line, and the second line will be a short line. After that, the order of long and short lines is random.**

**You’ll get sound feedback during this task: if a short line appears on the screen and you correctly respond to it, you will hear the “ding”; if you don’t respond to it, you’ll hear the “whoosh”; if a long line appears on the screen and you accidentally respond to it, you’ll also hear the “whoosh.”**

**Any questions?**

1. Hit “enter” to start task.
2. Participant will hit mouse button to begin
3. Task will simply end after a few trials and return you to the Status window
4. **Ask subject how it went & answer any questions they may have**
5. Click “Continue” and “Finish”

## ## ## ## ##

1. Start ACTUAL scenario PEST: "scenario_cpt_stable_PEST.sce"
2. Type subject # followed by “_” [underscore] and then session # (1=Aug/Sep, 2=Nov/Dec, 3=Feb/Mar). For example, “99_1” is subject 99, session 1
3. When screen shows 'ready', read PEST task instructions:

**“Now we’ll do the real thing. This time, the long line will never change in length, but the short line length will vary throughout the task. As your performance improves, the short line will get longer, and as your performance diminishes the short line will get shorter.**

**On average, you will see more long lines than short ones, but it is perfectly normal to see a few short lines in a row. Your job is always to respond as accurately and quickly as possible to the short line.**  **The first line you see will be the long line, and the second line will be the short line.**

**Do you have any questions?”**

1. Note start time: ______________

[Note: Most participants should complete this thresholding procedure (PEST) in 10-12 minutes. If someone gets to 15 minutes, you will have to stop the procedure (remember, hit “q”) and use a threshold of 101 for that participant. In this case, there’ll be no need to check the log file. Simply use 101 as threshold]

1. Hit “enter”
2. Participant clicks the mouse to start task
3. Move quietly back so that the participant is not distracted by you
4. Click 'continue' and 'finish' to close task
5. Note end time:______________
6. Hit “continue” and then “finish”
7. Open PEST log file for participant (located on the desktop\HEP-MBSR Line CPT\Logfiles\ ) Example: "PEST_cpt_stable_2_1” [example is for id #2, session 1]
8. Go to the end of the file (ctrl+end or scroll down) where you see “final threshold level (standard PEST): ”
9. Record the threshold number that appears here:_______. (e.g., 92)

## ## ## ## ##

1. Start practice task: "scenario_cpt_stable_practice.sce"
2. Input participant id (e.g., 99_1)
3. In next window enter threshold [“initial value for target height” (i.e., threshold value)]
4. When screen shows 'ready', read instructions:

**“You will now do the same task you just did, except both the short and long lines will never change length. Also, the short line will be very rare, and you will mostly see long lines. First we’ll do a minute of practice with sound feedback. Remember, the first line is long, and the second line is short.”**

1. Hit “enter”
2. Participant clicks mouse to start task
3. Note: These are practice trials. So, now is your chance to make sure they get the task.

- They should be responding ONLY to short lines. If they are responding too much, read: **“Remember, only respond to the short lines”.**
- They should be using their right hand on the left mouse button. Correct them if this is not the case.

1. When practice task completes, close task by clicking 'continue' and 'finish'
2. **Ask participant how it went & answer any questions**

## ## ## ## ##

1. Start actual task: "scenario_cpt_stable.sce"
2. Enter idnum_session (e.g., 99_1)
3. Enter threshold as previously
4. When screen shows 'ready', read instructions:

**“Now you will do a much longer version of the task without sound feedback. Remember that we’re interested in your natural ability to detect the short and long lines, so please don’t try to strategize. As in the other tasks, the first line you see will be long and the second line you see will be short, but after that the order of lines is random.** **Your job is to be as accurate and fast as possible in responding to the short lines, while not responding to the long lines.”**

1. Click 'Enter' button to start task
2. Note start time:______________ [task about ~24 mins]
3. Note: it’s important to be quiet from here to end of task
4. Participant clicks mouse to begin
5. MOVE CHAIR AWAY and please be as quiet as possible.
6. When real task completes, close task by clicking 'continue' and 'finish', and inform participant that they'll now get a short break
7. Note end time: ________________
8. **“Do you have any comments on that task? How was it for you?”**

(Write their response verbatim with legible handwriting)

_________________________________________________________________________

_________________________________________________________________________

_________________________________________________________________________

1. Note end time:_____________

After IAT & CPT Tasks

After behavioral tasks are done & participant STILL PRESENT:

1. If they have questions about what the task, remember they will do the task 2 more times. We will answer their questions at that point.
2. If participant has not already completed mock scan, refer to that protocol and proceed
3. If participant needs to complete computer questionnaires, refer to that protocol and proceed
4. See post session runlog
5. Thank them for participating and remind them of their next session if they have one

After all participants

1. Backup all data from each computer to the network drive.
   1. Start explorer. By doing so, DOIT will know you want access to wireless network & you will sign-in using your normal WISC ID and password.
   2. Double-click on “SecureFx” shortcut on desktop
   3. Highlight “HEPMBSR Data Backup tezpur.keck.waisman.wisc.edu”
   4. Enter your usual LAB/KECK user name and password as prompted
   5. Click “connect” button
   6. IAT Data Files
      1. Goto Desktop\HEP-MBSR FaceIAT
      2. You can sort by Date Modified
      3. There are 3 files for each participant: **FaceIAT (v11)-2-1.edat, FaceIAT (v11)-2-1.txt,** and **Subject_2_StimuliOrder.txt**. In these examples, the files are for participant idnum 2, session 1. Drag these files to the IAT folder in SecureFx window
      4. Make sure all 3 files are there for your participants
   7. Line CPT Data Files
      1. Goto Desktop\HEP-MBSR Line CPT\Logfiles
      2. Hint: You can sort by Date Modified
      3. There are 2 data files for each participant: **PEST_cpt_stable__2_1.txt** and **cpt_stable_data_2_1.txt**. In this example, the files are for participant idnum 2, session 1. Drag these files for each participant to the CPT folder in SecureFx window
      4. Make sure both files are there for your participants
   8. Exit SecureFx & close the connection
2. Turn off computer
3. Turn off lights, close & lock door.
4. Take down the “Do not Disturb” sign & put it back on the shelf inside of the door
5. File participant folders back in main file.
6. Put laptop back in cabinet #10

**If you cannot backup data, please email Donal with the participant #s that did not get backed up.**

Any questions, just email me (Donal) at [dgmaccoon@wisc.edu](mailto:dgmaccoon@students.wisc.edu). 263-1968 (office) or 240-9950 (home). Thanks!

Heat Stimulation & Emotion Regulation Simulation Session Runlog

## HEP-MBSR Heat Stimulation and Emotion Regulation

## Simulation Session Run Log

**Simulation Scan (___/___/___)**

**1. SIMULATION ROOM Preparation (T124)**

**1) Subject folder**

 Check **WiscCal** to see which order Behavioral Tasks and Mock Scan will occur in. Check to see if there is **another mock scan** or **fMRI scan immediately after** your session. If there is, move the pain equipment accordingly after your session.

 Get subject’s Mock+Behavioral folder from the “LUTZ” cabinet on second floor. Complete

**Pre-Session Run Log**. If necessary to save time, have subject read over and sign new consent form

at home and bring to next visit.

 Post “HEP-MBSR” greeting (located in copy room behind door) by parking lot entrance if you have a session after 6pm. Or print out at Y:\MBSR\fmri\runlog\Welcome_sheet

**2) Mock scanner**

 Place “Do Not Disturb” sign outside door

 Place soft pillow with a **clean cover** (in cabinet above sink) on the head coil, with higher end of pillow on top

 **Login** to E-prime computer (computer on the left). If someone is already logged in, LOG THEM OUT AND LOG YOURSELF IN. This is critical.

 Check headphones **volume** (on Taskbar, click on speaker, adjust “Volume Control” [should be between second and third bar from the bottom]. Windows Media Player volume should be on max)

 Turn on Avotec **goggles** (flip switch on cart to the lower right of mock scanner)

 Turn on mock scanner **back light** (plug into outlet)

**3) Thermal stimulator**

 Get thermal stimulator and box from **Cabinet 9** in computer bay

 Move cabinet next to mock scanner. Set up **thermal stimulator** on cabinet, thermode side facing the mock scanner bed. **Plug thermode** in to the machine.

 Set up heat stimulation **laptop** on desk, near E-prime computer. Make sure it is **plugged in** and turn it on (push button on left).

 Hook up **cables** from E-prime **computer** 🡪 **laptop** and **laptop** 🡪 **stimulator** by matching color labels.

 Enable laptop to receive signals from the e-prime computer: Go to “**Settings**” on start menu 🡪 “**Control Panel**” 🡪 “**Accessibility Options**” 🡪 “**General**” tab 🡪 **UNCLICK “Support Serial Key Devices”** 🡪 then **RECLICK** 🡪 “**Apply**” 🡪 “**OK**”

 **Serial test**: Make sure **laptop and PC are communicating** (always do this after any computer abnormality)

___Open “**GO MBSR TEAM” word file** on desktop of laptop.

___On E-prime computer: Go to desktop folder “HEPMBSR”, open “**testSerial.ebs**” (running

man icon), press **F7**. Enter **any subject #** (does not matter for serial test), any session #.

___Make sure when you hit space bar on E-prime computer, it moves a space on the laptop.

___Laptop: close word file. E-prime computer: close program by hitting **Control + Alt + Shift**

 **Turn on** thermal stimulator

 Open “**shortcut to pain**” on laptop. Wait for system initialization to complete. Click “**test**” and “**ramp up**”. Press the **space bar** until “Waiting for a trigger” appears on the screen.

 Check **water levels** on stimulator (Should be between minimum and maximum). If not enough, use **screwdriver** to open, insert **distilled water** with **syringe** (all located in box). (If need to replace water,

go to first sink on the right in T155 [open with key B1234]. Use right tap labeled “DW” (only distilled water can be used in the machine). Syringes located in drawers in T155)

**4) E-Prime Computer**

 **Login to** **E-prime** computer

 **Configure computer to run the button box**

___Go to Start 🡪 settings 🡪 control panel 🡪 system 🡪 device manager

___Select “+” next to "**NetMos multifunction**" device. Click once on "NetMos PCL..."

___Click "**remove**" at the bottom.

___Select Yes to restart the computer (only the e-prime computer)

___Login again. It will ask about installing the device. Keep hitting **ENTER** until it is installed.

___When you are logged in, go to Start 🡪 settings 🡪 control panel 🡪 system 🡪 device

manager

___Select “+” next to "**Ports (COM + LPT**)”

___Double-click on “**COM1**”

___On "port settings" tab, click the "Advanced" button

___**Unclick "Fifo Buffers**", press OK. Press OK on other windows.

 Set up **cat picture** (desktop folder “align_EBS”, **align** file, **F7**, Ctrl + Alt + Shift to quit)

**2. WAITING ROOM**

 Greet and escort subject to simulation room (T124)

 Invite subject to use the restroom or have a drink of water.

 Collect paperwork for pre-session runlog

**3. SIMULATION ROOM (T124)**

**1) Orient subject to mock scanner**

 Explain purpose of session: to acclimate to mock scanner, practice heat stimulation and emotion regulation tasks

*I want to now give you an overview of what will take place during this session. This is the mock scanner* (scanner)*. It is not magnetic, so you do not have to worry about metal objects today. This is the head coil* (head coil)*. These are the goggles through which you will be seeing the stimuli for the experiments* (goggles)*.*

*You will be positioned on the table with a special pillow and neck brace around your neck designed to help you reduce head movement. You will wear headphones through which you will hear instructions for the tasks and what the scanner will sound like* (headphones)*. Once you are positioned and the ear phones are in, you will be positioned inside the scanner* (bed)*.*

 Provide overview of the heat stimulation and emotion regulation tasks.

*You will be practicing two different tasks today. While you are in the practice scanner today, you will be viewing pictures and hearing descriptions similar to those on the day of the actual scan. You will be doing a heat stimulation task and an emotion regulation task. The heat stimulation task involves having a warm stimulus applied to your wrist, and you will be asked to either notice your sensations, emotions, and thoughts or to notice the music. The emotion regulation task involves viewing pictures and listening*

*to descriptions about the pictures. We will first begin with finding the right temperature for you in the heat stimulation task.*

**2) Find subject’s heat stimulation threshold**

 Sit participant in front of **laptop.**

*We will now going to do some* ***threshold testing*** *to make sure you’ll be comfortable in the experiment. I will fit this thermode to your wrist, and this is where the heat stimulation will come from. I also want to let you know that the heat we are using is* ***completely safe*** *and* ***cannot burn you.***

 Fit heat thermode on **left wrist** (make sure it is comfortable). Have Pp hold cord with thumb and

forefinger.

 Explain procedure (see instructions below).

# INSTRUCTIONS

*When the program starts, you will feel heat begin to rise. When the temperature has reached what you think is an* ***8 out of 10,*** *where 0 is no pain and* ***10 is the most pain tolerable****, press the*

***space bar*** (make sure they understand that it is most pain TOLERABLE)*. After you hit the space bar, the temperature will return to baseline, and the procedure will begin again in about 30s. This will happen 10 times.*

*Is this clear?*

*Here is a piece of scrap paper* [give subject post-it and pen] *where you should make a tick mark after each time you hit the space bar. You will be finished after the 10^th^ time, and we ask you to make tick marks so that we know when you are done.*

*If you ever feel the need to stop for any reason, you may hit the S key* [point out S key]*. An emergency has never happened, and this is for your peace of mind. Also, if you need to remove the thermode from your wrist, you can just lift the thermode (-SHOW THEM-). Only the side touching your wrist will be hot.*

*Do you have any questions? Are you ready to begin? When you are ready, you may press the* ***space bar.***

 Press “**function”** and **“F5**” at the **same time** to blank out the screen. Subject presses **space bar** to begin ramp up procedure.

 When finished, help subject **take off thermode** and have subject sit on scanner bed (so can’t see temperatures on laptop).

*You may sit and rest on the scanner bed while I set up. You may also* ***read the directions*** *to the heat stimulation and emotion regulation tasks, and I will go over them with you afterwards to make sure you understand.*

 **Give subject Instructions sheet** (rip off **back page of run log**). Give subject button box so can see

what the instructions are explaining.

 Answer any questions about directions that the subject may have.

 After 10 runs, press “function” and “F5” simultaneously again to bring back screen.

 Record the 10 temperatures.

| 1._____ 2._____ 3._____ 4._____ 5._____ | **Average**  **6._____ 7._____ 8._____ 9._____ 10._____** |
| --- | --- |

 Determine average temperature of heat stimuli (based on **average of final 5 [round off** to get a whole number]

##### Average temperature:_________.0°

 Determine **final temperature** of heat stimuli (**average temp - 1°**. Use calculator on computer if

needed.)

| **Final temperature (average- 1°**)**:______________.0°** |
| --- |

 If final temperature is **below 45°**, make sure they understood the original directions. If understood

directions, see if subject can tolerate a couple trials of **HEP_MBSR_45** on laptop.

*Unfortunately, your temperature is below the threshold we would like to keep for the study.*

*-Let me make sure you* ***understood the original directions****… You rated your pain an 8 out of 10, where 10 is the MOST PAIN TOLERABLE, correct?*

***-THEN-***

*-If you agree, since your temperature was below the threshold, we can* ***try a few trials of the minimum***

***temperature****. If it is tolerable, we may continue with the study. If you cannot tolerate this temperature,*

*then that it is fine, but you cannot participate in the fMRI portion of the study.*

| If subject cannot tolerate **at least 45°, SUBJECT IS EXLUDED** FROM THE STUDY!!!!! |
| --- |

 **Laptop**: Hit “**S**” and “**Back**” on current screen (Keep choosing “yes”). **Do not save data**. THIS IS TO PREVENT ACCIDENTAL TRIGGERING OF THE THERMODE!!!!!

**3) Acclimate subject to scanner**

 Explain importance of comfort to minimize movement

*Before we have you lie down on the scanner bed, it is important for to know that you should feel as* ***comfortable*** *as possible while in the scanner. On the day of the actual scan, feel free to tell the MRI*

*technician if you are uncomfortable at all while he/she is setting you up. It is extremely important that you are comfortable because the more comfortable you are, the less you will move. It is important to have as little movement as possible because it makes the images of your brain less blurry and easier to analyze. If you move your head too much, your data will not be usable. To help you remain still, we will fit your head with a neck brace and a soft pillow.*

 Prepare **neckbrace + soft pillow**. Help subject put on neck brace. Make sure it is not backwards or upside down. (If subject is wearing ponytail or anything that can make the head uncomfortable, have them remove it)

 Ask subject to lie down, with their shoulders nearly touching the base of the head coil. Make sure the neck brace is comfortable with the pillow.

*Please lie down with your head on the pillow and your shoulders almost touching where the pillow is lying*. [Have subject use stool to help them get on the scanner bed] *Is that comfortable?*

 Prepare **vacuum pillow** if neck brace is **uncomfortable** or **goggles cannot fit** over subject’s head.

Place **small pad** on head coil. Smooth out balls in vacuum pillow. Deflate the VacPillow **slightly** using the VAC tap (the tube is in the cupboard above the sink area) and **clamp shut**. Keeping **tube attached** and **vacuum on**, place VacPillow with a clean cover on the headcoil. Make sure to position the valve on the right (i.e. kitchen-sink) side of the headcoil. Put **headphones** on subject. Wrap VacPillow around the head of the subject tightly, make sure that the valve is on the correct side of the subject’s head, and on the outside of the pillow. Make sure that the cover is not obstructing the subject’s eyes. **Unclamp** vacuum pillow, deflate the pillow until it fits the subject well. (Be careful with the valve, it can rip easily!) **Clamp**

when deflated completely, remove tube. (In the case the pillow is being used in the scanner room, place head/neck padding with clean cover on head coil instead.)

*Since the neck brace was uncomfortable/we need to lower your head, we are going to fit the vacuum pillow around your head to keep your head more stable and to help you feel more comfortable. How does that feel?*

 Explain and put **headphones** on subject

*Please put on these headphones so you can hear the instructions.* [Subject puts on headphones] *Are you comfortable?*

 Put heat **thermode** back on **left** wrist.

*Now I am putting the thermode back on your wrist so we can use it to practice the heat stimulation task. Is this too tight?*

 Explain **button box**. Position under right hand. **Pinky finger** on the **right** **goes on the red button.** Pointer finger moves cursor left. Pinky finger moves cursor right. Middle two fingers to select.

*Here is the button box. You place your four right fingers on the four buttons* [show subject the box and position right hand]*. You will have to make a few ratings with the button box during the experiment, which I will explain further later. To move the cursor to the left, press the button with your pointer finger on the left. To move the cursor right, press the button with your pinky finger on the right. To select your*

*response, press with any of your two middle fingers.* [lightly touch fingers so that subject can feel which fingers to use]

 Turn on **fluorescent light** on top of file cabinet. Turn off room light. (To make the screen more clear)

*I am turning off the lights so you can see the screen in the goggles more clearly.*

 Position the **head coil and goggles** over the subject’s head

*Now I am going to pull the head coil over your head so you can see the stimuli through the goggles.*

____Pull head coil slowly over head. Make sure the goggles are high enough so that they do not hit

subject’s face.

____Eyeball proper placement of each goggle over each eye

*Now close your right eye and look with your left eye. Is any part of the screen cut off?* [adjust

accordingly until they can see most of the screen] *Is it in focus?* [adjust grey focus panel until it is clear]

*Now close your left eye and look with the right eye. Is any part of the screen cut off?* Follow same

procedure.

____Adjust the 3 planes of movement for the goggles for each eye. Ask subject to close right eye and look with left eye. Adjust directions if any part of the screen is cut off. Adjust **focus**. Ask subject to close left eye and look with right eye. Adjust directions if any part of the screen is cut off. Adjust **focus**. (Avoid cranking at maximum!)

____Make sure subject can clearly see **one image** (instead of two). If not, move goggles closer to the

eyes or adjust in horizontal plane.

____If having difficulty, ask subject to do task using **only one eye** (other eye closed). **Save time!**

 **Position subject into bore**

*Now I am going to slowly move you into the scanner. Let me know if you are uncomfortable at any time.*

*How are you feeling?* Adjust wires if necessary.

____Slowly push subject into bore. (Make sure wires don’t get tangled). Stop when edge of bed hits

the tape mark.

____ Inquire about subject’s well-being.

 Turn on room light if needed.

**4) Play scanner sounds**

*Now I am going to play some sounds you will hear in the scanner on the day of the actual scan. The*

*sound will be very loud the day of the scan, but you will be wearing ear plugs.*

 **E-prime computer:** Take down cat picture by pressing **Control + Alt + Shift, OK, OK**

 Open “**3.T sound**” folder on desktop. Play about 30 s of “**spgr**.wav” and “**epi**.wav”. Volume should be at maximum in Windows Media Player window. Make sure volume is adequate for subject to hear. If not, increase volume in Volume Control.

**5) Set up E-prime computer and Laptop for experiment**

 **E-prime**: Open “**HEPMBSR**” folder on desktop. Open **“MBSR_Pain_Sim.ebs**” file, press **F7**, enter **Subject ID** and **Session #(1)**.

 **Check temperature** from Page 3 of Run Log. **Temperature:_______**

 **Laptop**: Choose panel that says **HEP_MBSR_(correct temperature)**. **Press spacebar**.

**6) Reminder of Heat Stimulation task**

 Read reminder (below)

***Reminder***

*Let me ask a couple of questions to make sure you understood the instructions:*

*-What will you be focusing your attention on when the* ***cross*** *is on the screen? (*Experimenter*: “That’s right/wrong, you will be focusing on* ***your sensations, emotions and thoughts****”)*

*What will you be focusing your attention on when the letter “****M****” is on the screen? (*Experimenter*: “That’s right/wrong, you will* ***be focusing on the music****”)*

*You will be asked* ***to rate the pain*** *on two rating screens: the first for* ***intensity*** *and the second for* ***unpleasantness****. The intensity scale measures how* ***hot*** *the pain is, and the unpleasantness scale measures how much the* ***pain bothers you****. You will see a screen numbered* ***0-10*** *with an arrow underneath. You can* ***move the arrow using the two outer buttons*** *on the button box and once the arrow is below the number you would like to select you can press either of* ***the two inner buttons to make your choice****.*

*Please remember to keep your* ***eyes open*** *throughout the experiment and to* ***maintain your gaze*** *on the letter or cross at the center of the screen. It is very important that you don’t move during the experiment, so please remain extremely still. If you need to stop the experiment, please let me know.*

*Are you ready to start the heat stimulation task?*

**7) Practice Heat Stimulation task**

 E-prime computer should say “Wait for ttl”. Laptop should say “Waiting for a trigger”. **E-prime computer:** Now press the **spacebar** and click down the **scroll bar** (middle button) of mouse. This will begin the task.

 Make sure the **heat was triggered** on the laptop screen.

 Make sure subject is making **ratings correctly**. If not, check that fingers positioned correctly and that they understand how to use the button box.

 Monitor and Log Ratings:

1. I:___ U:___ 2. I:___ U:___ 3. I:___ U:___ 4. I:___ U:___

 Give pointers on ratings if necessary. “Now you know how fast the ratings you will go.” “Make sure to use one of the outer buttons first before choosing your selection with the inner buttons.” If you notice that the subject’s **ratings were not high enough (no rating of 6 or above for heat trials)**, ask

*I noticed that you did not rate the heat very highly. Did you feel 2 heat stimuli that were hotter than the other 2?* [to make sure the heat was triggering properly]. *It seems like the hotter heat did not bother you too much. Is this true?* [If yes], *Since we want the heat to be hot enough to cause you a little*

*discomfort, is it ok if we try the heat* ***one degree higher?*** [If no], *I want to make sure you understood the ratings directions…etc.*

**If subject says yes**, click the program on laptop that is HEP_MBSR_(one degree higher) and have it run through a couple of trials. Ask the participant how much they would have rated those trials (remember one trial is warm, the other is hot. Ask after the hot trial.) **NEW TEMPERATURE:________________**

**If subject says no**, make sure they understood the ratings directions. Try a few more trials of their current temperature and have them rate the heat properly.

 After task, inquire about comfort and if the **heat was tolerable**.

*How was that?  Were you able to hear the music and instructions? Will you be able to tolerate a similar but longer experiment in the scanner?*

Circle one: YES NO

If pain not tolerable, hit “Back” on laptop and try a couple of trials with one degree lower, etc. until the subject feels comfortable. **NEW TEMPERATURE: _______**

| If subject cannot tolerate **at least 45°, SUBJECT IS EXLUDED** FROM THE STUDY!!!!! |
| --- |

 Hit “**S**” and “**Exit” out of laptop program** to PREVENT ACCIDENTAL HEAT TRIGGERING

**Heat Stimulation Task Notes______________________________________________________________**

**_______________________________________________________________________________________**

**_______________________________________________________________________________________**

**_______________________________________________________________________________________**

**8) Explain Emotion Regulation task**

 Inquire about subject’s well-being.

*How are you feeling after the heat stimulation task? You may now* ***let go of the button box.*** *We will not need it for the next task. Are you ready for the emotion regulation task?*

 Remind of task (See below)

***INSTRUCTIONS***

*To remind you, in the emotion regulation task, you will be viewing pictures and listening to descriptions about the pictures. Please listen to the descriptions carefully and view the picture for the entire time it is presented. One type of description you will hear will say that the* ***picture is a scene from a movie*** *and that the people are actors or actresses. Another type of description you will hear will ask you to imagine the person or people in the scene are* ***loved ones****. Imagine that whatever is happening in the scene is happening to him or her. Try to* ***really envision or imagine your loved one in the scene depicted.***

*Before we begin, please think of a few loved ones who are* ***male, female, and children****. If you see a picture of a male, envision one of your male loved ones. If you see a picture of a child, imagine one of your child loved ones. If you see a picture of a woman, imagine one of your female loved ones. If you see a group of people, imagine a group of loved ones. Remember to*

*really envision your loved one in the scene depicted. Can you* ***tell me the relationship*** *you have with some of the loved ones you will use in the task?*

 Confirm and record some loved ones subject will use (e.g. best female friend, father, nephew)

______________________________________________________________________________

*Some of the pictures will be very disturbing and some will be neutral. If you are ever uncomfortable with the pictures, please let us know, and we will stop the study.*

*Again, you should try your best to look at the picture for the entire time it is presented. You will see a fixation cross after each picture. When you see the fixation cross, please clear your mind of the image you just saw and the state of mind you were in. Do you have any questions?*

**9) E-Prime computer: Practice Emotion Regulation task**

 **Click mouse** to end heat stimulation task. Close heat stim e-prime windows.

 Open Emotion Regulation E-prime run file (Desktop: HEPMBSR/**HEPMBSR_ER_Practice.ebs**). Hit **F7** to run file.

 Enter **Subject ID** and **Session #** 1

 **Click mouse** when you see “Get Ready!” screen to begin

 (Begin **heat stimulation setup cleanup** if you have time) Always try to save time!

** Click mouse** when you see “Please Remain Still!” This ends the task.

 After task, make sure the subject can tolerate the disturbing nature of the pictures

 Today only practice 6 trials. Real scan is much longer – 51 trials

[After the task] *How are you feeling?* *Were you able to hear the descriptions*? *Were you able to handle the disturbing pictures? Today you only saw 6 pictures, but on the day of the scan you will view 51 pictures. Do you think you will be able to do this? Were you really able to think that some scenes were from a movie and in other scenes imagine that the situation was happening to a loved one? Do you have any questions?*

**Emotion Regulation Task Notes_____________________________________________________________**

**_______________________________________________________________________________**

**_______________________________________________________________________________**

**_______________________________________________________________________________**

**10) Closings**

 **Pull subject out** of scanner bore.

 Move goggles further from face. Carefully pull back head coil.

 Help subject remove heat thermode, headphones and button box

 Ask if subject has any questions or concerns

 Subject is still interested in the study?

*Do you have any questions or concerns about the study? Are you still interested in participating in the study? Great, we will see you on the day of the fMRI scan. Have a good day/evening.*

 Complete any **post-session run log procedures** needed for the subject if they are done with this visit (give reminder notice, fill out payment form). Show subject the way out of the building.

**11) Data management**

Heat Stimulation Data

 E-prime computer: In desktop “HEPMBSR” folder, drag MBSR_pain_sim-###-1**.txt** and **.edat files** to “Pain_Practice_Data”

 Desktop “HEPMBSR” folder: Delete testSerial##.txt file

 **Record final temperature,** if subject uses **vacuum pillow,** any **abnormalities** in the session, and your **initials** in Desktop**: “SimSession_Notes” Very Important!!!!** (Make sure **temperature** in SimSession_Notes **matches** with what is written in the run log!)

Emotion Regulation Data

 Desktop “HEPMBSR” folder: drag HEPMBSR_ER_Practice-###-1**.edat** and **.txt** files to “**ER_Practice_Data**” folder

fMRI folder

 In “**fMRI Scan Run Log**,” enter the heat stimulation task **final temperature** on first line of the run

log. Indicate whether **vacuum pillow** was used.

**12) Cleanup**

 Log out of E-prime computer. Shut down laptop.

 Move back cabinet. Put away button box and headphones

 Throw away pillow cover

 Turn off Avotec goggles, scanner back light, fluorescent light

 Pack cables, thermode and laptop CAREFULLY back into box. (wrap up cables, put thermode on top) Bring pain stimulator and box to computer bay (Cabinet 9) or scanner room if needed for fMRI scan.

 Bring in “Do Not Disturb” sign

 Put back “HEP-MBSR” welcome sign in copy room if no other subjects are arriving. Do not forget!

 Put subject file in cabinet

**13) Behavioral Tasks or Post-Session Run Log**

 Go on to behavioral tasks protocol if necessary

 Complete **Post-Session Run Log**

#### Important Contact Information

General issues: **Antoine Lutz** (heat stim task), office: (26)2-8705, cell: 608-335-9845, room T-233, alutz@wisc.edu

**Helen Weng** (emot reg task), office: (26)3-0269, cell: 917-543-7292, room S117-D, hweng@wisc.edu

Donal MacCoon (behavioral task), office: (26)3-1968, room A-132, dgmaccoon@wisc.edu

Jenna Sheftel (scheduling), cell: 847-602-0482, mbsrstudy@gmail.com

Pain equipment/software issues: **Tim Salomons**, office: (26)3-1968, room A-123, tvsalomons@wisc.edu

Mock scanner issues: **Michael Anderle,** room T121, office: (26)2-9230, manderle@wisc.edu

Ron Fisher, Room T121, office: (26)2-9230, ronaldfisher@wisc.edu

Computer/server issues: Adrian Pederson, Room T125, (26)5-6608, [apederson@wisc.edu](mailto:apederson@wisc.edu)

**SIMULATION SESSION NOTES (**note in “SimSession_Notes” on desktop)_______**___________________**

HEP-MBSR fMRI Study

Instructions Sheet

Heat Stimulation Task

***INSTRUCTIONS***

*In the heat stimulation task, you will receive cues and instructions telling you where to focus your attention. You will also receive several bursts of heat, some of which may be painful. On some trials you will be instructed to* ***notice sensations, emotions and thoughts****. This will be accompanied by a* ***cross*** *that will appear on the screen. We will ask you to continue to pay attention to your sensations, emotions and thoughts as best you can for the entire time the cross is on the screen, including* ***during the bursts of heat****. On other trials you will be instructed to* ***notice the music****. This will be accompanied by the letter “****M****” which will appear on the screen. We will ask you to continue to pay attention to the music as best you can for the entire time the letter “M” is on the screen, including* ***during the bursts of heat.***

*Following the bursts of heat, you will be asked* ***to rate the pain*** *on two rating screens: the first for* ***intensity*** *and the second for* ***unpleasantness****. You can compare intensity to the volume on a radio and the unpleasantness as how much this noise bothers you. Thus, the intensity scale measures how hot the pain is, and the unpleasantness scale measures how much the pain bothers you. You will see a screen numbered* ***0-10*** *with an arrow underneath. You can* ***move the arrow using the outer two buttons*** *on the button box (pointer finger on blue, pinky on red), and once the arrow is below the number you would like to select you can press either of* ***the two inner buttons to make your choice****. The screens go by rather quickly so choose the number that corresponds with your first instinct. If you happen to make a mistake while you press, you can report it to me at the end of the block, but you should only do so if you failed to select the number you were trying to select. Do not change your mind after making a rating.*

*Please remember to keep your* ***eyes open*** *throughout the experiment and to* ***maintain your gaze*** *on the letter or cross at the center of the screen. It is very important that you don’t move during the experiment, so please remain extremely still. If you need to stop the experiment, please let us know.*

Emotion Regulation Task

*In the emotion regulation task, you will be viewing pictures and listening to descriptions about the pictures. Please listen to the descriptions carefully and view the picture for the entire time it is presented. One type of description you will hear will say that the* ***picture is a scene from a movie*** *and that the people are actors or actresses. Therefore,* ***the scene is not real****. Another type of description you will hear will ask you to imagine the person or people in the scene are* ***loved ones****. We want you to think of a loved one and imagine that whatever is happening in the scene is happening to him or her. Try to* ***really envision your loved one in the scene depicted.***

*Some of the pictures will be very disturbing and some will be neutral. If you are ever uncomfortable with the pictures, please let us know, and we will stop the study.*

fMRI Scan Runlog

**HEP-MBSR fMRI Scan Run Log**

**fMRI Scan (___/___/___)**

**1. PREPARATION before Subject arrives (Subject arrives 15 min before scan time. Please arrive at least 30 min before subject arrives)**

***If not written, check Y:\MBSR\fmri\**SimSession_Notes** or **Sim Session Runlog** in Mock folder***

 Record heat stimulation temperature from simulation session. **TEMPERATURE: _________°C_____**

 Subject needs **Vacuum pillow** CIRCLE ONE: Yes No

 Get fMRI Scan folder from “LUTZ” cabinet on second floor. Complete **Pre-Session Run Log**.

 Post “HEP-MBSR” greeting (located in copy room) by parking lot entrance if you have a session after 6pm. Or print out at Y:\MBSR\fmri\runlog\Welcome_sheet

**MRI Control Room**:

**a) Set up the thermal stimulator (Bring in equipment to MRI tech 30 min before scan if possible):**

 Get thermal stimulator and box from **Cabinet 9** in computer bay or get from previous session

 Set up heat stimulation **laptop** in MRI control room, near E-prime computer. Make sure it is **plugged in** and turn it on (push button on left).

 Hook up cables from **laptop** 🡪 **stimulator** by matching color labels. MRI technicians should set up the heat stimulator connections. All you need to do is plug the **yellow cable** into the laptop and plug in laptop power cord. Do not connect laptop to e-prime computer until after eye calibration.

 **Check water levels**: After laptop is connected to heat stimulator (yellow), Open “**shortcut to pain**” on laptop. Wait for system initialization to complete. Click “**test**” to turn on green light on stimulator. **THIS WAY MRI TECH CAN CHECK WATER LEVELS** before subject arrives!! Give MRI tech necessary supplies to fill water if necessary (screwdrivers are in PET room)

| *****MRI TECH WILL DO THIS*****  MRI COMPUTER ROOM (next to MRI Control Room, B1234 key)   Get thermode filter and grounding wire in drawer (top drawer under “sun” computer) in MRI Control  Room   Go to back of Computer room. Unscrew round cap (covers pipe) off of panel in back. Pull out end of  MRI thermode from the pipe. Attach end MRI thermode to filter.   Place tinfoil around exposed pipe (to prevent signal interference)   Plug in one end of grounding wire onto panel that says “Airline – J6 or J7). Attach black end of  grounding wire to a screw on the thermode filter.   Attach one end of long yellow cable to TSA Serial RS232 in computer room.  MRI CONTROL ROOM   Attach other end to laptop in control room.   **Turn on** thermal stimulator   If not enough water, use **screwdriver** to open, insert **distilled water** with **syringe** (all located in box). (If need to replace water, go to first sink on the right in T155 [open with key B1234]. Use right tap labeled “DW” (only distilled water can be used in the machine). Syringes located in drawers in T155). Maximum water to put in is 10ml. |
| --- |

**b) Login to and set up computers**

 **E-prime** computer: Open WinCal icon on desktop. Click “**Enable**”

 Set up **cat picture** ( Open “**Calibration Images**” folder on desktop, open **Align.ebs** and press **F7**

Enter Subject ID#1, Session #1, Yes to overwrite, click ok, MRI tech sometimes does this)

 Make sure viewing button is set to #**2** so the subject can see the image

**MRI Prep Room**:

**c) Prepare SCR sensors**

 Get SCR sensors (hanging), hydrophilic ointment, 2 collars, plastic knife, tape from cabinet in MRI control room

 Align collars over hole of sensors, align blue tab with cord. Press down for a few seconds to ensure adhesion.

 Use plastic knife to insert hydrophilic ointment into hole. Go back and forth with knife to fill hole

**d) Get out hard copies of questionnaires for participant**

**2. KECK WAITING ROOM (15 min before scan)**

 Welcome the participant. Ask him/her whether he/she needs a drink or to go to the restroom. If they use the restroom, ask them to only use warm water **WITHOUT SOAP** to wash their hands. This is so we get a good skin conductance response.

*Do you need to use the restroom? If you do, when you wash your hands,* ***please do not use soap.*** *We need to attach skin sensors to your fingers, and the soap will interfere with your skin response.* If does not need to use restroom [Bring participant to bathroom by the MRI Prep Room]: *The first we thing we need you to do is to was your hands* ***WITHOUT SOAP.*** *This is because we are attaching skin sensors to your fingers, and the soap will interfere with your skin response. When you are finished, you may enter the Prep Room* [point out room] *and we’ll fill out some questionnaires and prepare you for the scan.*

**3. MRI PREP ROOM**

 Complete the **MRI safety screening form** (Make sure everything is checked off and that weight and birthday are filled in)

*First we will do the MRI safety screen one more time to make sure that nothing has changed from your*

*last MRI screening, and that it is safe for you to be in the scanner.*

 If subject is female and is not sure if she is pregnant, get **pregnancy test** from cabinet in control room when you give tech the consent form and MRI screening. One line: not pregnant. 2 lines: pregnant. (Remind her to not use soap when washing hands)

 **Questionnaires**: Have subject fill out **3** questionnaires from the questionnaire packet. They fill out **New Medication Questionnaire, PANAS-General, and PANAS-Now Pre-scan. They do not fill out PANAS-Now Post-Scan until AFTER the scan.**

*Now you will fill out 3 questionnaires. The first one will ask you about new medications you have been taking. The next questionnaire which will ask about your feelings* ***IN GENERAL.*** *The questionnaire after that will ask you about the same feelings, but how you are feeling* ***RIGHT NOW.*** *Please do not fill out the 4^th^ questionnaire. We will fill it out after the scan.*

 While subject is filling out questionnaires, bring in completed **MRI safety** screening form and copy of **consent** form to **MRI tech** in MRI Control Room. Do **water test** if necessary.

 Ask MRI tech **how much more time** s/he needs.

 If haven’t already, have subject go to bathroom and wash hands with **warm water without soap!**

*Now we are going to attach some sensors to your fingers to measure your skin response. Please first wash your hands with warm water but WITHOUT SOAP. This is because the soap may interfere with measuring the skin response.*

 Prepare and attach SCR sensors (explain each step to the subject as you do them)

___Peel off white part of collars

___Attach to ring and middle fingers of **left hand**, center on fleshy part of finger

___Push down a little in the center, then push on sticky part of collar. Immediately secure sensors to fingers with tape.

___Cords run down arm, tape over **back of wrist**

___ Use deionized water to clean sensors after scan in room T168

*Now I am going to attach the skin sensors to the middle and fourth finger of your left hand. There will be one more sensor on your pointer finger to measure your heart rate.*

 Have subject read the **Instructions** (tear off back sheet of run log).

 Answer any questions.

 Write down subject’s loved ones. *For the emotion regulation task, please think of a few loved ones who are* ***male, female, and children****. If you see a picture of a male, envision one of your male loved ones. If you see a picture of a child, imagine one of your child loved ones. If you see a picture of a woman, imagine one of your female loved ones. If you see a group of people, imagine a group of loved ones. Remember to really envision your loved one in the scene depicted. Can you* ***tell me the relationship*** *you have with some of the loved ones you will use in the task?*

 **Confirm and record some loved ones** subject will use (e.g. best female friend, father, nephew)

_________________________________________________________________________________

 Give the following final reminders:

1) **MINIMIZING MOVEMENT**

*It is important to be as comfortable as possible when the MRI technician is setting you up so that you are less likely to move your head. Please feel free to be picky with your comfort when we are setting you up. Try your best not to move your body, especially the head, face, or upper body. Please refrain from moving during the scan as well as during the little breaks you will have. We will let you know if you are moving too much.*

2) **ORDER OF SCANS**

*First we will do the heat stimulation task, and we will remind you of the instructions once you are in the scanner. Then there will be a period of time where we are taking pictures of your brain and you can close your eyes. After that, we will do the emotion regulation task, and we will remind you of the instructions. We will be able to speak with you between each run through an* ***intercom****. We will also give you a* ***squeeze ball*** *which you can squeeze if you need to talk to us.* ***Do you have any questions?***

**KEEP EYES OPEN**

*Please keep your eyes open for the entire time unless we say it is ok for you to close them. Please maintain your gaze on the fixation point or cross and images. It is important for our data for you to keep your gaze fixed on the screen, even if you are only looking at a fixation cross. Do your best to keep your eyes on the fixation cross for the entire time.*

 Bring participant to MRI room and introduce him/her to the MRI technician.

**4. MRI CONTROL ROOM**

**1) Preparation**

 Have subject **remove everything from pockets, jewelry, piercings, etc.** (can store in the control room), can leave belongings on chair and in white box

 MRI Tech will do final bathroom and metals check with subject

 Be sure **YOU** **(NO WALLET, WATCH, COIN, PAPER CLIP, KEY, NECKLACE, CHAIN,**

**EAR/BELLY/TOE RING, AND ANYTHING FROM YOUR POCKETS)** do not have any metal on you

before going in the scanner room.

**5. SCANNER ROOM**

 Check Page 1 of runlog or Y:\MBSR\fmri\**SimSession_Notes** on SCR Computer to see **WHICH PILLOW** subject will use. Tell MRI Tech if using the **vacuum pillow** (will use neck brace otherwise)

 Assist MRI technician with neck brace/pillow, headphones, respiratory belt, adjust goggles (usually don’t have to do much)

 Give subject **four-button box**, so that pinky finger (right) is on the red button. Place their right hand four fingers on the four buttons and explain to them that they will move the rating screen right and left with the two outer buttons and stop the arrow with either of the two inner buttons.

 **Tape down button box** to make it more secure

 **Tape down squeeze ball** and explain they can squeeze it if anything goes wrong or they want to talk to us (MRI tech usually explains)

**6. MRI CONTROL ROOM**

**1) SET UP MRI SCAN COMPUTERS (Heat Stimulation Task)**

**a) E-Prime Computer: Goggle Calibration (MRI Tech usually does this)**

 Take down cat picture (Control + Alt + Shift)

 If not open, open Wincal and click Enable

 Make sure eye-tracking device is turned on and viewing button set to 2

 MRI tech will calibrate eye-tracking

 **Disable Wincal and close program (E-prime will not work if Wincal is not disabled)**

**7. MRI SCANNER ROOM**

 Assist MRI Tech with putting subject into scanner. Ensure subject comfort.

**8. MRI CONTROL ROOM: Heat Stimulation Task**

***WHILE MRI TECH DOES FIELD MAPPING***

**a)** **Serial test**: Make sure **laptop and PC are communicating**.

___**Unplug Avotec serial port** (needed for calibration) from serial cable from E-prime computer

___**Plug in** **laptop 🡪 E-prime computer** cable (match up **pink**)

___**Laptop**: Go to “**Settings**” on start menu 🡪 “**Control Panel**” 🡪 “**Accessibility Options**” 🡪 “**General**” tab 🡪 **UNCLICK “Support Serial Key Devices”** 🡪 then **RECLICK** 🡪 “**Apply**” 🡪 “**OK**”

___**Laptop**: Open Desktop “GO MBSR TEAM” word file

___On **E-prime computer**: Go to desktop folder “HEPMBSR”, open “**testSerial.ebs**” (running

man icon), press **F7**. Enter **any subject #** (does not matter for serial test), any session #.

___Make sure when you hit space bar on E-prime computer, it moves a space on the laptop.

___Stop program by hitting Control + Alt + Shift

| **b) Check RANDOMIZATION and TEMPERATURE VERY IMPORTANT!**   Get **HEPMBSR fMRI BINDER.** Turn to “HEPMBSR Pain Task Subject Randomization” (first  page), and assign current subject the **next available randomization**. **Write in Subject ID**.  **c) Check Subject Temperature VERY IMPORTANT!**   Either written on Page 1 of fMRI Scan Run Log. Or SCR Computer: open  Y:\MBSR\fmri\**SimSession_Notes** |
| --- |
| **HEAT STIMULATION RANDOMIZATION** |
| **Record Randomization Code:__________________**  **Record E-Prime file name: ________________________________________.ebs** |
| **HEAT STIMULATION TEMPERATURE** |
| **Subject Temperature:___________°C** |

**d) VOLUME CHECK** (MRI Tech talks to subject)

 Play experiment sounds for subject while tech plays scanner sounds. Desktop: “HEPMBSR”, play

**instruction_HEP_pain.wav** and **10.wav**

 MRI Tech will find optimal sound levels

**e) Computers: Heat Stimulation Task**

 **Laptop**: Open pain.exe on desktop. Check temperature from Page 1 of Run Log or (c). Choose panel that says **HEP_MBSR_(correct temperature)**. **Press spacebar**.

*****WHEN MRI TECH DONE WITH MANUAL PRE-SCAN***** (this is to avoid premature triggering of the e-prime program)

 **E-prime computer**: Open “**HEPMBSR**” folder on desktop. Open appropriate **“HEP_MBSR_painorder_#_#.ebs**” file written above, press **F7**, enter **Subject ID** and **Session # (Make sure this is correct!)**. (Aug/Sept=1, Nov/Dec=2, Mar/Apr=3). Do note overwrite any files!

 E-prime computer should say “Wait for ttl”. Laptop should say “Waiting for a trigger”.

**f) HEAT STIMULATION TASK INSTRUCTIONS:**

**Read to subject over intercom**

*How are you doing? You’re doing a great job.*

*First we will do the heat stimulation task. To remind you of the task, let me ask a couple of questions to*

*make sure you understood the instructions:*

*-What will you be focusing your attention on when the* ***cross*** *is on the screen? (*Experimenter*: “That’s right/wrong, you will be focusing on* ***your sensations, emotions and thoughts****”)*

*What will you be focusing your attention on when the letter “****M****” is on the screen? (*Experimenter*: “That’s right/wrong, you will* ***be focusing on the music****”)*

*You will be asked* ***to rate the pain*** *on two rating screens: the first for* ***intensity*** *and the second for* ***unpleasantness****. The intensity scale measures how* ***hot*** *the pain is, and the unpleasantness scale measures how much the* ***pain bothers you****. You will see a screen numbered* ***0-10*** *with an arrow underneath. You can* ***move the arrow using the two outer buttons*** *on the button box and once the arrow is below the number you would like to select you can press either of* ***the two inner buttons to make your choice****.* (Lightly touch subject’s fingers so they know which buttons to press, make sure they understand how to use it.)

*Please remember to keep your* ***eyes open*** *throughout the experiment and to* ***maintain your gaze*** *on the letter or cross at the center of the screen. It is very important that you don’t move during the experiment, so please remain extremely still. If you need to stop the experiment, please let us know.*

*Are you ready to start the heat stimulation task?*

*****WHEN MRI TECH IS READY for FUNCTIONAL SCANNING*****

**g) HEAT STIMULATION TASK RUN 1**

(Make sure E-prime computer, laptop, and eye gaze computer are all triggered to collect data)

**1) E-prime computer**

 Make sure the viewing button is on **#2** . This way, the participant can see the images.

 When MRI triggers, the program will start.

If there is a mistake, and you need to start over E-prime at Run 1. Quit out of eprime Control+Alt+Shift. Restart e-prime file. Enter subject #. For **Session #, enter “11” at Time 1**, “21” at Time 2, “31” at Time 3.

*****MRI TECH WILL COUNTDOWN TO SCAN*****

**2) Eye Tracking Computer**

 To start eye-tracking: Wait for tech’s countdown. **Press F3** to start. Make sure the clock at bottom starts.

**3) Run 1 Data Monitoring (Log simultaneously in Y:\MBSR\fmri\fmri_scan_notes)**

RUN 1 BEGINS

| *****RUN 1 MONITORING*** NOTES** | | | |
| --- | --- | --- | --- |
| 1. Laptop: **heat** triggered | **YES** | NO |  |
| 2. Subject is making **ratings** correctly  (check light box above E-prime computer) | **YES** | NO |  |
| 3. **Sleepiness**  (via eye-tracking, verbal report, etc.) | YES | **NO** |  |
| 4. Suboptimal **eye-tracking** activity  (flickering, zooming in and out of screen) | YES | **NO** |  |
| 5. **Movement**  (due to speaking, discomfort, sneezing, etc)  from **AFNI** Motion Plot  (no large spikes, drift ok) | YES  YES | **NO**  **NO** |  |
| 6. Suboptimal **heart rate** collection | YES | **NO** |  |
| 7. Suboptimal **respiration** collection | YES | **NO** |  |
| 8. E-prime problems | YES | **NO** |  |
| 9. End of scan: subject comfortable?  **Remind subject** how to use button box or about movement, etc. | **YES**  YES | NO  NO |  |
| **GENERAL RUN 1 NOTES** | | | |

*****END OF RUN***
4) Eye-tracking computer**

 At around 8 min when scan is over, hit **F3** or **STOP** icon. Click **SAVE** icon. Save in **C:\Data\HEP_MBSR_Pain**. Naming convention: **S(###)_pain_time(#)_run(#). (**Example: S042_pain_time1_run2)

 Hit the **WHITE PAGE** icon to reinitialize the program. Clock should go to 00:00

**5) E-prime computer**

 You will see “End of Run” screen. **DO NOT ADVANCE TO NEXT SCREEN** until MRI Tech has done the **manual pre-scan**.

**6)** MRI tech checks subject comfort. **Remind** subject about ratings, movement, etc. if necessary. Be **warm and encouraging.**

*****WHEN MRI TECH IS DONE WITH MANUAL PRE-SCAN*****

**h) HEAT STIMULATION TASK RUN 2**

(Make sure E-prime computer, laptop, and eye gaze computer are all triggered to collect data)

**1) E-prime computer**

 Now **click the mouse** to advance to “Wait for ttl” screen. When MRI triggers, the program will start.

If there is a mistake, and you need to start over E-prime at Run 2. Go to “**Emergency**” folder.

Choose appropriate file according to troubleshooting guide in binder.

*****MRI TECH WILL COUNTDOWN TO SCAN*****

**2) Eye Tracking Computer**

 To start eye-tracking: Wait for tech’s countdown. **Press F3** to start. Make sure the clock at bottom starts.

**3) Run 2 Data Monitoring (Log simultaneously in Y:\MBSR\fmri\fmri_scan_notes)**

RUN 2 BEGINS

| *****RUN 2 MONITORING*** NOTES** | | | |
| --- | --- | --- | --- |
| 1. Laptop: **heat** triggered | **YES** | NO |  |
| 2. Subject is making **ratings** correctly  (check light box above E-prime computer) | **YES** | NO |  |
| 3. **Sleepiness**  (via eye-tracking, verbal report, etc.) | YES | **NO** |  |
| 4. Suboptimal **eye-tracking** activity  (flickering, zooming in and out of screen) | YES | **NO** |  |
| 5. **Movement**  (due to speaking, discomfort, sneezing, etc)  from **AFNI** Motion Plot  (no large spikes, drift ok) | YES  YES | **NO**  **NO** |  |
| 6. Suboptimal **heart rate** collection | YES | **NO** |  |
| 7. Suboptimal **respiration** collection | YES | **NO** |  |
| 8. E-prime problems | YES | **NO** |  |
| 9. End of scan: subject comfortable?  **Remind subject** how to use button box or about movement, etc. | **YES**  YES | NO  NO |  |
| **GENERAL RUN 2 NOTES** | | | |

*****END OF RUN***
4) Eye-tracking computer**

 At around 8 min when scan is over, hit **F3** or **STOP** icon. Click **SAVE** icon. Save in **C:\Data\HEP_MBSR_Pain**. Naming convention: **S(###)_pain_time(#)_run(#). (**Example: S042_pain_time1_run2)

 Hit the **WHITE PAGE** icon to reinitialize the program. Clock should go to 00:00

**5) E-prime computer**

 You will see “End of Run” screen. **DO NOT ADVANCE TO NEXT SCREEN** until MRI Tech has done the **manual pre-scan**.

**6)** MRI tech checks subject comfort. **Remind** subject about ratings, movement, etc. if necessary. Be **warm and encouraging.**

*****WHEN MRI TECH IS DONE WITH MANUAL PRE-SCAN*****

**h) HEAT STIMULATION TASK RUN 2**

(Make sure E-prime computer, laptop, and eye gaze computer are all triggered to collect data)

**1) E-prime computer**

 Now **click the mouse** to advance to “Wait for ttl” screen. When MRI triggers, the program will start.

If there is a mistake, and you need to start over E-prime at Run 3. Go to “**Emergency**” folder.

Choose appropriate file according to troubleshooting guide in binder.

*****MRI TECH WILL COUNTDOWN TO SCAN*****

**2) Eye Tracking Computer**

 To start eye-tracking: Wait for tech’s countdown. **Press F3** to start. Make sure the clock at bottom starts.

**3) Run 3 Data Monitoring (Log simultaneously in Y:\MBSR\fmri\fmri_scan_notes)**

RUN 3 BEGINS

| *****RUN 3 MONITORING*** NOTES** | | | |
| --- | --- | --- | --- |
| 1. Laptop: **heat** triggered | **YES** | NO |  |
| 2. Subject is making **ratings** correctly  (check light box above E-prime computer) | **YES** | NO |  |
| 3. **Sleepiness**  (via eye-tracking, verbal report, etc.) | YES | **NO** |  |
| 4. Suboptimal **eye-tracking** activity  (flickering, zooming in and out of screen) | YES | **NO** |  |
| 5. **Movement**  (due to speaking, discomfort, sneezing, etc)  from **AFNI** Motion Plot  (no large spikes, drift ok) | YES  YES | **NO**  **NO** |  |
| 6. Suboptimal **heart rate** collection | YES | **NO** |  |
| 7. Suboptimal **respiration** collection | YES | **NO** |  |
| 8. E-prime problems | YES | **NO** |  |
| 9. End of scan: subject comfortable?  **Remind subject** how to use button box or about movement, etc. | **YES**  YES | NO  NO |  |
| **GENERAL RUN 3 NOTES** | | | |

*****END OF RUN***
4) Eye-tracking computer**

 At around 8 min when scan is over, hit **F3** or **STOP** icon. Click **SAVE** icon. Save in **C:\Data\HEP_MBSR_Pain**. Naming convention: **S(###)_pain_time(#)_run(#). (**Example: S042_pain_time1_run2)

 Hit the **WHITE PAGE** icon to reinitialize the program. Clock should go to 00:00

**5) E-prime computer**

 **Click mouse** to end E-prime program

**6)** MRI tech checks subject comfort. You **remind** subject about movement, etc. if necessary. Be **warm and encouraging.**.

**9. Collection of ANATOMICAL data**

 Press a different button other than #2 on visual box to blank out screen.

 MRI Tech will tell the participant that we will collect pictures of their brain while they remain still in the scanner. The participant can relax and close eyes.

 Tell Lisa to let the subject know

*You can now* ***let go of the button box*** *since you will no longer need to use it. Also, if the* ***thermode*** *is bothering you, you may use your hands to carefully remove it. Try your best not to move your head while you do this.*

 **UNPLUG laptop serial port** from e-prime computer

 **PLUG IN Avotec serial port** (from eye tracking computer) to e-prime computer

 **CLEAN UP pain equipment** if NO OTHER SCANS after this one. Save time!

**10. RECALIBRATE AVOTEC AND WINCAL for Emotion Regulation Task**

 MRI Tech tells subject to look at dots

 E-prime computer: Open Wincal and click Enable

 MRI tech will set viewing button set to 2 and calibrate eye-tracking

 **Disable Wincal and close program (E-prime will not work if Wincal is not disabled)**

**11.** **EMOTION REGULATION TASK**

**a) Emotion Regulation Randomization (Make sure this is correct!)**

 Check **HEPMBSR fMRI binder**. Turn to “HEPMBSR Emotion Regulation Task Subject Randomization,” assign current subject the **next available randomization**. **Write in Subject ID**.

| **EEmotion Regulation Randomization** |
| --- |

**Record Time Point**  1  2  3 **E-prime Set**:  A  B

**Record E-prime file name:___________________________________________________.ebs**

**b) SCR Computer (SET UP DURING ANATOMICALS)**

 Open “Shortcut to **AquireSignal**” on SCR Computer desktop

 Select from menu: **Operate** 🡪 **Data Logging** 🡪 **Changing File Binding** 🡪 **(My Computer: C:/HEPMBSR**) 🡪 **hepmbsr_events** 🡪 **Save** 🡪 **OK**

 Select from menu: **Operate** 🡪 **Data Logging** 🡪 **Retrieve**

 **OK** 🡪 **white arrow** in upper left corner and enter

“hepmbsr###_(time point#)_(Set)_run(#)”, (e.g. “hepmbsr025_1_A_run2, hepmbsr025_1_A_run3”)

**DO NOT HIT SAVE YET!!!!!**

*****AFTER ANATOMICALS ARE FINISHED*****

*****READ SUBJECT EMOTION REGULATION INSTRUCTIONS*****

*To remind you of the emotion regulation task, you will be viewing pictures and listening to descriptions about the pictures. Please listen to the descriptions carefully and view the picture for the entire time it is presented. One type of description you will hear will say that the* ***picture is a scene from a movie*** *and that the people are actors or actresses. Therefore,* ***the scene is not real****. Another type of description you will hear will ask you to imagine the person or people in the scene are* ***loved ones****. We want you to think of a loved one and imagine that whatever is happening in the scene is happening to him or her. Try to* ***really envision your loved one in the scene depicted.***

*Remember to use the loved ones you thought of before*. Remind subject of loved ones (from Page 3): ________________________________________________________________________________

*Please keep your* ***eyes open*** *throughout the experiment and to* ***maintain your gaze*** ***on the fixation cross*** *at the center of the screen. Always look at the fixation cross when it is on the screen. It is very important that you don’t move during the experiment, so please remain extremely still. If you need to stop the experiment, please let us know.*

**c) E-Prime Computer: Emotion Regulation Task**

 Check participant’s comfort and attention.

 If MRI tech asks, the protocol is **238 boldreps**

*****WHEN MRI TECH IS DONE WITH MANUAL PRE-SCAN***** (to avoid premature triggering of e-prime program)

 Open folder on desktop labeled “**HEPMBSR\ ER\**”. Open appropriate **Time period** (e.g. Time 1) Open appropriate e-prime file according to participant randomization (see above). **Press F7.** Enter **subject # and session #** (Aug/Sept=1, Nov/Dec=2, Mar/Apr=3). Do not overwrite any files!

 Make sure the #2 button on the box to the left of the eye-tracking computer is pressed. This way, the participant can see the images.

 Screen should say “**Get Ready!**” No further action is required as it will be triggered when MRI tech starts scan.

 Make sure **E-prime computer and SCR computer are ready** before functional scans begin

E-Prime Computer

*****CONFIRM WITH MRI TECH THAT YOU ARE READY*****

**d) SCR Computer**

 **Click “save**” **right before** **scan block begins**

 Skin conductance data – verify that it’s picking it up (you see activity)!

___Adjust the window: double-click values on the y-axis, enter #(about 8), then press ENTER

Change Y-axis values to closely view the response.

___If it clips or goes out of the window, adjust the y-axis again!

 Screen will freeze when scan is over. Exit when done and **restart** from the **very first step** (each run)

**e) Run 1 Data Monitoring** **(Log simultaneously in Y:\MBSR\fmri\fmri_scan_notes)**

RUN 1 BEGINS

| *****RUN 1 MONITORING*** NOTES** | | | |
| --- | --- | --- | --- |
| 1. **Sleepiness**  (via eye-tracking, verbal report, etc.) | YES | **NO** |  |
| 2. Suboptimal **eye-tracking** activity  (flickering, zooming in and out of screen) | YES | **NO** |  |
| 3. **Movement**  (due to speaking, discomfort, sneezing, etc)  from **AFNI** Motion Plot  (no large spikes, drift ok) | YES  YES | **NO**  **NO** |  |
| 4. Suboptimal **skin conductance** collection | YES | **NO** |  |
| 5. Suboptimal **heart rate** collection | YES | **NO** |  |
| 6. Suboptimal **respiration** collection | YES | **NO** |  |
| 7. E-prime problems | YES | **NO** |  |
| 8. End of scan: subject comfortable?  **Remind subject** about movement, etc. | **YES**  YES | NO  NO |  |
| **GENERAL RUN 1 NOTES** | | | |

*****END OF RUN*****

 Close SCR setup. Start up from beginning.

**1) SCR Computer**

 Open “Shortcut to **AquireSignal**” on SCR Computer desktop

 Select from menu: **Operate** 🡪 **Data Logging** 🡪 **Changing File Binding** 🡪 **(My Computer: C:/HEPMBSR**) 🡪 **hepmbsr_events** 🡪 **Save** 🡪 **OK**

 Select from menu: **Operate** 🡪 **Data Logging** 🡪 **Retrieve**

 **OK** 🡪 **white arrow** in upper left corner and enter

“hepmbsr###_(time point#)_(Set)_run(#)”, (e.g. “hepmbsr025_1_A_run2, hepmbsr025_1_A_run3”)

**DO NOT HIT SAVE YET!!!!!**

*****WHEN MRI TECH DONE WITH MANUAL PRE-SCAN*****

**2) E-Prime Computer**

 Screen will say “Please Remain Still!” when block is over. Wait for MRI tech to **start the manual pre-scan**. Confirm with MRI tech, then **hit the mouse button once**, and you will see “GET READY!”**ONLY** click mouse if screen says “PLEASE REMAIN STILL!” **NEVER click** when screen says “GET READY!”

 If you need to restart the paradigm at a certain block, go to the “Blocks 2-3” folder. Choose the numbered file that corresponds to the number of the block you wish to start with

 Make sure **E-prime computer and SCR computer are ready** before functional scans begin

E-Prime Computer

If there is a mistake, and you need to start over E-prime at Run 2. Go to “**Emergency Eprime** (Runs 2-3)” folder. Go to A folder or B folder. Choose **Run2**_.ebs file. Check binder for details.

*****CONFIRM WITH MRI TECH THAT YOU ARE READY*****

**d) SCR Computer**

 **Click “save**” **right before** **scan block begins**

 Skin conductance data – verify that it’s picking it up (you see activity)!

___Adjust the window: double-click values on the y-axis, enter #(about 8), then press ENTER

Change Y-axis values to closely view the response.

___If it clips or goes out of the window, adjust the y-axis again!

 Screen will freeze when scan is over. Exit when done and **restart** from the **very first step** (each run)

**Run 2 Data Monitoring** **(Log simultaneously in Y:\MBSR\fmri\fmri_scan_notes)**

RUN 2 BEGINS

| *****RUN 2 MONITORING*** NOTES** | | | |
| --- | --- | --- | --- |
| 1. **Sleepiness**  (via eye-tracking, verbal report, etc.) | YES | **NO** |  |
| 2. Suboptimal **eye-tracking** activity  (flickering, zooming in and out of screen) | YES | **NO** |  |
| 3. **Movement**  (due to speaking, discomfort, sneezing, etc)  from **AFNI** Motion Plot  (no large spikes, drift ok) | YES  YES | **NO**  **NO** |  |
| 4. Suboptimal **skin conductance** collection | YES | **NO** |  |
| 5. Suboptimal **heart rate** collection | YES | **NO** |  |
| 6. Suboptimal **respiration** collection | YES | **NO** |  |
| 7. E-prime problems | YES | **NO** |  |
| 8. End of scan: subject comfortable?  **Remind subject** about movement, etc. | **YES**  YES | NO  NO |  |
| **GENERAL RUN 2 NOTES** | | | |

*****END OF RUN*****

 Close SCR setup. Start up from beginning.

**1) SCR Computer**

 Open “Shortcut to **AquireSignal**” on SCR Computer desktop

 Select from menu: **Operate** 🡪 **Data Logging** 🡪 **Changing File Binding** 🡪 **(My Computer: C:/HEPMBSR**) 🡪 **hepmbsr_events** 🡪 **Save** 🡪 **OK**

 Select from menu: **Operate** 🡪 **Data Logging** 🡪 **Retrieve**

 **OK** 🡪 **white arrow** in upper left corner and enter

“hepmbsr###_(time point#)_(Set)_run(#)”, (e.g. “hepmbsr025_1_A_run2, hepmbsr025_1_A_run3”)

**DO NOT HIT SAVE YET!!!!!**

*****WHEN MRI TECH DONE WITH MANUAL PRE-SCAN*****

**2) E-Prime Computer**

 Screen will say “Please Remain Still!” when block is over. Wait for MRI tech to **start the manual pre-scan**. Confirm with MRI tech, then **hit the mouse button once**, and you will see “GET READY!”**ONLY** click mouse if screen says “PLEASE REMAIN STILL!” **NEVER click** when screen says “GET READY!”

 If you need to restart the paradigm at a certain block, go to the “Blocks 2-3” folder. Choose the numbered file that corresponds to the number of the block you wish to start with

 Make sure **E-prime computer and SCR computer are ready** before functional scans begin

E-Prime Computer

If there is a mistake, and you need to start over E-prime at Run 3. Go to “**Emergency Eprime** (Runs 2-3)” folder. Go to A folder or B folder. Choose **Run3**_.ebs file .Check binder for details.

*****CONFIRM WITH MRI TECH THAT YOU ARE READY*****

**d) SCR Computer**

 **Click “save**” **right before** **scan block begins**

 Skin conductance data – verify that it’s picking it up (you see activity)!

___Adjust the window: double-click values on the y-axis, enter #(about 8), then press ENTER

Change Y-axis values to closely view the response.

___If it clips or goes out of the window, adjust the y-axis again!

 Screen will freeze when scan is over. Exit when done and **restart** from the **very first step** (each run)

**Run 3 Data Monitoring** **(Log simultaneously in Y:\MBSR\fmri\fmri_scan_notes)**

RUN 3 BEGINS

| *****RUN 3 MONITORING*** NOTES** | | | |
| --- | --- | --- | --- |
| 1. **Sleepiness**  (via eye-tracking, verbal report, etc.) | YES | **NO** |  |
| 2. Suboptimal **eye-tracking** activity  (flickering, zooming in and out of screen) | YES | **NO** |  |
| 3. **Movement**  (due to speaking, discomfort, sneezing, etc)  from **AFNI** Motion Plot  (no large spikes, drift ok) | YES  YES | **NO**  **NO** |  |
| 4. Suboptimal **skin conductance** collection | YES | **NO** |  |
| 5. Suboptimal **heart rate** collection | YES | **NO** |  |
| 6. Suboptimal **respiration** collection | YES | **NO** |  |
| 7. E-prime problems | YES | **NO** |  |
| 8. End of scan: subject comfortable?  **Remind subject** about movement, etc. | **YES**  YES | NO  NO |  |
| **GENERAL RUN 3 NOTES** | | | |

*****END OF RUN*****

 Close SCR setup.

 **Click mouse** to end e-prime program

**12. Post-fMRI Scan Procedures*.***

**a) MRI CONTROL ROOM:**

 Get **MRI data sheet** from MRI tech. Put in subject folder.

 Bring participant to MRI Control Room. **Take SCR sensors off.** Subject gathers belongings.

**b) MRI PREP ROOM**

 Bring Pp to MRI Prep Room. Have subject fill out "**PANAS-NOW (post-scan)**” questionnaire.

 **Paper questionnaires**: If **IRI** and **RASQ** are in subject’s folder, have subject fill them out by hand.

 Get Subject's self-report about the scan

| **Heat Stimulation Task:**  Do you have any feedback or comment about your experience during the heat stimulation task?  **Emotion Regulation Task:**  Were you able to really think that the pictures were a scene from a movie?  Were you able to really envision and think of your loved ones when you were instructed? | |
| --- | --- |
| **General Questions:**  Were you comfortable during the time you were in the scanner? **Y**  N  Did you ever feel anxious before or during the scanning session? Y **N**  Were you always able to see the display? **Y**  N  At any time did you doze off? Y **N**  Do you recall closing your eyes or averting your gaze while Y **N**  the pictures were on?  Did you feel any tingling or twitching in your hands or arms? Y  **N** | **Notes** |

 **Post-Scan IAPS Ratings**. **Set up survey monkey** :

___Open internet explorer, go to [www.surveymonkey.com](http://www.surveymonkey.com/)

Login: **ajshackman@gmail.com**, password: **LAN_acct1**

____Go to “**HEP-MBSR**” folder from pull-down menu

____Click on link to "**HEPMBSR ER Ratings**"

____Subject enters SubjectID and TV Character

____**Delete cookies when finished! Tools -> Options -> Delete Files**

*How are you feeling? If you are feeling up to it, we have one more computer task for you. You will make ratings about the pictures you just saw on a scale from 1-9. You will be asked to rate the pictures on how pleasant or unpleasant they make you feel, how arousing (which means excited or anxious) the pictures make you feel, how much you want to look at or approach the person in the picture, and how much you want to look away or withdraw from the person.* (elaborate if necessary, especially for "arousal")

 If subject does not want to complete ratings (get yellow folder in prep room):

*Would you be able to fill out the ratings at home?*

***-* If has internet** (hand sheet with surveymonkey address on it): *Could you please go to this website at home and make the picture ratings?* (Explain directions out loud.)

**- If does not have internet** (hand ratings packet and stamped envelope): *Could you fill these ratings out and mail them back to us in this envelop?* (Explain directions out loud)

 While participant completing IAPS ratings, **wash out SCR sensors** and do **data management.**

 Complete relevant **post-scan run log** procedures (reminder notice, payment form, etc)

 Thank subject for participation.

*Thank you for participating in the fMRI part of the study. You did very well.*

 Get picture of brain from copy room. Attach sticker (in Michael Anderle’s mailbox) to picture. Give to subject.

**13. Data Management**

**a) Subject Randomizations (Check binder).** On SCR computer:

 Pain: log Subject ID in “Y:\MBSR\fmri\**HEPMBSR_Pain_Randomization**”. **Save.**

 ER: **Enter Subject ID and your initials in** “Y:\MBSR\fmri\**HEPMBSR_ER_Randomization**”. **Save.**

**b) E-prime computer: HEPMBSR folder**

 Delete testSerial##.txt file

 Copy **pain** .edat and .txt data files from Desktop: **HEPMBSR/pain/** to Y drive: Y:\MBSR\fmri\**eprime_pain_data**

 Drag **pain** .edat and .txt files to folder “**pain_time1_eprime_data**”

 Copy **emotion regulation** .edat and .txt data files from **HEPMBSR/ER/Time 1** to Y drive: Y:\MBSR\fmri\**eprime_er_data**

 Drag **emotion regulation** .edat and .txt files to “**ER_eprime_data_time1**”

 If hard drive is getting full, delete files from both folders on the hard drive. Make sure it is on Y Drive!!!!!!!

**c) Eye-tracking computer**

 Copy **pain** eye track files (C:\DATA\**HEP-MBSR_pain**) to Y drive: Y:\MBSR\fmri\**eye_pain_data\Time 1**.

 Copy **emotion regulation** eye track files (C:\DATA\**HEPMBSR**) to Y drive: Y:\MBSR\fmri\**eye_er_data\Time 1.**

 Drag data on hard drive to “**ER_eye_data**”

 If hard drive is getting full, delete files. Make sure it is on Y Drive!!!!!!!

**d) SCR Computer**

 Copy files (C:\HEPMBSR) to Y drive: Y:\MBSR\fmri\**scr_er_data.**

 Drag data to “**HEPMBSR_SCR**”

 If hard drive is getting full, delete files from “HEPMBSR_SCR”. Make sure it is on Y Drive!!!!!!!

**e) fMRI Scan Notes**

 **Log scan notes** in “Y:\MBSR\fmri\**fmri_scan_notes**.xls” (should do this as monitoring data)

**9. Clean Up**

 Put away pain equipment if needed in Cabinet 9

 Logout of computers

 Complete **post-session run log** and put away folder

 Bring “HEP-MBSR” greeting back to copy room if necessary (check to see if another participant is coming)

***FOR ANY PROBLEMS, REFER TO **TROUBLESHOOTING** SECTION OF BINDER***

#### Important Contact Information

General issues: **Antoine Lutz** (heat stim task), office: (26)2-8705, cell: 608-335-9845, room T-233, alutz@wisc.edu

**Helen Weng** (emot reg task), office: (26)3-0269, cell: 917-543-7292, room S117-D, hweng@wisc.edu

Jenna Sheftel (scheduling), cell: 847-602-0482, mbsrstudy@gmail.com

Pain equipment/software issues: **Tim Salomons**, office: (26)3-1968, room A-123, tvsalomons@wisc.edu

_________________________________________________________________________________

**NOTES:**

HEP-MBSR fMRI Study

Instructions Sheet

Heat Stimulation Task

***INSTRUCTIONS***

*In the heat stimulation task, you will receive cues and instructions telling you where to focus your attention. You will also receive several bursts of heat, some of which may be painful. On some trials you will be instructed to* ***notice sensations, emotions and thoughts****. This will be accompanied by a* ***cross*** *that will appear on the screen. We will ask you to continue to pay attention to your sensations, emotions and thoughts as best you can for the entire time the cross is on the screen, including* ***during the bursts of heat****. On other trials you will be instructed to* ***notice the music****. This will be accompanied by the letter “****M****” which will appear on the screen. We will ask you to continue to pay attention to the music as best you can for the entire time the letter “M” is on the screen, including* ***during the bursts of heat.***

*Following the bursts of heat, you will be asked* ***to rate the pain*** *on two rating screens: the first for* ***intensity*** *and the second for* ***unpleasantness****. You can compare intensity to the volume on a radio and the unpleasantness as how much this noise bothers you. Thus, the intensity scale measures how hot the pain is, and the unpleasantness scale measures how much the pain bothers you. You will see a screen numbered* ***0-10*** *with an arrow underneath. You can* ***move the arrow using the outer two buttons*** *on the button box and once the arrow is below the number you would like to select you can press either of* ***the two inner buttons to make your choice****. The screens go by rather quickly so choose the number that corresponds with your first instinct. If you happen to make a mistake while you press, you can report it to me at the end of the block, but you should only do so if you failed to select the number you were trying to select. Do not change your mind after making a rating.*

*Please remember to keep your* ***eyes open*** *throughout the experiment and to* ***maintain your gaze*** *on the letter or cross at the center of the screen. It is very important that you don’t move during the experiment, so please remain extremely still. If you need to stop the experiment, please let us know.*

Emotion Regulation Task

*In the emotion regulation task, you will be viewing pictures and listening to descriptions about the pictures. Please listen to the descriptions carefully and view the picture for the entire time it is presented. One type of description you will hear will say that the* ***picture is a scene from a movie*** *and that the people are actors or actresses. Therefore,* ***the scene is not real****. Another type of description you will hear will ask you to imagine the person or people in the scene are* ***loved ones****. We want you to think of a loved one and imagine that whatever is happening in the scene is happening to him or her. Try to* ***really envision your loved one in the scene depicted.***

*Some of the pictures will be very disturbing and some will be neutral. If you are ever uncomfortable with the pictures, please let us know, and we will stop the study.*

*Please remember to keep your* ***eyes open*** *throughout the experiment and to* ***maintain your gaze*** *on the fixation cross at the center of the screen. Please do not let your gaze wander, and fixate on the cross every time you see it. Please keep your eyes on the fixation cross even though it is not very interesting. This will help us with our data analysis. It is also very important that you don’t move during the experiment, so please remain extremely still. If you need to stop the experiment, please let us know.*

TSST Runlog

## HEP-MBSR Studies

## TSST Runlog (under progress)

Subject ID:____________ Session: _______________ Date: _______________

TSST committee:____________________________ Experimenter(s):__________________

TSST Visit # (circle one): **1 2 3**

Comments on the session:

__________________________________________________________________________________________________________________________________________________________________________________________________________________________________________________________________________________________________________________________________________________________________________________________________________________________________________________________________________________________________________________________________________________________________

___________________________________________________________________________________________________________________________________________________________________________________________________________________________________________________________________________________________________________________________________________________________________________________________________________________________________________________________________________________________________________________________________________________________________________________________________________________________________________________________________________________________________________________________________________________________________________________________________________________________________________________________________________________________________________________________________________________________________________________________________________________________________________________________________

________________________________________________________________________________________________________________________________________________________________________________________________________________________________________________________________________________________________________________________

Experimenter ____________________ Date______________ Time #1

TSST committee:

# TSSTcap Experimenter Runlog & Script

**To prepare for the session:**

1. label paperwork and videotape
2. label 6 salivettes with subject ID, sample #, and time (1, 2, or 3)
3. label 2 microcentrifuge tubes for blister fluid with subject ID, sample #, and time (1,2, or 3)
4. experiment room set-up:
   - [materials list]
5. TSST room set-up:
   - video camera & tripod
   - microphone
   - TSST forms

**Experiment Room Phase I:**

|  | **relative time** | **actual time** |
| --- | --- | --- |
| arrival | 0 |  |
| saliva sample #1 | +20 |  |
| blister start |  |  |
| blister finish |  |  |
| saliva sample #2 |  |  |

1. Session Overview: “Before we begin today, I will run through what you'll be doing to refresh your memory. First you'll just rest here for about 20 minutes and then we'll take your first saliva sample. Next we begin raising the blisters on your arm, but I'll explain how that works in more detail when we get there. Then we'll go to another room to do the stress test. Finally, we'll return here to put the cream on your arm and finish up the saliva samples. Overall, this session should take a little over 2 hours. Do you have any questions?”
2. Baseline cortisol sample: “We will begin by collecting a sample of your saliva. Please take the cotton roll out of this tube and chew on it for about a minute, until it is saturated. Then put it back into the tube and put the cap back on.”

Experimenter ____________________ Date______________ Time #1

TSST committee:

1. Computerized questionnaires: “While we raise the blisters on your arm, you’ll be completing several questionnaires on the computer.” Computerized Questionnaires: Start internet explorer, go to Survey Monkey (<http://www.surveymonkey.com/>), enter name and password, type in participant ID # and begin first questionnaire.
2. Blister induction (nurse): “Next we will begin the process that raises the blisters on your arm. This machine here is a vacuum that will apply suction to your skin through the holes in this template. It will feel like a pinch. The pinch will probably feel like it gets a little stronger and then a little weaker as the process continues. The nurse will be checking on the progress regularly. When the blisters begin to form, the suction will be decreased slightly. The time it takes for the blisters to form is different for everyone, but will probably take about an hour. During this process, please sit quietly, relax and close your eyes if that is comfortable for you.”

Notes:

1. Removal of fluid from baseline blisters (nurse)

Notes: “How would you rate your discomfort during the blister procedure on a scale of 1 to 10, where 1 indicates no discomfort at all and 10 indicates the most discomfort you could imagine?”

- Collect saliva sample #2 and record time in chart.

|  | **relative time** | **actual time** | **flare size (cm x cm)** |
| --- | --- | --- | --- |
| TSST start | 0 |  | NA |
| TSST end | +15 |  | NA |
| saliva sample #3 | **+15** |  | NA |
| apply capsaicin | +18 |  | NA |
| saliva sample #4 | **+25** |  | NA |
| flare measurement #1 | +28 |  |  |
| saliva sample #5 | **+35** |  | NA |
| flare measurement #2 | +38 |  |  |
| saliva sample #6 | **+45** |  | NA |
| flare measurement #3 | +48 |  |  |
| blister fluid removal | +50 |  |  |

Experimenter ____________________ Date______________ Time #1

TSST committee:

**TSST Task:**

- Escort participant to the TSST room.

Introduction**:** “Please imagine that this is a job interview. We want you to apply for your ideal job. Please imagine that you really want this job. We will give you 5 minutes to convince these two people why you and nobody else should get the job. Explain why you are the perfect person for this position. In order to convince the committee you will give an impromptu speech for 5 minutes. Please also keep in mind that these two people are Psychologists, well-trained in interpreting body language. Notice the video camera and the microphone - the whole interview will be video taped for later analysis. There will also be a second task. The committee will inform you about this at a later stage. You will now have 3 minutes to prepare your speech. However - you won’t be allowed to use your notes during the speech. Do you have any questions?”

**Experiment Room Phase II:**

- Greet the participant as they leave the TSST room and escort them back to the experiment room. Collect saliva sample #3.
- Apply capsaicin to the skin, record time: "Now I'll apply the capsaicin cream. You won't feel anything for several minutes and then it will start to feel warm and may itch some. You will also notice that you skin will begin to turn red in the area. It will feel like its getting progressively warmer for several minutes and then the feeling will start to taper off. After about 30 minutes, we will wash the cream off and take the fluid from the remaining blisters. During this time, you will collect saliva samples every ten minutes. We will also be measuring the size of the red area on your arm and photographing it periodically. Do you have any questions?"
- Collect saliva samples every 10 minutes post-TSST. Collect flare measurements after each saliva sample.
  - - Measure diameter of the flare at its widest and longest points. Take a digital photograph of the area.
- After the final flare measurement and photograph, have the nurse remove the blister fluid.
- After the blisters have been covered, read the debriefing, give the participant care instructions and MD contact information. Answer any questions.
- Escort the participant to the exit.

Experimenter ____________________ Date______________ Time #1

TSST committee:

**Data saving and Clean-up:**

1. Put saliva samples and blister fluid samples in -80 freezer
2. Download the digital photos to the network
   1. [insert instructions here]
3. Sterilize blister template
   1. [insert instructions here]
4. Breakdown experiment room and TSST room.
   1. Put video camera and microphone away.
   2. Put experiment room equipment away
   3. Wipe down table with disinfectant.

TSST Committee Member Instructions – Visit #1

# Protocol #: 2005-0356

**TSST & Capsaicin Challenge**

TSST Visit #1

Subject #: ____________ Date: ___________________

TSST begin (time): _____ Committee: ______________

- Start the stop watch when the experimenter leaves the room. After 3 mintues is up: **“Your time is up, please leave your notes on the table and come to the microphone. You may begin.”**
- Start video camera recording.
- When 5 minutes has elapsed: **“Your time is up, you can stop now.”**
  - PAUSE THE STOPWATCH!!

| 1. Did the subject speak for the entire 5 minutes? | **YES** | **NO** |  |
| --- | --- | --- | --- |
| 2. Did you ask them questions? | **YES** | **NO** |  |
| 3. Did you have the impression that the subject was stressed? | **YES** | **NO** |  |
| 4. Any other comments? | **YES** | **NO** |  |

**Questions:**

- What is your position on the death penalty?
- What is your position on abortion?
- What do your friends like most about you?
- What do you think of team work?
- Do you feel capable of leading a team? (Why? / Why not?)
- What are your weaknesses?
- What do you think of Psychologists?
- Please complete the following sentence: I am the best at……
- Are you vain?
- Do you think you will make it to the top?

## Mental Math Task

- Instructions: **“Next we are going to test your ability to concentrate. We would like you to count backwards in increments of 17 from the number 2043. If you make a mistake, I’ll let you know and you have to begin again at 2043. Please begin.”**
- START THE STOPWATCH!!
- If they make a mistake: **“That is incorrect, please start over at 2043.”**

## TSST: 2043 minus

| 2043 | 1278 | 513 |
| --- | --- | --- |
| 2026 | 1261 | 496 |
| 2009 | 1244 | 479 |
| 1992 | 1227 | 462 |
| 1975 | 1210 | 445 |
| 1958 | 1193 | 428 |
| 1941 | 1176 | 411 |
| 1924 | 1159 | 394 |
| 1907 | 1142 | 377 |
| 1890 | 1125 | 360 |
| 1873 | 1108 | 343 |
| 1856 | 1091 | 326 |
| 1839 | 1074 | 309 |
| 1822 | 1057 | 292 |
| 1805 | 1040 | 275 |
| 1788 | 1023 | 258 |
| 1771 | 1006 | 241 |
| 1754 | 989 | 224 |
| 1737 | 972 | 207 |
| 1720 | 955 | 190 |
| 1703 | 938 | 173 |
| 1686 | 921 | 156 |
| 1669 | 904 | 139 |
| 1652 | 887 | 122 |
| 1635 | 870 | 105 |
| 1618 | 853 | 88 |
| 1601 | 836 | 71 |
| 1584 | 819 | 54 |
| 1567 | 802 | 37 |
| 1550 | 785 | 20 |
| 1533 | 768 | 3 |
| 1516 | 751 |  |
| 1499 | 734 |  |
| 1482 | 717 |  |
| 1465 | 700 |  |
| 1448 | 683 |  |
| 1431 | 666 |  |
| 1414 | 649 |  |
| 1397 | 632 |  |
| 1380 | 615 |  |
| 1363 | 598 |  |
| 1346 | 581 |  |
| 1329 | 564 |  |
| 1312 | 547 |  |
| 1295 | 530 |  |

- After 5 minutes is up: **“Time is up, you can stop now. You can go now. Please pick up your notes and meet [experimenter] in the hallway.”**
- Stop video camera recording.

| **Try** | **Amount of correct counting steps** | **Last correct number** |
| --- | --- | --- |
| 1^st^ try |  |  |
| 2^nd^ try |  |  |
| 3^rd^ try |  |  |
| 4^th^ try |  |  |
| 5^th^ try |  |  |
| 6^th^ try |  |  |
| 7^th^ try |  |  |
| 8^th^ try |  |  |
| 9^th^ try |  |  |
| 10^th^ try |  |  |
| 11^th^ try |  |  |
| 12^th^ try |  |  |
| 13^th^ try |  |  |
| 14^th^ try |  |  |
| 15^th^ try |  |  |
| 16^th^ try |  |  |
| 17^th^ try |  |  |
| 18^th^ try |  |  |
| 19^th^ try |  |  |
| 20^th^ try |  |  |

**Comments:**

TSST Committee Member Instructions – Visit #2

# Protocol #: 2005-0356

**TSST & Capsaicin Challenge**

TSST Visit #2

Subject #: ____________ Date: ___________________

TSST begin (time): _____ Committee: ______________

- Start the stop watch when the experimenter leaves the room. After 3 mintues is up: **“Your time is up, please leave your notes on the table and come to the microphone. You may begin.”**
- Start video camera recording.
- When 5 minutes has elapsed: **“Your time is up, you can stop now.”**
  - PAUSE THE STOPWATCH!!

| 1. Did the subject speak for the entire 5 minutes? | **YES** | **NO** |  |
| --- | --- | --- | --- |
| 2. Did you ask them questions? | **YES** | **NO** |  |
| 3. Did you have the impression that the subject was stressed? | **YES** | **NO** |  |
| 4. Any other comments? | **YES** | **NO** |  |

**Questions:**

- What is your position on the death penalty?
- What is your position on abortion?
- What do your friends like most about you?
- What do you think of team work?
- Do you feel capable of leading a team? (Why? / Why not?)
- What are your weaknesses?
- What do you think of Psychologists?
- Please complete the following sentence: I am the best at……
- Are you vain?
- Do you think you will make it to the top?

## Mental Math Task

- Instructions: **“Next we are going to test your ability to concentrate. First, we want you to add 3 to a given number. Then multiply your answer by 2. Next add 3 to that resulting number and then multiply it by 2 again. Please verbally report your answer at each step. Continue with this pattern until I tell you to stop. If you make a mistake, I will let you know and you’ll have to begin again at the starting number. You will start with the number 5. Please begin.”**
- START THE STOPWATCH!!
- If they make a mistake: **“That is incorrect, please start over at 5.”**

| 5 + 3 = **8** | 8 x 2 = **16** |
| --- | --- |
| 16 + 3 = **19** | 19 x 2 = **38** |
| 38 + 3 = **41** | 41 x 2 = **82** |
| 82 + 3 = **85** | 85 x 2 = **170** |
| 170 + 3 = **173** | 173 x 2 = **346** |
| 346 + 3 = **349** | 349 x 2 = **698** |
| 698 + 3 = **701** | 701 x 2 = **1402** |
| 1402 + 3 = **1405** | 1405 x 2 = **2810** |
| 2810 + 3 = **2813** | 2813 x 2 = **5626** |
| 5626 + 3 = **2629** | 5629 x 2 = **11,258** |
| 11,258 + 3 = **11,261** | 11,261 x 2 = **22,522** |
| 22,522 + 3 = **22,525** | 22,525 x 2 = **45,050** |
| 45,050 + 3 = **45,053** | 45,053 x 2 = **90,106** |
| 90,106 + 3 = **90,109** | 90,109 x 2 = **180,218** |
| 180,218 + 3 = **180,221** | 180,221 x 2 = **360,442** |
| 360,442 + 3 = **360,445** | 360,445 x 2 = **720,890** |
| 720,890 + 3 = **720,893** | 720,893 x 2 = **1,441,786** |
| 1,441,786 + 3 = **1,441,789** | 1,441,789 x 2 = **2,883,578** |

- After 5 minutes is up: **“Time is up, you can stop now. You can go now. Please pick up your notes and meet [experimenter] in the hallway.”**
- Stop video camera recording.

| **Try** | **Amount of correct counting steps** | **Last correct number** |
| --- | --- | --- |
| 1^st^ try |  |  |
| 2^nd^ try |  |  |
| 3^rd^ try |  |  |
| 4^th^ try |  |  |
| 5^th^ try |  |  |
| 6^th^ try |  |  |
| 7^th^ try |  |  |
| 8^th^ try |  |  |
| 9^th^ try |  |  |
| 10^th^ try |  |  |
| 11^th^ try |  |  |
| 12^th^ try |  |  |
| 13^th^ try |  |  |
| 14^th^ try |  |  |
| 15^th^ try |  |  |
| 16^th^ try |  |  |
| 17^th^ try |  |  |
| 18^th^ try |  |  |
| 19^th^ try |  |  |
| 20^th^ try |  |  |

**Comments:**

TSST Committee Member Instructions – Visit #3

# Protocol #: 2005-0356

**TSST & Capsaicin Challenge**

TSST Visit #3

Subject #: ____________ Date: ___________________

TSST begin (time): _____ Committee: ______________

- Start the stop watch when the experimenter leaves the room. After 3 mintues is up: **“Your time is up, please leave your notes on the table and come to the microphone. You may begin.”**
- Start video camera recording.
- When 5 minutes has elapsed: **“Your time is up, you can stop now.”**
  - PAUSE THE STOPWATCH!!

| 1. Did the subject speak for the entire 5 minutes? | **YES** | **NO** |  |
| --- | --- | --- | --- |
| 2. Did you ask them questions? | **YES** | **NO** |  |
| 3. Did you have the impression that the subject was stressed? | **YES** | **NO** |  |
| 4. Any other comments? | **YES** | **NO** |  |

**Questions:**

- What is your position on the death penalty?
- What is your position on abortion?
- What do your friends like most about you?
- What do you think of team work?
- Do you feel capable of leading a team? (Why? / Why not?)
- What are your weaknesses?
- What do you think of Psychologists?
- Please complete the following sentence: I am the best at……
- Are you vain?
- Do you think you will make it to the top?

## Mental Math Task

- Instructions: **“Next we are going to test your ability to concentrate. We would like you to count backwards in increments of 14 from the number 2077. If you make a mistake, I’ll let you know and you have to begin again at 2077. Please begin.”**
- START THE STOPWATCH!!
- If they make a mistake: **“That is incorrect, please start over at 2077.”**

## TSST: 2077 minus 14

| 2077 | 1447 | 817 | 187 |
| --- | --- | --- | --- |
| 2063 | 1433 | 803 | 173 |
| 2049 | 1419 | 789 | 159 |
| 2035 | 1405 | 775 | 145 |
| 2021 | 1391 | 761 | 131 |
| 2007 | 1377 | 747 | 117 |
| 1993 | 1363 | 733 | 103 |
| 1979 | 1349 | 719 | 89 |
| 1965 | 1335 | 705 | 75 |
| 1951 | 1321 | 691 | 61 |
| 1937 | 1307 | 677 | 47 |
| 1923 | 1293 | 663 | 33 |
| 1909 | 1279 | 649 | 19 |
| 1895 | 1265 | 635 | 5 |
| 1881 | 1251 | 621 |  |
| 1867 | 1237 | 607 |  |
| 1853 | 1223 | 593 |  |
| 1839 | 1209 | 579 |  |
| 1825 | 1195 | 565 |  |
| 1811 | 1181 | 551 |  |
| 1797 | 1167 | 537 |  |
| 1783 | 1153 | 523 |  |
| 1769 | 1139 | 509 |  |
| 1755 | 1125 | 495 |  |
| 1741 | 1111 | 481 |  |
| 1727 | 1097 | 467 |  |
| 1713 | 1083 | 453 |  |
| 1699 | 1069 | 439 |  |
| 1685 | 1055 | 425 |  |
| 1671 | 1041 | 411 |  |
| 1657 | 1027 | 397 |  |
| 1643 | 1013 | 383 |  |
| 1629 | 999 | 369 |  |
| 1615 | 985 | 355 |  |
| 1601 | 971 | 341 |  |
| 1587 | 957 | 327 |  |
| 1573 | 943 | 313 |  |
| 1559 | 929 | 299 |  |
| 1545 | 915 | 285 |  |
| 1531 | 901 | 271 |  |
| 1517 | 887 | 257 |  |
| 1503 | 873 | 243 |  |
| 1489 | 859 | 229 |  |
| 1475 | 845 | 215 |  |
| 1461 | 831 | 201 |  |

- After 5 minutes is up: **“Time is up, you can stop now. You can go now. Please pick up your notes and meet [experimenter] in the hallway.”**
- Stop video camera recording.

| **Try** | **Amount of correct counting steps** | **Last correct number** |
| --- | --- | --- |
| 1^st^ try |  |  |
| 2^nd^ try |  |  |
| 3^rd^ try |  |  |
| 4^th^ try |  |  |
| 5^th^ try |  |  |
| 6^th^ try |  |  |
| 7^th^ try |  |  |
| 8^th^ try |  |  |
| 9^th^ try |  |  |
| 10^th^ try |  |  |
| 11^th^ try |  |  |
| 12^th^ try |  |  |
| 13^th^ try |  |  |
| 14^th^ try |  |  |
| 15^th^ try |  |  |
| 16^th^ try |  |  |
| 17^th^ try |  |  |
| 18^th^ try |  |  |
| 19^th^ try |  |  |
| 20^th^ try |  |  |

**Comments:**

Questionnaires Runlog

## Questionnaires Runlog

Logsheet

Subject ID:____________ Session (circle one): 1=Aug/Sep; 2=Nov/Dec; 3=Feb/Mar, 2007

Date: _______________ Room #: ________ Experimenter(s):________________

Notes: _________________________________________________________________

_______________________________________________________________________

_______________________________________________________________________

_______________________________________________________________________

_______________________________________________________________________

_______________________________________________________________________

_______________________________________________________________________

_______________________________________________________________________

_______________________________________________________________________

_______________________________________________________________________

_______________________________________________________________________

_______________________________________________________________________

_______________________________________________________________________

_______________________________________________________________________

Questionnaires

Participants will always complete questionnaires AFTER the behavioral tasks (e.g., IAT and CPT)

Setup

Move the computer off of the table.

Participants can sit at the center of the table.

Put participant ID # on questionnaire packet that you find in their folder. If packet is not in folder, black behavioral notebook has some extras you can use.

Give them a pen & the packet and ask them to complete all the questionnaires.

After all participants

Take down the “Do not Disturb” sign & put it back

Put completed questionnaire packet in “completed forms” folder

File participant folders back in main file.

Put laptop back in cabinet #10 & return keys

Any questions, just email me (Donal) at [dgmaccoon@wisc.edu](mailto:dgmaccoon@students.wisc.edu). 263-1968 (office) or 240-9950 (home). Thanks!

#### Chapter 4

Handout Timeline

**Handout Timeline**

Participant Handouts

Clinic Visit Handouts

| **Title** | Pre-  Info | Info | V1 | V2 | V3 | V4 | V5 | V6 | V7 | V8 | V9 | V10 |
| --- | --- | --- | --- | --- | --- | --- | --- | --- | --- | --- | --- | --- |
| Information Packet | 🟆 |  |  |  |  |  |  |  |  |  |  |  |
| Physician Authorization Form |  | 🟆 |  |  |  |  |  |  |  |  |  |  |
| Medical History/ Physical Activity History |  | 🟆 |  |  |  |  |  |  |  |  |  |  |
| Registration form |  | 🟆 |  |  |  |  |  |  |  |  |  |  |
| Cortisol Kit #1 |  |  | 🟆 |  |  |  |  |  |  |  |  |  |
| Cortisol Kit #3 |  |  |  |  |  |  |  |  | 🟆 |  |  |  |
| Saliva Sampling Instructions |  | 🟆 | 🟆 |  |  | 🟆 |  | 🟆 |  |  |  |  |
| Payment form |  |  | 🟆 |  |  | 🟆 |  |  | 🟆 |  |  | 🟆 |
| New Medication Questionnaire |  |  | 🟆 | 🟆 | 🟆 | 🟆 | 🟆 | 🟆 | 🟆 | 🟆 | 🟆 | 🟆 |
| Big Five Inventory (BFI) |  |  |  | 🟆 |  |  | 🟆 |  |  | 🟆 |  |  |
| DPES (compassion, contentment, & interest) |  |  |  | 🟆 |  |  | 🟆 |  |  | 🟆 |  |  |
| Hand Measurement |  |  |  | 🟆 |  |  | 🟆 |  |  | 🟆 |  |  |
| Index of Clinical Stress (ICS) **[cut]** |  |  |  | 🟆 |  |  | 🟆 |  |  | 🟆 |  |  |
| Five Factor Mindfulness Scale (5-M) [**cut]** |  |  |  | 🟆 |  |  | 🟆 |  |  | 🟆 |  |  |
| Medical Symptom Checklist (MSCL) |  |  |  | 🟆 |  |  | 🟆 |  |  | 🟆 |  |  |
| Penn State Worry Questionnaire (PSWQ) **[cut]** |  |  |  | 🟆 |  |  | 🟆 |  |  | 🟆 |  |  |
| Perceived Stress Scale (PSS) |  |  |  | 🟆 |  |  | 🟆 |  |  | 🟆 |  |  |
| Ryff well-being (54 items) |  |  |  | 🟆 |  |  | 🟆 |  |  | 🟆 |  |  |
| Satisfaction With Life Scale (subjective well-being) |  |  |  | 🟆 |  |  | 🟆 |  |  | 🟆 |  |  |
| Symptom Checklist-90-R (SCL-90-R) |  |  |  | 🟆 |  |  | 🟆 |  |  | 🟆 |  |  |
| STAI (Y-2, Trait) **[cut]** |  |  |  | 🟆 |  |  | 🟆 |  |  | 🟆 |  |  |
| Stress reactive rumination scale (SRRS) |  |  |  | 🟆 |  |  | 🟆 |  |  | 🟆 |  |  |
| StressPoints |  |  |  | 🟆 |  |  | 🟆 |  |  | 🟆 |  |  |
| Interpersonal Reactivity Index (IRI) |  |  |  | 🟆 |  |  | 🟆 |  |  | 🟆 |  |  |
| Reactivity to Affective Stimuli Questionnaire-11 Item (RASQ-11) |  |  |  | 🟆 |  |  | 🟆 |  |  | 🟆 |  |  |
| PANAS – general (taken at fmri) |  |  |  | 🟆 | 🟆 |  | 🟆 | 🟆 |  | 🟆 | 🟆 |  |
| PANAS – NOW (taken before and after fmri) |  |  |  | 🟆 | 🟆 |  | 🟆 | 🟆 |  | 🟆 | 🟆 |  |
| Balanced Emotional Empathy Scale (BEES) **[cut]** |  |  |  |  | 🟆 |  |  | 🟆 |  |  | 🟆 |  |
| Marlowe–Crowne Social Desirability Scale (MCSDS) **[cut]** |  |  |  |  | 🟆 |  |  | 🟆 |  |  | 🟆 |  |
| Participant Stimulation Debriefing |  |  |  |  | 🟆 |  |  | 🟆 |  |  | 🟆 |  |
| Blood Draw Questionnaire |  |  |  |  |  |  |  |  |  |  |  | 🟆 |
| Experience Check during 4-months post-class (v4).doc |  |  |  |  |  |  |  |  |  |  | 🟆 |  |

Class Handouts for Research: Instructors will not see responses to these questionnaires. They will be collected by a researcher.

| **Title** | **C1** | **C2** | **C3** | **C4** | **C5** | **C6** | **All Day** | **C7** | **C8** |
| --- | --- | --- | --- | --- | --- | --- | --- | --- | --- |
| Group Climate Questionnaire (R3) |  |  | 🟆 |  |  |  |  |  | 🟆 |
| 4-month practice log (HEP & MBSR;R4) |  |  |  |  |  |  |  |  | 🟆 |
| WAI client (short form;R2) |  |  | 🟆 |  |  |  |  |  | 🟆 |
| Home Assignments Log (HEP & MBSR, R1) | 🟆 | 🟆 | 🟆 | 🟆 | 🟆 | 🟆 |  | 🟆 |  |
| Body Scan Survey (MBSR Only, R5) | 🟆 | 🟆 | 🟆 | 🟆 | 🟆 | 🟆 |  | 🟆 | 🟆 |
| Blood Draw Questionnaire (given by nurses) | 🟆 |  |  |  |  |  |  |  | 🟆 |
| Saliva Sampling Instructions (given by nurses) |  |  |  |  |  |  |  |  | 🟆 |
| Cortisol Kit #2 (given by nurses) |  |  |  |  |  |  |  |  | 🟆 |

Instructor Handouts for Research

| **Title** | **C1** | **C2** | **C3** | **C4** | **C5** | **C6** | **All Day** | **C7** | **C8** |
| --- | --- | --- | --- | --- | --- | --- | --- | --- | --- |
| Post-Class Teacher Questionnaire (T1) | 🟆 | 🟆 | 🟆 | 🟆 | 🟆 | 🟆 | 🟆 | 🟆 | 🟆 |
| Class makeup Form (T2) | 🟆 | 🟆 | 🟆 | 🟆 | 🟆 | 🟆 | 🟆 | 🟆 | 🟆 |
| WAI teacher (short form; T3) |  |  | 🟆 |  |  |  |  |  | 🟆 |
| Group Climate Questionnaire (T4) |  |  | 🟆 |  |  |  |  |  | 🟆 |

#### Appendix

Additional Forms

**Additional Forms/Information**

Recruitment E-Mail (v6/12/06)

| **RECRUITMENT E-MAIL**: Use the following e-mail in response to initial e-mail inquiries that DO NOT CONTAIN a phone number at which to contact the individual. |
| --- |

Dear Potential Well-Being Study Participant,

We recently received an e-mail from you expressing your interest in participating in our study. UW Health Integrative Medicine has agreed to let us conduct a study on individuals interested in participating in Mindfulness-Based Stress Reduction (MBSR) or a Health Enhancement Program (HEP) this Fall. The goal of the study is to evaluate the effects of these two programs on pain regulation, negative affect, attention, and social stress.

If you think you are interested in participating in our study please call us at (608)-262-9888 and leave a voicemail message including your name, e-mail address, street address, phone number, and best times to call you or send us an e-mail including your name, street address, phone number, and best times to call you. Please be sure to include your phone number so that we may contact you by phone to go over the details of the study, ask you a few questions, and answer any questions that you may have.

You can expect to receive a phone call from us within the next two weeks. During this phone call we will ask you a number of questions and answer any questions you might have about the study. Once you have left a voicemail message with your contact information, please wait at least two weeks to call again. We appreciate your patience.

Thank you for your inquiry.

Sincerely,

The Well-Being Study Team

| **ABRIDGED E-MAIL**: Use the following e-mail in response to initial e-mail inquiries that DO CONTAIN a phone number at which to contact the individual. |
| --- |

Dear Potential Well-Being Study Participant,

 We recently received an e-mail from you expressing your interest in participating in our study.   UW Health Integrative Medicine has agreed to let us conduct a study on individuals interested in participating in Mindfulness-Based Stress Reduction (MBSR) or a Health Enhancement Program (HEP) this Fall.  The goal of the study is to evaluate the effects of these two programs on pain regulation, negative affect, attention, and social stress.

You can expect to receive a phone call from us at the number that you provided within the next two weeks. During this phone call we will ask you a number of questions and answer any questions you might have about the study. We appreciate your patience.

 Thank you for your inquiry.

 Sincerely,

 The Well-Being Study Team

| **THANK YOU E-MAIL SENT**: Use the following e-mail in response to e-mails CONTAINING a phone number responding to our request for more contact information |
| --- |

Dear Potential Well-Being Study Participant,

Thank you for your e-mail response containing contact information.

You can expect to receive a phone call from us at the number that you provided within the next two weeks. During this phone call we will ask you a number of questions and answer any questions you might have about the study. We appreciate your patience.

 Thank you for your inquiry.

 Sincerely,

 The Well-Being Study Team


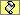


| **REJECTION E-MAIL SENT**: Use the following e-mail in response to e-mails containing information that would automatically exclude him/her for participating in the study (i.e., over the age of 65). |
| --- |

Hello,

 We recently received an e-mail from you expressing your interest in participating in our study.

 We thank you for your interest; however, based on our exclusion criteria, you do not qualify for the study.  We will destroy any information we currently have on you so you can be assured of confidentiality.  I would like to wish you the best during any classes you decide to take at UW Health.

 Sincerely,

 The Well-Being Study Team

Information Packet

Dear Potential Well-Being Study Participant,

During our phone conversation on __________ we agreed to send you more information about participation in the University of Wisconsin’s Well-Being Study.

As a reminder, you are scheduled to attend an information session on ___________ from 6:30-8:30pm. The information session will be held at UW’s Integrative Medicine Center in room #________, which is located at:

- Address: Research Park Clinic
   621 Science Dr.
   Madison, WI   53711

Phone Number: 608-265-8303

In this packet we have included a map to help direct you to the Integrative Medicine Center as well as brief explanations of the two programs included in the study, the Health Enhancement Program (HEP) and the Mindfulness Based Stress Reduction (MBSR) program. During the information session you will have the opportunity to ask questions about both programs as well as the study overall.

In addition, at the information session, should you decide to participate in the study, we will schedule your first 3 clinic visits. These will occur between ________ and _______, 2006. Currently we have a fair degree of flexibility with times and days for clinic visits, but later we will not. For this reason it is of ***utmost importance*** that you bring your calendar to the information session. That way we will be able to schedule times that work best for you and be certain that we can count on your attendance.

Should you have any questions before the information session please feel free to contact Jenna Sheftel, the study coordinator, at **(608)** **262-9888** or via e-mail (MBSRstudy@gmail.com). We look forward to seeing you at the information session.

Sincerely,

The Well-Being Study Team

Lab for Affective Neuroscience

University of Wisconsin

INTEGRATIVE MEDICINE CENTER

- Address: Research Park Clinic
   621 Science Dr.
   Madison, WI   53711

Phone Number: 608-265-8303

- Map:


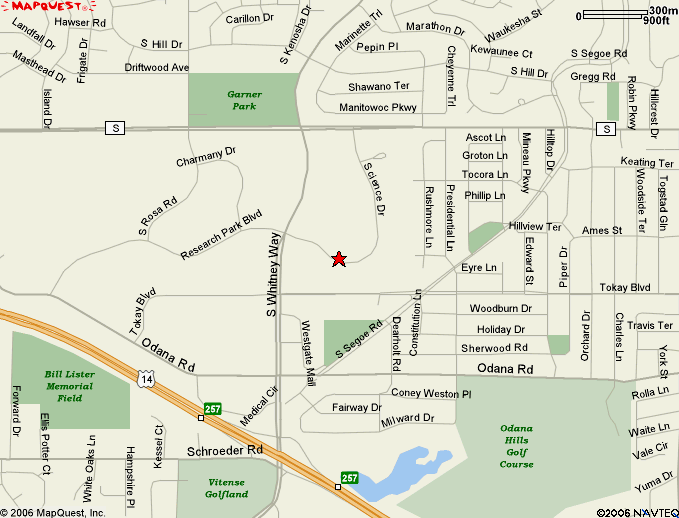


**Health Enhancement Program**

The class will focus four modules for improving well-being. These four elements are physical activity, nutrition, functional movement, and guided imagery.

1. Physical Activity Instructor: Jude Sullivan, M.S.

In this unit participants will engage in consistent rhythmic movement that can be accomplished in a single session per day or aggregated over multiple sessions. The goal is to work towards the completing up to 30 minutes daily for 6 days per week. Activities include walking, or any similar activity preferred by the participant, complemented by and passive, static (non-moving) stretching. The effort expended while engaging in the rhythmic activity is not to exceed what participants would self-judge to be between “fairly light” and “somewhat hard”.

1. Nutrition Instructor: Julie Thurlow, DrPH, RD, LD

Optimal nutrition is not only the provision of nutrients to prevent deficiencies but also provision of amounts of nutrients and other dietary components/constituents to provide optimal health and decrease the risk of the development of chronic disease. Current best evidence indicates that the protective effects of food come from a dietary pattern that provides foods in appropriate types and amounts, and not supplements of specific nutrients and/or food components. Participants will learn what the current recommended dietary pattern is for their specific age, gender and activity as well as ways of identifying the strengths and weakness of their current diet and how to effect change if necessary.

1. Functional Movement Instructor: Jude Sullivan, M.S.

People of all ages and levels of physical ability are innately dependent on the ability to move. However, it is observed that as we age lifestyle choices we make reduce our ability to take advantage of the infinite manner in which we can move and use our bodies. Functional movement training will help improve*:*

🞛 Balance 🞛 Core Stability 🞛 Agility 🞛 Mobility 🞛

Specifically, we address quality posture and alignment. Posture and alignment are the keys to creating optimal function. In addition, functional movement focuses on proprioception, defined as a sensory modality that includes sensation of joint movement and joint position sense. As a result, the goal of the overall training plan is to expand one’s movement repertoire in order to become more physically adaptable and “free.” This freedom opens doors to choose more activities to participate in as well as supply one with the necessary tools to be ready to adjust reactively as one needs (e.g. when tripping on a curb or slipping on a patch of ice).

1. Music Therapy Instructor: Pamela Polcyn

The American Music Therapy Association defines music therapy as “an established healthcare profession that uses music to address physical, emotional, cognitive, and social needs of individuals of all ages. Music therapy interventions can be designed to promote wellness, manage stress, alleviate pain, express feelings, enhance memory, improve communication and promote physical rehabilitation.

Music therapy can be used for stress reduction using active music making, music and relaxation with guided imagery, and songwriting. Exploring a variety of music interventions with class participants will maximize the class’ impact for a group with diverse musical and cultural backgrounds as well as physical and emotional needs.

**HEP All-Day Format:**

| 9:00-9:15 | Welcome, Introduction and Ground Rules |
| --- | --- |
|  |  |
| 9:15-9:45 | Guided Music Therapy Practice |
|  |  |
| 9:45-10:15 | Music Therapy Self-Practice |
|  |  |
| 10:15-10:45 | Group Discussion of Music Therapy Practice |
|  |  |
| 10:45-11:15 | Nutrition Review |
|  |  |
| 11:15-11:45 | Group Discussion of Nutrition Review |
|  |  |
| 11:45-12:45 | Lunch |
|  |  |
| 12:45-1:15 | Walking Activity |
|  |  |
| 1:15-1:45 | Stretch and Mobility Movement Activity |
|  |  |
| 1:45-2:15 | Group Discussion of Walking & Stretching Activity |
|  |  |
| 2:15-2:45 | Guided Functional Movement Practice |
|  |  |
| 2:45-3:15 | Functional Movement Self-Practice |
|  |  |
| 3:15-3:45 | Group Discussion of Functional Movement |
|  |  |
| 3:45-4:00 | Wrap Up of Days Events |

**Mindfulness Based Stress Reduction**

Two current MBSR instructors for the Mindfulness Center at Integrative Medicine will teach the MBSR class. They are Cindy McCallum and Ms. Katherine Bonus MA. Katherine Bonus is the Manager and Instructor of Mindfulness Based Stress Reduction Program (MBSR) at UW Health Integrative Medicine.

Mindfulness meditation originates from a Buddhist tradition, but in its current formulation requires no particular religious orientation or belief system. It involves systematic training in the development of a sustained, non-aroused state of attention and clear awareness. Mindfulness is a meditation practice that cultivates paying attention in the present moment, bare of judgment, commentary, and decision.

- It trains the recognition of automatic reactivity such as habituated patterns of behavior, emotional reactivity, momentary distractions and loss of attention.
- It cultivates awareness, stability, insight and choice.
- Mindfulness includes working with how one relates to environmental stimuli, one’s sensitivity to bodily sensations, breathing, states of mind (thinking and emotions), and habitual patterns of automatic reactivity.
- MBSR training includes the cultivation of attitudes of self-appreciation, compassion and empathy.
- Audiotapes or compact discs of varying length but no longer than 45 min with guided instruction are initially provided that guide the participants in their formal meditation practice of the
  - body scan,
  - sitting meditation,
  - gentle yoga.

- Participants will be requested to practice for 6 days per week at home. Participants are asked to select a daily practice time best for them and to record on a log sheet what they practiced and how long.

The classes will normally involve a review of previously assigned homework, including a discussion of difficulties participants may have experienced, as well as introduction of new material, and hands-on practice/experience with new techniques or skills. The 8-week schedule is outlined below.

Weeks 1 - 2: Practice of a body scan technique and an introduction to sitting with awareness of breathing

Weeks 3 – 4: Alternate body scan with gentle yoga practice. Time spent sitting with awareness of breathing increases

Weeks 5 - 6: Begin walking meditation practice. Sitting exercises continue.

.

Week 7: Continued practice with technique of preference. .

Week 8: Perform a body scan at least twice this week. Continue sitting and yoga. Provision of guided imagery tapes from Dr. Kabat-Zinn’s collection of 5 tapes/compact discs.

**MBSR All-Day Format:**

| 9:00 - 9:30 | (30 min) | Welcome Overview of Day & Intro. |
| --- | --- | --- |
|  |  |  |
| 9:30 – 10:30 | (1 hour) | Yoga |
|  |  |  |
| 10:30 – 10:40 | (15 min) | Sitting |
|  |  |  |
| 10:40 – 11:00 | (20 min) | Walking |
|  |  |  |
| 11:00 – 11:30 | (30 min) | Sitting |
|  |  |  |
| 11:30 – 12:00 | (30 min) | Walking |
|  |  |  |
| 12:00 – 1:00 | (1 hour) | Lunch and rest |
|  |  |  |
| 1:00 - 1:15 | (15 min) | Stretch, Walking in a circle |
|  |  |  |
| 1:15 – 2:00 | (45 min) | Mountain meditation or loving kindness forgiveness meditation |
|  |  |  |
| 2:00 – 3:00 | (1 hour) | Fast walking and Laughing meditation |
|  |  |  |
| 3:00 – 4:00 | (1 hour) | Wrap Up: Dialogue and Inquiry about participants’ first hand experience of the day. |

Class Assignment Notification, HEP-cohort1 (v6/23/06)

Dear Well-Being Study Participant,

We would like to thank you for completing the first three clinic visits and confirm that you have been assigned to a class. We have included in this letter important information regarding dates and times of classes for the group to which you have been assigned. You will also find information to direct you to UW Health Integrative Medicine, where the classes will be held. Should you have a conflict with any of the dates or times listed below, please contact us as soon as possible at (608) 262-9888 so that we may re-schedule you if need be, or answer any questions you may have before attending your first class.

**You have been assigned to the Health Enhancement Program (HEP) group.**

Dates/Times of Classes:

HEP classes meet at all of the eight class dates plus an all-day class. The dates and times listed below. It is important that you attend each and every class.

Class 1 Thursday, 8/31/2006 6:00-8:30 pm

Class 2 Thursday, 9/7/2006 6:00-8:30 pm

Class 3 Thursday, 9/14/2006 6:00- 8:30 pm

Class 4 Thursday, 9/21/2006 6:00-8:30 pm

Class 5 Thursday, 9/28/2006 6:00-8:30 pm

Class 6 Thursday, 10/5/2006 6:00-8:30 pm

**All-Day Session Saturday, 10/7/2006 9:00am-4:00pm**

Class 7 Thursday, 10/12/2006 6:00-8:30 pm

Class 8 Thursday, 10/19/2006 6:00-8:30 pm

First Day of Class

- You have been scheduled for a blood draw on **Thursday, 8/31/2006** at ___________ pm before class begins. This will occur at the UW Health Integrative Medicine Center in **Room # 2386 on the 2^nd^ floor**. We would appreciate it if you would please arrive on time for this blood draw.
- Class #1 will be held in #1111A.
  - The proper attire for this class is comfortable, light weight clothing that is appropriate for exercising and will not constrain movement. The emphasis is on comfort, not style.

Map to UW Health Integrative Medicine:

INTEGRATIVE MEDICINE CENTER

- Address: Research Park Clinic
   621 Science Dr.
   Madison, WI   53711

Phone Number: 608-265-8303

- Map:


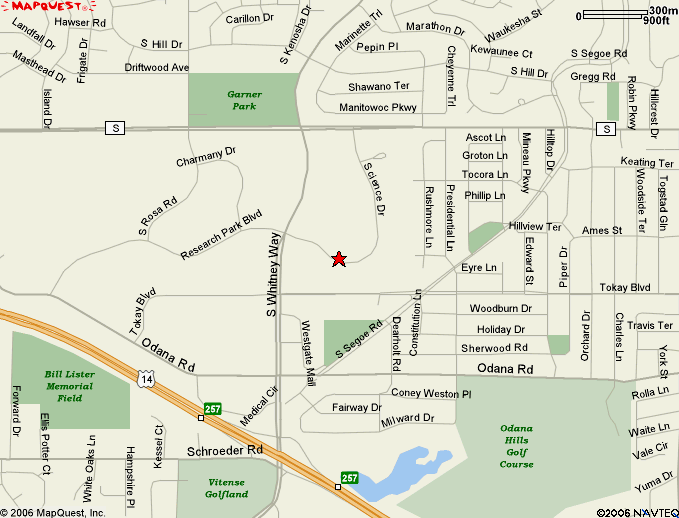


Should you have questions regarding any of the above information, or conflicts with any of the dates and times of the classes, please contact Jenna Sheftel, the study coordinator, at (608) 262-9888.

Thank you again for your interest in our study, and we look forward to seeing you at your classes!

Sincerely,

The Well-Being Study Team

Class Assignment Notification, HEP-cohort2 (v6/23/06)

Dear Well-Being Study Participant,

We would like to thank you for completing the first three clinic visits and confirm that you have been assigned to a class. We have included in this letter important information regarding dates and times of classes for the group to which you have been assigned. You will also find information to direct you to UW Health Integrative Medicine, where the classes will be held. Should you have a conflict with any of the dates or times listed below, please contact us as soon as possible at (608) 262-9888 so that we may re-schedule you if need be, or answer any questions you may have before attending your first class.

**You have been assigned to the Health Enhancement Program (HEP) group.**

Dates/Times of Classes:

HEP classes meet at all of the eight class dates plus an all-day class. The dates and times listed below. It is important that you attend each and every class.

Class 1 Monday, 9/25/2006 6:00-8:30 pm

Class 2 Monday, 10/2/2006 6:00-8:30 pm

Class 3 Monday, 10/9/2006 6:00- 8:30 pm

Class 4 Monday, 10/16/2006 6:00-8:30 pm

Class 5 Monday, 10/23/2006 6:00-8:30 pm

Class 6 Monday, 10/30/2006 6:00-8:30 pm

**All-Day Session Saturday, 11/4/2006 9:00am-4:00pm**

Class 7 Monday, 11/6/2006 6:00-8:30 pm

Class 8 Monday, 11/13/2006 6:00-8:30 pm

First Day of Class

- You have been scheduled for a blood draw on Monday, 9/25/2006 at _____________ pm before class begins. This will occur at the UW Health Integrative Medicine Center in _______________________. We would appreciate it if you would please arrive on time for this blood draw.
- Class #1 will be held in #1111A.
  - The proper attire for this class is comfortable, light weight clothing that is appropriate for exercising and will not constrain movement. The emphasis is on comfort, not style.

Map to UW Health Integrative Medicine:

INTEGRATIVE MEDICINE CENTER

- Address: Research Park Clinic
   621 Science Dr.
   Madison, WI   53711

Phone Number: 608-265-8303

- Map:


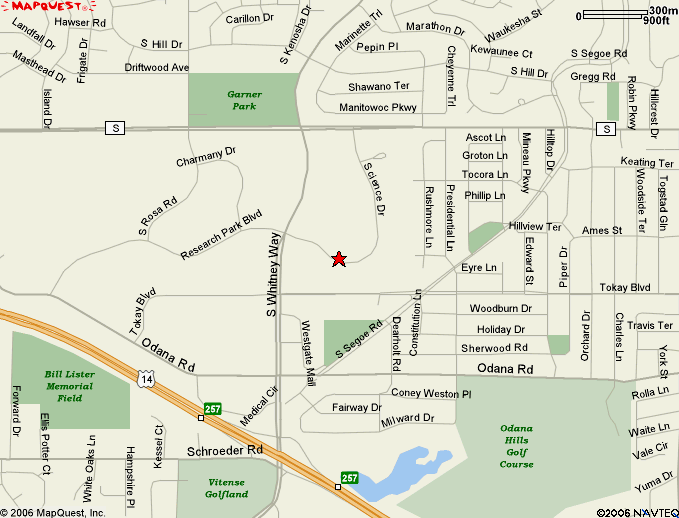


Should you have questions regarding any of the above information, or conflicts with any of the dates and times of the classes, please contact Jenna Sheftel, the study coordinator, at (608) 262-9888.

Thank you again for your interest in our study, and we look forward to seeing you at your classes!

Sincerely,

Class Assignment Notification, MBSR-cohort1 (v6/23/06)

Dear Well-Being Study Participant,

We would like to thank you for completing the first three clinic visits and confirm that you have been assigned to a class. We have included in this letter important information regarding dates and times of classes for the group to which you have been assigned. You will also find information to direct you to UW Health Integrative Medicine, where the classes will be held. Should you have a conflict with any of the dates or times listed below, please contact us as soon as possible at (608) 262-9888 so that we may re-schedule you if need be, or answer any questions you may have before attending your first class.

.

**You have been assigned to the Mindfulness- Based-Stress-Reduction (MBSR) group.**

Dates/Times of Classes:

MBSR classes meet at all of the eight class dates plus an all-day class. The dates and times listed below. It is important that you attend each and every class.

Class 1 Wednesday, 8/30/2006 6:00-8:30 pm

Class 2 Wednesday, 9/6/2006 6:00-8:30 pm

Class 3 Wednesday, 9/13/2006 6:00- 8:30 pm

Class 4 Wednesday, 9/20/2006 6:00-8:30 pm

Class 5 Wednesday, 9/27/2006 6:00-8:30 pm

Class 6 Wednesday, 10/4/2006 6:00-8:30 pm

**All-Day Session Saturday, 10/7/2006 9:00am-4:00pm**

Class 7 Wednesday, 10/11/2006 6:00-8:30 pm

Class 8 Wednesday, 10/18/2006 6:00-8:30 pm

First Day of Class

- You have been scheduled for a blood draw on **Wednesday, 8/30/2006** at _____________ pm before class begins. This will occur at the UW Health Integrative Medicine Center in **Room # 2386 on the 2^nd^ floor**. We would appreciate it if you would please arrive on time for this blood draw.
- Class #1 will be held in #1111A.
  - The proper attire for this class is comfortable, light weight clothing that is appropriate for exercising and will not constrain movement. The emphasis is on comfort, not style.

Map to UW Health Integrative Medicine:

INTEGRATIVE MEDICINE CENTER

- Address: Research Park Clinic
   621 Science Dr.
   Madison, WI   53711

Phone Number: 608-265-8303

- Map:


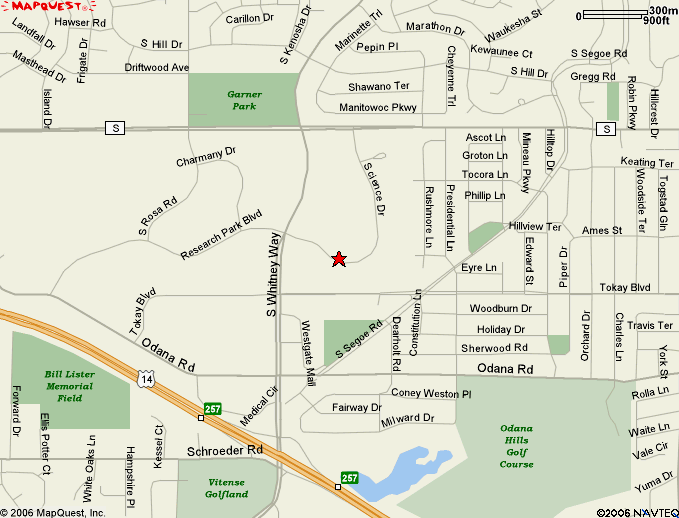


Should you have questions regarding any of the above information, or conflicts with any of the dates and times of the classes, please contact Jenna Sheftel, the study coordinator, at (608) 262-9888.

Thank you again for your interest in our study, and we look forward to seeing you at your classes!

Sincerely,

The Well-Being Study Team

Class Assignment Notification, MBSR-cohort2 (v6/23/06)

Dear Well-Being Study Participant,

We would like to thank you for completing the first three clinic visits and confirm that you have been assigned to a class. We have included in this letter important information regarding dates and times of classes for the group to which you have been assigned. You will also find information to direct you to UW Health Integrative Medicine, where the classes will be held. Should you have a conflict with any of the dates or times listed below, please contact us as soon as possible at (608) 262-9888 so that we may re-schedule you if need be, or answer any questions you may have before attending your first class.

**You have been assigned to the Mindfulness-Based-Stress-Reduction (MBSR) group.**

Dates/Times of Classes:

MBSR classes meet at all of the eight class dates plus an all-day class. The dates and times listed below. It is important that you attend each and every class.

Class 1 Tuesday 9/26/2006 6:00-8:30 pm

Class 2 Tuesday 10/3/2006 6:00-8:30 pm

Class 3 Tuesday 10/10/2006 6:00- 8:30 pm

Class 4 Tuesday 10/17/2006 6:00-8:30 pm

Class 5 Tuesday 10/24/2006 6:00-8:30 pm

Class 6 Tuesday 10/31/2006 6:00-8:30 pm

**All-Day Session Saturday 11/4/2006 9:00am-4:00pm**

Class 7 Tuesday 11/7/2006 6:00-8:30 pm

Class 8 Tuesday 11/14/2006 6:00-8:30 pm

First Day of Class

- You have been scheduled for a blood draw on **Tuesday, 9/26/2006** at _____________ pm before class begins. This will occur at the UW Health Integrative Medicine Center in _______________________. We would appreciate it if you would please arrive on time for this blood draw.
- Class #1 will be held in #1111A.
  - The proper attire for this class is comfortable, light weight clothing that is appropriate for exercising and will not constrain movement. The emphasis is on comfort, not style.

Map to UW Health Integrative Medicine:

INTEGRATIVE MEDICINE CENTER

- Address: Research Park Clinic
   621 Science Dr.
   Madison, WI   53711

Phone Number: 608-265-8303

- Map:


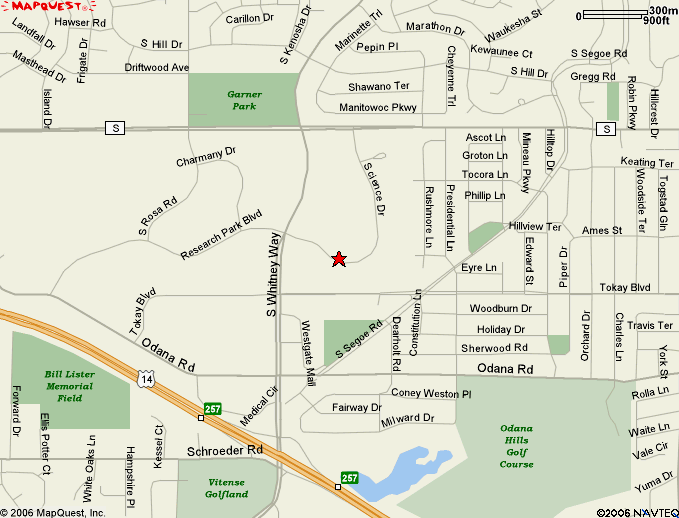


Should you have questions regarding any of the above information, or conflicts with any of the dates and times of the classes, please contact Jenna Sheftel, the study coordinator, at (608) 262-9888.

Thank you again for your interest in our study, and we look forward to seeing you at your classes!

Sincerely,

The Well-Being Study Team

Maps & Directions

WAISMAN CENTER

- Address: 1500 Highland Ave

Madison, WI 53705

- Directions:

*From the east...*
Follow University Avenue to Campus Drive; stay in the right lane and exit at Highland Avenue. Turn right at exit stop sign and continue on Highland to the Waisman Center, approximately 1/2 mile. Enter parking lot just past Nielsen Tennis Stadium, by north tower. (See map of Madison Area).

*From the west...*
Follow University Avenue and turn left at the University Bay Drive/Farley Avenue intersection. Continue over the hill onto Highland Avenue. The Waisman Center is at the bottom of the hill on the left, across the street from UW-Hospital and Clinics. Enter parking lot on north side of the two towers. (Madison Area Map).

- Parking:

Please park and enter the building on the north side of the Waisman Center (see Waisman Center map). Upon request, a parking permit can be acquired.

- Maps:

##### Waisman Center Map

*
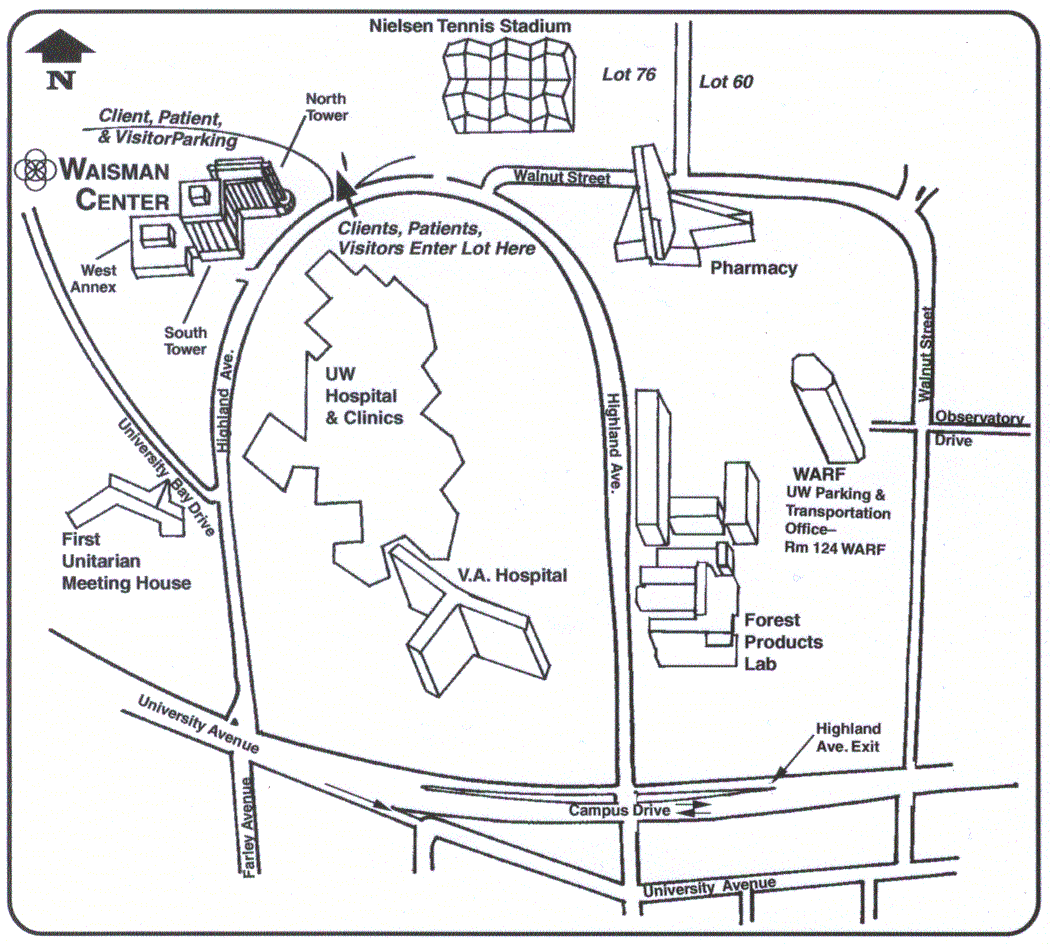
*

##### Map of Madison Area

INTEGRATIVE MEDICINE CENTER

- Address: Research Park Clinic
   621 Science Dr.
   Madison, WI   53711
- Phone Number: 608-265-8303
- Map:


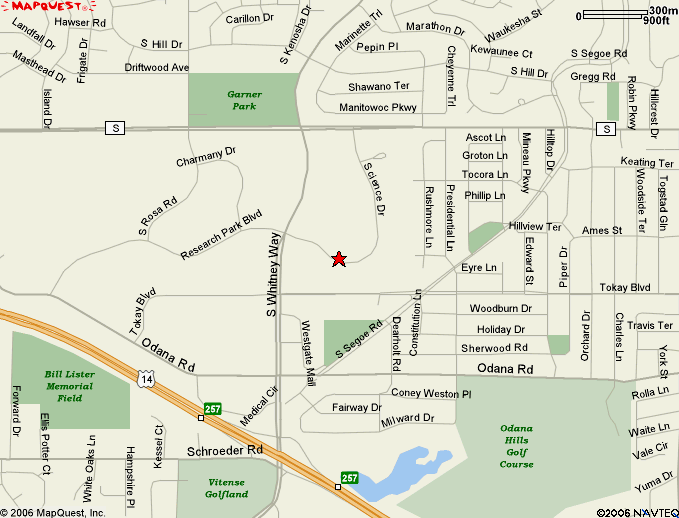


Consent Form

**Subject#**_________ **Protocol # 2005-0356**

**Date:**____________

**RESEARCH PARTICIPANT INFORMATION AND CONSENT FORM**

**Title of Study:** The effects mindfulness training on pain regulation, negative affect, attention, and social stress.

**Study Investigators**: Antoine Lutz, Ph. D., Richard J. Davidson, Ph. D., Donal MacCoon, Ph. D., Katherine Bonus, M.A., Jackie Kuta, Melissa Rosenkranz, Helen Weng.

##### **Invitation**

You are invited to participate in a research study examining two programs designed to improve health and well-being. The first program, the Health-Enhancement Program (HEP), involves physical activity and nutrition programs used in weight management, cardiac rehab and diabetes prevention programs. The second program, Mindfulness-Based-Stress-Reduction (MBSR), uses yoga and meditation techniques. This study will test the impact of these programs on pain regulation, social stress, basic emotion and attention functions, and on the physical factors that affect these processes.

Participating in this study will involve attending one of these two eight-week classes designed to increase your health and well-being. You will be randomly assigned to the class. At the conclusion of the study, you will be invited to participate to the second class should you be interested. This study will also involve having your brain scanned by functional magnetic resonance imaging (fMRI), performing a task involving identifying letters on a computer screen, answering a series of questionnaires, having small blisters formed by suction applied to the underside of your forearm, participating in a stressful social situation, and providing blood and saliva samples. One of the fMRI studies involves the infliction of safe and controlled amounts of pain. If you choose to participate, several moderate heat sensations will be administered on your palm.

Participation in this study is voluntary. The study procedures and the possible risks and benefits are described below. Please read the information carefully and discuss any questions you have before you decide whether or not to join this study.

**Purpose**

This study will find out if HEP and MBSR classes will decrease negative emotions on self-report measures, increase attention on a behavioral task, decrease stress levels on a social stress test, decrease general psychosocial stress as indexed by the salivary cortisol profile through the day, change your immune response, change the aging of your cells, and reveal different neural patterns in response to moderately painful heat and aversive visual stimuli as indexed by functional Magnetic Resonance Imaging (fMRI).

About 52 men and women will participate in this research study at the University of Wisconsin. Your participation will last for about 34 weeks (Testing before your 8-week class, testing immediately following the completion of that class, testing four months after the first class is over). You will be asked to come to the clinic three times for each of the first two testing phases and four times for the third testing phase. Visits last from 15 minutes to 3 hours. Between visits, you will be asked to collect saliva samples and complete practice/diary logs. Over the course of the study, you will be asked to complete three brain scans, three blood draws, 33 saliva samples, three computer tasks, and numerous questionnaires.

## Why are you being asked to participate?

We are looking for about 52 people who are willing to participate in research.

You are being asked to participate because our screening interview indicated that you meet the study’s criteria. Participants will be required to have their physician complete a “Physician Authorization Form” prior to participating in the study (attached). You also indicated in our screening interview that it was realistic for your schedule to attend 10 clinic visits of about 2 hours each as well as attending one 8-week class that meets weekly from 6-8:30 and has an all-day session on a Saturday.

## What does this study consist of?

You will have study visits at three different times during the study: prior to your first training program, immediately after the training program, and four months after the training program. The first two phases will require three clinic visits and the 3^rd^ phase will require four clinic visits for a total of 10 visits over the course of approximately 9 months. At the conclusion of the study, you will be invited to participate in the second class should you be interested. The table below describes what will happen at each visit and about how long the visit will take. Note, that initial paperwork and “mock” MRI scan will only be necessary for visit 1.

|  | Task | Time (mins) |
| --- | --- | --- |
| Visit 1, 4, 7 | Initial paperwork (visit 1 only) | 15 |
|  | Mock scan (visit 1 only) | 15 |
|  | Cortisol kit instructions | 15 |
|  | Computer task | ~70 |
|  | Questionnaires | 5 |
|  | Subtotal (hours): | 2 |
|  |  |  |
| Visit 2, 5, 8 | Introduction and baseline cortisol | 20 |
|  | TSST/capsaicin challenge (mean 60, range 25-90) | 60 |
|  | Cortisol samples/questionnaires | 30 |
|  | Subtotal (mean hours): | 1.83 |
|  |  |  |
| Visit 3, 6, 9 | Scans for both studies | 90 |
|  | Questionnaires | 15 |
|  | Subtotal (hours): | 1.75 |
|  |  |  |
|  | TOTAL per phase (hours): | 5.58 |
|  |  |  |
| Before Class #1 | Blood Draw | 15 |
| After Class #8 | Blood Draw | 15 |
| Visit 10 | Blood Draw | 15 |
|  |  |  |
|  | TOTAL for all 3 phases (hours): | 17.50 |

The following procedures will be done during the study.

- Computer tasks: You may see random letters, digits, adjectives, emotion faces, or negative pictures similar to those described in the Emotion Regulation Task (see below) on a computer screen and asked to respond by pressing a response button to particular stimuli. The tasks will take up to 70 minutes to complete.
- Blood sample collection: About 1 teaspoon of blood may be drawn before, immediately after, and 4-months after your first class. We will count cell types, measure average cellular aging, and see how your cells respond to an anti-inflammatory substance in the test tube.
- MRI simulation: The simulator looks and sounds just like the real MRI scanner. It is used so that you can know how it feels to lie in the scanner. During this time you will also learn the tasks that you will do when you have the real MRI brain scan.
- MRI brain scanning: You will lie on a table and will be slowly moved to the center of a large metal doughnut-shaped magnet. The MRI machine produces a magnetic field that passes through your body without disturbing any of your body parts. It uses a computer to show us what the inside of your body looks like. Radio signals will also be passed through your body. The MRI machine used in your scan has formal approval from the FDA. We will collect several scans each time you are in the machine. You will wear a pair of special goggles through which you will see words or images

Individual scans can last from a few seconds to 20 minutes and you may have to lie still during that time. The entire scanning session may last as long as 1 ½ hours and we will try to keep you comfortable. You will be able to stop the scan at any time. Research staff will be with you during these procedures. During the scans, you will do:

- - Pain Regulation Task: While you are inside the scanner, you will see pictures that indicate you are about to feel something hot. The heat-generating device is permitted for experimental use by the FDA, and has been used safely for almost a decade. It has several features to ensure that the heat produced is safe and well-controlled. If you would like more information on this device, do not hesitate to ask the researchers. The thermal stimuli will start by a 9 to 15 second non painful ramp up heat and then will be either warm or hot for 10 seconds. The temperature of the stimulus will not be greater than 49°C(120°F), which is considered a safe maximum temperature for hot tap water. In addition, your individual pain threshold will be determined and will not be exceeded during the task. You will receive up to 40 bursts of something hot, some of which will be painful and others will not. Following each thermal stimulus you will be asked to rate its painfulness using the same 0-10 scale used in the mock scanner.
  - Emotion Regulation Task: While you are inside the scanner, you will see negative pictures that may cause you to feel some distress. These pictures have been used in many previous research studies. You will also hear descriptions of the pictures through headphones as you are viewing them. While you are in the scanner, your eye activity will be monitored using small cameras and a respiratory belt will be used to measure your respiration.

During the scans, you will wear:

- - Eye Tracking: The goggles have an FDA approved camera inside of them to record your eye movements. The information collected from this camera will be stored in either digital or video format. Looking at your eye movements may help us to understand how you feel about the images or symbols you are looking at. Your name will not be associated with information collected from this camera.
- Saliva (spit) collection: You will be asked to collect saliva samples at some of your clinic visits and on your own at home. From your saliva, we will measure a hormone called cortisol that is related to stress. To collect saliva, you will chew on a piece of cotton and put the cotton in a plastic tube. You will be given supplies to do this.
- Social stress test, blister formation and inflammation:
  - The social stress test we will use is a standard and widely used way of inducing psychological stress. It consists of two 5-minute challenges. During one you will be asked to give a speech. During the other you will be asked to perform a 5-minute calculation task. The entire procedure should last no longer than 2.5 hours.
  - In order to see how stress affects the function of your immune system, we will create a small welt on your forearm. The welt will be created by a cream containing “capsaicin”, which is the substance that makes chili peppers hot. This cream is sold over the counter at most drug stores and is commonly used to treat pain or improve circulation. Applying the cream to your forearm skin will cause a mild to moderate burning or itching sensation and a small area of raised, red skin surrounded by a larger area of redness about 2-4 inches (5-10cm) across. The burning or itching sensations and redness typically last for 30-60 minutes. We will measure the area of skin covered by the redness at various times for 30 minutes after putting the capsaicin on your skin.
  - In order to measure the chemicals in your body that cause the skin response to capsaicin, we will form several small blisters (pea size or smaller) on your forearm. To form the blisters, suction is applied to your skin by a vacuum that slowly causes a blister to form. This process causes very little pain. The time it takes for the blisters to form is different for different people. Typically, it takes between 25 minutes and an hour, but may take as long as 1.5 hours. After the blisters are formed, a nurse will remove the fluid with a very small syringe.
  - These procedures will happen in the following order: first the blisters are formed, then you will do the social stress test, then the capsaicin will be applied.
- Questionnaires: You will fill out approximately 15 questionnaires during various clinic visits that ask you various questions about yourself (e.g., how well a particular statement describes you). Questions include information about psychological symptoms (e.g., anxiety, depression, and stress), medical symptoms, and personality. You will be asked to complete these measures at least three times. Also, you will be asked to complete practice logs daily and food/nutrition diaries.
- Study Treatments: You will be asked to attend two separate eight-week classes designed to enhance your well-being: Health Enhancement Program (HEP) or the Mindfulness-Based Stress Reduction (MBSR). The class you attend first will be randomly assigned. You will be expected to attend all sessions of each class (2 hours and 15 minutes per week for eight weeks, plus one all day session) and practice or complete assignments at home (up to 45 minutes per night). Both classes will involve lecture, group discussion, and various hands-on exercises. Topics, experiences, and exercises may include, light exercise, stretching, meditation, yoga, nutrition, guided imagery, and reflexology.
- If preliminary results are promising, we would like to contact participants four months after the final data-collection visit to collect well-being questionnaire data, behavioral/attention task, cortisol, and a blood sample. If a four-month follow-up is not conducted, your bonus will be contingent only on completing the first six visits and your class. If you are interested in providing information that would allow us to contact you in the future, please complete the sheet entitled “Contact Information for Long-Term Follow-up” attached to this consent form. If at any time you decide that you prefer not to be contacted in the future, please inform Dr. Antoine Lutz at the Department of Psychology, Waisman Center, Room T231, (608) 262-8705, alutz@wisc.edu, or Dr. Donal MacCoon, Room A132, (608) 263-1968, dgmaccoon@wisc.edu, and they will gladly take your name off our contact list.

## What are the costs to me for my participation? Will I be paid for participating?

Your participation includes completing 10 clinic visits and completing one of the two 8-week classes that we are researching. We will decide, based on chance (i.e., the flip of a coin) which class you will participate in. After the research study is completed, you are free to take the class that you did not complete during the research study, at your own expense. In this research study, you will be monetarily reimbursed for your participation after completing each phase of measurements and will receive a bonus for completing the final round of measures. Specifically, you will be paid at the rate of $25 per clinic visit, or $250 for ten visits total. This is approximately $14 per research hour. You will not be paid for your time attending the intervention program. You can withdraw from the research study at any time without penalty. In addition to the $250 for the 10 clinical visits, you will be paid a bonus of $225 for completing the entire program (attending all 10 clinic visits and all classes), a total of $475. After the study, you will be given information about the class you did not attend. The second class is optional; if you wish to join the second class, you must pay for it ($475) and you will not be paid for your time in the class since it is not part of the research study. The bonus is not contingent upon your participation in the second 8-week class.

## What are the potential benefits to me for my participation?

The only direct benefits to you are any that result from your involvement with the two programs designed to increase health and well-being. The information gained from this study may contribute to scientific knowledge about how such programs impact pain regulation, negative affect, attention, and social stress.

.

## What are the potential risks, discomforts, and inconveniences to me?

- Painful stimulation: The painful stimulation, such as the feeling of moderate heat on the palm of your hand, will cause you discomfort but should not injure you or cause any long-term damage. The stimulus will not exceed 49°C (120°F.), which is considered a safe maximum temperature for hot tap water. Although temperatures in this range have been used safely in other research, some minor irritation is possible. One hand will receive stimulation during practice and the opposite hand will be stimulated during testing. You will be given an emergency button in the hand that is not being stimulated which will allow you to stop the study. It may take longer for you to push the emergency button when it is not in your dominant hand (i.e. your writing hand), increasing the risk of any irritation occurring on your dominant hand.
- Blood sample collection: There may be some discomfort, minor bruising, or fainting when taking a blood sample. There is also a very small chance (less than 1%) of infection at the needle puncture site.
- Saliva (spit) collection: Some participants may experience slight discomfort related to chewing a cotton swab. Because you will put these swabs in your mouth, this discomfort is likely to be minimized.
- MRI brain scanning: During the MRI portion of the study, some individuals may feel uncomfortable being in an enclosed space, or may be tired and/or may experience physical discomfort from lying still on their back during the scanning session. Some people have also reported tingling or tapping sensations, or muscle twitches in different parts of their body during the imaging procedure. These sensations are not hazardous and should not cause you any discomfort. Occasionally, people who have clasped their hands tightly together during the study have reported a feeling of electrical shock in their hands and arms. This is also not hazardous; however, to avoid any possible discomfort, you should not clasp your hands together during the study. You are free to stop your participation in the MRI at any time if you feel claustrophobic or uncomfortable, or for any other reason. You will hear loud tapping and pinging noises as the MRI machine images your head. Earplugs or headphones will be provided to make this noise less unpleasant, but these earplugs do not completely block the sounds, so that we can still talk to you.
- Effects of images: Some of the images you will see you might find unpleasant. If you are concerned about the images, you are free to refuse to participate in any portion of the study.
- Social stress test: Participation in the stress test will be stressful. Prior to investigation all participants will be informed that they will be asked to perform a 15-minute mental stress test. If anxiety or distress becomes too uncomfortable, participants are free to terminate the stress test before the 15-minute interview is completed. To the best of our knowledge no negative long-term consequences have been reported since the implementation of this stress test in 1993.
- Capsaicin cream: Capsaicin cream (.1%) is available over-the-counter at most local pharmacies. It is associated with a mild to moderate burning or itching sensation that lasts approximately 30-60 min., but in rare cases can persist for up to 48h. Severe discomfort has been reported in a small number of individuals. If severe discomfort occurs, the cream can be thoroughly removed with soap and cold water and the discomfort is expected to disappear completely. In addition, increased sensitivity to heat and touch may occur and last for several hours after removal of capsaicin. For this reason, you should avoid exposing the area of your forearm where the cream was applied to the sun or hot water for the rest of the day.
- Suction blisters: There is a slight risk of infection due to the hole in the blister roof where the fluid is removed. However, no incidences of infection have been reported with this procedure, even in individuals with impaired immune systems. The most commonly observed negative effect is a pink or white spot at the site of the blister that will disappear after some time. The study physician, Dr. James Davis, will be available by cell phone to speak to you at any time for questions regarding concerns about this procedure. He can be reached at 608-217-9405.
- Breach of confidentiality: When providing information for study purposes, there is the risk that this information will not remain confidential. The investigators take this issue very seriously and every effort will be made to keep your information in a locked, secure environment. Additionally, your name will be removed from all documentation and you will be identified by number only.

# **Possible discovery of findings related to medical imaging: There is a remote chance that imaging may reveal unsuspected findings that are of potential medical importance. Therefore, the images obtained during your session will be reviewed by a radiologist (a physician trained in the interpretation of medical images) in order to screen for abnormalities. Such findings can potentially be serious, but this is rare; most often they have little or no medical importance. Occasionally the significance of a finding cannot reliably be determined from the images obtained. Knowing about unsuspected abnormalities on your images may be of some benefit to you, particularly if they are medically important. On the other hand, if the significance of a finding is low or uncertain, there may be little benefit in knowing about it and there may be some risks; for example, it could affect your insurability or employability, or just make you worried**

# **Whether or not you want to be informed of findings of potential medical significance is up to you. Please indicate your preference by signing your initials on the appropriate blank:**

______ Do not inform me of any abnormal findings.

______ If abnormal findings appear to pose a significant risk to my health, please inform me of them.

______ Even if the significance of abnormal findings is uncertain, please inform me of them.

## Name of physician to contact

If you do wish us to report any findings to your physician, you must provide us with the name and location of your primary physician, prior to your scan.

Name of primary physician
City or clinic
Health Care Provider

Whether or not you want your physician to be informed of findings of potential medical significance is up to you. Please indicate your preference by signing your initials on the appropriate blank:

______ Do not inform my physician of any abnormal findings.

______ If abnormal findings appear to pose a significant risk to my health, please inform my physician of them.

______ Even if the significance of abnormal findings is uncertain, please inform my physician of them.

## Disclaimer for normal volunteers

If you volunteer for this research study, the MRI scan that we will perform is NOT necessarily equivalent to a MRI scan used to diagnose medical problems. Many potentially serious problems may be undetectable on these scans. A negative MRI should not be used to avoid a visit to your primary physician. Should you be having physical symptoms that you are concerned about, you should see your primary physician, who will determine the studies required to arrive at a diagnosis.

**Who should not participate in this study?**

Some subjects should not participate in MR studies. These include persons with metallic implants, such as prostheses or aneurysm clips, or persons with electronic implants, such as cardiac pacemakers. The magnetic field generated by the MR machine can cause a displacement or malfunctioning of these devices. We know of no risks or adverse effects from the radio signals used in this study. Some subjects report some anxiety or claustrophobia in the MR scanner since the head must be placed fully inside the scanner tube. In addition, fatigue and physical discomfort due to the length of the MRI session are possible.

Women who are pregnant must not participate in this study. The potential risks to a fetus from the MRI scan are not known. If you have any reason to suspect that you might be pregnant or become pregnant during the course of this study, you must not participate. You must be right-handed and be free of neurological problems to participate in this study.

Individuals who have diabetes mellitus or peripheral vascular disease, or those who are currently taking steroidal anti-inflammatory medication should not participate in the capsaicin/suction blister part of the study, but may participate in the rest of the study procedures.

##### **How will I be compensated in the event of injury?**

Your participation is not expected to result in any injury. In the event that you are physically injured as a result of participating in this research, emergency care will be available. You will, however, be responsible for the charges for the emergency care. There is no commitment to provide any compensation for research-related injury. You should realize, however, that you have not released this institution from liability for negligence. Please contact the investigator, Dr. Antoine Lutz, at (608) 262-8705, [alutz@wisc.edu](mailto:alutz@wisc.edu), or Dr. Donal MacCoon, Room A132, (608) 263-1968, [dgmaccoon@wisc.edu](mailto:dgmaccoon@wisc.edu).

## Who will see the results of this research?

Your participation in this study is confidential, which means that nobody outside of authorized study personnel will have access to the information you give us. All data will be stripped of personal information and identified by an identification number only. The results of this study may be published or presented at professional conferences, but you will not be identified by name. In the unlikely event that we would receive a court order to release your study records to authorities, we would be obligated to do so.

## Is my participation voluntary?

Yes, your participation in this study is entirely voluntary. You may withdraw from this study at any time. Your decision to withdraw will not affect any benefits to which you are entitled.

Before you sign this form, please ask questions on any aspects of this study that are unclear to you. You may take as much time as necessary to think this over. You are free to withdraw from this study at any time, even after the scanning has begun.

**Contact Information**

Please feel free to ask questions before agreeing to participate. Should you choose to participate, you are encouraged to ask questions throughout the study. If you want more information on the rights of research subjects, you may contact the hospital Patient Relations Representative at (608) 263-8009. If you have any questions after participating in the study, please contact the investigator, Dr. Antoine Lutz, at (608) 262-8705, or Dr. Donal MacCoon, Room A132, (608) 263-1968.

Authorization: I, ___________________________________, have read the above and decided to participate in the

(Printed name of subject)

research project described there.

___________________________________ ­ _______________

Signature of Subject Date

____________________________________ ­ _______________

Signature of Person Obtaining Consent Telephone

## Pregnancy Status – Female Participants Only

The regulations for this type of research do not allow the participation of female subjects who are pregnant. Please sign this statement only if you are certain you are not pregnant. If you are not certain, please do not sign this statement.

I confirm that I am not pregnant.

______________________________________________________

Signature of Subject Date

**Contact Information for Long-Term Follow-up**

**Please provide whatever contact information you feel comfortable releasing to our laboratory.**

**All information will be kept confidential.**

The information you provide will be used if we have difficulty contacting you for the 4-month follow-up.

It is often valuable for research to measure longer-term outcomes. To cover this possibility,

please check **one** of the following:

- - - - Don’t contact me for anything beyond the 4-month follow-up for the current study.
      - You may contact me for the current study beyond the 4-month follow-up.
      - You may contact me for any follow-up for the current study AND for asking for my participation in future studies.

Your Name: _____________________________________________________

Your current contact information:

Street address: ______________________________________________ City, State, Zip: ____________________________________

Phone (home) ____________________________(work)______________________________(cell) ____________________________

Email: ___________________________________________ Social Security Number: _____________________________________

Drivers License: State ________ Number ________________________

**Please list contact information for a parent or other relative who will always know how to contact you:**

Name: _____________________________________________________

Relation to you: _____________________________________________

Street address: ______________________________________________ City, State, Zip: ____________________________________

Phone: ____________________________________________________ Email: ___________________________________________

**Please list contact information for a friend or an additional relative (at a different address than the relative listed above) who will always know how to contact you:**

Name: _____________________________________________________

Relation to you: _____________________________________________

Street address: ______________________________________________ City, State, Zip: ____________________________________

Phone: ____________________________________________________ Email: ___________________________________________

| **Name:** |  | **Phone #:** |  | **Date Of Birth:** |  |
| --- | --- | --- | --- | --- | --- |
|  |  |  |  |  |  |

| *U W H E A L T H S P O R T S M E D I C I N E F I T N E S S C E N T E R* | *( 6 0 8 ) 2 6 3 – 7 9 3 6* |
| --- | --- |

#### *`*

| 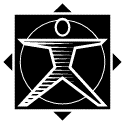 | *Physician’s Authorization Form* The above named patient is interested in participating in an exercise program at the UW Health Sports Medicine Fitness Center. Prior to participating new members must acquire authorization from their physician. Please complete this form as thoroughly as possible for this patient. **Forms missing information (incomplete sections) will be returned for completion prior to participation.** |
| --- | --- |
|  |  |

**I. Medical History** (check and specify all applicable)

|  |  | Heart Disease (MI, PTCA, CABG, etc.) |  |
| --- | --- | --- | --- |
|  |  |  |  |
|  |  | Diabetes Mellitus |  |
|  |  |  |  |
|  |  | Neurological Disorder |  |
|  |  |  |  |
|  |  | Stroke |  |
|  |  |  |  |
|  |  | Peripheral Arterial Disease |  |

**II. Risk Factors** (check and specify all applicable)

v

*It is our policy to recommend a graded exercise tolerance test (ETT) for men over the age of 40 and women over 50 who have 2 or more of the following risk factors who are BEGINNING a VIGOROUS exercise program:*

|  |  |  | | | | | |  | | |
| --- | --- | --- | --- | --- | --- | --- | --- | --- | --- | --- |
|  |  | Diagnosed Hypertension | | | |  | | | | |
|  |  | Resting SBP > 160 OR Resting DBP > 90 OR taking any antihypertensive medication | | | | | | | | |
|  |  |  | | | | | |  | | |
|  |  | Hyperlipidemia | | | |  | | | | |
|  |  | Total Cholesterol = |  | HDL = |  | | TChol/HDL Ratio = | |  |  |
|  |  |  | | | | | |  | | |
|  |  | Diabetes *(Type I or II)* | | | |  | | | | |
|  |  |  | | | | | |  | | |
|  |  | Obesity | | | |  | | | | |
|  |  |  | | | | | |  | | |
|  |  | Cigarette Smoking | | | |  | | | | |
|  |  |  | | | | | |  | | |
|  |  | Family history of coronary or atherosclerotic disease (parents/siblings prior to age 55) | | | | | | | | |
|  |  |  | | | |  | | | | |
|  |  | Inactivity or Sedentary Lifestyle | | | |  | | | | |

**III. Screening Choices** (select one)

|  |  | A graded exercise tolerance test is not indicated for this patient. | | |
| --- | --- | --- | --- | --- |
|  |  |  |  |  |
|  |  | I have performed a graded exercise tolerance test and have enclosed a copy of the test results. | | |
|  |  |  |  |  |
|  |  | I would like my patient to receive a graded exercise tolerance test through the UW Preventive Cardiology Program before participating in an exercise program. (This test is not included in the program fee). | | |

**IV. Patient Limitations/Restrictions**

|  | *Specify:* |
| --- | --- |
|  |  |

|  |  | No restrictions or limitations for this patient |
| --- | --- | --- |

|  |  | I would like to speak with the exercise coordinator prior to my patient participating in any exercise program. Please call me at the below number. |
| --- | --- | --- |

### V. Physician’s Authorization

|  | Physician’s Signature: |  | Date: |  |
| --- | --- | --- | --- | --- |
|  | PRINT Physician Name: |  |  |  |
|  | Clinic Name & Address: |  | Phone: |  |

Mail, FAX (608-263-2215) Attn: **Fitness Center-HEP/MBSR,** or deliver completed form to UW Health Sports Medicine Center, 621 Science Drive, Madison, WI 53711_

Medical & Physical Activity History Form
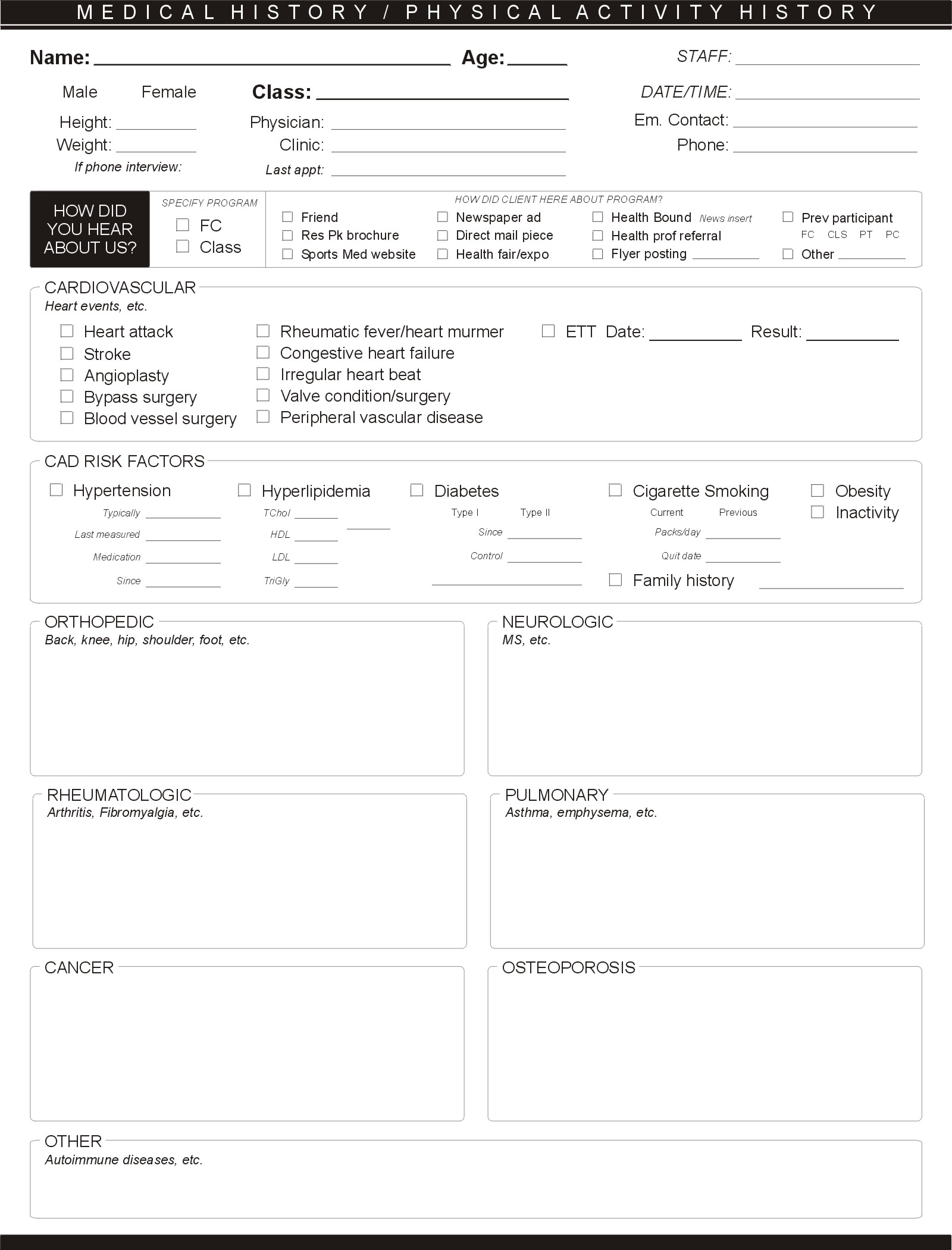


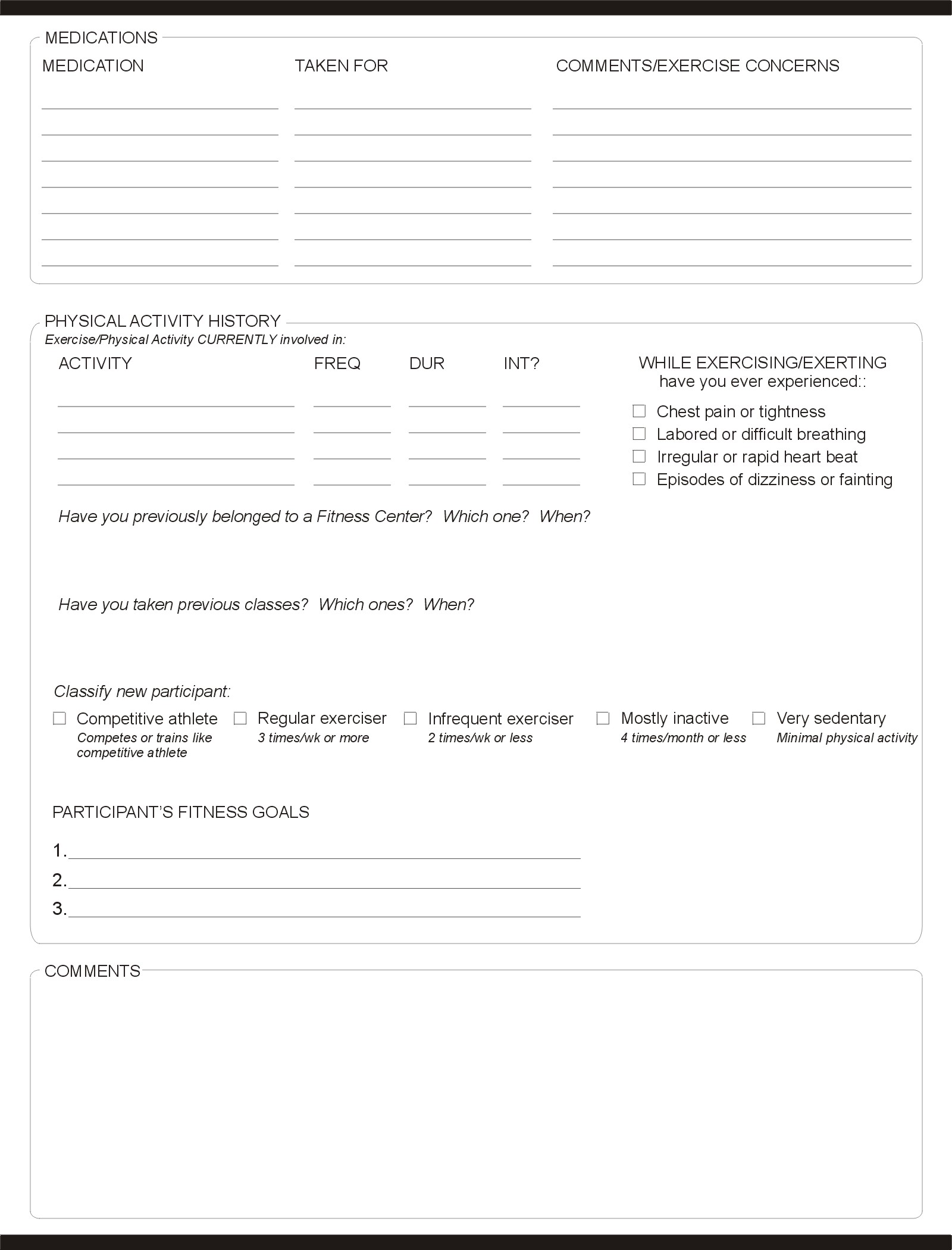


Registration Form

HEALTH ENHANCEMENT PROGRAM &

MINDFULNESS BASED STRESS REDUCTION PROGRAM

# UW Health

# Name: ___________________________________ Date: ______________________

**General Information:**

1. Please describe what you consider to be stressful in your life, i.e. job, relationships, a chronic or life threatening illness, etc.

**2.** What are your greatest worries and stresses?

1. What are the current ways you use to try to manage stress?

Helpful: 1)

2)

3)

Not helpful: 1)

2)

3)

1. Please rate your overall stress level at this point in your life using a 1 – 10 point scale.

“1” = stress free, and “10” = stressed to the max. Mark an “X” at the appropriate area on the line below.

“1” _________________________________________________________ “10”

**-1-**

**(over)**

1. Please describe any previous experience you have had with physical activity, stress reduction, nutrition, meditation, acupressure, relaxation, reflexology, mindfulness, healing touch, imagery, and other mind-body approaches to healing and health. If you have not had any prior experience, please write “no experience”.
2. Have you ever followed any kind of special diet?

Please Describe.

How long?

1. Do you currently follow any kind of special diet?

Please Describe.

How long?

1. What types of music do you listen to on a consistent basis?
2. Do you find music nurturing?
3. Do you listen to music when you feel stressed?

- 2 -

## Name _______________________________________

1. What target goals would you like to set for yourself in taking this program?
2. What do you care about most in your life?

1. What gives you pleasure? What do you enjoy?

**14.** What are your greatest hopes?

**15.** Are you currently involved in a specific medical treatment or psychological counseling program? Please list any current medications.

**16.** Please list any previous hospitalizations with dates.

-3-

(over)

**17.** Please describe any complementary or alternative treatments you have received or are receiving.

**18.** Please describe your physical health right now.

**19.** Describe your sleep quality.

**20.** Please describe your emotional health. What are your biggest emotional challenges?

**21.** Please describe your spiritual health.

What is most challenging spiritually?

**-4-**

###### Name _______________________________________

**22.** How do you feel about the future?

**23.** Please describe your support system?

**24.** At the completion of this class, imagining that you do learn everything that you want to learn, how would you like to experience yourself? For example, how do you want to be feeling in your body? How do you want to be experiencing your mind, your emotions, your connections with other people, etc?

**25.** Please add anything else that’s important for the instructor to know about you and your situation.

**Thank you very much for completing these questions.**

**- 5 -**

MRI Screening Form

# MRI Screening FormMRI Form

Date: _____/_____/_____ By: ___________________________________

Subject: ______________________________________________________________________

Study: _________________________ PI: ___________________________________

ID#: ___________________________ SS#: _____-_____-_____

Sex: Female Male Age: ______ DOB: _____/_____/_____

Weight: _______ Home Phone: _____-_____-_____

Height: ____’____” Work Phone: _____-_____-_____

HatlSize:______ Recruitment:____________________________ _____________________________________________________________________________________________

# ***YES NO***

*___ _*__ **Do you have corrected vision?**

___ ___ **Is your vision sufficient to see without glasses, as if you were looking through binoculors?**

___ ___ **Do you wear contact lens?**

___ ___ **Do you have TMJ (Temporal Mandibular Joint) disorder or other problems with biting/chewing?**

Type? _________________________________________________________________________________

*___ ___* **Have you ever had an MRI scan?**

When? ________________________________________________________________________________

Why? _________________________________________________________________________________

*___ ___* **Have you ever had surgery or a similar invasive procedure?**

When? ________________________________________________________________________________

Type? _________________________________________________________________________________

___ ___ **Have you ever had heart surgery?**

When? ________________________________________________________________________________

Type? _________________________________________________________________________________

___ ___ Do you have a Pacemaker?

___ ___ Do you have an implanted cardiac defibrillator?

___ ___ Do you have an artificial heart valve?

___ ___ Do you have cardiac pace wires?

___ ___ **Have you ever had head or brain surgery?**

When? ________________________________________________________________________________

Type? _________________________________________________________________________________

___ ___ **Have you ever had eye surgery?**

___ ___ Do you have lens implants?

___ ___ **Do you wear dentures?**

___ ___ **Have you ever had ear surgery?**

___ ___ **Do you have a cochlear implant?**

___ ___ **Do you wear a hearing aid?**

****CONTINUED ON REVERSE SIDE****

MR Screening, Rev 06/20/04, WI, Page 1 of 2

***YES NO***

___ ___ **Have you ever had back surgery?**

When? _______________________________________________________________________

Type? ________________________________________________________________________

___ ___ **Do you have any implanted devices of any type?**

___ ___ Breast/Penile?

___ ___ Electrodes?

___ ___ Pumps (e.g., drug infusion device)?

___ ___ Bone or socket?

___ ___ Neurostimulators/Biostimulator?

___ ___ **Do you have any dental or orthodontic implants (fillings are O.K.)?**

Type? _______________________________________________________________________

___ ___ **Do you have any type of prosthesis?**

Type? ________________________________________________________________________

___ ___ **Do you have any type of orthopedic implant (e.g., pins, rods, screws, nails)?**

Type? _________________________________________________________________________________

___ ___ **Do you have any permanent cosmetics (e.g., eyeliner)?**

___ ___ **Do you have any tattoos on your upper body?**

Where/Extent? _________________________________________________________________

___ ___ **Do you have any body piercing(s)?**

Where? _______________________________________________________________________

[If “YES”, inform the subject they will need to be removed prior to entering the scanning room.]

___ ___ **Do you have a history of any metal in your body?**

___ ___ **Have you every worked as an occupational metal grinder?**

Description: ___________________________________________________________________

___ ___ **Have you ever worked with metal as a hobby?**

Description: ___________________________________________________________________

___ ___ **Did you routinely wear safety glasses?**

___ ___ **Have you ever sought medical attention for metal in your eyes?**

___ ___ **Have you ever had metal fragments removed from your eyes?**

___ ___ **Have you ever been struck by a gun shot, B.B. or shrapnel?**

___ ___ **Do you have any physical disabilities?**

Type? ________________________________________________________________________

___ ___ **Do you have any involuntary motor disorders?**

Type? ________________________________________________________________________

___ ___ **Have you ever experienced claustrophobia?**

When? ______________________________________________________________________

___ ___ **Do you have any back problems that would prevent you from lying still for up to 2 hours?**

**Female Subjects**

___ ___ **Are you or is there a chance you are pregnant?**

___ ___ **Do you have an intrauterine device (IUD)?**

****ANY QUESTIONABLE CONDITIONS MUST BE APPROVED BY THE MRI TECHNICIAN****

MRI Screening Form Key

**MRI Screening Form Key**

_______________________________________________________________________________________________

Date: _____/_____/_____ By: ___________________________________

Subject: ____________________________________________________________________________

Study: _________________________ PI: ______________________________

ID#: ___________________________ SS#: _____-_____-_____

Sex: Female Male Age: ______ DOB: _____/_____/_____

Weight: _______ Home Phone: _____-_____-_____

Height: ____’____” Work Phone: _____-_____-_____

Hat Size: ______ Recruitment: ____________________________

This is a tricky issue, and persons having heads too large to fit into the head coil (with goggles and bite bar) are actually rather common. The critical dimension is not really hat size, per se, but rather the dimension from the occiput to the front of the mouth. The reason for asking about hat size is really rule-of-thumb. Persons reporting hat sizes greater than 7 ¾ will probably not fit in the head coil. This issue is further complicated by the fact that very few people know their hat size.

_______________________________________________________________________________________________

**General rule: We are doing research, not medical diagnosis. Our subjects don’t need the MRI for diagnosis of a medical problem. Because of that, we should be very conservative in our acceptance of subjects. If an item come up that leads you to reject a subject, explain the importance of these screening procedures and the fact that we are being particularly strict because this is controlled research and not necessary diagnosis. With a patient population, this will require delicate wording.**

*Yes No*

*___ _*__ **Do you have corrected vision?**

Do you know your vision rating or prescription?_______________________________________________

___ ___ **Do you wear contact lens?**

The reason for asking these questions relates to the goggles that will be used to present visual stimuli. We don’t really know the range of vision that we can accommodate with the focus on the device. If subjects can wear contacts, they should. If someone reported vision of something extreme, say 2000/2000, I would question their compatibility. This is really a rule-of-thumb issue.

___ ___ **Do you have TMJ (Temporal Mandibular Joint) disorder or other problems with biting/chewing?**

Type? _________________________________________________________________________________

This has to do with the Bite Bar. We don’t know what problems might occur with this, but because they will have the device in their mouth in a stationary position for over one hour, we should be sensitive to potential problems. I don’t know how often this issues will come up, but I would describe the Bite Bar and see what the subject’s reaction is. If some one is positive for TMJ, I would not accept them. There have been complaints of pain from subjects who had TMJ and we didn’t know it.

*___ ___* **Have you ever had an MRI scan?**

When? ________________________________________________________________________________

Why? _________________________________________________________________________________

This is background. If they have had an MRI, they were once *safe*, but that does not mean that are still *safe* If they had an MRI for some type of head injury, for example a car accident, that may alert to potential problems that the questions below will not specifically address.

*___ ___* **Have you ever had surgery?**

When? ________________________________________________________________________________

Type? _________________________________________________________________________________

Surgery involves metal parts entering the body. Often times this results in intentional placement of metal in the body (i.e., clips or screws), and, in rare cases, metal can be left in the body accidentally. If there has been a surgery, inquire about the type, are stitches still present, were staples used to close any openings, etc. The questions will be motivated by the type of surgery. Recent surgery would not exclude a subject, but we want to know if there are potential problems.

___ ___ **Have you ever had heart surgery?**

When? ________________________________________________________________________________

Type? _________________________________________________________________________________

If someone has had heart surgery, there is the chance that something was implanted. Cardiac pace wires (of which the subject may not even be aware) can remain. At this point, I would not accept anyone who has had heart surgery.

___ ___ Do you have a Pacemaker?

___ ___ Do you have an implanted cardiac defibrillator?

___ ___ Do you have an artificial heart valve?

___ ___ Do you have cardiac pace wires?

___ ___ **Have you ever had head or brain surgery?**

Unless this was the focus of the study, for both scientific and saftey reasons, I would not accept anyone who has had brain surgery. They are, by definition, not of the same category as non-brain surgery subjects.

When? ________________________________________________________________________________

Type? _________________________________________________________________________________

___ ___ **Have you ever had eye surgery?**

___ ___ Do you have lens implants?

Lens implants should be a flag. While not metal, they could affect the *tissue heating* that is a result of the physics of MRI. I would reject subjects with lens implants. Cataract surgery done before 1986 (or thereabouts) needs to be followed up.

___ ___ **Do you wear dentures?**

Many dentrues have metal in their design. The subject might be required to remove them for the scan. Some type of dental implants are actually held in place with magnets.

___ ___ **Have you ever had ear surgery?**

___ ___ **Do you have a cochlear implant?**

___ ___ **Do you wear a hearing aid?**

Reject subjects with ear implants. If the subject wears a hearing aid, highlight this item so that at the time of scan it is removed.

___ ___ **Have you ever had back surgery?**

When? ________________________________________________________________________________

Type? _________________________________________________________________________________

This is a warning indicator. Persons with back trouble (past or current) may not be able to be supine for the duration of the scan.

___ ___ **Do you have any implanted devices of any type?**

___ ___ Breast/Penile?

___ ___ Electrodes?

___ ___ Pumps (e.g., drug infusion device)?

___ ___ Bone or socket?

___ ___ Neurostimulators/Biostimulator?

Reject subjects with these types of implants. Some bone & socket replacements may be acceptable.

___ ___ **Do you have any dental or orthodontic implants (fillings are O.K.)?**

Type? _________________________________________________________________________________

Braces are not acceptable. Most types of *permanent* retainers are acceptable (i.e., those that stabilize the front teeth).

___ ___ **Do you have any type of prosthesis?**

Type? _________________________________________________________________________________

Mechanical prostheses would need to be removed.

___ ___ **Do you have any type of orthopedic implant (e.g., pins, rods, screws, nails)?**

Type? _________________________________________________________________________________

This is a warning indicator. Depending on location and material, they may be acceptable.

___ ___ **Do you have any permanent cosmetics (e.g., eyeliner)?**

___ ___ **Do you have any tattoos on your upper body?**

Where/Extent? _________________________________________________________________________

The ink used in tattoos and permanent cosmetics can have a metallic base and can cause “tissue heating” in some cases. This is not necessarily a rejection factor, but we should know about it.

___ ___ **Do you have any body piercing(s)?**

Where? ________________________________________________________________________________

Any earrings on the head must be removed. Piercings on other parts of the body may have to be removed.

___ ___ **Do you have a history of any metal in your body?**

Obviously we need to know about this. Rejection depends on the what, where & how.

___ ___ **Have you every worked as an occupational metal grinder?**

Description: __________________________________________________________________________

Working with metal often leads to accidents in which metal gets into and remains in the body.

___ ___ **Have you ever worked with metal as a hobby?**

Description: __________________________________________________________________________

___ ___ Did you routinely wear safety glasses?

Many people answer “yes” to the hobby question. For example, “sharpening ice skates.” It is very important that you pursue this to inquire exactly what they did and for how long. It is *essential* that they tell you they wore safety glasses.

___ ___ **Have you ever sought medical attention for metal in your eyes?**

___ ___ **Have you ever had metal fragments removed from your eyes?**

___ ___ **Have you ever been struck by a gun shot, B.B. or shrapnel?**

All of the above should be obvious. If they answer “yes” to any of these, it needs to be pursued thoroughly. Any potential subject that reports *even the possibility* of having metal in their eyes needs to be checked with an eye orbit x-ray. Despite common misconception, there are many people who have bits of metal in their eyes and have do idea about it.

___ ___ **Do you have any physical disabilities?**

Type? ________________________________________________________________________________

We would need to know this in case they needed help getting to/from on/off the scanner.

___ ___ **Do you have any involuntary motor disorders?**

Type? ________________________________________________________________________________

If this would lead to movement during the scan, they should be rejected.

___ ___ **Have you ever experienced claustrophobia?**

When? _______________________________________________________________________________

This should be self-explanatory.

___ ___ **Do you have any back problems that would prevent you from lying still for up to 2 hours?**

This should be self-explanatory.

**Female Subjects**

___ ___ **Are you or is there a chance you are pregnant?**

Pregnant women should be rejected. It may be appropriate in certain protocols to include a formal pregnancy test.

___ ___ **Do you have an intrauterine device (IUD)?**

This needs to be followed up. We would need to confirm with their doctor that the device contained no metal (usually copper).

Finally, there could be problems with severely obese persons fitting into the scanner. We should pay attention to the weight-height ratio. There are actually safety parameters build into the scanner which prevent its operation if the weight-height ratio is out of limit.

MRI Doctors Note Packet (v6/12/06)

Waisman Center

1500 Highland Ave.

Madison, Wisconsin 53705

Dear _________________________,

We understand that you are interested in participating in a research study here at the University of Wisconsin-Madison, a study that will involve an MRI of your brain. As part of our screening procedures, you informed us that you had the following procedure performed: _____________________________________________________________________________in _________________________ (Month/Day/Year).

As we discussed on the phone, in order to ensure that it is safe for you to undergo MRI scanning, we will need for you to contact your physician to ask him/her some specific questions about this procedure. Please call your physician to ask him/her the questions you’ll find on the next page. Once you’ve done so, please return the sheet to us either in person, by fax (608-262-9440, attn: Jenna Sheftel), or by mail (please use the pre-labeled envelope that has been included if you return form by mail).

In addition, we would like to receive copies from your physician of the relevant paperwork from your medical chart that documents information about the hardware that was implanted. Your physician will likely ask you to provide written authorization for release of this information to our office. We are therefore enclosing a “Medical Release of Information” form that you can send to your physician’s office.

Many thanks for your assistance and continued interest in this research study. Whether or not you’re able to undergo the MRI portion of the study, we look forward to working with you. Should you have any questions, please feel free to call Jenna Sheftel, the study coordinator, at (608) 262-9888 (Madison local number).

Sincerely,

The Well-Being Study Team

**Questions to ask your physician**

I am interested in participating in a research study with a brain MRI component. Because I’ve had

_______________________________________________________, I need to collect some information

(your implant/condition)

from my doctor to help the researchers decide whether or not I can safely do the MRI portion of the

study (using a 3 Tesla MRI scanner).

1. Who is the manufacturer of the hardware that was implanted?
2. What is the model number of the hardware that was implanted?
3. To your knowledge, is this hardware safe for scanning in a 3 Tesla MRI scanner?

Yes No

(circle one)

1. To your knowledge, do I have any other conditions or implanted equipment that would make it unsafe for me to have an MRI of my head in a 3 Tesla scanner?

Yes No If yes, describe: _____________________________________

(circle one)

1. Would an x-ray be in order to decide if a head MRI is safe for me? (The researchers can provide this free of charge).

Yes No

(circle one)

1. Can you please send copies of the portions of my medical record that contain information about the hardware that was implanted to the study researchers? I will be sending to you a release of information for this purpose that has the contact information.

Yes No If no, reason: _______________________________________

(circle one)

Waisman Center

1500 Highland Ave.

Madison, Wisconsin 53705

tEL: 608-265-8107

faX: 608-262-9440

####

##### MEDICAL RELEASE OF INFORMATION

Physician’s Name and Address:

­­­­­­­­­­­­­­­­­­­­­­­­­­­­­______________________________________________

______________________________________________

______________________________________________

**I, ____________________________________, give my permission for the release of information from my**

(Please print your name)

**medical record regarding _______________________________________________________________ for the**

(Please briefly describe the implant/metal in question)

**purpose of determining whether it is safe for me to undergo an MRI of my brain for a research study at the**

**WM Keck Laboratory for Functional Brain Imaging & Behavior at the University of Wisconsin – Madison.**

I wish for this information to be released to:

WM Keck Laboratory for Functional Brain Imaging & Behavior

University of Wisconsin – Madison

1500 Highland Ave. #T117

Madison, WI 53705

Phone: (608) 265-8107

Fax: (608) 262-9440

ATTN: ____________________

Authorization: I am the individual to whom the information/record applies. This release shall be valid for a period of 90 days from the date indicated below.

________________________________________________________ _________________________________

Signature Date

_________________________________________________

Date of Birth

___________________________________________________

Social Security Number

Saliva Sampling Instructions

Saliva sampling Instructions

**The saliva sampling kit:**

The kit consists of the following parts:

- **Three containers** labeled **“Day 1”, “Day 2”** or “**Day3**”.
- Each container contains **a set of 5 plastic tubes**. On each plastic tube there is a label stating the Day (1, 2 or 3) as well as a time point (for example “Day 1 - Awakening”).
- Additionally we will supply you with a **timetable and questionnaire sheet**, where all the requested time points for saliva collection are listed. We will ask you to record the time you took your saliva samples there, as well as rate your stress level when you have taken the sample. The scale is from 1 to 7, with 1 having no stress and 7 having extreme stress. We will also ask you to describe your activities during the day.

### How do I collect saliva samples?

**Please open the container only to take out a single swab at the corresponding time point!**

As soon as you wake up in the morning, open the container with the label “Day 1”. Take out one cotton swab and close the container immediately. Chew the swab for about a minute. Then take the corresponding tube with the label “Day 1 – Awakening” and place the swab into the tube. Close the tube with the stopper and place the tube in the refrigerator. Finally, note the time when you collected your first sample on the time schedule.

After 30 minutes, repeat the whole procedure: Open the container, take out one swab, close the container, chew the swab for about one minute, take the corresponding tube (“Day 1 – 30 min after awakening”), place the swab in the tube, close the tube, put it into the refrigerator and finally note the time point when you have chewed the swab.

Repeat this procedure for every saliva sample for Day 1, Day 2 and Day 3.

**Please be also aware of the following instructions:**

Blood in saliva and high or low pH can lead to false elevated results. Therefore please note:

- **Do not clean your teeth after awakening until you have taken the two morning saliva samples.**
- **Do not eat prior to saliva collection.**
- **Do not drink coffee, milk or any fruit juice 10 minutes prior to saliva collection.**
- **The night before you collect the first saliva samples you should not drink alcohol or stay up too late.**

**Saliva collection schedule**

**Time table**

Subject ID __________________ Session ____________ Study __________ Date ____/____/____

|  | **Scheduled time point** | **Time when you collected the sample** | **Stress Level (1-7)  (Report after  taking sample)** |
| --- | --- | --- | --- |
| **Day 1** | **Awakening** |  |  |
|  | 30 minutes after awakening |  |  |
|  | Before lunch |  |  |
|  | 3 pm |  |  |
|  | bedtime |  |  |

| **Day 2** | Awakening |  |  |
| --- | --- | --- | --- |
|  | 30 minutes after awakening |  |  |
|  | Before lunch |  |  |
|  | 3pm |  |  |
|  | bedtime |  |  |

|  | **Scheduled time point** | **Time when you collected the sample** | **Stress Level (1-7)  (Report after  taking sample)** |
| --- | --- | --- | --- |
| **Day 3** | Awakening |  |  |
|  | 30 minutes after awakening |  |  |
|  | Before lunch |  |  |
|  | 3pm |  |  |
|  | Bedtime |  |  |

**Stress Level Instructions:** Please report the current level of stress you are feeling once you have completed the saliva sample. The scale is from 1 to 7, with 1 having no stress and 7 having extreme stress.

**Saliva Collection Schedule**

**Questionnaire**

Subject ID __________________ Session ____________ Study __________ Date ____/____/____

|  | **Description of the  activity during the day** | **General comments** |
| --- | --- | --- |
| **Day 1** |  |  |
| **Day 2** |  |  |
| **Day 3** |  |  |

**Others questions:**

Did you take a nap today?

How many hours did you sleep?

Did you feel healthy today?

Were you engaged in any stressful events during the day (in terms of social interactions, work, class, etc)?

# Cortisol Background Information (sheet 1)

Subject ID __________________ Session ____________ Study __________ Date ____/____/____

1). What time did you wake up today? _______

Is this a typical wake up time for you? **YES** or **NO**  (circle one)

If no, what time is typical?_______

2). How many hours of sleep did you get last night? _______

Is this a typical amount for you? **YES** or  **NO**

If no, how many hours are typical?______

3). Did you take a nap today? **YES** or **NO**

If so, when did you start to nap and how long was it? _________________

4). Do you:

1. Feel like you have a cold, cold symptoms, the flu, stomach virus, or sinus infection today? **YES** or **NO**
2. Have seasonal allergies? **YES** or **NO**
   - 1. If yes, are they acting up today? **YES** or **NO**
3. Feel like you might be coming down with something like a cold or infection today? **YES** or **NO**

5). Have you had an allergic reaction within the past week? **YES**  or **NO**

If yes, to what did you react (food, bee sting, medication, etc.)? ________

6). Are you on any antibiotics? **YES**  or **NO**

If yes, please list the name(s). ___________________

7). Did you take any over the counter medication today? **YES**  or **NO**

If yes, please list the type and brand name. __________________

8). Did you take any prescription medication today? **YES**  or **NO**

If yes, please list the name(s).____________________

9). Did you have any caffeine today? **YES**  or  **NO**

If yes, how much? ____________

How much caffeine do you usually consume? ____________

10). Did you drink any alcohol today? **YES** or **NO**

If yes, how many drinks today? ______

How many drinks do you usually have? ______

11). What did you eat/drink today for lunch? _________________________________

What time did you eat lunch? ___________________

12). What did you eat/drink tonight for dinner/snack? __________________________

What time did you eat dinner/snack? ________________

13). Do you smoke cigarettes or chew tobacco? **YES** or **NO**

If yes, how many packs/pouches today? ____________

How many packs/pouches do you usually have? ___________

14). What is your height? ____________

15). What is your weight? ____________

**Cortisol Background Information (sheet 2)**

Subject ID __________________ Session ____________ Study __________ Date ____/____/____

### Background questions:

How old are you? __________

How tall are you? __________

How much do you weigh? __________

What is your profession or what do you study? __________

What is your highest educational level? __________

| 1. Do you smoke?   If yes, how much? | **YES** | **NO** |  |
| --- | --- | --- | --- |

**Do you have or had any of the following health complaints or diseases?**

| 1. Do you have diabetes? (high blood sugar) | **YES** | **NO** |  |
| --- | --- | --- | --- |
| 1. Do you have any other metabolic diseases? (such as Addison’s disease…) | **YES** | **NO** |  |
| 4. Do you have a hormonal disease? (such as thyroid disease..) | **YES** | **NO** |  |
| 5. Do you take any hormonal medication? (such as Prednisolone, or any hydrocortisone or hormonal components…) | **YES** | **NO** |  |
| 6. Do you have any allergic disease? (such as to food or bees … ) | **YES** | **NO** |  |
| 7. Do you take any allergy medication? | **YES** | **NO** |  |
| 8. Do you use any medication or creams containing cortisone? | **YES** | **NO** |  |
| 9. Do you have high or low blood pressure? | **YES** | **NO** |  |
| 10. Do you have heart problems? | **YES** | **NO** |  |
| 11. Do you sometimes have shortness of breath in the absence of physical exercise? | **YES** | **NO** |  |
| 17. Do you use any other kind of medication? | **YES** | **NO** |  |
| 18. Have you had the flu or a cold within the last four weeks? | **YES** | **NO** |  |
| 19. Have you had a very stressful life event within the last 2 months?  (e.g. accident, death of a family member or friend, divorce etc.) | **YES** | **NO** |  |
|  |  |  |  |

How many hours of sleep do you normally get?

What time do you normally go to bed?

What time do you normally get up?

| Do you drink alcohol?  If yes, how often? | **YES** | **NO** |  |
| --- | --- | --- | --- |
| Do you drink more alcohol than you want to?  If yes, how often? | **YES** | **NO** |  |

How much do you drink per week? _____________________________________

Post Class Teacher Questionnaire

# Post Class Teacher Questionnaire

Class Date: ____________

Teacher’s Name: ___________________________________

Please check all that apply.

In relation to the course guidelines, during today’s class have there been any changes to:

□ Class Length

□ Content Order

□ Overall Content

Please give a detailed description of any changes that were made during today’s class in the space provided:

|  |
| --- |
|  |
|  |
|  |
|  |
|  |
|  |
|  |
|  |
|  |

4-month Practice Log (HEP, cohort #1) (v6/12/06)

Dear Participant:

What matters most about this log is that you are honest in completing it. Your instructor will not receive this information. We hope you will do what you wish to do to improve your own well-being. If that means taking a break or never practicing again, that is fine. What is important is that you record what you actually have or have not done. The information you provide will be more accurate if you take a moment each day to complete this log. However, if you forget to do so, please fill in days as soon as you can and let us know that those days were based on recall in the “comments” section.

For each day, please record the NUMBER of MINUTES you practice each day. If you practice more than once, enter this as a separate total (minutes) on the same day. For example, if you practiced physical activity for 20 minutes in the morning and 20 minutes in the evening on 10/20/06, you would enter “20, 20” in the 10/20/06 row, Physical Activity column. If you engaged in a different practice, please follow the same procedure but briefly note in the “other” column the name of the practice.

| **Date** | **Physical Activity** | **Functional Movement** | **Music Therapy** | **Nutrition** | **Other (specify)** | **Comments** |
| --- | --- | --- | --- | --- | --- | --- |
| 10/20/06 |  |  |  |  |  |  |
| 10/21/06 |  |  |  |  |  |  |
| 10/22/06 |  |  |  |  |  |  |
| 10/23/06 |  |  |  |  |  |  |
| 10/24/06 |  |  |  |  |  |  |
| 10/25/06 |  |  |  |  |  |  |
| 10/26/06 |  |  |  |  |  |  |
| 10/27/06 |  |  |  |  |  |  |
| 10/28/06 |  |  |  |  |  |  |
| 10/29/06 |  |  |  |  |  |  |
| 10/30/06 |  |  |  |  |  |  |
| 10/31/06 |  |  |  |  |  |  |

**For each day, please record the NUMBER of MINUTES you practice each day. If you practice more than once, enter this as a separate total (minutes) on the same day.**

| Date | Physical Activity | Functional Movement | Music Therapy | Nutrition | Other (specify) | Comments |
| --- | --- | --- | --- | --- | --- | --- |
| 11/1/06 |  |  |  |  |  |  |
| 11/2/06 |  |  |  |  |  |  |
| 11/3/06 |  |  |  |  |  |  |
| 11/4/06 |  |  |  |  |  |  |
| 11/5/06 |  |  |  |  |  |  |
| 11/6/06 |  |  |  |  |  |  |
| 11/7/06 |  |  |  |  |  |  |
| 11/8/06 |  |  |  |  |  |  |
| 11/9/06 |  |  |  |  |  |  |
| 11/10/06 |  |  |  |  |  |  |
| 11/11/06 |  |  |  |  |  |  |
| 11/12/06 |  |  |  |  |  |  |
| 11/13/06 |  |  |  |  |  |  |
| 11/14/06 |  |  |  |  |  |  |
| 11/15/06 |  |  |  |  |  |  |
| 11/16/06 |  |  |  |  |  |  |
| 11/17/06 |  |  |  |  |  |  |
| 11/18/06 |  |  |  |  |  |  |
| 11/19/06 |  |  |  |  |  |  |
| 11/20/06 |  |  |  |  |  |  |
| 11/21/06 |  |  |  |  |  |  |
| 11/22/06 |  |  |  |  |  |  |
| 11/23/06 |  |  |  |  |  |  |
| 11/24/06 |  |  |  |  |  |  |
| 11/25/06 |  |  |  |  |  |  |
| 11/26/06 |  |  |  |  |  |  |
| 11/27/06 |  |  |  |  |  |  |
| 11/28/06 |  |  |  |  |  |  |
| 11/29/06 |  |  |  |  |  |  |
| 11/30/06 |  |  |  |  |  |  |

**For each day, please record the NUMBER of MINUTES you practice each day. If you practice more than once, enter this as a separate total (minutes) on the same day.**

| **Date** | **Physical Activity** | **Functional Movement** | **Music Therapy** | **Nutrition** | **Other (specify)** | **Comments** |
| --- | --- | --- | --- | --- | --- | --- |
| 12/1/06 |  |  |  |  |  |  |
| 12/2/06 |  |  |  |  |  |  |
| 12/3/06 |  |  |  |  |  |  |
| 12/4/06 |  |  |  |  |  |  |
| 12/5/06 |  |  |  |  |  |  |
| 12/6/06 |  |  |  |  |  |  |
| 12/7/06 |  |  |  |  |  |  |
| 12/8/06 |  |  |  |  |  |  |
| 12/9/06 |  |  |  |  |  |  |
| 12/10/06 |  |  |  |  |  |  |
| 12/11/06 |  |  |  |  |  |  |
| 12/12/06 |  |  |  |  |  |  |
| 12/13/06 |  |  |  |  |  |  |
| 12/14/06 |  |  |  |  |  |  |
| 12/15/06 |  |  |  |  |  |  |
| 12/16/06 |  |  |  |  |  |  |
| 12/17/06 |  |  |  |  |  |  |
| 12/18/06 |  |  |  |  |  |  |
| 12/19/06 |  |  |  |  |  |  |
| 12/20/06 |  |  |  |  |  |  |
| 12/21/06 |  |  |  |  |  |  |
| 12/22/06 |  |  |  |  |  |  |
| 12/23/06 |  |  |  |  |  |  |
| 12/24/06 |  |  |  |  |  |  |
| 12/25/06 |  |  |  |  |  |  |
| 12/26/06 |  |  |  |  |  |  |
| 12/27/06 |  |  |  |  |  |  |
| 12/28/06 |  |  |  |  |  |  |
| 12/29/06 |  |  |  |  |  |  |
| 12/30/06 |  |  |  |  |  |  |
| 12/31/06 |  |  |  |  |  |  |

**For each day, please record the NUMBER of MINUTES you practice each day. If you practice more than once, enter this as a separate total (minutes) on the same day.**

| **Date** | **Physical Activity** | **Functional Movement** | **Music Therapy** | **Nutrition** | **Other (specify)** | **Comments** |
| --- | --- | --- | --- | --- | --- | --- |
| 1/1/07 |  |  |  |  |  |  |
| 1/2/07 |  |  |  |  |  |  |
| 1/3/07 |  |  |  |  |  |  |
| 1/4/07 |  |  |  |  |  |  |
| 1/5/07 |  |  |  |  |  |  |
| 1/6/07 |  |  |  |  |  |  |
| 1/7/07 |  |  |  |  |  |  |
| 1/8/07 |  |  |  |  |  |  |
| 1/9/07 |  |  |  |  |  |  |
| 1/10/07 |  |  |  |  |  |  |
| 1/11/07 |  |  |  |  |  |  |
| 1/12/07 |  |  |  |  |  |  |
| 1/13/07 |  |  |  |  |  |  |
| 1/14/07 |  |  |  |  |  |  |
| 1/15/07 |  |  |  |  |  |  |
| 1/16/07 |  |  |  |  |  |  |
| 1/17/07 |  |  |  |  |  |  |
| 1/18/07 |  |  |  |  |  |  |
| 1/19/07 |  |  |  |  |  |  |
| 1/20/07 |  |  |  |  |  |  |
| 1/21/07 |  |  |  |  |  |  |
| 1/22/07 |  |  |  |  |  |  |
| 1/23/07 |  |  |  |  |  |  |
| 1/24/07 |  |  |  |  |  |  |
| 1/25/07 |  |  |  |  |  |  |
| 1/26/07 |  |  |  |  |  |  |
| 1/27/07 |  |  |  |  |  |  |
| 1/28/07 |  |  |  |  |  |  |
| 1/29/07 |  |  |  |  |  |  |
| 1/30/07 |  |  |  |  |  |  |
| 1/31/07 |  |  |  |  |  |  |

**For each day, please record the NUMBER of MINUTES you practice each day. If you practice more than once, enter this as a separate total (minutes) on the same day.**

| **Date** | **Physical Activity** | **Functional Movement** | **Music Therapy** | **Nutrition** | **Other (specify)** | | **Comments** |
| --- | --- | --- | --- | --- | --- | --- | --- |
| 2/1/07 |  |  |  |  |  | |  |
| 2/2/07 |  |  |  |  |  | |  |
| 2/3/07 |  |  |  |  |  | |  |
| 2/4/07 |  |  |  |  |  | |  |
| 2/5/07 |  |  |  |  |  | |  |
| 2/6/07 |  |  |  |  |  | |  |
| 2/7/07 |  |  |  |  |  | |  |
| 2/8/07 |  |  |  |  |  | |  |
| 2/9/07 |  |  |  |  |  | |  |
| 2/10/07 |  |  |  |  |  | |  |
| 2/11/07 |  |  |  |  |  | |  |
| 2/12/07 |  |  |  |  |  | |  |
| 2/13/07 |  |  |  |  |  | |  |
| 2/14/07 |  |  |  |  |  | |  |
| 2/15/07 |  |  |  |  |  | |  |
| 2/16/07 |  |  |  |  |  |  | |
| 2/17/07 |  |  |  |  |  |  | |
| 2/18/07 |  |  |  |  |  |  | |
| 2/19/07 |  |  |  |  |  |  | |
| 2/20/07 |  |  |  |  |  |  | |
| 2/21/07 |  |  |  |  |  |  | |
| 2/22/07 |  |  |  |  |  |  | |
| 2/23/07 |  |  |  |  |  |  | |
| 2/24/07 |  |  |  |  |  |  | |
| 2/25/07 |  |  |  |  |  |  | |
| 2/26/07 |  |  |  |  |  |  | |
| 2/27/07 |  |  |  |  |  |  | |
| 2/28/07 |  |  |  |  |  |  | |

**For each day, please record the NUMBER of MINUTES you practice each day. If you practice more than once, enter this as a separate total (minutes) on the same day.**

| **Date** | **Physical Activity** | **Functional Movement** | **Music Therapy** | **Nutrition** | **Other (specify)** | **Comments** |
| --- | --- | --- | --- | --- | --- | --- |
| 3/1/07 |  |  |  |  |  |  |
| 3/2/07 |  |  |  |  |  |  |
| 3/3/07 |  |  |  |  |  |  |
| 3/4/07 |  |  |  |  |  |  |
| 3/5/07 |  |  |  |  |  |  |
| 3/6/07 |  |  |  |  |  |  |
| 3/7/07 |  |  |  |  |  |  |
| 3/8/07 |  |  |  |  |  |  |
| 3/9/07 |  |  |  |  |  |  |
| 3/10/07 |  |  |  |  |  |  |
| 3/11/07 |  |  |  |  |  |  |
| 3/12/07 |  |  |  |  |  |  |
| 3/13/07 |  |  |  |  |  |  |
| 3/14/07 |  |  |  |  |  |  |
| 3/15/06 |  |  |  |  |  |  |

On average, I recorded this information (circle one): Daily Weekly Monthly Just before I turned it in

How accurately does this log reflect your actual practice (circle # below):

Not at All Poorly Not very well Ok Pretty well Very well Perfect

1 2 3 4 5 6 7

Comments: ______________________________________________________________________________

______________________________________________________________________________

______________________________________________________________________________

______________________________________________________________________________

### THE INSTRUCTOR WILL NOT SEE YOUR RESPONSES

4-month Practice Log (HEP, cohort #2) (v6/12/06)

Dear Participant:

What matters most about this log is that you are honest in completing it. Your instructor will not receive this information. We hope you will do what you wish to do to improve your own well-being. If that means taking a break or never practicing again, that is fine. What is important is that you record what you actually have or have not done. The information you provide will be more accurate if you take a moment each day to complete this log. However, if you forget to do so, please fill in days as soon as you can and let us know that those days were based on recall in the “comments” section.

For each day, please record the NUMBER of MINUTES you practice each day. If you practice more than once, enter this as a separate total (minutes) on the same day. For example, if you practiced physical activity for 20 minutes in the morning and 20 minutes in the evening on 11/14/06, you would enter “20, 20” in the 11/14/06 row, Physical Activity column. If you engaged in a different practice, please follow the same procedure but briefly note in the “other” column the name of the practice.

| **Date** | **Physical Activity** | **Functional Movement** | **Music Therapy** | **Nutrition** | **Other (specify)** | **Comments** |
| --- | --- | --- | --- | --- | --- | --- |
| 11/14/06 |  |  |  |  |  |  |
| 11/15/06 |  |  |  |  |  |  |
| 11/16/06 |  |  |  |  |  |  |
| 11/17/06 |  |  |  |  |  |  |
| 11/18/06 |  |  |  |  |  |  |
| 11/19/06 |  |  |  |  |  |  |
| 11/20/06 |  |  |  |  |  |  |
| 11/21/06 |  |  |  |  |  |  |
| 11/22/06 |  |  |  |  |  |  |
| 11/23/06 |  |  |  |  |  |  |
| 11/24/06 |  |  |  |  |  |  |
| 11/25/06 |  |  |  |  |  |  |
| 11/26/06 |  |  |  |  |  |  |
| 11/27/06 |  |  |  |  |  |  |
| 11/28/06 |  |  |  |  |  |  |
| 11/29/06 |  |  |  |  |  |  |
| 11/30/06 |  |  |  |  |  |  |

**Foor each day, please record the NUMBER of MINUTES you practice each day. If you practice more than once, enter this as a separate total (minutes) on the same day.**

| **Date** | **Physical Activity** | **Functional Movement** | **Music Therapy** | **Nutrition** | **Other (specify)** | **Comments** |
| --- | --- | --- | --- | --- | --- | --- |
| 12/1/06 |  |  |  |  |  |  |
| 12/2/06 |  |  |  |  |  |  |
| 12/3/06 |  |  |  |  |  |  |
| 12/4/06 |  |  |  |  |  |  |
| 12/5/06 |  |  |  |  |  |  |
| 12/6/06 |  |  |  |  |  |  |
| 12/7/06 |  |  |  |  |  |  |
| 12/8/06 |  |  |  |  |  |  |
| 12/9/06 |  |  |  |  |  |  |
| 12/10/06 |  |  |  |  |  |  |
| 12/11/06 |  |  |  |  |  |  |
| 12/12/06 |  |  |  |  |  |  |
| 12/13/06 |  |  |  |  |  |  |
| 12/14/06 |  |  |  |  |  |  |
| 12/15/06 |  |  |  |  |  |  |
| 12/16/06 |  |  |  |  |  |  |
| 12/17/06 |  |  |  |  |  |  |
| 12/18/06 |  |  |  |  |  |  |
| 12/19/06 |  |  |  |  |  |  |
| 12/20/06 |  |  |  |  |  |  |
| 12/21/06 |  |  |  |  |  |  |
| 12/22/06 |  |  |  |  |  |  |
| 12/23/06 |  |  |  |  |  |  |
| 12/24/06 |  |  |  |  |  |  |
| 12/25/06 |  |  |  |  |  |  |
| 12/26/06 |  |  |  |  |  |  |
| 12/27/06 |  |  |  |  |  |  |
| 12/28/06 |  |  |  |  |  |  |
| 12/29/06 |  |  |  |  |  |  |
| 12/30/06 |  |  |  |  |  |  |
| 12/31/06 |  |  |  |  |  |  |

**For each day, please record the NUMBER of MINUTES you practice each day. If you practice more than once, enter this as a separate total (minutes) on the same day.**

| **Date** | **Physical Activity** | **Functional Movement** | **Music Therapy** | **Nutrition** | **Other (specify)** | **Comments** |
| --- | --- | --- | --- | --- | --- | --- |
| 1/1/07 |  |  |  |  |  |  |
| 1/2/07 |  |  |  |  |  |  |
| 1/3/07 |  |  |  |  |  |  |
| 1/4/07 |  |  |  |  |  |  |
| 1/5/06 |  |  |  |  |  |  |
| 1/6/07 |  |  |  |  |  |  |
| 1/7/07 |  |  |  |  |  |  |
| 1/8/07 |  |  |  |  |  |  |
| 1/9/07 |  |  |  |  |  |  |
| 1/10/07 |  |  |  |  |  |  |
| 1/11/07 |  |  |  |  |  |  |
| 1/12/07 |  |  |  |  |  |  |
| 1/13/07 |  |  |  |  |  |  |
| 1/14/07 |  |  |  |  |  |  |
| 1/15/07 |  |  |  |  |  |  |
| 1/16/07 |  |  |  |  |  |  |
| 1/17/07 |  |  |  |  |  |  |
| 1/18/07 |  |  |  |  |  |  |
| 1/19/07 |  |  |  |  |  |  |
| 1/20/07 |  |  |  |  |  |  |
| 1/21/07 |  |  |  |  |  |  |
| 1/22/07 |  |  |  |  |  |  |
| 1/23/07 |  |  |  |  |  |  |
| 1/24/07 |  |  |  |  |  |  |
| 1/25/07 |  |  |  |  |  |  |
| 1/26/07 |  |  |  |  |  |  |
| 1/27/07 |  |  |  |  |  |  |
| 1/28/07 |  |  |  |  |  |  |
| 1/29/07 |  |  |  |  |  |  |
| 1/30/07 |  |  |  |  |  |  |
| 1/31/07 |  |  |  |  |  |  |

**For each day, please record the NUMBER of MINUTES you practice each day. If you practice more than once, enter this as a separate total (minutes) on the same day.**

| **Date** | **Physical Activity** | **Functional Movement** | **Music Therapy** | **Nutrition** | **Other (specify)** | **Comments** |
| --- | --- | --- | --- | --- | --- | --- |
| 2/1/07 |  |  |  |  |  |  |
| 2/2/07 |  |  |  |  |  |  |
| 2/3/07 |  |  |  |  |  |  |
| 2/4/07 |  |  |  |  |  |  |
| 2/5/07 |  |  |  |  |  |  |
| 2/6/07 |  |  |  |  |  |  |
| 2/7/07 |  |  |  |  |  |  |
| 2/8/07 |  |  |  |  |  |  |
| 2/9/07 |  |  |  |  |  |  |
| 2/10/07 |  |  |  |  |  |  |
| 2/11/07 |  |  |  |  |  |  |
| 2/12/07 |  |  |  |  |  |  |
| 2/13/07 |  |  |  |  |  |  |
| 2/14/07 |  |  |  |  |  |  |
| 2/15/07 |  |  |  |  |  |  |
| 2/16/07 |  |  |  |  |  |  |
| 2/17/07 |  |  |  |  |  |  |
| 2/18/07 |  |  |  |  |  |  |
| 2/19/07 |  |  |  |  |  |  |
| 2/20/06 |  |  |  |  |  |  |
| 2/21/06 |  |  |  |  |  |  |
| 2/22/06 |  |  |  |  |  |  |
| 2/23/06 |  |  |  |  |  |  |
| 2/24/06 |  |  |  |  |  |  |
| 2/25/06 |  |  |  |  |  |  |
| 2/26/06 |  |  |  |  |  |  |
| 2/27/06 |  |  |  |  |  |  |
| 2/28/06 |  |  |  |  |  |  |

**For each day, please record the NUMBER of MINUTES you practice each day. If you practice more than once, enter this as a separate total (minutes) on the same day.**

| Date | Physical Activity | Functional Movement | Music Therapy | Nutrition | Other (specify) | Comments |
| --- | --- | --- | --- | --- | --- | --- |
| 3/1/07 |  |  |  |  |  |  |
| 3/2/07 |  |  |  |  |  |  |
| 3/3/07 |  |  |  |  |  |  |
| 3/4/07 |  |  |  |  |  |  |
| 3/5/07 |  |  |  |  |  |  |
| 3/6/07 |  |  |  |  |  |  |
| 3/7/07 |  |  |  |  |  |  |
| 3/8/07 |  |  |  |  |  |  |
| 3/9/07 |  |  |  |  |  |  |
| 3/10/07 |  |  |  |  |  |  |
| 3/11/07 |  |  |  |  |  |  |
| 3/12/07 |  |  |  |  |  |  |
| 3/13/07 |  |  |  |  |  |  |
| 3/14/07 |  |  |  |  |  |  |
| 3/15/07 |  |  |  |  |  |  |
| 3/16/07 |  |  |  |  |  |  |
| 3/17/07 |  |  |  |  |  |  |
| 3/18/07 |  |  |  |  |  |  |
| 3/19/07 |  |  |  |  |  |  |
| 3/20/07 |  |  |  |  |  |  |
| 3/21/07 |  |  |  |  |  |  |
| 3/22/07 |  |  |  |  |  |  |
| 3/23/07 |  |  |  |  |  |  |
| 3/24/07 |  |  |  |  |  |  |
| 3/25/07 |  |  |  |  |  |  |
| 3/26/07 |  |  |  |  |  |  |
| 3/27/07 |  |  |  |  |  |  |
| 3/28/07 |  |  |  |  |  |  |
| 3/29/07 |  |  |  |  |  |  |
| 3/30/07 |  |  |  |  |  |  |
| 3/31/07 |  |  |  |  |  |  |

**For each day, please record the NUMBER of MINUTES you practice each day. If you practice more than once, enter this as a separate total (minutes) on the same day.**

| **Date** | **Physical Activity** | | **Functional Movement** | | | **Music Therapy** | | **Nutrition** | | **Other (specify)** | | **Comments** |
| --- | --- | --- | --- | --- | --- | --- | --- | --- | --- | --- | --- | --- |
| 4/1/07 | |  | |  |  | |  | |  | |  | |
| 4/2/07 | |  | |  |  | |  | |  | |  | |
| 4/3/07 | |  | |  |  | |  | |  | |  | |
| 4/4/07 | |  | |  |  | |  | |  | |  | |
| 4/5/07 | |  | |  |  | |  | |  | |  | |
| 4/6/07 | |  | |  |  | |  | |  | |  | |
| 4/7/07 | |  | |  |  | |  | |  | |  | |
| 4/8/07 | |  | |  |  | |  | |  | |  | |
| 4/9/07 | |  | |  |  | |  | |  | |  | |

On average, I recorded this information (circle one): Daily Weekly Monthly Just before I turned it in

How accurately does this log reflect your actual practice (circle # below):

Not at All Poorly Not very well Ok Pretty well Very well Perfect

1 2 3 4 5 6 7

Comments: ______________________________________________________________________________

______________________________________________________________________________

______________________________________________________________________________

______________________________________________________________________________

### THE INSTRUCTOR WILL NOT SEE YOUR RESPONSES

4-month Practice Log (MBSR, cohort #1) (v6/12/06)

Dear Participant:

What matters most about this log is that you are honest in completing it. Your instructor will not receive this information. We hope you will do what you wish to do to improve your own well-being. If that means taking a break or never practicing again, that is fine. What is important is that you record what you actually have or have not done. The information you provide will be more accurate if you take a moment each day to complete this log. However, if you forget to do so, please fill in days as soon as you can and let us know that those days were based on recall in the “comments” section.

For each day, please record the NUMBER of MINUTES you practice each day. If you practice more than once, enter this as a separate total (minutes) on the same day. For example, if you practiced the body scan for 45 minutes in the morning and 45 minutes in the evening on 10/19/06, you would enter “45, 45” in the 10/19/06 row, Body Scan column. If you engaged in a different practice, please follow the same procedure but briefly note in the “other” column the name of the practice.

| **Date** | **Sitting Meditation** | **Body Scan** | **Yoga** | **Mindful Walking** | **Other (specify)** | **Comments** |
| --- | --- | --- | --- | --- | --- | --- |
| 10/19/06 |  |  |  |  |  |  |
| 10/20/06 |  |  |  |  |  |  |
| 10/21/06 |  |  |  |  |  |  |
| 10/22/06 |  |  |  |  |  |  |
| 10/23/06 |  |  |  |  |  |  |
| 10/24/06 |  |  |  |  |  |  |
| 10/25/06 |  |  |  |  |  |  |
| 10/26/06 |  |  |  |  |  |  |
| 10/27/06 |  |  |  |  |  |  |
| 10/28/06 |  |  |  |  |  |  |
| 10/29/06 |  |  |  |  |  |  |
| 10/30/06 |  |  |  |  |  |  |
| 10/31/06 |  |  |  |  |  |  |

**For each day, please record the NUMBER of MINUTES you practice each day. If you practice more than once, enter this as a separate total (minutes) on the same day.**

| **Date** | **Sitting Meditation** | **Body Scan** | **Yoga** | **Mindful Walking** | **Other (specify)** | **Comments** |
| --- | --- | --- | --- | --- | --- | --- |
| 11/1/06 |  |  |  |  |  |  |
| 11/2/06 |  |  |  |  |  |  |
| 11/3/06 |  |  |  |  |  |  |
| 11/4/06 |  |  |  |  |  |  |
| 11/5/06 |  |  |  |  |  |  |
| 11/6/06 |  |  |  |  |  |  |
| 11/7/06 |  |  |  |  |  |  |
| 11/8/06 |  |  |  |  |  |  |
| 11/9/06 |  |  |  |  |  |  |
| 11/10/06 |  |  |  |  |  |  |
| 11/11/06 |  |  |  |  |  |  |
| 11/12/06 |  |  |  |  |  |  |
| 11/13/06 |  |  |  |  |  |  |
| 11/14/06 |  |  |  |  |  |  |
| 11/15/06 |  |  |  |  |  |  |
| 11/16/06 |  |  |  |  |  |  |
| 11/17/06 |  |  |  |  |  |  |
| 11/18/06 |  |  |  |  |  |  |
| 11/19/06 |  |  |  |  |  |  |
| 11/20/06 |  |  |  |  |  |  |
| 11/21/06 |  |  |  |  |  |  |
| 11/22/06 |  |  |  |  |  |  |
| 11/23/06 |  |  |  |  |  |  |
| 11/24/06 |  |  |  |  |  |  |
| 11/25/06 |  |  |  |  |  |  |
| 11/26/06 |  |  |  |  |  |  |
| 11/27/06 |  |  |  |  |  |  |
| 11/28/06 |  |  |  |  |  |  |
| 11/29/06 |  |  |  |  |  |  |
| 11/30/06 |  |  |  |  |  |  |

**For each day, please record the NUMBER of MINUTES you practice each day. If you practice more than once, enter this as a separate total (minutes) on the same day.**

| **Date** | **Sitting Meditation** | **Body Scan** | **Yoga** | **Mindful Walking** | **Other (specify)** | **Comments** |
| --- | --- | --- | --- | --- | --- | --- |
| 12/1/06 |  |  |  |  |  |  |
| 12/2/06 |  |  |  |  |  |  |
| 12/3/06 |  |  |  |  |  |  |
| 12/4/06 |  |  |  |  |  |  |
| 12/5/06 |  |  |  |  |  |  |
| 12/6/06 |  |  |  |  |  |  |
| 12/7/06 |  |  |  |  |  |  |
| 12/8/06 |  |  |  |  |  |  |
| 12/9/06 |  |  |  |  |  |  |
| 12/10/06 |  |  |  |  |  |  |
| 12/11/06 |  |  |  |  |  |  |
| 12/12/06 |  |  |  |  |  |  |
| 12/13/06 |  |  |  |  |  |  |
| 12/14/06 |  |  |  |  |  |  |
| 12/15/06 |  |  |  |  |  |  |
| 12/16/06 |  |  |  |  |  |  |
| 12/17/06 |  |  |  |  |  |  |
| 12/18/06 |  |  |  |  |  |  |
| 12/19/06 |  |  |  |  |  |  |
| 12/20/06 |  |  |  |  |  |  |
| 12/21/06 |  |  |  |  |  |  |
| 12/22/06 |  |  |  |  |  |  |
| 12/23/06 |  |  |  |  |  |  |
| 12/24/06 |  |  |  |  |  |  |
| 12/25/06 |  |  |  |  |  |  |
| 12/26/06 |  |  |  |  |  |  |
| 12/27/06 |  |  |  |  |  |  |
| 12/28/06 |  |  |  |  |  |  |
| 12/29/06 |  |  |  |  |  |  |
| 12/30/06 |  |  |  |  |  |  |
| 12/31/06 |  |  |  |  |  |  |

**For each day, please record the NUMBER of MINUTES you practice each day. If you practice more than once, enter this as a separate total (minutes) on the same day.**

| **Date** | **Sitting Meditation** | **Body Scan** | **Yoga** | **Mindful Walking** | **Other (specify)** | **Comments** |
| --- | --- | --- | --- | --- | --- | --- |
| 1/1/07 |  |  |  |  |  |  |
| 1/2/07 |  |  |  |  |  |  |
| 1/3/07 |  |  |  |  |  |  |
| 1/4/07 |  |  |  |  |  |  |
| 1/5/07 |  |  |  |  |  |  |
| 1/6/07 |  |  |  |  |  |  |
| 1/7/07 |  |  |  |  |  |  |
| 1/8/07 |  |  |  |  |  |  |
| 1/9/07 |  |  |  |  |  |  |
| 1/10/07 |  |  |  |  |  |  |
| 1/11/07 |  |  |  |  |  |  |
| 1/12/07 |  |  |  |  |  |  |
| 1/13/07 |  |  |  |  |  |  |
| 1/14/07 |  |  |  |  |  |  |
| 1/15/07 |  |  |  |  |  |  |
| 1/16/07 |  |  |  |  |  |  |
| 1/17/07 |  |  |  |  |  |  |
| 1/18/07 |  |  |  |  |  |  |
| 1/19/07 |  |  |  |  |  |  |
| 1/20/07 |  |  |  |  |  |  |
| 1/21/07 |  |  |  |  |  |  |
| 1/22/07 |  |  |  |  |  |  |
| 1/23/07 |  |  |  |  |  |  |
| 1/24/07 |  |  |  |  |  |  |
| 1/25/07 |  |  |  |  |  |  |
| 1/26/07 |  |  |  |  |  |  |
| 1/27/07 |  |  |  |  |  |  |
| 1/28/07 |  |  |  |  |  |  |
| 1/29/07 |  |  |  |  |  |  |
| 1/30/07 |  |  |  |  |  |  |
| 1/31/07 |  |  |  |  |  |  |

**For each day, please record the NUMBER of MINUTES you practice each day. If you practice more than once, enter this as a separate total (minutes) on the same day.**

| **Date** | **Sitting Meditation** | | **Body Scan** | | **Yoga** | | **Mindful Walking** | | **Other (specify)** | | **Comments** |
| --- | --- | --- | --- | --- | --- | --- | --- | --- | --- | --- | --- |
| 2/1/07 | |  |  |  | |  | |  | |  | |
| 2/2/07 | |  |  |  | |  | |  | |  | |
| 2/3/07 | |  |  |  | |  | |  | |  | |
| 2/4/07 | |  |  |  | |  | |  | |  | |
| 2/5/07 | |  |  |  | |  | |  | |  | |
| 2/6/07 | |  |  |  | |  | |  | |  | |
| 2/7/07 | |  |  |  | |  | |  | |  | |
| 2/8/07 | |  |  |  | |  | |  | |  | |
| 2/9/07 | |  |  |  | |  | |  | |  | |
| 2/10/07 | |  |  |  | |  | |  | |  | |
| 2/11/07 | |  |  |  | |  | |  | |  | |
| 2/12/07 | |  |  |  | |  | |  | |  | |
| 2/13/07 | |  |  |  | |  | |  | |  | |
| 2/14/07 | |  |  |  | |  | |  | |  | |
| 2/15/07 | |  |  |  | |  | |  | |  | |
| 2/16/07 | |  |  |  | |  | |  | |  | |
| 2/17/07 | |  |  |  | |  | |  | |  | |
| 2/18/07 | |  |  |  | |  | |  | |  | |
| 2/19/07 | |  |  |  | |  | |  | |  | |
| 2/20/07 | |  |  |  | |  | |  | |  | |
| 2/21/07 | |  |  |  | |  | |  | |  | |
| 2/22/07 | |  |  |  | |  | |  | |  | |
| 2/23/07 | |  |  |  | |  | |  | |  | |
| 2/24/07 | |  |  |  | |  | |  | |  | |
| 2/25/07 | |  |  |  | |  | |  | |  | |
| 2/26/07 | |  |  |  | |  | |  | |  | |
| 2/27/07 | |  |  |  | |  | |  | |  | |
| 2/28/07 | |  |  |  | |  | |  | |  | |

**For each day, please record the NUMBER of MINUTES you practice each day. If you practice more than once, enter this as a separate total (minutes) on the same day.**

| **Date** | **Sitting Meditation** | | **Body Scan** | | | **Yoga** | | **Mindful Walking** | | **Other (specify)** | | **Comments** |
| --- | --- | --- | --- | --- | --- | --- | --- | --- | --- | --- | --- | --- |
| 3/1/07 | |  | |  |  | |  | |  | |  | |
| 3/2/07 | |  | |  |  | |  | |  | |  | |
| 3/3/07 | |  | |  |  | |  | |  | |  | |
| 3/4/07 | |  | |  |  | |  | |  | |  | |
| 3/5/07 | |  | |  |  | |  | |  | |  | |
| 3/6/07 | |  | |  |  | |  | |  | |  | |
| 3/7/07 | |  | |  |  | |  | |  | |  | |
| 3/8/07 | |  | |  |  | |  | |  | |  | |
| 3/9/07 | |  | |  |  | |  | |  | |  | |
| 3/10/07 | |  | |  |  | |  | |  | |  | |
| 3/11/07 | |  | |  |  | |  | |  | |  | |
| 3/12/07 | |  | |  |  | |  | |  | |  | |
| 3/13/07 | |  | |  |  | |  | |  | |  | |
| 3/14/07 | |  | |  |  | |  | |  | |  | |

On average, I recorded this information (circle one): Daily Weekly Monthly Just before I turned it in

How accurately does this log reflect your actual practice (circle # below):

Not at All Poorly Not very well Ok Pretty well Very well Perfect

1 2 3 4 5 6 7

Comments: ______________________________________________________________________________

______________________________________________________________________________

______________________________________________________________________________

______________________________________________________________________________

### THE INSTRUCTOR WILL NOT SEE YOUR RESPONSES

4-month Practice Log (MBSR, cohort #2) (v6/12/06)

Dear Participant:

What matters most about this log is that you are honest in completing it. Your instructor will not receive this information. We hope you will do what you wish to do to improve your own well-being. If that means taking a break or never practicing again, that is fine. What is important is that you record what you actually have or have not done. The information you provide will be more accurate if you take a moment each day to complete this log. However, if you forget to do so, please fill in days as soon as you can and let us know that those days were based on recall in the “comments” section.

For each day, please record the NUMBER of MINUTES you practice each day. If you practice more than once, enter this as a separate total (minutes) on the same day. For example, if you practiced the body scan for 45 minutes in the morning and 45 minutes in the evening on 11/15/06, you would enter “45, 45” in the 11/15/06 row, Body Scan column. If you engaged in a different practice, please follow the same procedure but briefly note in the “other” column the name of the practice.

| **Date** | **Sitting Meditation** | **Body Scan** | **Yoga** | **Mindful Walking** | **Other (specify)** | **Comments** |
| --- | --- | --- | --- | --- | --- | --- |
| 11/15/06 |  |  |  |  |  |  |
| 11/16/06 |  |  |  |  |  |  |
| 11/17/06 |  |  |  |  |  |  |
| 11/18/06 |  |  |  |  |  |  |
| 11/19/06 |  |  |  |  |  |  |
| 11/20/06 |  |  |  |  |  |  |
| 11/21/06 |  |  |  |  |  |  |
| 11/22/06 |  |  |  |  |  |  |
| 11/23/06 |  |  |  |  |  |  |
| 11/24/06 |  |  |  |  |  |  |
| 11/25/06 |  |  |  |  |  |  |
| 11/26/06 |  |  |  |  |  |  |
| 11/27/06 |  |  |  |  |  |  |
| 11/28/06 |  |  |  |  |  |  |
| 11/29/06 |  |  |  |  |  |  |
| 11/30/06 |  |  |  |  |  |  |

**For each day, please record the NUMBER of MINUTES you practice each day. If you practice more than once, enter this as a separate total (minutes) on the same day.**

| **Date** | **Sitting Meditation** | **Body Scan** | **Yoga** | **Mindful Walking** | **Other (specify)** | **Comments** |
| --- | --- | --- | --- | --- | --- | --- |
| 12/1/06 |  |  |  |  |  |  |
| 12/2/06 |  |  |  |  |  |  |
| 12/3/06 |  |  |  |  |  |  |
| 12/4/06 |  |  |  |  |  |  |
| 12/5/06 |  |  |  |  |  |  |
| 12/6/06 |  |  |  |  |  |  |
| 12/7/06 |  |  |  |  |  |  |
| 12/8/06 |  |  |  |  |  |  |
| 12/9/06 |  |  |  |  |  |  |
| 12/10/06 |  |  |  |  |  |  |
| 12/11/06 |  |  |  |  |  |  |
| 12/12/06 |  |  |  |  |  |  |
| 12/13/06 |  |  |  |  |  |  |
| 12/14/06 |  |  |  |  |  |  |
| 12/15/06 |  |  |  |  |  |  |
| 12/16/06 |  |  |  |  |  |  |
| 12/17/06 |  |  |  |  |  |  |
| 12/18/06 |  |  |  |  |  |  |
| 12/19/06 |  |  |  |  |  |  |
| 12/20/06 |  |  |  |  |  |  |
| 12/21/06 |  |  |  |  |  |  |
| 12/22/06 |  |  |  |  |  |  |
| 12/23/06 |  |  |  |  |  |  |
| 12/24/06 |  |  |  |  |  |  |
| 12/25/06 |  |  |  |  |  |  |
| 12/26/06 |  |  |  |  |  |  |
| 12/27/06 |  |  |  |  |  |  |
| 12/28/06 |  |  |  |  |  |  |
| 12/29/06 |  |  |  |  |  |  |
| 12/30/06 |  |  |  |  |  |  |
| 12/31/06 |  |  |  |  |  |  |

**For each day, please record the NUMBER of MINUTES you practice each day. If you practice more than once, enter this as a separate total (minutes) on the same day.**

| **Date** | **Sitting Meditation** | **Body Scan** | **Yoga** | **Mindful Walking** | **Other (specify)** | **Comments** |
| --- | --- | --- | --- | --- | --- | --- |
| 1/1/07 |  |  |  |  |  |  |
| 1/2/07 |  |  |  |  |  |  |
| 1/3/07 |  |  |  |  |  |  |
| 1/4/07 |  |  |  |  |  |  |
| 1/5/07 |  |  |  |  |  |  |
| 1/6/07 |  |  |  |  |  |  |
| 1/7/07 |  |  |  |  |  |  |
| 1/8/07 |  |  |  |  |  |  |
| 1/9/07 |  |  |  |  |  |  |
| 1/10/07 |  |  |  |  |  |  |
| 1/11/07 |  |  |  |  |  |  |
| 1/12/07 |  |  |  |  |  |  |
| 1/13/07 |  |  |  |  |  |  |
| 1/14/07 |  |  |  |  |  |  |
| 1/15/07 |  |  |  |  |  |  |
| 1/16/07 |  |  |  |  |  |  |
| 1/17/07 |  |  |  |  |  |  |
| 1/18/07 |  |  |  |  |  |  |
| 1/19/07 |  |  |  |  |  |  |
| 1/20/07 |  |  |  |  |  |  |
| 1/21/07 |  |  |  |  |  |  |
| 1/22/07 |  |  |  |  |  |  |
| 1/23/07 |  |  |  |  |  |  |
| 1/24/07 |  |  |  |  |  |  |
| 1/25/07 |  |  |  |  |  |  |
| 1/26/07 |  |  |  |  |  |  |
| 1/27/07 |  |  |  |  |  |  |
| 1/28/07 |  |  |  |  |  |  |
| 1/29/07 |  |  |  |  |  |  |
| 1/30/07 |  |  |  |  |  |  |
| 1/31/07 |  |  |  |  |  |  |

**For each day, please record the NUMBER of MINUTES you practice each day. If you practice more than once, enter this as a separate total (minutes) on the same day.**

| **Date** | **Sitting Meditation** | | **Body Scan** | | **Yoga** | | **Mindful Walking** | | **Other (specify)** | | **Comments** |
| --- | --- | --- | --- | --- | --- | --- | --- | --- | --- | --- | --- |
| 2/1/07 | |  |  |  | |  | |  | |  | |
| 2/2/07 | |  |  |  | |  | |  | |  | |
| 2/3/07 | |  |  |  | |  | |  | |  | |
| 2/4/07 | |  |  |  | |  | |  | |  | |
| 2/5/07 | |  |  |  | |  | |  | |  | |
| 2/6/07 | |  |  |  | |  | |  | |  | |
| 2/7/07 | |  |  |  | |  | |  | |  | |
| 2/8/07 | |  |  |  | |  | |  | |  | |
| 2/9/07 | |  |  |  | |  | |  | |  | |
| 2/10/07 | |  |  |  | |  | |  | |  | |
| 2/11/07 | |  |  |  | |  | |  | |  | |
| 2/12/07 | |  |  |  | |  | |  | |  | |
| 2/13/07 | |  |  |  | |  | |  | |  | |
| 2/14/07 | |  |  |  | |  | |  | |  | |
| 2/15/07 | |  |  |  | |  | |  | |  | |
| 2/16/07 | |  |  |  | |  | |  | |  | |
| 2/17/07 | |  |  |  | |  | |  | |  | |
| 2/18/07 | |  |  |  | |  | |  | |  | |
| 2/19/07 | |  |  |  | |  | |  | |  | |
| 2/20/06 | |  |  |  | |  | |  | |  | |
| 2/21/06 | |  |  |  | |  | |  | |  | |
| 2/22/06 | |  |  |  | |  | |  | |  | |
| 2/23/06 | |  |  |  | |  | |  | |  | |
| 2/24/06 | |  |  |  | |  | |  | |  | |
| 2/25/06 | |  |  |  | |  | |  | |  | |
| 2/26/06 | |  |  |  | |  | |  | |  | |
| 2/27/06 | |  |  |  | |  | |  | |  | |
| 2/28/06 | |  |  |  | |  | |  | |  | |

**For each day, please record the NUMBER of MINUTES you practice each day. If you practice more than once, enter this as a separate total (minutes) on the same day.**

| **Date** | **Sitting Meditation** | | **Body Scan** | | | **Yoga** | | **Mindful Walking** | | **Other (specify)** | | **Comments** |
| --- | --- | --- | --- | --- | --- | --- | --- | --- | --- | --- | --- | --- |
| 3/1/07 | |  | |  |  | |  | |  | |  | |
| 3/2/07 | |  | |  |  | |  | |  | |  | |
| 3/3/07 | |  | |  |  | |  | |  | |  | |
| 3/4/07 | |  | |  |  | |  | |  | |  | |
| 3/5/07 | |  | |  |  | |  | |  | |  | |
| 3/6/07 | |  | |  |  | |  | |  | |  | |
| 3/7/07 | |  | |  |  | |  | |  | |  | |
| 3/8/07 | |  | |  |  | |  | |  | |  | |
| 3/9/07 | |  | |  |  | |  | |  | |  | |
| 3/10/07 | |  | |  |  | |  | |  | |  | |
| 3/11/07 | |  | |  |  | |  | |  | |  | |
| 3/12/07 | |  | |  |  | |  | |  | |  | |
| 3/13/07 | |  | |  |  | |  | |  | |  | |
| 3/14/07 | |  | |  |  | |  | |  | |  | |
| 3/15/07 | |  | |  |  | |  | |  | |  | |
| 3/16/07 | |  | |  |  | |  | |  | |  | |
| 3/17/07 | |  | |  |  | |  | |  | |  | |
| 3/18/07 | |  | |  |  | |  | |  | |  | |
| 3/19/07 | |  | |  |  | |  | |  | |  | |
| 3/20/07 | |  | |  |  | |  | |  | |  | |
| 3/21/07 | |  | |  |  | |  | |  | |  | |
| 3/22/07 | |  | |  |  | |  | |  | |  | |
| 3/23/07 | |  | |  |  | |  | |  | |  | |
| 3/24/07 | |  | |  |  | |  | |  | |  | |
| 3/25/07 | |  | |  |  | |  | |  | |  | |
| 3/26/07 | |  | |  |  | |  | |  | |  | |
| 3/27/07 | |  | |  |  | |  | |  | |  | |
| 3/28/07 | |  | |  |  | |  | |  | |  | |
| 3/29/07 | |  | |  |  | |  | |  | |  | |
| 3/30/07 | |  | |  |  | |  | |  | |  | |
| 3/31/07 | |  | |  |  | |  | |  | |  | |

**For each day, please record the NUMBER of MINUTES you practice each day. If you practice more than once, enter this as a separate total (minutes) on the same day.**

| **Date** | **Sitting Meditation** | | **Body Scan** | | | **Yoga** | | **Mindful Walking** | | **Other (specify)** | | **Comments** |
| --- | --- | --- | --- | --- | --- | --- | --- | --- | --- | --- | --- | --- |
| 4/1/07 | |  | |  |  | |  | |  | |  | |
| 4/2/07 | |  | |  |  | |  | |  | |  | |
| 4/3/07 | |  | |  |  | |  | |  | |  | |
| 4/4/07 | |  | |  |  | |  | |  | |  | |
| 4/5/07 | |  | |  |  | |  | |  | |  | |
| 4/6/07 | |  | |  |  | |  | |  | |  | |
| 4/7/07 | |  | |  |  | |  | |  | |  | |
| 4/8/07 | |  | |  |  | |  | |  | |  | |
| 4/9/07 | |  | |  |  | |  | |  | |  | |
| 4/10/07 | |  | |  |  | |  | |  | |  | |

On average, I recorded this information (circle one): Daily Weekly Monthly Just before I turned it in

How accurately does this log reflect your actual practice (circle # below):

Not at All Poorly Not very well Ok Pretty well Very well Perfect

1 2 3 4 5 6 7

Comments: ______________________________________________________________________________

______________________________________________________________________________

______________________________________________________________________________

______________________________________________________________________________

### THE INSTRUCTOR WILL NOT SEE YOUR RESPONSES

Home Assignments Log (HEP)

## IDNUM__________________ Date log started __ __/__ __/__ __ __ __ m m d d y y y y

**Date log stopped** __ __/__ __/__ __ __ __ m m d d y y y y

# Home Practice Log (HEP)

Please record the NUMBER of MINUTES you practice each of the following, each day. If you practice more than once, enter this as a separate total (minutes) on the same day. For example, if you practiced the stretching for 20 minutes in the morning and 20 minutes in the evening on Day 2, you would enter “20, 20” in the Day 2, Exercise/Stretching square. Please total the number of minutes for all practice each day.

|  | Day of Class  (do not count in-class practice) | Day 2 | Day 3 | Day 4 | Day 5 | Day 6 | Day 7 |
| --- | --- | --- | --- | --- | --- | --- | --- |
| **Physical Activity** |  |  |  |  |  |  |  |
| **Functional Movement** |  |  |  |  |  |  |  |
| **Music Therapy** |  |  |  |  |  |  |  |
| **Nutrition** |  |  |  |  |  |  |  |
| **TOTALS** |  |  |  |  |  |  |  |

Comments:

______________________________________________________________________________

______________________________________________________________________________

______________________________________________________________________________

______________________________________________________________________________

______________________________________________________________________________

______________________________________________________________________________

______________________________________________________________________________

**AT THE END OF THE SEVEN-DAY PERIOD PLEASE BRING THIS LOG TO CLASS.**

**THE INSTRUCTOR WILL NOT SEE YOUR RESPONSES**

Home Assignments Log (MBSR)

## IDNUM__________________ Date log started __ __/__ __/__ __ __ __ m m d d y y y y

**Date log stopped** __ __/__ __/__ __ __ __ m m d d y y y y

# Home Practice Log (MBSR)

Please record the NUMBER of MINUTES you practice each of the following, each day. If you practice more than once, enter this as a separate total (minutes) on the same day. For example, if you practiced the body scan for 45 minutes in the morning and 45 minutes in the evening on Day 2, you would enter “45, 45” in the Day 2, Body Scan square. Please total the number of minutes for all practice each day.

|  | Day of Class  (do not count in-class practice) | Day 2 | Day 3 | Day 4 | Day 5 | Day 6 | Day 7 |
| --- | --- | --- | --- | --- | --- | --- | --- |
| **Sitting Meditation** |  |  |  |  |  |  |  |
| **Body Scan** |  |  |  |  |  |  |  |
| **Yoga** |  |  |  |  |  |  |  |
| **Mindful**  **Walking** |  |  |  |  |  |  |  |
| **TOTALS** |  |  |  |  |  |  |  |

Comments:

______________________________________________________________________________

______________________________________________________________________________

______________________________________________________________________________

______________________________________________________________________________

______________________________________________________________________________

______________________________________________________________________________

______________________________________________________________________________

**AT THE END OF THE SEVEN-DAY PERIOD PLEASE BRING THIS LOG TO CLASS.**

**THE INSTRUCTOR WILL NOT SEE YOUR RESPONSES**

Make-Up Class Documentation (HEP)

Make-Up Class Documentation

Health Enhancement Program

Teacher’s Name: ________________________

Client’s Name: __________________________

Date of Missed Class _____________ Date of Make-Up Class_____________

Please provide a detailed description of the content of the make-up class including deviations from the content of missed class in the space provided below.

Make-Up Class Documentation (MBSR)

Make-Up Class Documentation

Mindfulness Based Stress Reduction Program

Teacher’s Name: ________________________

Client’s Name: __________________________

Date of Missed Class _____________ Date of Make-Up Class_____________

Please provide a detailed description of the content of the make-up class including deviations from the content of missed class in the space provided below.

Adverse Reactions Protocol

# Adverse Reactions

What should you do in the case of adverse reactions or pre-existing conditions in study participants? Please note that this policy ONLY applies to Davidson projects. "Quick reference" guidelines for the following are available online (https://psyphz.psych.wisc.edu/web/members/misc/Adverse_reactions_quick_reference.pdf) and may be posted in each control room.

## Documentation

You will notice the phrase "Document the occurrence in detail" many times below. Probably the single most important thing you can do when responding to adverse reactions in a study participant is to write down in detail everything you think may be relevant. What happened? What did the subject say or do? How did you respond? With whom did you consult? What did they say? How did you resolve the problem? etc. It is particularly important that you include the date and time of the occurrence, as well any follow-up actions.

Richie, as well as Isa and/or Donna should be notified immediately if adverse reactions arise in a study participant. Donna must be notified for any incident occurring in the Brain Imaging lab and Isa must be notified for any incident occurring in the Brogden lab.  If you are unsure who to send documentation to, send it to both.

| **Richie Davidson** | Brogden: Waisman:  Home: Cell: | 263-2659 265-8189 256-5376 345-8336 | [rjdavids@wisc.edu](mailto:rjdavids@wisc.edu) |
| --- | --- | --- | --- |
| **Donna Cole** | Waisman:  Home: Cell: | 265-8107 837-5809  438-3902 | [dmcole@wisc.edu](mailto:dmcole@wisc.edu) |
| **Isa Dolski** | Brogden:  Home: Cell: | 263-0132 232-1441 446-0932 | [idolski@wisc.edu](mailto:idolski@wisc.edu) |
| **Hyejeen Lee** | Brogden:  Office: Cell: (preferred) | 262-4443 262-1567 628-2178 | [hyejeenlee@wisc.edu](mailto:hyejeenlee@wisc.edu) |
| **Tim Salomons** | Waisman: Home: | 263-1968 442-9913 | [tvsalomons@wisc.edu](mailto:tvsalomons@wisc.edu) |

##

## Responsibilities of Study Supervisors

It is expected that all study supervisors, be they faculty, staff, post-doc, grad or undergrad students, will communicate the information in this document to all of their research assistants. It is also expected that supervisors will meet with each new research assistant to answer questions, and conduct a review session for all research assistants once per semester.

## Assessment

Always take a moment to assess the nature of an adverse reaction. Is it medical or psychological?

### Medical Problems

**Emergencies**

- If the problem is an emergency(e.g., the participant is unconscious, choking, or experiencing a potentially life threatening event), call 911 and terminate the experimental session. Inform Richie, Isa/Donna, and your supervisors immediately. Document the occurrence in detail.

**Severe Physical Distress**

- If the participant is in severe physical distress (such as migraine headache, vomiting, light-headedness), but the problem is not an emergency, end the session. We do not want data from someone who is ill or in pain. Reassure the participant that they will be paid or given credit for their time.
  - If appropriate, ask if they would like to use a phone to contact their doctor or others. Make sure they have a safe way home. If they need a ride home, you can get a free taxi from Union Cab using the "Davidson Lab" account (phone 242-2000; account #8005). Email Isa ([idolski@wisc.edu](mailto:idolski@wisc.edu)) to let her know that Union Cab was used, along with the pick-up and drop-off locations.
  - ***Do not transport a study participant in your own car.***
  - Document the occurrence in detail. Inform Richie and your supervisors within 24 hours.

**Minor Physical Discomfort**

- If the participant is experiencing minor physical discomfort, see if you can help to alleviate the problem (e.g., eye drops, cup of water, allowing the subject to walk around).
  - Subjects who are in the lab for a long period of time often suffer caffeine or nicotine withdrawal. Remember to offer soda or coffee and allow people to take a cigarette break for particularly long studies (e.g., studies that last the entire day).
- Do not give subjects any type of over-the-counter or prescription medication.
- Encourage participants to continue with the session if they are feeling well enough. However, if the participant requests to end the session, do so immediately. Inform them that they will be paid or given credit for their participation.
  - Avoid displaying annoyance at having to end a session or make special provisions for a subject.
- If the subject is in significant physical distress or ends a session, document the occurrence and inform your supervisors, Isa/Donna and Richie by email.
- Also, any time a subject reports skin irritation related to study procedures or when a subject’s skin has been significantly over-abraded, your supervisors and Richie should be informed. Emailing is ok.

**Pre-Existing Medical Conditions**

- Note that different studies in the lab have different exclusionary criteria. These criteria should always be explicitly identified and known by all experimenters and assistants.
- If you learn prior to or while running an experimental session that a subject has a condition that rules out study participation (e.g., pregnancy, high blood pressure, diabetes, etc.), cancel the session.
- If you are not sure whether you should run the session, contact your supervisor, Richie, or others associated with your study. If no one can be reached, continue the session unless you think doing so could be potentially harmful to the participant.  Document in detail what you have learned about the participant’s condition.
- If the session is cancelled, reassure the study participant that they will be compensated (i.e., money or credits) for the time that they have invested.

### Psychological Problems

**Emotional Reaction to Study Procedures**

- Several of our protocols elicit negative emotions in our participants. Occasionally, participants become upset (e.g., tearful or agitated) to the point that they are not calm by the end of the session. For experiments that elicit negative emotions, tell subjects at the outset that it is important to be honest about their emotional reactions, and that if the experiment becomes too upsetting they should feel free to let the experimenter know. After a practice block of pictures or emotional stimuli, ask the subject in a non-suggestive way, "how is it going"? This will give them an opportunity to voice any concerns about the stimuli.
- If a participant becomes emotionally distraught during a session, make sure that they know they can stop the experiment at any time. Depending on the design of the experiment, it may be sufficient to take a break from the procedures rather than stopping altogether.
- Express empathy and kindness toward the participant. Acknowledge that our stimuli and tasks can tap into painful emotions. Indicate that other participants occasionally have the type of reaction that they are experiencing and it is not abnormal.
- Do not assume that just because a person has an emotional reaction that they are incapacitated. Ask the participant if they would like a minute alone or if there is anything that you can do to help. However, it is possible that a subject could be very upset, but too scared or embarrassed to request that the session be terminated. In cases like this, the experimenter may need to make a decision about whether to go on. We should err on the cautious side in most cases. The well-being of our participants is a higher priority than our need to collect data. Offer to make a phone call for them or allow them to use the phone. Possibly suggest that the participant contact a friend to help them through their distress. Possibly offer a free cab ride ([see above for procedure](https://psyphz.psych.wisc.edu/web/members/help/adverse.html#cab)).
- Let the participant know that your supervisor or Richie (i.e., "Dr. Davidson, the director of the lab") would be glad to talk to them about their experiences. If the participant would like to talk to Richie or others, find out at what phone number they would like to be called. (If you know that Richie or another lab member won’t be able to call soon, for instance if they are out of town, indicate this to the participant.)
  - Do not give home phone numbers to subjects.
  - If the person indicates that their reaction is related to ongoing stress in their life, it may be appropriate to offer a Community Sources for Psychological Treatment list (available in control rooms or in the lab "Members" section at [http://psyphz.psych.wisc.edu/members/help/comm_trtmt.html](https://psyphz.psych.wisc.edu/web/members/help/comm_trtmt.html) - login using your NetID and password).
- Document the occurrence and inform your supervisors. If the session was ended early, inform Richie and Isa/Donna by email.
- The study coordinator should determine (in consultation with Richie) whether a follow-up call is in order. Should such a call be made, it is important to respect the privacy of study participants. We do not want roommates, spouses, etc. to know that a call is coming from the Psychology department; rather, identify yourself as being "from the University." Unless you know the study participant lives alone, do not leave an answering machine message with sensitive information. If the study participant endorsed [suicidal ideation](https://psyphz.psych.wisc.edu/web/members/help/adverse.html#suicide) DO NOT make a follow-up call. The clinical student who conducted the assessment will make the decision regarding future contacts.

**Suicidal Ideation and Psychopathology**

- At times, we become aware of suicidal ideation in study participants or candidates for studies. This occurs most frequently when screening depressed individuals.
- In studies where the probability is high that we will come into contact with suicidal individuals, a treating clinician will always be associated with the study and emergency procedures will be precisely defined.
- However, the potential exists that we would learn about suicidal ideation in an unselected subject population. In these studies, where suicidality is not expected, the most likely way we would learn that a subject is suicidal is from their Beck Depression Inventory (BDI) or from the MASQ. The BDI and MASQ are self-report instruments administered as part of many of the studies in the lab, and have items about suicidal ideation (item # 9 on BDI-2, item #61 on the MASQ-short form, and item #89 on the MASQ-standard form). These items should always be checked while the subject is in the lab. Depending on the subject’s responses to these items, further action may be required (see below for [instructions](https://psyphz.psych.wisc.edu/web/members/help/adverse.html#instruct)).

**BDI #9**: Subjects are to circle the number next to the item or items that describe(s) themselves the best.
0    I don't have any thoughts of harming myself.
1    I have thoughts of harming myself, but I would not carry them out.
2a   I feel I would be better off dead.
2b   I feel my family would be better off if I were dead
3a   I have definite plans about committing suicide.
3b   I would kill myself if I could.

- - If the subject endorses a **0** for this item, **no further action is required**.
- If the subject endorses a **1 or 2** for this item, **offer the Community Sources for Psychological Treatment list**, saying that you noticed on their questionnaires that they endorsed items showing that they are feeling distressed. As long as the subject has not circled a 3 for this item, it is not necessary to obtain further assistance unless a 5 is endorsed on the MASQ suicide item. The Community Sources for Psychological Treatment list is available in control rooms or in the lab "Members" section at [http://psyphz.psych.wisc.edu/members/help/comm_trtmt.html](https://psyphz.psych.wisc.edu/web/members/help/comm_trtmt.html) - you will need to login with your UW NetID and password.
- If the subject endorses a **3** for this item, **obtain further assistance** (see below for [instructions](https://psyphz.psych.wisc.edu/web/members/help/adverse.html#instruct).) Note that the subject may have circled a 3 and a 2. Even if another number is also circled, you must obtain further assistance.

**MASQ**: Subjects are to rate how much they have felt or experienced the things described in items on the questionnaire. 1 = not at all -- 5 = extremely

_______ Thought about death or suicide. (item #61, short form; item #89, standard form)

- If the subject endorses a **1** for this item, **no further action is required**.
- If the subject endorses a **2 or 3** for this item, **offer the Community Sources for Psychological Treatment list**, saying that you noticed on their questionnaires that they endorsed items showing that they are feeling distressed. If the subject’s response is less than a 4 for this item, it is not necessary to obtain further assistance unless a 2 or 3 is endorsed on the BDI suicide item. The Community Sources for Psychological Treatment list is available in control rooms or in the lab "Members" section at [http://psyphz.psych.wisc.edu/members/help/comm_trtmt.html](https://psyphz.psych.wisc.edu/web/members/help/comm_trtmt.html).
- If the subject endorses a **4 or 5** for this item, **obtain further assistance** (see below for instructions.)

Procedures for obtaining further assistance with subjects who indicate suicidality:

Your first step should be calling lab members who have clinical training. There should be a current phone list in the control rooms or in the Brogden lab kitchen. These lab members include:

HyeJeen Lee: cell (preferred) - 628-2178; work - 262-1567; home - 298-0288
Tim Salomons: work - 263-1968; home - 442-9913

If you cannot reach any of these lab members, call the Psychology Clinic on-call therapist at 657-2889. He or she has documentation on how to handle such a situation.

The lab member or on-call therapist will assess the subject’s risk for self-harm. Ideally, this person will be able to talk to the subject face-to-face. If the lab member or on-call therapist can be reached only by phone, do the following:

- Once the experimental session is completed, have the lab member/therapist wait on the phone while you indicate to the subject that you noticed on their questionnaires that they endorsed items showing distress, and that in such instances you are obligated to have a therapist associated with the lab speak to the subject. You may say encouraging remarks like, "It should just take a little bit of your time." If the subject refuses, encourage them to take the phone call. If they refuse twice, finish the session with the subject and discuss your options with lab member or on-call therapist. Document the occurrence and inform your supervisors and Richie immediately.

## General Instructions

- To document an occurrence, indicate the date and time of the incident and all who were present. Describe the incident with as much objective information as possible (signs and symptoms revealed by the study participant, what you said and did in response, the participant’s reactions to your response). Indicate what you did to ensure the safety of the subject.
- Always inform your supervisors of any adverse reaction in a study participant, however minor.
- Do not hesitate to request help from others in the lab, even lab members you do not know by name. There is a list of emergency contact numbers in each control room and at [https://psyphz.psych.wisc.edu/web/members/help/emergency_contact.html](https://psyphz.psych.wisc.edu/web/members/help/Emergency_Numbers.pdf)  (log in using your UW netID & password). You may also download a [PDF version](https://psyphz.psych.wisc.edu/web/members/help/Emergency_Numbers.pdf).
- REMEMBER: You can always telephone graduate students or full-time staff members at home for help. Reasons why you might solicit help:
  1. You cannot reach your supervisor or Richie.
  2. You need help assessing the nature of the problem.
  3. You need help determining what actions to take.
- Information about our subjects should remain confidential. Do not reveal details about study participants, such as name, address, phone number or other identifying information, to non-essential people.  Of course Richie or the on-call therapist should be given this information if needed.
- If something occurred that warrants contacting Richie, these procedures should be followed:
  - Check the bulletin board outside of Isa's office (Room 379 Brogden) and see if there is a sheet indicating Richie’s whereabouts. Whenever Richie is out of town, there is a sheet indicating his whereabouts with information about how to contact him.
  - If he is out of town, call the number that is listed and if he is not there, leave a detailed message. Remember to respect the privacy of participants. (For long distance, dial 8 rather than 9 before the number). At your first available opportunity, also send an email to Richie with detailed information about the occurrence. **Do not include the subject�s name and phone number** in your voice message or email; rather, let Richie know where you and your supervisor can be reached.
  - If Richie is in town (as indicated by the absence of a "Whereabouts" sheet outside of Room 379, call his cell (345-8336) or home phone (256-5376). If you cannot reach him, follow the procedures described above for leaving a message. Isa/Donna should also be notified immediately as well (see [numbers at the top of this document](https://psyphz.psych.wisc.edu/web/members/help/adverse.html#contact)).
- Note that many of the procedures described in this document will vary slightly for the different studies in the lab. It is important to anticipate how various occurrences will be handled based on the particulars of your own experiment.
- Control rooms should always be equipped with this list of instructions, a current phone list, and a supply of Community Sources for Psychological Treatment lists.

The following list of counseling/consultation organizations is provided for participants who seek various forms of mental health care.  The services listed below are open to all persons regardless of religious or university affiliation; some have fees on a sliding scale based on an individual’s ability to pay.  Some of these organizations offer only short-term counseling and there may be waiting lists for appointments, so we encourage you to contact more than one.

| **Name & Location** | **Fee** | **Number** |
| --- | --- | --- |
| Wisconsin Psychiatric Institute & Clinics (Department of Psychiatry) 6001 Research Park Blvd. Madison, WI 53719 |  | 263-6100 |
| Family Services 128 E. Olin Avenue, Suite 100 Madison, WI 53713 | sliding scale | 252-1320 <http://www.fsmad.org/> |
| Lutheran Social Services  Mental Health Outpatient Clinic 5 Odana Court Madison, WI 53719 | sliding scale | 277-0610 <http://www.lsswis.org/> |
| Catholic Charities 30 South Franklin St. Madison, WI 53703 | sliding scale | 256-2358; <http://www.catholiccharitymadison.org/> |
| UW Psychology Clinic 1202 W. Johnson St., Room 351  Madison, WI 53706 | sliding scale | 262-5925 |
| Mental Health Center of Dane County 625 W. Washington Ave. Madison, WI 53703 |  | 280-2700 (substance abuse line)  280-2600 (24-hour Crisis Line) <http://www.mhcdc.org/> |
| Jewish Social Services 6434 Enterprise Lane Madison, WI 53719 |  | 278-1808 <http://www.jewishmadison.org/JSS/index.htm> |
| PICADA: Prevention-Intervention Center for Alcohol and Drug Abuse 128 East Olin Ave. Madison, WI 53713 | free screening and referral | 246-7606 <http://www.fsmad.org/PICADA/PICADA.htm> |
| NAMI (National Alliance of the Mentally Ill) 2059 Atwood Avenue Madison, WI 53704 |  | 249-7188 <http://www.nami.org/> |
| Tellurian UCAN, Inc.  300 Femrite Drive Madison, WI 53714 | sliding scale | 222-7311 <http://www.tellurian.org/> |

### HMO Options

Individuals may only be able to access the following services provided they are members of the particular HMO.

| Unity Health Plan | 362-3308 362-3310 |
| --- | --- |
| Physicians Plus Medical Group Counseling Referral Line | 282-8960 |
| Dean Medical Center | 252-8226 |
| Group Health Cooperative (GHC) | 257-1204 |

### UW Students Only

| University Health Services Counseling and Consultation Services 115 North Orchard Street  Madison, WI 53715 | 265-5600 |
| --- | --- |
| Campus Alcohol, Tobacco, & Other Treatment Services | 262-1744 |
| University Crisis Response Service | 265-6565 |

# Emergency Phone Numbers

| Richie Davidson | |
| --- | --- |
| Brogden | 263-2659 |
| Waisman | 265-8189 |
| Home | 256-5376 |
| Cell | 345-8336 |
| Isa Dolski | |
| Office | 263-0132 |
| Home | 232-1441 |
| Cell (rarely on) | 446-0932 |
| Donna Cole | |
| Office | 265-8107 |
| Home | 837-5809 |
| Cell (rarely on) | 438-3902 |

| Lab Main Line | |
| --- | --- |
| Brogden | 262-4443 |
| Emergency | |
| 911 | |
| Campus Police | |
| 262-2957 | |

## Psychological Assessment (for suicidal ideation)

| Hyejeen Lee | | | |
| --- | --- | --- | --- |
| Cell | | | 628-2178 |
| Office | | | 262-1567 |
| Home | | | 298-0288 |
| Tim Salomons | | | |
| Office | 263-1968 | | |
| Home | 442-9913 | | |
| Psychology Clinic | | | |
| Pager: | | 657-2889 | |
